# Assessment of the structure–activity relationship of analogs of the *Naegleria fowleri* enolase inhibitor HEX†

**DOI:** 10.1039/d5md00277j

**Published:** 2025-05-23

**Authors:** Samuel Kwain, James W. D. Morris, Jillian E. M. McKeon, Colm P. Roster, Monireh Noori, Aysiah R. Gibbs, Robert L. Stevenson III, Colin D. McMillen, Brian N. Dominy, James C. Morris, Daniel C. Whitehead

**Affiliations:** a Department of Chemistry, Clemson University Clemson SC 29634 USA dwhiteh@clemson.edu; b Department of Genetics and Biochemistry, Clemson University Clemson SC 29634 USA jmorri2@clemson.edu; c Eukaryotic Pathogens Innovation Center, Clemson University Clemson SC 29634 USA

## Abstract

The pathogenic free-living amoeba *Naegleria fowleri* causes primary amoebic meningoencephalitis (PAM), a highly fatal disease with limited treatment options, underscoring the urgent need for new therapeutics. Our previous studies identified (1-hydroxy-2-oxopiperidin-3-yl)phosphonic acid (HEX), an inhibitor of human enolase 2 (ENO2) involved in glucose metabolism, as a potent inhibitor of *N. fowleri* enolase (*Nf*ENO) with potent amoebicidal activity. In this study, we explored the structure–activity relationship (SAR) of HEX by modifying its hydroxamate and phosphonate functional groups, as well as introducing steric alterations to generate new analogs. Functional assays and computational-assisted SAR analysis provided insights into the impact of HEX modifications on *N. fowleri* agonism. Ultimately, the results of this study demonstrated that the activity of the HEX scaffold toward NfENO is rather sensitive to structural purturbations, confirming the necessity of both key functional groups – the hydroxamate and phosphonate – to maintain potency. Additionally, structural modifications of the parent compound into bicyclic analogs resulted in loss of biological activity ostensibly due to unfavorable steric interactions in the active site. These findings enhance our understanding of the activity of HEX's molecular architecture, and underscore potential limitations of further structural tuning efforts of the scaffold by means of SAR.

## Abstract 2

The SAR of the *N. fowleri* enolase inhibitor HEX revealed that the activity of the HEX scaffold toward *Nf*ENO is rather sensitive to structural perturbations, confirming the necessity of both the hydroxamate and phosphonate groups to maintain potency.

## Graphical abstract

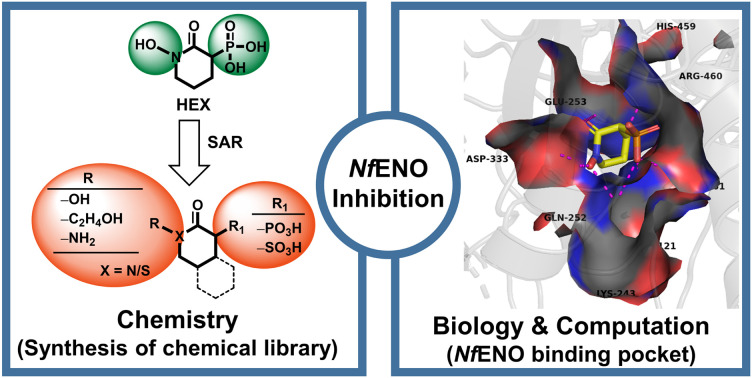

## Introduction

*Naegleria fowleri*, commonly known as the “brain-eating” amoeba, is a pathogenic free-living amoeba (pFLA) that thrives in warm freshwater. It causes primary amoebic meningoencephalitis (PAM), a rapidly fatal brain infection with a mortality rate exceeding 97%.^1^ Infection occurs when trophozoites in contaminated water enter the nasal cavity, migrate along the olfactory nerves, and cross the cribriform plate to reach the brain.^2,3^ Death primarily results from cerebral edema and increased intracranial pressure due to inflammation rather than direct brain tissue destruction.^2,4^ Although *N. fowleri* infections are rare, cases have been increasingly reported in the Southern United States and other regions worldwide.^5^ Symptoms typically appear 1–9 days post-exposure and include severe headache, fever, vomiting, and seizures.^4^ Due to its similarity to viral and bacterial meningitis, PAM is often misdiagnosed.^6^ In the U.S., treatment involves a combination of antimicrobials, antifungals, and anticancer agents, including dexamethasone, azithromycin, miltefosine, fluconazole, and amphotericin B.^7^ However, therapeutic success remains rare, and most patients succumb within 1–2 weeks of symptom onset.^8^ Given its high fatality rate, limited treatment options, and diagnostic challenges, the U.S. National Institute of Allergy and Infectious Diseases (NIAID) classifies *N. fowleri* as a category B emerging infectious pathogen.

Milanes *et al.* have demonstrated that glucose metabolism is critical for trophozoite viability, providing ATP and key metabolic intermediates, though its precise role in infection remains unclear.^9^ Their subsequent study identified a single enolase gene (*Nf*ENO) in *N. fowleri*, which shares 47% sequence identity with human enolase (ENO2).^10^ In humans, ENO2 is a key glycolytic enzyme that converts 2-phosphoglycerate (2-PG) to phosphoenolpyruvate (PEP), a crucial step in energy production, notably upregulated in cancer metabolism.^11^

**Fig. 1:**
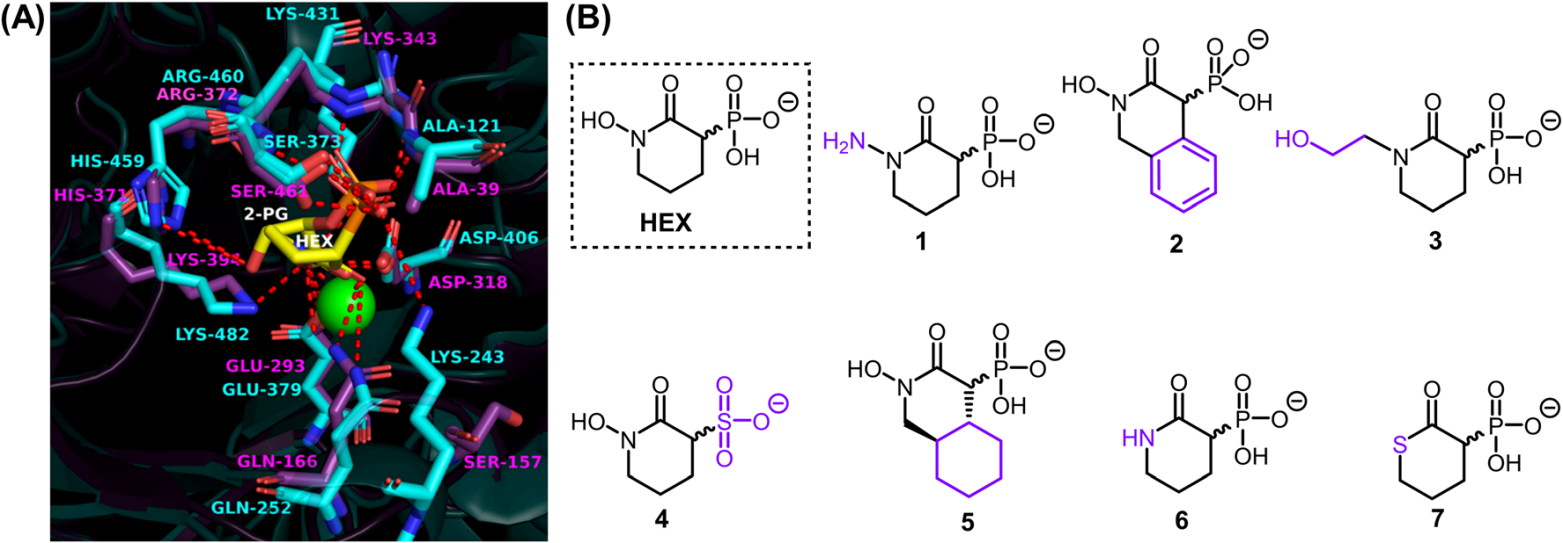
(A) Superimposed crystal structures of active site ENO2-HEX bound (51DZ, magenta) and 2-PG bound at the active site of *Nf*ENO (7UGH, cyan) showing conservation of residues at the binding pocket except for Lys243 (*Nf*ENO) and Ser157 (ENO). (B) Structure of HEX and 1–7.

Muller *et al.* previously demonstrated that the phosphonate compound (1-hydroxy-2-oxopiperidin-3-yl)phosphonic acid (HEX, Fig. 1) selectively inhibits human ENO2, effectively killing ENO1-deleted glioma cells and eliminating intracranial ENO1-deficient tumors in mice.^11^ Given the 47% sequence similarity between *Nf*ENO and human ENO2, and the fact that HEX can permeate the blood-cerebrospinal fluid (CSF) barrier to reach the brain, Milanes and colleagues screened HEX and related phosphonate ENO2 inhibitors against *Nf*ENO and *N. fowleri* trophozoites. Remarkably, HEX strongly inhibited *Nf*ENO (IC_50_ = 0.14 ± 0.04 μM) and was toxic to trophozoites in culture (EC_50_ = 0.21 ± 0.02 μM).^10^ A structural comparison of *Nf*ENO bound to 2-PG (PDB: 7UGH) and human ENO2 bound to HEX (PDB: 51DZ) revealed a highly conserved enzyme fold, with a root mean square deviation (RMSD) of 1.9 Å across 417 Cα atom pairs. The binding pockets of both enzymes are nearly identical, with all residues within 5 Å of HEX and 2-PG being conserved, except Lys243 in *Nf*ENO, which corresponds to Ser157 in ENO2 (Fig. 1A). Docking simulations further confirmed that HEX binds orthosterically to *Nf*ENO with a predicted binding affinity of −8.9 kcal mol^−1^, forming interactions akin to its binding with ENO2.^10^

In this study, we synthesized and characterized a series of HEX analogs (1–7) to investigate their structure–activity relationship. These analogs were assessed for their inhibitory activity against *Nf*ENO and their efficacy against *N. fowleri* trophozoites. Additionally, computational modeling was employed to analyze how these compounds might interact with *Nf*ENO, providing a deeper understanding of the underlying SAR. The findings from this work offer key insights into the molecular framework of HEX, paving the way for the development of next-generation *N. fowleri* inhibitors with improved efficacy and selectivity.

## Result and discusions

The analogs 1–7 were designed to probe specific questions regarding structural modifications of HEX. Analog 1 examines the effect of replacing HEX's hydroxyl group with an amine. Given the prevalence of arenes in biologically active compounds, analog 2 evaluates the significance of incorporating a benzene ring into the scaffold. Our previous study demonstrated that the simultaneous presence of hydroxyl and phosphonate groups on HEX is essential for *Nf*ENO agonist activity.^10^ To further investigate this, 3 systematically explores the impact of increasing the distance between these groups. Similarly, to better understand the phosphonate group's beneficial role, 4 replaces it with a structurally similar, negatively charged sulfonate. To assess the influence of an alicyclic ring, 5 introduces a cyclohexane moiety into HEX. Finally, analogs 6 and 7 probe the effect of the hydroxamate group's hydrogen-bond donating properties by substituting it with either an amide (6) or a thioate (7).

**Scheme 1:**
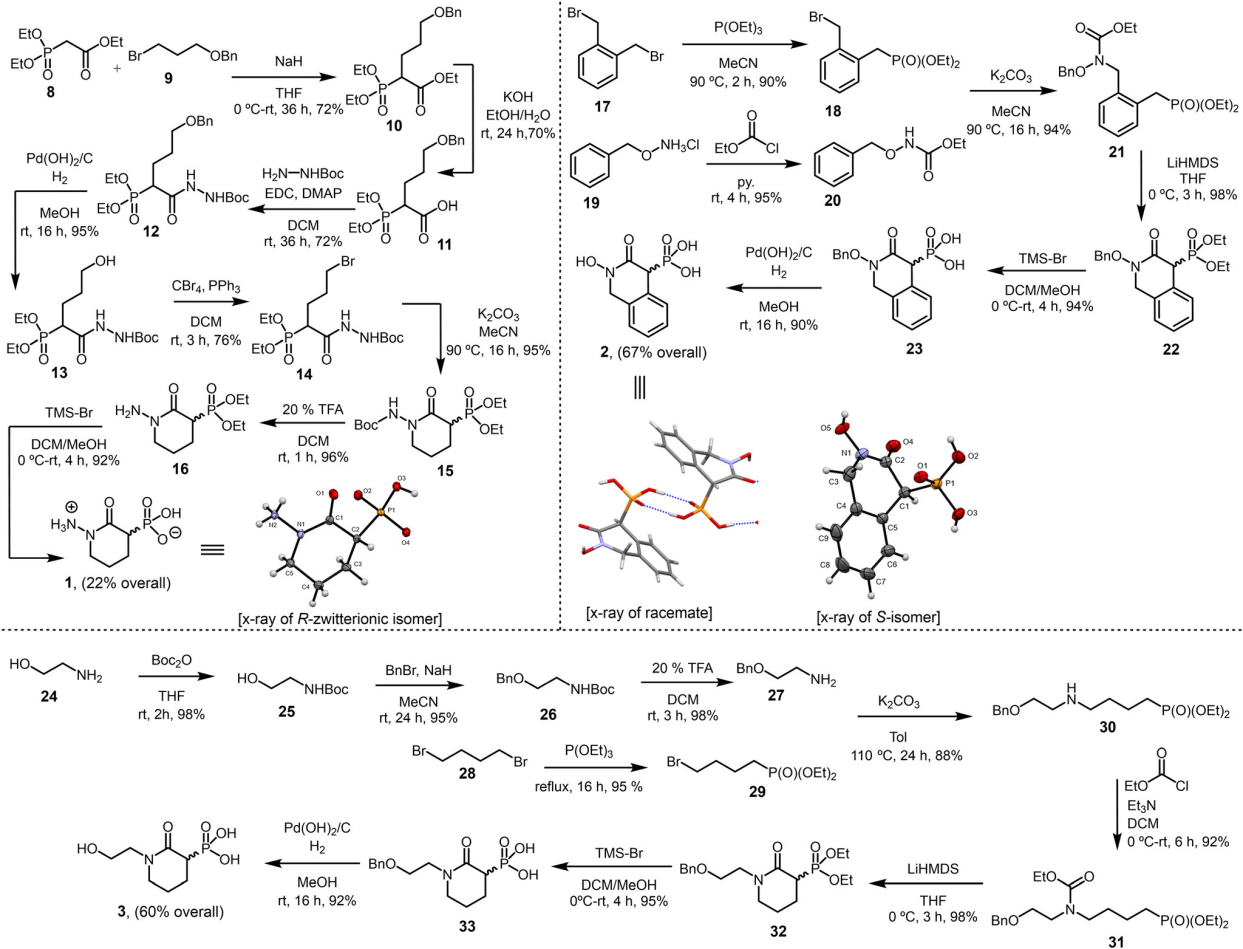
Synthesis of analogs 1–3.

We synthesized compound 1 by treating ethyl 2-(diethoxyphosphoryl)acetate (8) with ((3-bromopropoxy)methyl)benzene (9) to afford phosphonate derivative 10 in 72% yield. Hydrolysis of 10, followed by amidation with *tert*-butyl hydrazinecarboxylate, yielded Boc-protected hydrazine derivative 12 (72%). Hydrogenolysis of the benzyl-protecting group, followed by Appel reaction furnished bromohydrazine derivative 14 (76%). With 14 in hand, we pursued cyclization. We envisioned that the amide nitrogen would undergo an S_N_2 attack on the carbon bearing the bromo group, forming the cyclized product 15. Interestingly, treating 14 with potassium carbonate in refluxing MeCN efficiently yielded 15 (95%). This compound was obtained as a racemic mixture and could be separated by chiral HPLC but its enantiomers would rapidly be racemized in aqueous solution due to the highly acidic C3 α-proton – a hallmark of HEX analogs.^11^ The final steps of the synthesis included Boc deprotection with trifluoroacetic acid and phosphonate ester cleavage with bromotrimethylsilane, leading to the final product (1) with an overall 22% yield. Notably, 1 crystallized successfully, and its X-ray structure confirmed the (*R*)-isomer as a zwitterion (Scheme 1), crystallizing as a monohydrate in the chiral space group *P*2_1_2_1_2_1_.

We synthesized compound 2 through a sequence of high-yielding transformations. First, bis(bromomethyl)benzene (17) underwent a Michaelis–Arbuzov reaction to afford bromophosphonate derivative 18 (90%). In parallel, *O*-benzylhydroxylamine salt (19) was reacted with ethyl chloroformate to yield carbamate 20 (95%). The coupling of 18 with 20 produced carbamate derivative 21 (94%). Cyclization of 21 with LiHMDS, followed by phosphonate ester cleavage with TMS-Br, yielded hydroxamate 23 (94%). Finally, hydrogenolysis furnished the final product (2) with an overall 67% yield. X-ray crystallography confirmed it as a racemic mixture (Scheme 1), crystallizing in the centrosymmetric space group *P*2_1_/*n*.

We synthesized compound 3 through a concise sequence of reactions. First, 2-(benzyloxy)ethan-1-amine (27, obtained in three steps) reacted with diethyl (4-bromobutyl)phosphonate (29) to yield diethyl (4-((2-(benzyloxy)ethyl)amino)butyl)phosphonate (30) in 88% yield. Treatment of 30 with ethyl chloroformate, followed by cyclization with LiHMDS, phosphonate ester cleavage, and hydrogenolysis, delivered the final product (3) with an overall yield of 60% (Scheme 1).

**Scheme 2:**
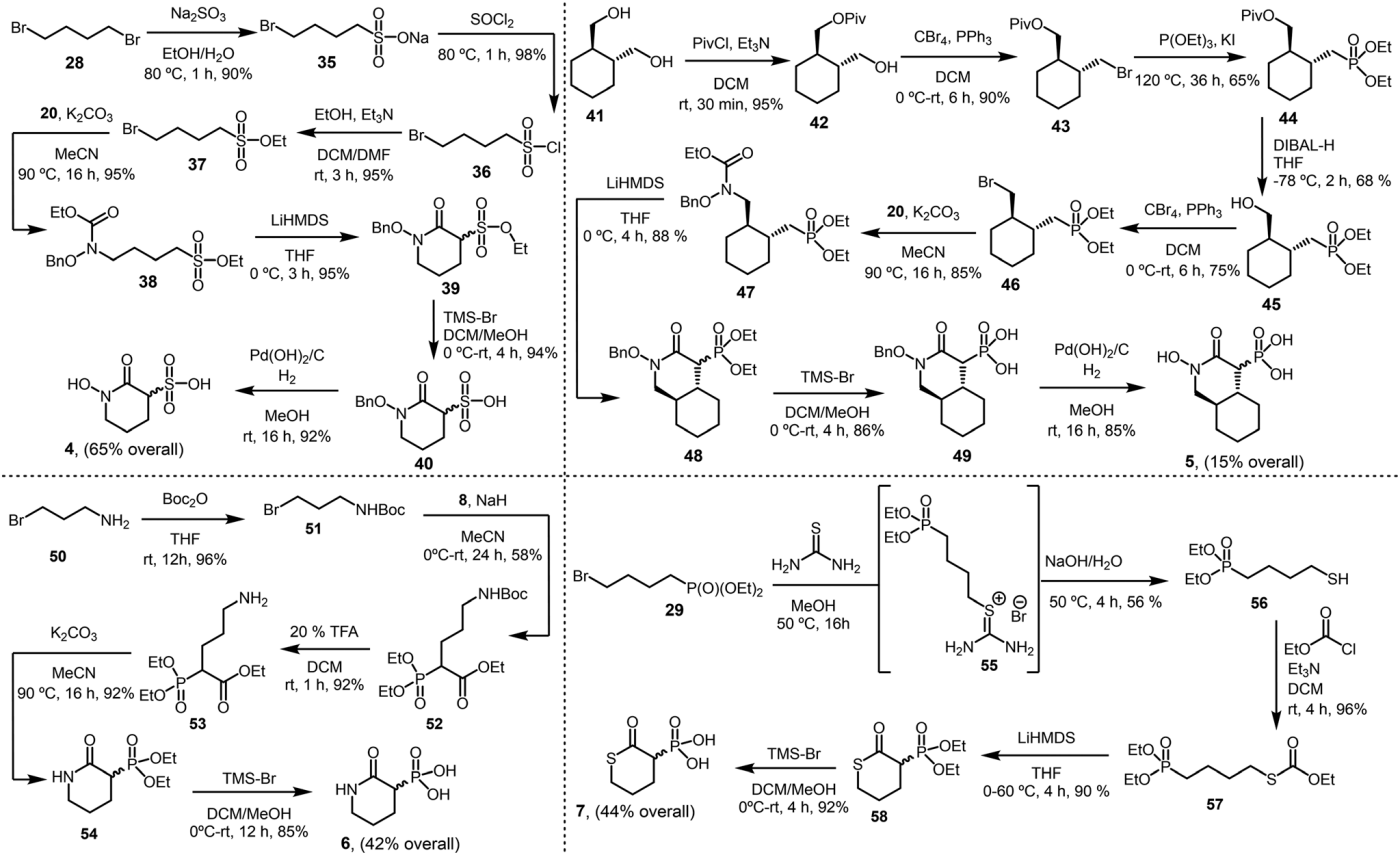
Synthesis of analogs 4–7.

We synthesized compound 4 by first preparing ethyl 4-bromobutane-1-sulfonate (37) through a three-step process following an established synthetic route.^12,13^ The reaction of 37 with carbamate 20 (see Scheme 1), followed by cyclization using LiHMDS, sulfonate ester cleavage, and hydrogenolysis, yielded the final product (4) with an overall yield of 85% (Scheme 2).

For compound 5, we treated ((1*R*,2*R*)-cyclohexane-1,2-diyl)dimethanol (41) with pivaloyl chloride to obtain the mono-protected pivalate derivative 42 (95%). Compound 42 was then subjected to Appel conditions to produce bromopivalate 43, which underwent a Michaelis–Arbuzov reaction to afford the pivalate-protected phosphonate intermediate 44. Initial attempts to remove the pivaloyl group from 44 – using various hydrolysis conditions such as NaOH, KOH, or K_2_CO_3_ in methanol; dilute HCl or H_2_SO_4_ in methanol; nucleophilic cleavage with aqueous ammonia; and reductive cleavage with lithium aluminum hydride (LiAlH_4_) – were unsuccessful, as all returned the starting material. Notably, treating 44 with di-iso-butylaluminum hydride (DIBAL-H) successfully afforded the desired phosphonate derivative 45 (68%). Subsequent transformation of 45 into bromo-phosphonate 46 and its reaction with 20 led to the carbamate derivative 47 (85%). The final steps included cyclization, phosphonate ester cleavage, and hydrogenolysis to deliver the final product (5) with an overall yield of 15% (Scheme 2).

To obtain compound 6, we reacted *tert*-butyl(3-bromopropyl)carbamate (51) with 8, followed by Boc deprotection, yielding the amino derivative 53 (92%). Thermally induced cyclization, followed by phosphonate ester cleavage, furnished the final product (6) with an overall yield of 42% (Scheme 2).

For compound 7, we treated 29 with thiourea, followed by hydrolysis, to obtain diethyl (4-mercaptobutyl)phosphonate 56 (56%). The reaction of 56 with ethyl chloroformate yielded the carbonothioate derivative 57 (96%). Notably, cyclization of 57 using LiHMDS in refluxing THF produced the thiopyran-2-one derivative 58 (90%). Subsequent phosphonate ester cleavage afforded the final product (7) with an overall yield of 44% (Scheme 2).

### SAR studies

To assess the impact of structural modifications on the biological activity of HEX derivatives, we tested the new analogs (1–7) against *N. fowleri* trophozoites and recombinant *Nf*ENO. All compounds were confirmed to be over 95% pure by proton NMR. Table 1 summarizes the functional responses of these newly synthesized derivatives compared to the reference agonist HEX.

**Table 1:** Effect of HEX derivatives 1–7 on *Nf*ENO and *N. fowleri*

Compound	Structure	*Nf*ENO IC_50_ (μM)	*N. fowleri* EC_50_ (μM)	Human cell line CC_50_ (μM)
HEX	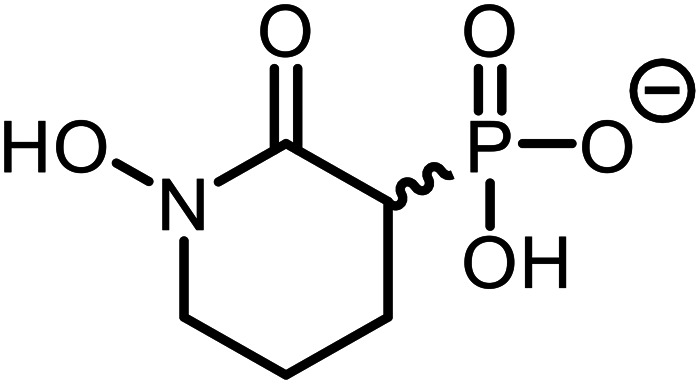	0.14 ± 0.04	0.21 ± 0.02	>100
1	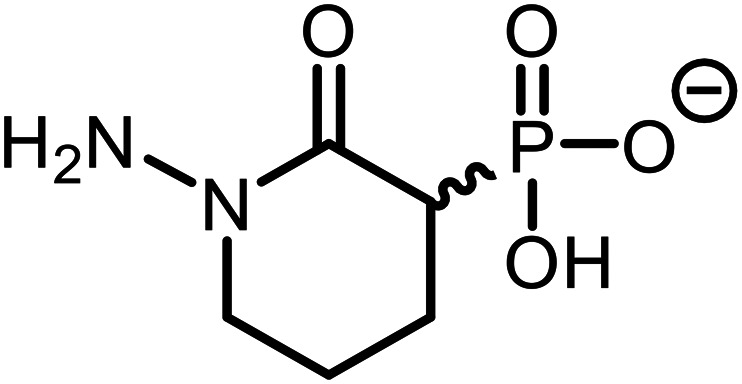	>25	>25	ND
2	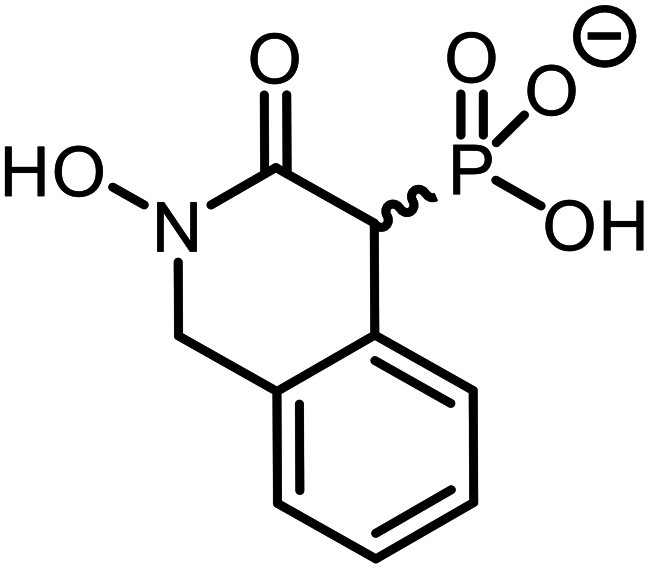	>25	>25	>25
3	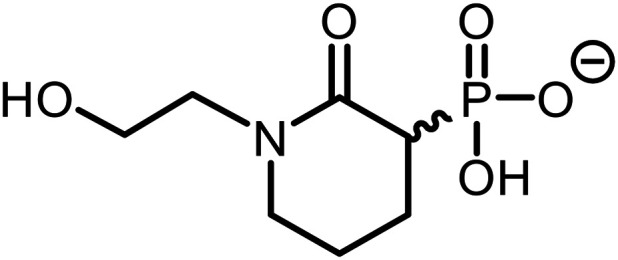	>25	>25	ND
4	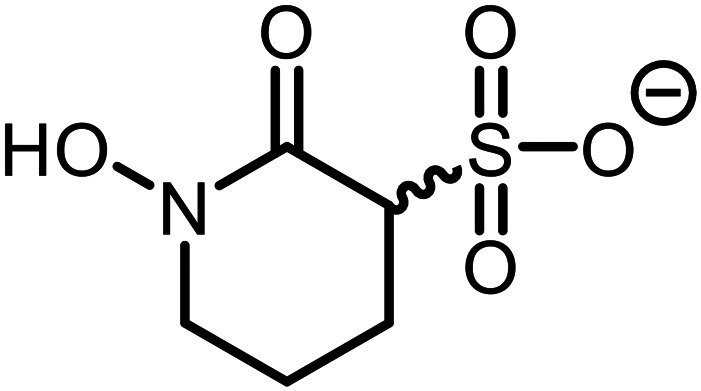	>25	>25	ND
5	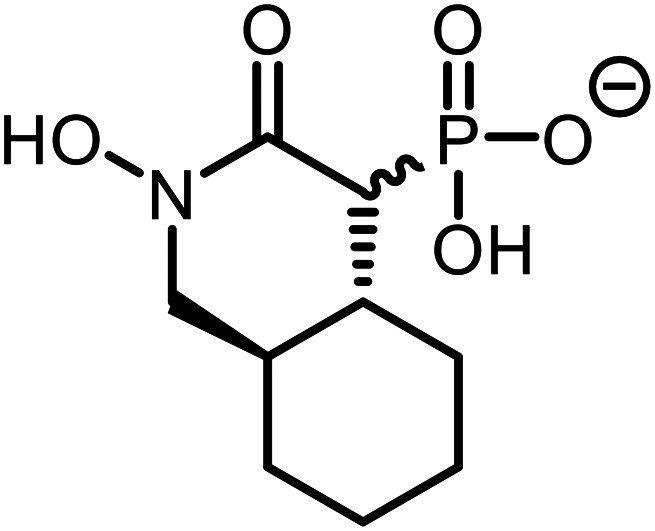	>25	>25	ND
6	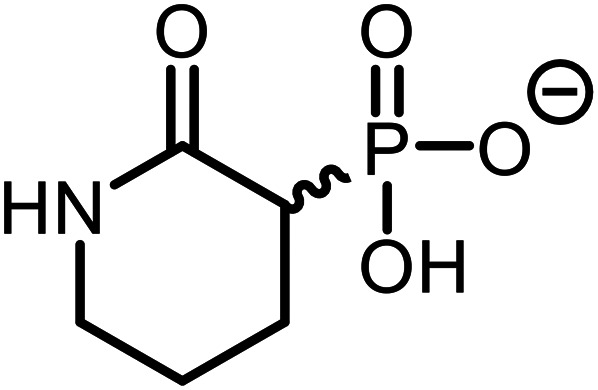	>25	>25	ND
7	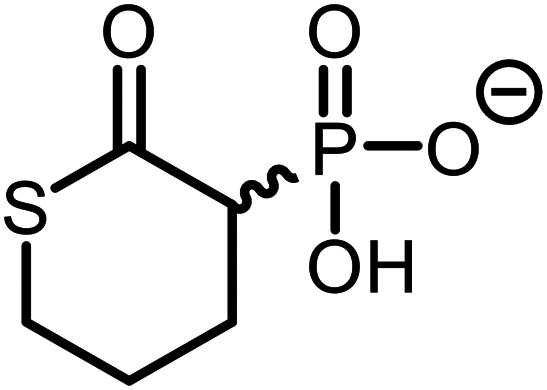	>25	>25	ND

**Fig. 2:**
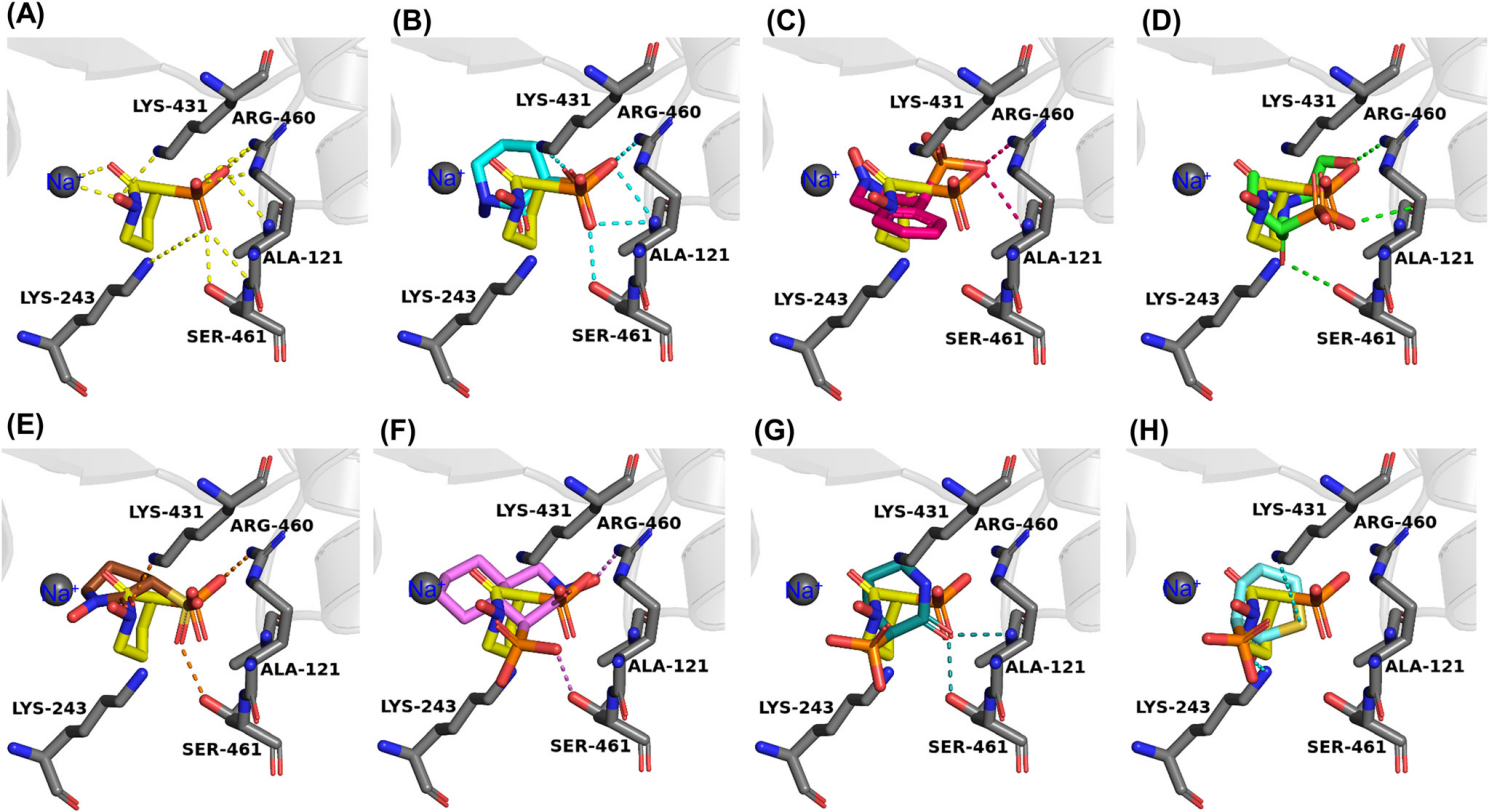
(A) Binding pose of *Nf*ENO–HEX complex (binding energy (BE) = −8.9 kcal mol^−1^). (B) Binding pose of *Nf*ENO–1 (BE = −3.2 kcal mol^−1^). Replacement of hydroxyl with amine (1, cyan sticks) do not overlap with HEX (yellow sticks) inside the binding site. (C) Binding pose of *Nf*ENO–2 (BE = −3.8 kcal mol^−1^). Installing an arene (2, pink sticks) disrupts proper fit with HEX (yellow sticks). (D) Binding pose of *Nf*ENO–3 (BE = −2.9 kcal mol^−1^). Altering the distance between hydroxyl and phosphonate groups (3, green sticks) disrupts proper fit with HEX (yellow sticks). (E) Binding pose of *Nf*ENO–4 (BE = −3.4 kcal mol^−1^). Replacement of phosphonate with sulfonate (4, brown sticks) disrupts proper fit with HEX (yellow sticks). (F) Binding pose of *Nf*ENO–5 (BE = −3.1 kcal mol^−1^). Installing an alicyclic ring (5, magenta sticks) disrupts proper fit with HEX (yellow sticks). (G) Binding pose of *Nf*ENO–6 (BE = −3.4 kcal mol^−1^). Replacement of hydroxymate with amide (6, green sticks) disrupts proper fit with HEX (yellow sticks). (H) Binding pose of *Nf*ENO–7 (BE = −2.8 kcal mol^−1^). Replacement of hyroxymate with thioate (7, cyan sticks) disrupts proper fit with HEX (yellow sticks).

Replacing the hydroxyl group with an amine (1) significantly reduced activity compared to HEX, likely due to differences in hydrogen-bonding capacity despite their similar physicochemical properties and size. The zwitterionic nature of 1, as revealed by its X-ray crystal structure, suggests that protonation of the amine may shift its p*K*_a_ relative to HEX under physiological conditions, potentially reducing cell permeability and thereby limiting activity against *Naegleria fowleri*. Furthermore, the presence of a hydrazinium cation in 1, in place of the hydroxamate group in HEX, introduces electrostatic changes that may impair chelation of the catalytic sodium cation in the *Nf*ENO active site – another likely contributor to the loss of activity. This assertion was further supported by the SAR analysis aided by computational modeling (Fig. 2A and B).

While aromatic rings are commonly found in biologically active compounds – facilitating membrane permeability and hydrophobic interactions that enhance drug–receptor binding – the incorporation of a benzene ring (2) did not improve *Nf*ENO agonist activity.

Further modifications in size and polarity were not well tolerated by either the receptor or the amoeba as increasing the distance between the hydroxyl and phosphonate groups (3), replacing the phosphonate with a sulfonate (4), or increasing steric demand and lipophilicity by introducing an alicyclic ring (5) did not enhance potency.

Similarly, replacing the hydroxamate group with either an amide (6) or a thioate (7) had no effect on *Nf*ENO at 100 μM. This underscores the crucial role of the hydroxamate group's hydrogen-bond donating properties in *Nf*ENO agonist activity.

### Computational analysis

To gain insights into the key interactions between *Nf*ENO and the newly developed ligands, and to further rationalize the underlying SAR, we analyzed the putative binding poses of representative ligands using molecular docking. HEX binds orthosterically to *Nf*ENO with a predicted binding affinity of −8.9 kcal mol^−1^, forming key interactions similar to those observed with ENO2.^10^ The binding pose analysis revealed that the carbonyl and hydroxamate moieties chelate the catalytic Na^+^ cation, while the anionic phosphonate forms a salt bridge with Arg460. Additional hydrogen bonds are formed with Lys243, Lys431, Ser461, and Ala121 (Fig. 2A).

The HEX derivatives (1–7) dock within the *Nf*ENO active site with binding affinities ranging from −2.8 to −3.8 kcal mol^−1^, which suggests less-favorable interactions with the binding pocket of *Nf*ENO than the parent molecule, HEX. This observation is consistent with the lackluster biological activity of 1–7. Notably, modifications such as replacing the hydroxyl with an amine (1), increasing the distance between the hydroxyl and phosphonate groups (3), and substituting the phosphonate with sulfonate (4) induce both electrostatic and conformational changes within the binding pocket. These alterations result in binding poses that do not overlap with HEX, precluding the carbonyl and hydroxamate moieties from chelating the catalytic Na^+^ cation (Fig. 2B, D and E).

Introducing an arene (2) or an alicyclic group (5) increases steric hindrance within the binding pocket, disrupting the proper fit of these ligands in the HEX-binding site. Consequently, their binding poses diverge from HEX, ultimately preventing the carbonyl and hydroxamate moieties from chelating the catalytic Na^+^ cation (Fig. 2C and F).

Similarly, replacing the hydroxamate group with an amide (6) or a thioate (7) induces an almost 180-degree rotation of the ligands within the binding pocket. This reorientation not only disrupts the chelation of the catalytic Na^+^ cation by the carbonyl and hydroxamate moieties but also prevents the anionic phosphonate groups from forming a salt bridge with Arg460 (Fig. 2G and H).

## Experimental section

### Chemistry

#### General information

Unless stated otherwise, all solvents were purified and dried according to standard methods prior to use. All reagents were purchased from commercial sources and used without purification unless otherwise noted. Unless stated otherwise, all reactions were performed under an inert atmosphere of argon in flame-dried glassware with magnetic stirring. All water and aqueous solutions were made using deionized (DI) water. Flash column chromatography was carried out using ZEOCHEM silica gel (40–63 μm). Analytical and preparative thin-layer chromatography (TLC) were performed on Sorbtech silica G TLC plates using UV light as visualizing agent, an ethanolic solution of phosphomolybdic acid and basic aqueous solution of potassium permanganate as developing agents. ^1^H and ^13^C NMR including 2D NMR spectra were obtained using Bruker avance 300 and 500 MHz spectrometers. Chemical shifts are reported in parts per million (ppm). Spectra are referenced to residual solvent peaks. The following abbreviations were used to designate multiplicities: s = singlet, d = doublet, t = triplet, q = quartet, p = pentet, sx = sextet, sep = septet, m = multiplet, br = broad. Infrared spectroscopy data were collected using an IR Affinity-1S instrument (with MIRacle 10 single reflection ATR accessory), and peaks are described as strong (s), moderate (m), and weak (w). All known compounds were characterized by ^1^H and ^13^C NMR and are in complete agreement with samples reported elsewhere. All new compounds were characterized by ^1^H, ^13^C and 2D NMR, ATR-FTIR, HRMS, XRD, and melting point (where appropriate). HRMS data were collected using an instrument equipped with electrospray ionization in positive mode (ESI+) and a time-of-flight (TOF) detector. Crystallographic data were collected using a Bruker D8 Quest diffractometer, with complete crystallographic details reported in the ESI.†

### Molecular modeling

The chemical structures of HEX and the analogs 1–7 were drawn with ChemOffice professional 19 suite (PerKinElmer, Waltham, MA), and three-dimensional (3D) structures were generated with VeraChem Vconf (VerChem LLC, Germantown, MD). The 3D structures were optimized with Gaussian 16 suite (Gaussian Inc., Wallingford, CT) using Density Functional Theory (DFT), employing the B3LYP/6-311G (d,p) level of theory.^14,15^ The 3D crystal structure of *Nf*ENO (PDB 7UGH) was retrieved from the RCSB protein data bank. The protein was then prepared for the docking analysis by first removing co-crystallized ligands, heteroatoms, and water molecules using Pymol Molecular Graphics 2.0 (Schrödinger LLC, New York, NY). The optimized ligands and the protein were further prepared using AutoDock Tools (The Scripps Research Institute, La Jolla, CA) to convert all structures into pdbqt formats. The grid box was prepared around the region of the active site of the protein. The size of the grid box was kept at 48, 52, and 52 for *X*, *Y*, and *Z*, respectively, with the center of the grid box maintained at −18.910, −1.626, and −15.343, respectively, for *X*, *Y*, and *Z*. The molecular docking studies were carried out *in vacuo* with AutoDock vina using specific docking parameters and scoring functions.^16^ The binding affinities of ligands were measured in kcal mol^−1^ as a unit for a negative score.^15^ The binding conformation with the highest negative value was taken as the best pose for the corresponding protein–ligand complex. Subsequently, the best binding pose of each complex was analyzed using Pymol and Discovery Studio (Dassault Systèmes, Waltham, MA) to reveal the protein–ligand interactions.

### *Nf*ENO assays

Using the full-length protein (NF0118810, AmoebaDB, https://amoebadb.org/amoeba/app/), an *E. coli* codon-optimized construct was designed to express a truncated version of *Nf*ENO (residues 44–512) to improve solubility.^10^*Nf*ENO was heterologously expressed in BL2(DE3)R3 Rosetta cells and expression induced with 0.5 mM isopropyl-β-d-1-thiogalactopyranoside (IPTG) overnight at 37 °C. Cells were lysed using a lysozyme and 10% Triton X-100 based lysis buffer and purified with Ni-NTA agarose beads (Qiagen). Activity assays were conducted in triplicate using a pyruvate kinase/lactate dehydrogenase-coupled assay. Approximately 75 nM of *Nf*ENO in assay buffer (100 mM HEPES, pH 8.5, 3.3 mM MgSO_4_, 120 mM KCl, 1.75 mM ADP, 1 U pyruvate kinase/lactate dehydrogenase, and 0.4 mM NADH) was added to black 96-well plates. For assays in the presence of inhibitor, compounds were resuspended in DMSO, added to the wells, and incubated with enzyme in assay buffer for 15 minutes at room temperature. DMSO was included as a vehicle control. Assays were initiated by addition of substrate solution (3.75 mM 2-PG) and fluorescence emission at 460 nm after excitation at 360 nm was monitored every 20 seconds for 4 minutes using a Biotek Synergy H1 microplate reader. The rate of fluorescence reduction was measured to monitor the conversion of NADH to NAD+. Kinetic analysis was performed with Prism 9.0 (GraphPad Software, San Diego, CA).

### *In vitro* growth inhibition of *N. fowleri* and amoebae viability

*N. fowleri* strain TY (ATCC 30107) trophozoites were routinely cultured in T-75 TC-treated flasks at 37 °C and 5% CO_2_ in media that has been previously described.^10^ All compounds described were reconstituted in DMSO for testing, with final concentrations kept at <1% in the assays. To test the inhibitory activity of the compounds on cell growth, 1 × 10^4^ cells per mL were seeded in white 96-well plates with 100 μM compound. Cells and compound were incubated at 37 °C and 5% CO_2_ for 48 hours. CellTiter-Glo reagent (Promega G7570) was then added to each well and luminescence was read using a BioTek Synergy H1 Microplate Reader. HEX2 was further tested at 25 μM given its modest activity observed at higher concentrations. All reactions were performed in technical triplicate. If growth inhibition was <50% at the concentration tested, EC_50_ was reported to be above that value.

To determine the amount of agent required to inhibit human cell growth 50% (the CC_50_ value), human embryonic kidney cells (ATCC HEK-293) were seeded at 5 × 10^4^ cells per well in 100 μL of Dulbecco's modified Eagle's medium in tissue culture-treated clear bottom black 96 well plates (Gibco). Cells were incubated with compounds for 48 hours, followed by addition of CellTiter Blue (Promega, Madison, WI, USA) After a 3 hour incubation and equilibration for 15 minutes at room temperature, fluorescence (Ex: 560 nm, EM: 590 nm) was measured and the percentage of growth inhibition was calculated using untreated controls.

### (1-Amino-2-oxopiperidin-3-yl)phosphonic acid


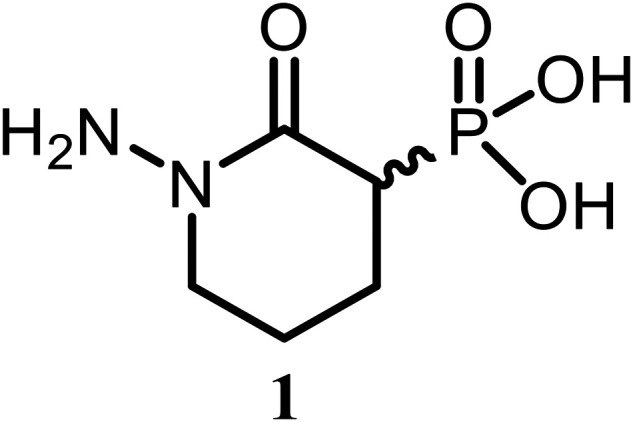
Off-white solid (92% yield); Mp: 206–207 °C; IR (neat): 3597 (w) 3328 (w), 3094 (m), 2983 (m), 1718 (m), 1456 (m), 1443 (m), 1421 (m), 1416 (m), 1392 (s), 1382 (s), 1261 (s), 1254 (m), 1186 (s), 1162 (m), 1088 (s), 1041 (m), 1016 (w), 935 (w), 908 (w), 877 (w), 865 (m), 776 (w), 748 (m), 686 (w), 625 (w), 617 (w), 577 (m) cm^−1^; ^1^H NMR (500 MHz, D_2_O) *δ* 3.62 (m, 4H), 3.06 (t, *J* = 6.6 Hz, 1H), 3.00 (t, *J* = 6.6 Hz, 1H), 2.16–2.05 (m, 1H), 2.03–1.97 (m, 1H), 1.87 (m, 1H); ^13^C{^1^H} NMR (126 MHz, D_2_O) *δ* 167.32 (d, *J* = 5.1 Hz), 49.69, 42.30 (d, *J* = 128.3 Hz), 22.12 (d, *J* = 4.0 Hz), 20.50 (d, *J* = 7.7 Hz); HRMS (ESI-TOF) *m*/*z*: [M + H]^+^ calcd for C_5_H_15_N_2_O_4_P 195.0535; found 195.0534.

### (2-Hydroxy-3-oxo-1,2,3,4-tetrahydroisoquinolin-4-yl)phosphonic acid


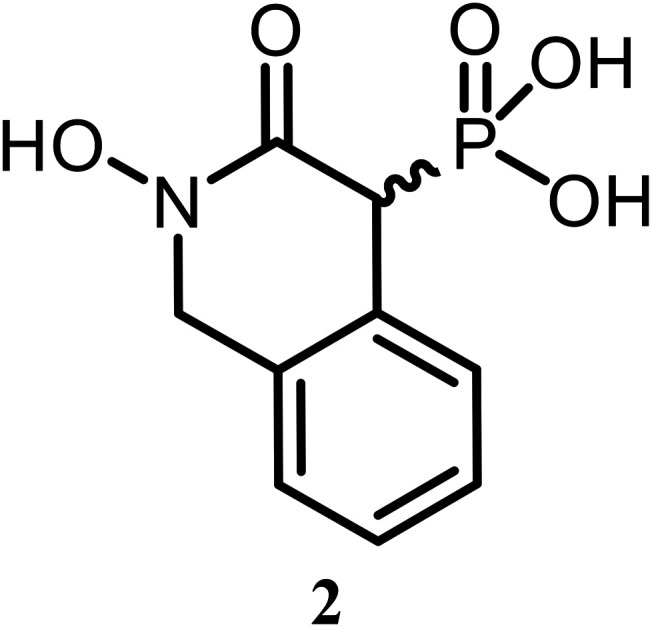
White solid (90% yield); Mp: 195–196 °C; IR (neat): 3591 (w) 3261 (w), 3097 (m), 2942 (m), 1716 (m), 1598 (m), 1583 (m), 1494 (m), 1476 (m), 1464 (m), 1453 (m), 1363 (s), 1343 (s), 1228 (s), 1214 (m), 1196 (s), 1178 (m), 1065 (s), 1042 (m), 1021 (w), 986 (w), 968 (w), 856 (w), 841 (m), 785 (w), 763 (m), 686 (w), 624 (w), 616 (w), 542 (m) cm^−1^; ^1^H NMR (500 MHz, DMSO-*d*_6_) *δ* 7.31–7.19 (m, 4H), 5.02 (dd, *J* = 14.8, 8.8 Hz, 1H), 4.48 (dd, *J* = 14.8, 3.1 Hz, 1H), 3.97 (d, *J* = 26.3 Hz, 1H); ^13^C{^1^H} NMR (126 MHz, DMSO-*d*_6_) *δ* 161.12 (d, *J* = 5.2 Hz, 1C), 132.49 (d, *J* = 4.8 Hz, 1C), 130.90 (d, *J* = 8.2 Hz, 1C), 129.22 (d, *J* = 4.1 Hz, 1C), 127.41, 126.95, 125.78, 54.42, 51.52 (d, *J* = 120.1 Hz, 1C); HRMS (ESI-TOF) *m*/*z*: [M + H]^+^ calcd for C_9_H_10_NO_5_P 244.0375; found 244.0372.

### (1-(2-Hydroxyethyl)-2-oxopiperidin-3-yl)phosphonic acid


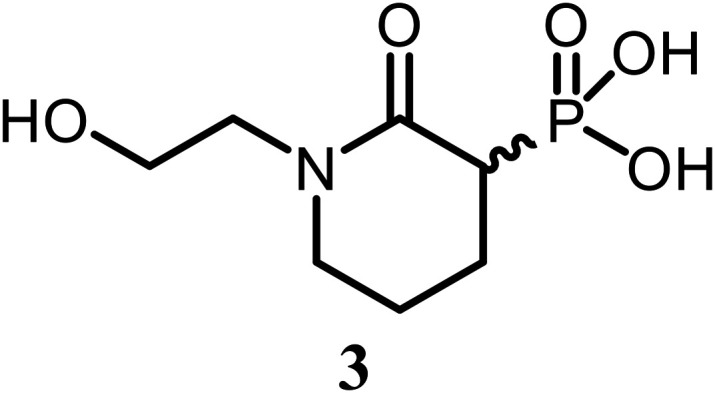
White solid (92% yield); Mp: 166–167 °C; IR (neat): 3424 (w) 3234 (w), 2962 (m), 1785 (m), 1546 (m), 1524 (m), 1486 (m), 1474 (m), 1453 (m), 1424 (m), 1374 (s), 1331 (s), 1232 (s), 1226 (m), 1182 (s), 1145 (m), 1075 (s), 1026 (m), 1017 (w), 975 (w), 923 (w), 824 (w), 812 (m), 774 (w), 721 (m), 656 (w), 632 (w), 612 (w), 562 (m) cm^−1^; ^1^H NMR (500 MHz, DMSO-*d*_6_) *δ* 3.49 (t, *J* = 6.2 Hz, 2H), 3.40 (m, 1H), 3.35 (d, *J* = 6.1 Hz, 2H), 3.27 (dt, *J* = 12.9, 6.2 Hz, 1H), 2.74 (tt, *J* = 6.8, 6.6 Hz, 1H), 1.94 (m, 3H), 1.64 (m, 1H); ^13^C{^1^H} NMR (126 MHz, DMSO-*d*_6_) *δ* 166.70 (d, *J* = 4.3 Hz), 58.94, 50.23, 49.23, 42.29 (d, *J* = 129.8 Hz), 23.00 (d, *J* = 3.7 Hz), 21.57 (d, *J* = 7.9 Hz); HRMS (ESI-TOF) *m*/*z*: [M + H]^+^ calcd for C_7_H_15_NO_5_P 224.0688; found 224.0686.

### 1-Hydroxy-2-oxopiperidine-3-sulfonic acid


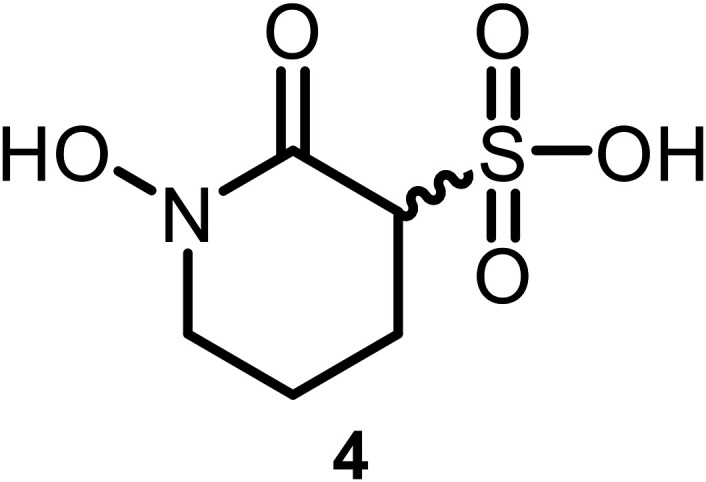
White solid (92% yield); Mp: 154–155 °C; IR (neat): 3586 (w) 3275 (w), 3092 (m), 2942 (m), 1702 (m), 1486 (m), 1474 (m), 1466 (m), 1453 (m), 1392 (s), 1362 (s), 1253 (s), 1234 (m), 1186 (s), 1162 (m), 1083 (s), 1042 (m), 1061 (w), 953 (w), 924 (w), 842 (w), 821 (m), 775 (w), 724 (m), 686 (w), 663 (w), 624 (w), 576 (m) cm^−1^; ^1^H NMR (500 MHz, D_2_O) *δ* 3.90–3.83 (m, 1H), 3.13–3.03 (m, 2H), 2.06–1.89 (m, 2H), 1.83–1.61 (m, 2H); ^13^C{^1^H} NMR (126 MHz, D_2_O) *δ* 170.01, 64.88, 50.88, 25.14, 21.04; HRMS (ESI-TOF) *m*/*z*: [M + H]^+^ calcd for C_5_H_9_NO_5_S 196.0280; found 196.0284.

### ((4*S*,4*a*R,8*aR*)-2-Hydroxy-3-oxodecahydroisoquinolin-4-yl)phosphonic acid


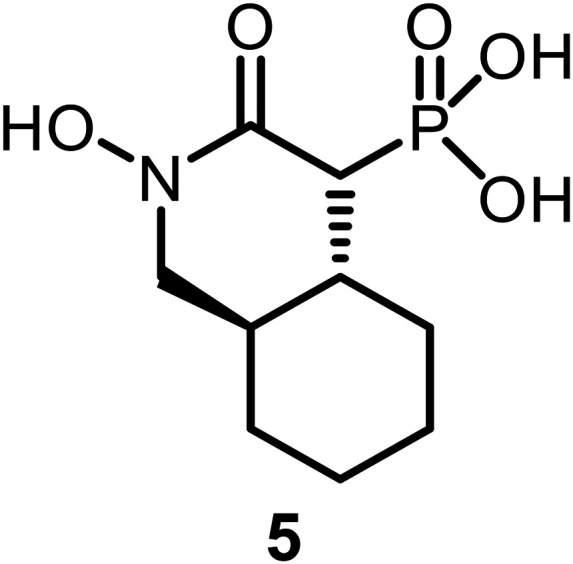
Off-white solid (85% yield); Mp: 141–142 °C; IR (neat): 3576 (m) 3235 (m), 3023 (m), 2992 (m), 1688 (m), 1492 (m), 1493 (m), 1461 (m), 1434 (m), 1383 (s), 1342 (s), 1231 (s), 1221 (m), 1196 (s), 1154 (m), 1075 (s), 1063 (m), 1066 (w), 982 (w), 974 (w), 853 (w), 842 (m), 763 (w), 741 (m), 686 (w), 665 (w), 643 (w), 562 (m) cm^−1^; ^1^H NMR (500 MHz, D_2_O) *δ* 3.38 (d, *J* = 6.4 Hz, 1H), 3.26 (d, *J* = 6.3 Hz, 1H), 2.51 (m, 1H), 2.29 (m, 1H), 1.67 (m, 4H), 1.19 (m, 3H), 1.04–0.99 (m, 2H); ^13^C{^1^H} NMR (126 MHz, D_2_O) *δ* 163.70, 56.38, 48.12 (d, *J* = 123.7 Hz), 39.05, 38.60, 32.50, 29.63, 25.63, 25.09; HRMS (ESI-TOF) *m*/*z*: [M + H]^+^ calcd for C_9_H_17_NO_5_P 250.0844; found 250.0843.

### (2-Oxopiperidin-3-yl)phosphonic acid


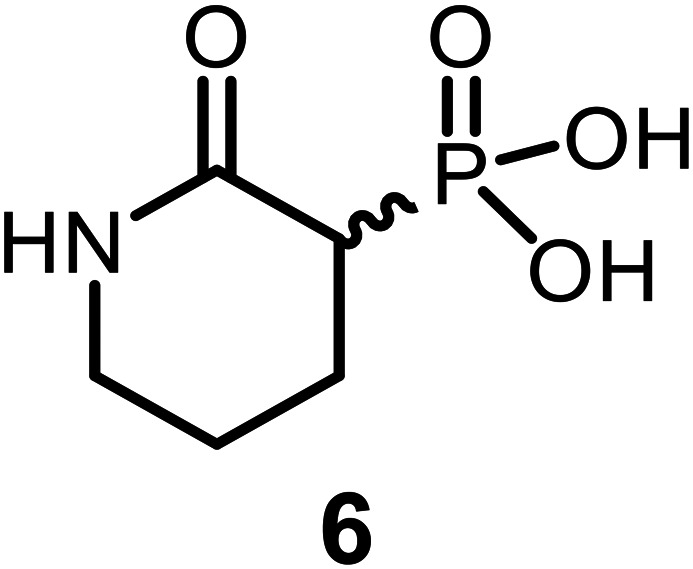
Pale yellow solid (85% yield); Mp: 135–136 °C; IR (neat): 3523 (w) 3419 (m), 3051 (m), 2983 (m), 1723 (s), 1481 (m), 1495 (m), 1434 (m), 1427 (m), 1397 (s), 1333 (s), 1270 (s), 1232 (m), 1195 (s), 1147 (m), 1088 (s), 1072 (m), 1068 (w), 994 (w), 967 (w), 825 (w), 815 (m), 786 (w), 743 (m), 686 (w), 642 (w), 658 (w), 575 (m) cm^−1^; ^1^H NMR (500 MHz, D_2_O) *δ* 3.14–3.01 (m, *J* = 6.0 Hz, 2H), 2.81 (dt, *J* = 26.6, 14.4 Hz, 1H), 1.96–1.83 (m, 2H), 1.84–1.77 (m, 1H), 1.80–1.68 (m, 2H), 1.55–1.44 (m, 1H); ^13^C{^1^H} NMR (126 MHz, D_2_O) *δ* 170.31, 41.58, 40.60 (d, *J* = 132.3 Hz, 1C), 21.75 (d, *J* = 3.9 Hz, 1C), 19.74 (d, *J* = 8.1 Hz, 1C); HRMS (ESI-TOF) *m*/*z*: [M + H]^+^ calcd for C_5_H_10_NO_4_P 180.0426; found 180.0427.

### (2-Oxotetrahydro-2*H*-thiopyran-3-yl)phosphonic acid


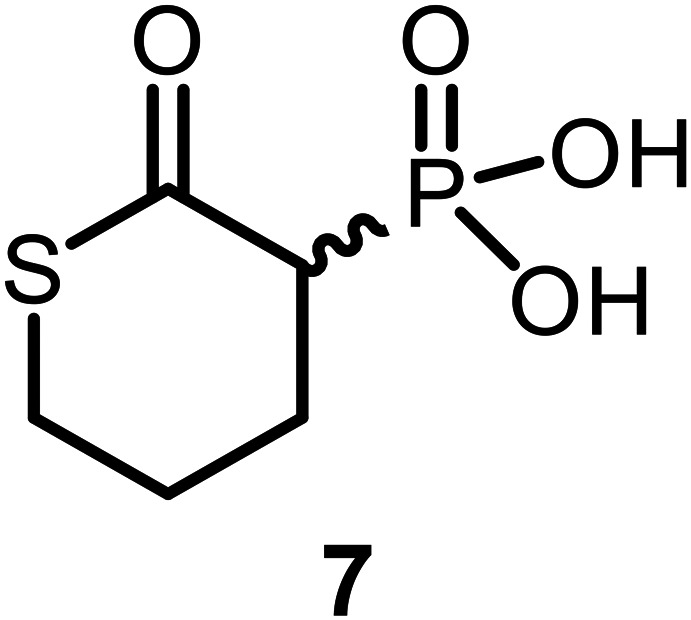
Pale yellow oil (92% yield); IR (neat): 3551 (w), 3034 (m), 2988 (m), 1745 (s), 1481 (m), 1491 (m), 1396 (s), 1324 (s), 1272 (s), 1248 (m), 1174 (s), 1087 (s), 1066 (m), 1031 (w), 982 (w), 956 (w), 843 (w), 837 (m), 756 (w), 734 (m), 696 (w), 612 (w), 615 (w), 540 (m) cm^−1^; ^1^H NMR (500 MHz, DMSO-d_6_) *δ* 2.71 (ddd, *J* = 22.3, 11.0, 4.1 Hz, 1H), 2.52–2.48 (m, 2H), 1.95–1.80 (m, 1H), 1.77 (m, 1H), 1.50 (m, 2H); ^13^C{^1^H} NMR (126 MHz, D_2_O) 172.34 (d, *J* = 4.9 Hz, 1C), 46.49 (d, *J* = 124.7 Hz, 1C), 29.86, 27.56 (d, *J* = 14.8 Hz, 1C), 25.95 (d, *J* = 4.3 Hz, 1C); HRMS (ESI-TOF) *m*/*z*: [M + H]^+^ calcd for C_5_H_9_O_4_PS 197.0037; found 197.0035.

## Conclusions

In conclusion, we have developed efficient syntheses of seven new structural analogs of the *N. fowleri* agonist HEX and evaluated them for biological activity against *Nf*ENO and *N. fowleri* parasites. These efforts revealed that modifying the hydroxamate and phosphonate functional groups or increasing steric demand in HEX did not enhance *Nf*ENO agonism. Computationally assisted SAR analyses further demonstrated that these structural modifications induce unfavorable electrostatic and conformational changes, disrupting the ligands' proper fit in the HEX-binding site. Although the newly developed HEX analogs did not improve *N. fowleri* agonism, this study provides valuable insights into the molecular architecture of HEX, guiding the design of next-generation *N. fowleri* agonists with enhanced efficacy and selectivity. The major conclusions from this study include confirmation that both the hydroxamate and phosphonate moieties are critically important for biological activity. Indeed, replacing these motifs with structurally similar functionalities (*i.e.* hydroxamate to hydrazide or amide and phosphonate to sulfonate) resulted in complete loss of biological activity. Additionally, the pursuit of bicyclic analogs was unproductive, most likely due to unfavorable steric interactions in the binding site of *Nf*ENO. These results underscore the rather limited structural space available for further tuning the biological activity of HEX in the context of *Nf*ENO inhibition.

## Author contributions

S. K.: data curation, formal analysis, investigation, methodology, supervision, writing – original draft (chemical synthesis, molecular docking); J. W. D. M.: investigation, methodology (chemical synthesis); J. E. M. M.: data curation, formal analysis, investigation, methodology (parasitology); C. P. R.: investigation, methodology (parasitology); M. N., A. R. G., R. L. S. III: investigation, methodology (chemical synthesis); C. D. M.: data curation, formal analysis, investigation, methodology, visualization, writing – review & editing (X-ray crystallography); B. N. D.: formal analysis, methodology, resources, supervision, validation (molecular docking); J. C. M.: conceptualization, formal analysis, funding acquisition, methodology, project administration, validation, writing – review & editing (parasitology); D. C. W.: conceptualization, formal analysis, funding acquisition, methodology, project administration, validation, writing – review & editing (chemical synthesis).

## Conflicts of interest

There is no conflict of interest to declare.

## Supplementary Material

MD-016-D5MD00277J-s001

MD-016-D5MD00277J-s002

## Data Availability

The data supporting this article have been included as part of the ESI.†
