# Supplementary material for: Assessment of the structure–activity relationship of analogs of the Naegleria fowleri enolase inhibitor HEX
Source: RSC Med Chem. 2025 May 23;16(8):3826–35. doi: 10.1039/d5md00277j (PMC12186339; doi:10.1039/d5md00277j)
Supplement: MD-016-D5MD00277J-s001 [file MD-016-D5MD00277J-s001.pdf]

Supplementary Information (SI) for RSC Medicinal Chemistry.
This journal is © The Royal Society of Chemistry 2025

Assessment of the structure-activity relationship of analogs of the *Naegleria fowleri* enolase inhibitor HEX

Samuel Kwain[1], James W.D. Morris[1], Jillian E. M. McKeon[2,3], Colm P. Roster[2,3], Monireh Noori[1], Aysiah Renee Gibbs[1], Robert L. Stevenson III[1], Colin D. McMillen[1], James C. Morris[2,3], Brian N. Dominy[1], Daniel C. Whitehead[1,3]

1. Department of Chemistry, Clemson University, Clemson, SC 29634, USA
2. Department of Genetics and Biochemistry, Clemson University, Clemson, SC 29634, USA
3. Eukaryotic Pathogens Innovation Center, Clemson University, Clemson, SC 29634, USA

Table of Contents

1.1 General methods …………………………………………………………...………… S3

1.2 Synthesis of compound 1 …………………………………………….…………..…… S4

1.3 Synthesis of compound 2 ……………………………………………...…….……….. S8

1. 4 Synthesis of compound 3 ………………………………………….……….....… S12

1.5 Synthesis of compound 4 ……………………………………….…….…..……… S16

1. 6 Synthesis of compound 5 ……………………………………………...…………… S19

1.7 Synthesis of compound 6 ……………………………………….………..………… S24

1. 8 Synthesis of compound 7 …………………………………………….…………..…… S27

2.0 X-ray crystallographic data ………………………………………….……………… S29

3.0 References ………………………………………………………….…...………… S32

4.0 NMR Spectra ………………………………………………………….………..……… S33

1. Chemistry

1.1 General methods

Unless stated otherwise, all solvents were purified and dried according to standard methods prior to use. All reagents were purchased from commercial sources and used without purification unless otherwise noted. Unless stated otherwise, all reactions were performed under an inert atmosphere of argon in flame-dried glassware with magnetic stirring. All water and aqueous solutions were made using deionized (DI) water. Flash column chromatography was carried out using ZEOCHEM silica gel (40-63 µm). Analytical and preparative thin-layer chromatography (TLC) were performed on Sorbtech silica G TLC plates using UV light as visualizing agent, an ethanolic solution of phosphomolybdic acid and basic aqueous solution of potassium permanganate as developing agents. ^{1}H and ^{13}C NMR including 2D NMR spectra were obtained using Bruker avance 300 and 500 MHz spectrometers. Chemical shifts are reported in parts per million (ppm). Spectra are referenced to residual solvent peaks. The following abbreviations were used to designate multiplicities: s = singlet, d = doublet, t = triplet, q = quartet, p = pentet, sx = sextet, sep = septet, m = multiplet, br = broad. Infrared spectroscopy data were collected using an IR Affinity-1S instrument (with MIRacle 10 single reflection ATR accessory), and peaks are described as strong (s), moderate (m), and weak (w). All known compounds were characterized by ^{1}H and ^{13}C NMR and are in complete agreement with samples reported elsewhere. All new compounds were characterized by ^{1}H, ^{13}C and 2D NMR, ATR-FTIR, HRMS, XRD, and melting point (where appropriate). HRMS data were collected using an instrument equipped with electrospray ionization in positive mode (ESI+) and a time-of-flight (TOF) detector.

1.2 Synthesis of compound 1

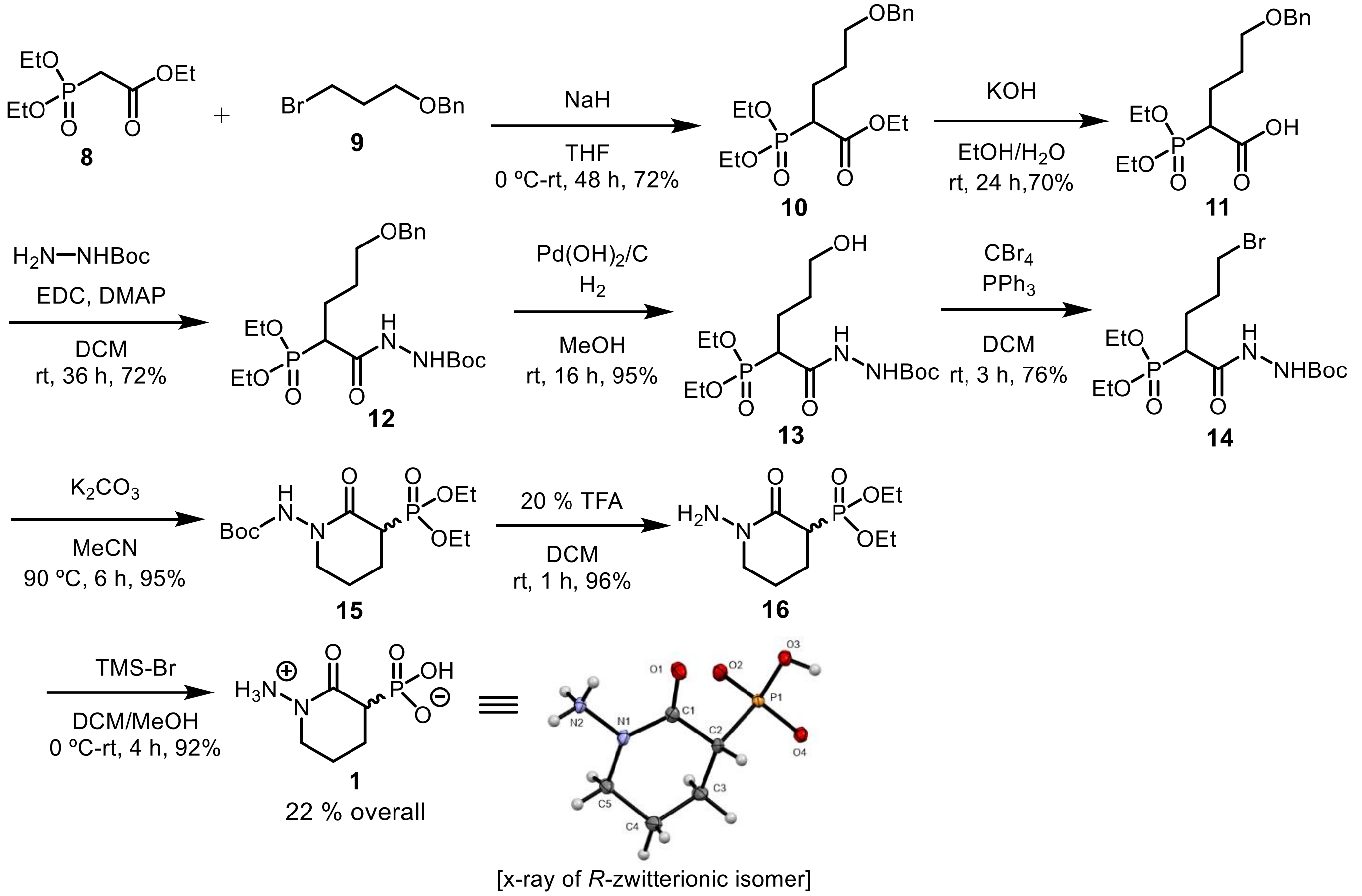

Scheme S1. Synthesis of **1**

ethyl 5-(benzyloxy)-2-(diethoxyphosphoryl)pentanoate. In a flame-dried 50 mL round-bottom flask equipped with a magnetic stir bar, ethyl 2-(diethoxyphosphoryl)acetate (**8**, 1 mmol) was dissolved in 5 mL of anhydrous THF at 0 °C. Sodium hydride (1.05 mmol) was then added, and the reaction mixture was stirred at 0 °C for 1 h. After that, ((3-bromopropoxy)methyl)benzene (**9**, 1 mmol) was added dropwise and the reaction mixture allowed to stir at room temperature for 48 h. Upon completion of the reaction, 50 mL of deionized water was added. The organic layer was extracted with ethyl acetate (3 × 50 mL), and the combined organic extracts were washed successively with 50 mL of water and 50 mL of saturated brine. The organic layer was dried and purified by flash chromatography on silica gel, using gradient elution from 100% hexanes to 20% hexanes in EtOAc. Compound **10** was obtained as a pale-yellow oil in 72% yield. ^{1}H NMR (500 MHz, $CDCl_3$) δ 7.34 – 7.19 (m, 5H), 4.44 (d, *J* = 2.9 Hz, 2H), 4.24 – 4.04 (m, 6H), 3.44 (tt, *J* = 6.2, 3.0 Hz, 2H), 3.01 – 2.88 (m, 1H), 2.41 (d, *J* = 9.3 Hz, 1H), 2.08 – 1.89 (m, 1H), 1.73 – 1.55

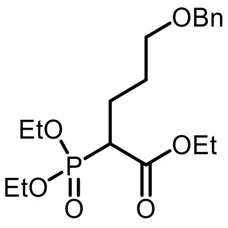

10

(m, 1H), 1.36 – 1.26 (m, 6H), 1.29 – 1.19 (m, 4H).; $^{13}C\{^1H\}$ NMR (126 MHz, $CDCl_3$) δ 169.10 (d, J = 4.7 Hz, 1C), 138.34, 128.29, 127.53, 127.48, 72.82, 69.39, 62.71 (d, J = 6.6 Hz, 1C), 62.60 (d, J = 6.8 Hz, 1C), 61.32, 45.40 (d, J = 131.1 Hz, 1C), 28.27 (d, J = 14.5 Hz, 1C), 23.93 (d, J = 4.8 Hz, 1C), 16.34 (d, J = 3.0 Hz, 1C), 16.29 (d, J = 2.8 Hz, 1C), 14.09.; HRMS (ESI–TOF) m/z: $[M+H]^+$ Calcd for $C_{18}H_{29}O_6P$ 373.1780; Found 373.1784.

5-(benzyloxy)-2-(diethoxyphosphoryl)pentanoic acid. In a flame-dried 25 mL round-bottom flask equipped with a magnetic stir bar, compound **10** (1 mmol) was dissolved in 5 mL of ethanol. Potassium hydroxide (1.5 mmol) was dissolved in 2 mL deionized water and then added to the reaction mixture, after which it was stirred at room temperature for 24 h. Upon completion of the reaction, 50 mL of deionized water was added. The organic layer was extracted with ethyl acetate (3 × 50 mL), and the combined organic extracts were washed successively with 50 mL of water and 50 mL of saturated brine. The organic layer was dried and purified by flash chromatography on silica gel, using gradient elution from 100% ethyl acetate to 10% methanol in EtOAc. Compound **11** was obtained as a pale-yellow oil in 70% yield. ^1H NMR (500 MHz, $CDCl_3$) δ 7.37 – 7.20 (m, 5H), 4.89 (s, 1H), 4.54 (s, 3H), 4.44 (s, 1H), 4.14 – 4.05 (m, 2H), 4.05 – 3.96 (m, 1H), 3.44 (qd, J = 10.5, 7.2, 5.2 Hz, 2H), 2.77 (ddd, J = 21.1, 10.6, 3.6 Hz, 1H), 1.92 (dt, J = 20.6, 12.1 Hz, 1H), 1.74 (pd, J = 10.7, 7.0, 5.8 Hz, 2H), 1.59 (dp, J = 13.1, 6.2 Hz, 1H), 1.24 (t, J = 7.3 Hz, 5H); $^{13}C\{^1H\}$ NMR (126 MHz, $CDCl_3$) δ 173.36 (d, J = 3.3 Hz, 1C), 138.62, 128.24, 127.60, 127.36, 72.69, 69.88, 62.29 (d, J = 6.6 Hz, 1C), 61.94 (d, J = 6.4 Hz, 1C), 48.04 (d, J = 125.9 Hz, 1C), 28.62 (d, J = 15.9 Hz, 1C), 24.56 (d, J = 9.9 Hz, 1C), 16.36 (d, J = 6.8 Hz, 1C), 16.27(d, J = 6.8 Hz, 1C); HRMS (ESI–TOF) m/z: $[M+H]^+$ Calcd for $C_{16}H_{15}O_6P$ 345.1467; Found 345.1466.

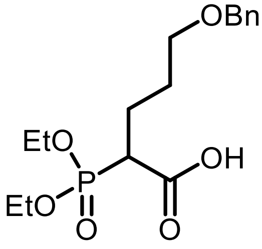

11

***tert*-butyl-2-(5-(benzyloxy)-2-(diethoxyphosphoryl)pentanoyl)hydrazine-1-carboxylate.** In a flame-dried 100 mL round-bottom flask equipped with a magnetic stir bar, compound **11** (1 mmol) was dissolved in 15 mL of anhydrous dichloromethane (DCM) at 0 °C. *Tert*-butyl hydrazinecarboxylate (1.5 mmol), 1-ethyl-3-(3-dimethylaminopropyl)carbodiimide (1.5 mmol), and 4-dimethylaminopyridine (3 mmol) were then added sequentially. The reaction mixture was stirred at room temperature for 36 h. Upon completion, 50 mL of deionized water was added to quench the reaction. The organic layer was extracted with ethyl acetate (3 × 50 mL), and the combined extracts were washed successively with 2 M aq. HCl (50 mL), water (50 mL), and saturated brine (50 mL). The organic phase was dried and concentrated under reduced pressure, and the crude product was purified by flash chromatography on silica gel using a gradient elution from 100% hexanes to 20% EtOAc in hexanes. Compound **12** was obtained as a pale-yellow oil in 72% yield. ^1H NMR (500 MHz, $CDCl_3$) δ 9.21 (s, 1H), 7.38 – 7.26 (m, 5H), 5.22 (d, J = 2.9 Hz, 1H), 4.42 (d, J = 3.0 Hz, 2H), 4.17 – 3.99 (m, 4H), 3.51 – 3.42 (m, 1H), 3.41

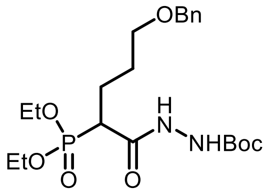

12

(t, *J* = 6.3 Hz, 1H), 3.03 – 2.89 (m, 1H), 2.70 (d, *J* = 11.7 Hz, 1H), 2.11 – 1.87 (m, 1H), 1.75 (dp, *J* = 15.2, 5.4 Hz, 1H), 1.69 – 1.57 (m, 1H), 1.50 – 1.36 (m, 10H), 1.24 (m, 5H).; $^{13}C\{^1H\}$ NMR (126 MHz, $CDCl_3$) δ 167.81, 155.28, 138.39, 128.24, 127.56, 127.43, 80.85, 72.61, 69.39, 63.37 (d, *J* = 6.2 Hz, 1C), 62.37 (d, *J* = 6.6 Hz, 1C), 43.95 (d, *J* = 133.4 Hz, 1C), 28.14, 27.70 (d, *J* = 14.7 Hz, 1C), 24.07 (d, *J* = 5.2 Hz, 1C), 16.33(d, *J* = 6.3 Hz, 1C), 16.27 (d, *J* = 6.0 Hz, 1C); HRMS (ESI–TOF) m/z: $[M+H]^+$ Calcd for $C_{21}H_{35}N_2O_7P$ 458.2182; Found 458.2184.

***tert*-butyl 2-(2-(diethoxyphosphoryl)-5-hydroxypentanoyl)hydrazine-1-carboxylate.** In a flame-dried 25 mL round-bottom flask equipped with a magnetic stirrer, compound **13** (1 mmol) was dissolved in 5 mL of non-distilled methanol. Pearlman's catalyst (0.2 mmol) was then added, and the flask was sealed and secured with parafilm to ensure airtightness. The reaction vessel was evacuated under vacuum, and a hydrogen balloon was introduced through the septum. The mixture was stirred at room temperature for 2 h. Upon completion, the mixture was filtered through a celite pad and washed with 5 mL of MeOH. The filtrate was dried, yielding compound **13** as a white solid in 95% yield. ^1H NMR (500 MHz, $CDCl_3$) δ 9.37 (s, 1H), 5.27 (d, *J* = 2.8 Hz, 2H), 4.15 (dd, *J* = 11.8, 5.7 Hz, 2H), 3.59 (ddt, *J* = 20.6, 13.8, 7.1 Hz, 3H), 3.16 – 3.06 (m, 1H), 2.85 (s, 1H), 2.09 – 2.01 (m, 2H), 2.03 – 1.92 (m, 1H), 1.89 – 1.80 (m, 2H), 1.71 – 1.55 (m, 4H), 1.28 (qd, *J* = 7.7, 7.1, 3.6 Hz, 10H); $^{13}C\{^1H\}$ NMR (126 MHz, $CDCl_3$) δ 168.40, 155.76, 81.37, 63.43 (d, *J* = 6.4 Hz, 1C), 62.88 (d, *J* = 6.7 Hz, 1C), 61.41, 43.66 (d, *J* = 133.5 Hz, 1C), 30.17 (d, *J* = 14.3 Hz, 1C), 28.14, 23.73, 16.32, 16.29; HRMS (ESI–TOF) m/z: $[M+H]^+$ Calcd for $C_{14}H_{29}N_2O_7P$ 369.1791; Found 369.1794

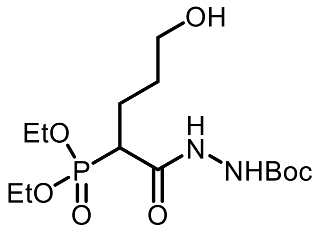

13

***tert*-butyl 2-(5-bromo-2-(diethoxyphosphoryl)pentanoyl)hydrazine-1-carboxylate.** In a flame-dried 500 mL round-bottom flask equipped with a magnetic stir bar, compound **13** (1 mmol) was dissolved in 5 mL of anhydrous DCM at 0 °C. Carbon tetrabromide (1.2 mmol) and triphenylphosphine (1.2 mmol) were then added sequentially. The reaction mixture was stirred at room temperature for 3 h. Upon completion, hexanes was added to precipitate the excess PPh_3 which was then filtered through a celite pad. The filtrate was dried and concentrated under reduced pressure, and the crude product was purified by flash chromatography on silica gel using a gradient elution from 100% hexanes to 20% EtOAc in hexanes. Compound **14** was obtained as a pale-yellow oil in 76% yield. ^1H NMR (500 MHz, $CDCl_3$) δ 9.43 (s, 1H), 4.23 (s, 1H), 4.20 – 4.10 (m, 2H), 4.11 (dd, *J* = 7.3, 3.1 Hz, 1H), 3.46 (s, 1H), 3.47 – 3.32 (m, 2H), 3.04 – 2.94 (m, 1H), 2.02 (s, 4H), 2.00 (t, *J* = 3.8 Hz, 1H), 1.91 – 1.81 (m, 1H), 1.41 (m, 8H), 1.31 (m, 5H); $^{13}C\{^1H\}$ NMR (126 MHz, $CDCl_3$) δ 167.38, 155.24, 81.03, 63.55 (d, *J* = 6.3 Hz, 1C), 62.55 (d, *J* = 6.6 Hz, 1C), 53.47, 43.10 (d, *J* = 133.0 Hz, 1C), 33.10, 30.40 (d, *J* = 14.8 Hz, 1C), 28.17, 25.49 (d, *J* = 5.0 Hz, 1C), 16.38, 16.33; HRMS (ESI–TOF) m/z: $[M+H]^+$ Calcd for $C_{14}H_{28}BrN_2O_6P$ 431.0947; Found 431.0947.

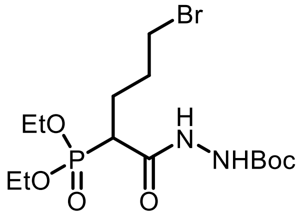

14

***tert*-butyl (3-(diethoxyphosphoryl)-2-oxopiperidin-1-yl)carbamate.** In a flame-dried 50 mL round-bottom flask equipped with a magnetic stir bar, compound **14** (1 mmol) was dissolved in 10 mL of anhydrous MeCN. Potassium carbonate (3 mmol) was then added, and the reaction mixture was stirred at 90 °C for 6 h. Upon completion of the reaction, 50 mL of deionized water was added. The organic layer was extracted with ethyl acetate (3 × 50 mL), and the combined organic extracts were washed successively with 50 mL of water and 50 mL of saturated brine. The organic layer was dried and purified by flash chromatography on silica gel, using gradient elution from 100% hexanes to 20% EtOAc in hexanes. Compound **15** was obtained as off-white oil in 95% yield. ^{1}H NMR (500 MHz, $CDCl_3$) δ 6.97 (s, 1H), 4.25 – 4.04 (m, 4H), 3.56 (m, 2H), 3.04 (tt, *J* = 6.4, 6.6 Hz 1H), 2.19 – 2.09 (m, 1H), 1.83 (m, 1H), 1.42 (s, 9H), 1.29 (t, *J* = 7.0 Hz, 6H); ^{13}C{^{1}H} NMR (126 MHz, $CDCl_3$) δ 165.12 (d, *J* = 4.8 Hz, 1C), 155.19, 81.56, 63.14 (d, *J* = 6.6 Hz, 1C), 62.11 (d, *J* = 6.8 Hz, 1C), 51.87, 41.96 (d, *J* = 137.4 Hz), 28.08, 22.85 (d, *J* = 4.4 Hz, 1C), 21.75 (d, *J* = 8.1 Hz, 1C), 16.39 (d, *J* = 6.1 Hz, 1C), 16.29 (d, *J* = 6.2 Hz, 1C); HRMS (ESI–TOF) m/z: $[M+H]^+$ Calcd for $C_{14}H_{27}N_2O_6P$ 351.1685; Found 351.1684.

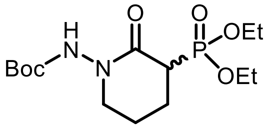

15

diethyl (1-amino-2-oxopiperidin-3-yl)phosphonate. In a flame-dried 25 mL round-bottom flask equipped with a magnetic stir bar, compound **15** (1 mmol) was dissolved in 10 mL of 20% trifluoroacetic acid in DCM. The reaction mixture was stirred at room temperature for 1 h. Upon completion of the reaction, 50 mL of deionized water was added. The organic layer was extracted with ethyl acetate (3 × 50 mL), and the combined organic extracts were washed successively with 50 mL of water and 50 mL of saturated brine. The organic layer was dried and purified by flash chromatography on silica gel, using gradient elution from 100% DCM to 10% MeOH in DCM. Compound **16** was obtained as off-white oil in 96% yield. ^{1}H NMR (500 MHz, $CDCl_3$) δ 8.14 (s, H), 8.10 (s, 0H), 4.32 – 4.11 (m, 4H), 3.65 (m, 2H), 3.28 – 3.12 (m, 1H), 2.25 – 2.10 (m, 3H), 1.90 (m, 1H), 1.36 (t, *J* = 7.1 Hz, 6H); ^{13}C{^{1}H} NMR (126 MHz, $CDCl_3$) δ 164.67 (d, *J* = 2.8 Hz, 1C), 64.29 (d, *J* = 6.8 Hz, 1C), 64.13 (d, *J* = 6.8 Hz, 1C), 51.13, 42.17 (d, *J* = 142.1 Hz, 1C), 22.57 (d, *J* = 4.7 Hz, 1C), 21.75 (d, *J* = 10.5 Hz, 1C), 16.22 (d, *J* = 6.8 Hz, 1C), 16.14 (d, *J* = 6.7 Hz, 1C); HRMS (ESI–TOF) m/z: $[M+H]^+$ Calcd for $C_9H_{19}N_2O_4P$ 251.1161; Found 251.1163.

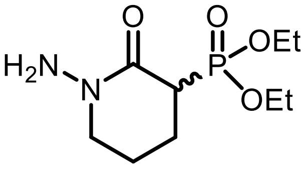

16

(1-amino-2-oxopiperidin-3-yl)phosphonic acid. In a flame-dried 25 mL round-bottom flask equipped with a magnetic stir bar, compound **16** (1 mmol) was dissolved in 5 mL of DCM at 0 °C. Bromotrimethylsilane (3 mmol) was added dropwise to the solution, and the reaction mixture was stirred at room temperature for 4 h. Upon completion of the reaction, 5 mL of methanol was added, and the mixture was evaporated under reduced pressure. This methanol addition and evaporation process was repeated twice more. The resulting crude material was purified by reverse-phase chromatography on C-18 silica gel using a gradient elution

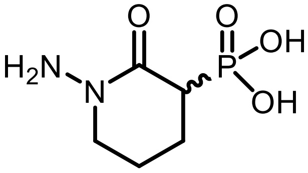

1

system, starting with 100% water and transitioning to 75% water in methanol. Finally, the water/methanol eluent was evaporated under reduced pressure using a rotovap, yielding the target compound **1** as an off-white solid in 92% yield; Mp: 206-207 ˚C; IR (neat): 3597 (w) 3328 (w), 3094 (m), 2983 (m), 1718 (m), 1456 (m), 1443 (m), 1421 (m), 1416 (m), 1392 (s), 1382 (s), 1261 (s), 1254 (m), 1186 (s), 1162 (m), 1088 (s), 1041 (m), 1016 (w), 935 (w), 908 (w), 877 (w), 865 (m), 776 (w), 748 (m), 686 (w), 625 (w), 617 (w), 577 (m) cm^{-1}; ^{1}H NMR (500 MHz, D_2O) δ 3.62 (m, 4H), 3.06 (t, J = 6.6 Hz, 1H), 3.00 (t, J = 6.6 Hz, 1H), 2.16 – 2.05 (m, 1H), 2.03 – 1.97 (m, 1H), 1.87 (m, 1H); $^{13}C\{^{1}H\}$ NMR (126 MHz, D_2O) δ 167.32 (d, J = 5.1 Hz), 49.69, 42.30 (d, J = 128.3 Hz), 22.12 (d, J = 4.0 Hz), 20.50 (d, J = 7.7 Hz); HRMS (ESI–TOF) m/z: $[M+H]^+$ Calcd for $C_5H_{15}N_2O_4P$ 195.0535; Found 195.0534.

1.3 Synthesis of compound 2

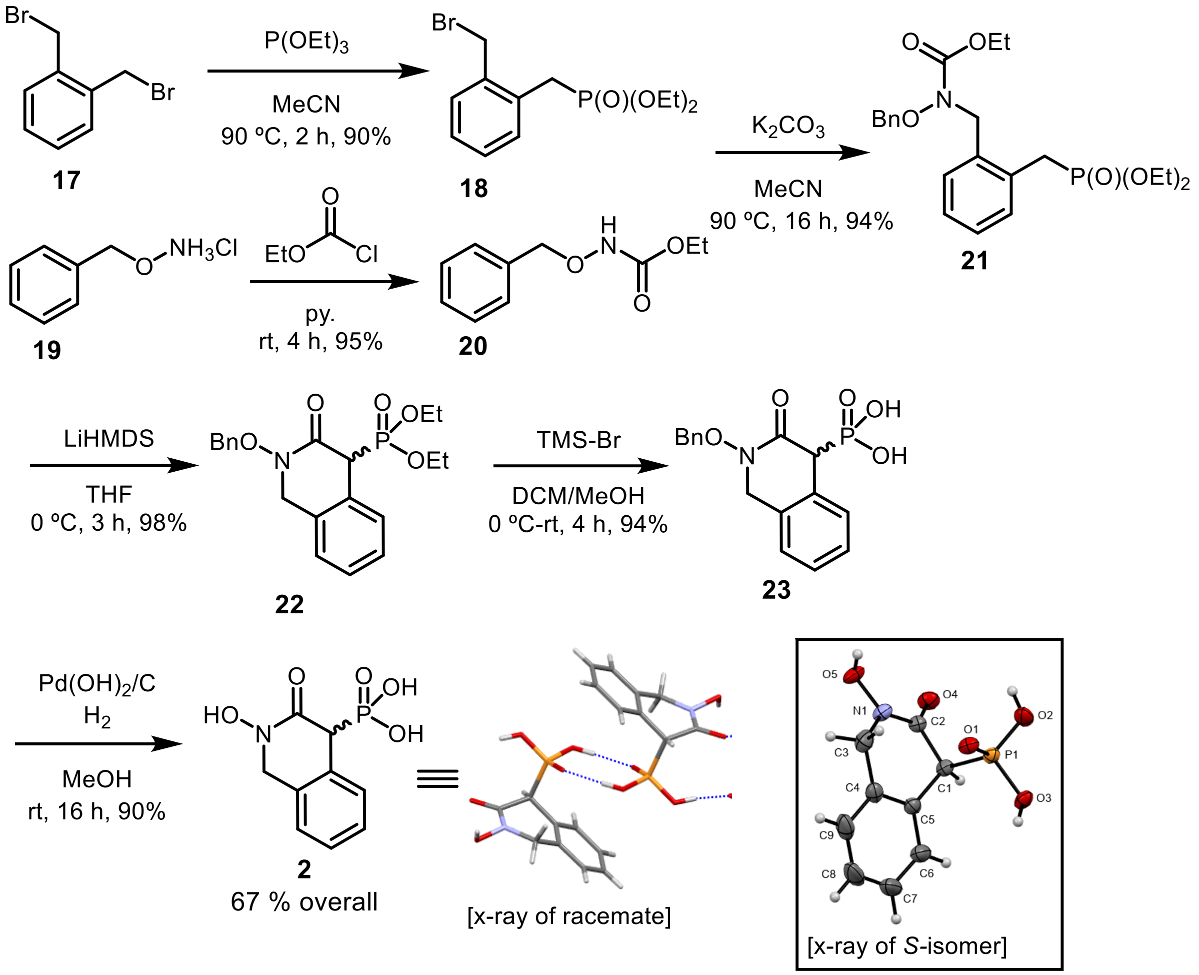

Scheme S2. Synthesis of **2**

diethyl (2-(bromomethyl)benzyl)phosphonate. In a flame-dried 50 mL round-bottom flask equipped with a magnetic stir bar, 1,2-bis(bromomethyl)benzene (10 mmol) and triethyl phosphite (10.2 mmol) were dissolved in 10 mL anhydrous MeCN. The reaction mixture was stirred at 90 °C for 1.6 h. Upon completion of the reaction, the solvent was evaporated and the crude mixture was purified by flash chromatography on silica gel, using gradient elution from 100% DCM to 3% MeOH in DCM to afford compound **18** as pale-yellow oil in 90 % yield. ^{1}H NMR (500 MHz, $CDCl_3$) δ 7.38 – 7.17 (m, 4H), 4.69 (d, *J* = 2.4 Hz, 2H), 4.08 – 3.91 (m, 3H), 3.34 (dd, *J* = 21.7, 2.5 Hz, 2H), 1.23 (td, *J* = 7.1, 2.2 Hz, 6H); $^{13}C\{^{1}H\}$ NMR (126 MHz, $CDCl_3$) δ 136.58 (d, *J* = 6.4 Hz, 1C), 131.72 (d, *J* = 5.6 Hz, 1C), 130.98 (d, *J* = 9.4 Hz, 1C), 130.75 (d, *J* = 3.3 Hz, 1C), 128.99 (d, *J* = 3.6 Hz, 1C), 127.66 (d, *J* = 3.7 Hz, 1C), 62.31, 62.26, 32.37, 30.79 (d, *J* = 137.8 Hz, 1C), 16.37, 16.33; HRMS (ESI–TOF) m/z: $[M+H]^+$ Calcd for $C_{12}H_{18}BrO_3P$ 321.0255; Found 321.0255.

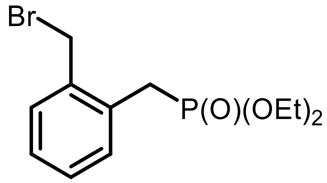

18

ethyl (benzyloxy)carbamate. In a flame-dried 50 mL round-bottom flask equipped with a magnetic stir bar, *O*-benzylhydroxylamine hydrochloride (5 mmol) was added to anhydrous pyridine (10 mL) and stirred at room temperature for 2 h under a nitrogen atmosphere. The reaction mixture was then cooled to 0 °C, and ethyl carbonochloridate (5 mmol) was added. Stirring was continued at room temperature for an additional 2 h. The resulting mixture was diluted with ethyl acetate (50 mL) and washed sequentially with 2 M aq. HCl (2 × 50 mL) and saturated aqueous sodium bicarbonate (2 × 50 mL). The organic layer was dried over anhydrous sodium sulfate, filtered, and concentrated under reduced pressure to afford ethyl benzyloxycarbamate (**20**) as a yellow oil in 95% yield. ^{1}H NMR (500 MHz, $CDCl_3$) δ 7.43 – 7.27 (m, 5H), 4.90 – 4.81 (m, 2H), 4.17 (q, 6.9 Hz, 2H), 1.24 (td, *J* = 8.0, 7.0 Hz, 3H); $^{13}C\{^{1}H\}$ NMR (126 MHz, $CDCl_3$) δ 157.78, 135.66, 129.12, 128.48, 128.44, 78.50, 61.80, 14.43; HRMS (ESI–TOF) m/z: $[M+H]^+$ Calcd for $C_{10}H_{13}NO_3$ 196.0974; Found 196.0976

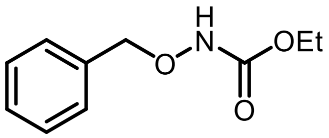

20

ethyl (benzyloxy)(2-((diethoxyphosphoryl)methyl)benzyl)carbamate. In a flame-dried 100 mL round-bottom flask equipped with a magnetic stir bar, compound **18** (3.3 mmol) and compound **20** (3 mmol) were dissolved in 15 mL anhydrous MeCN. Potassium carbonate (15 mmol) was then added, and the reaction mixture was stirred at 90 °C for 16 h. Upon completion of the reaction, 50 mL of deionized water was added. The organic layer was extracted with ethyl acetate (3 × 50 mL), and the combined organic extracts were washed successively with 50 mL of water and 50 mL of saturated brine. The organic layer was dried over anhydrous sodium sulfate, filtered, dried, and purified by flash chromatography on silica gel, using gradient elution from 100% DCM to 4% MeOH in DCM. Compound **21** was obtained as pale-yellow oil in 94% yield. ^{1}H NMR (500 MHz, $CDCl_3$) δ 7.42 – 7.19 (m, 9H), 4.81 (s, 2H), 4.58 (s, 2H), 4.31 – 4.22 (m, 2H), 4.00 (m,

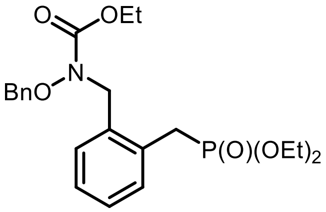

21

4H), 3.32 (s, 1H), 3.28 (s, 1H), 1.37 – 1.34 (m, 3H), 1.29 – 1.21 (m, 6H); $^{13}C\{^{1}H\}$ NMR (126 MHz, $CDCl_3$) δ 157.39, 135.10 (d, *J* = 6.6 Hz, 1C), 134.97, 131.26 (d, *J* = 5.6 Hz, 1C), 130.84 (d, *J* = 3.3 Hz, 1C), 129.47, 129.12, 128.56, 128.36, 128.08 (d, *J* = 3.5 Hz, 1C), 127.21 (d, *J* = 3.8 Hz, 1C), 77.62, 62.39, 62.18, 62.12, 51.55, 30.45 (d, *J* = 137.6 Hz, 1C), 16.38, 16.34, 14.53; HRMS (ESI–TOF) m/z: $[M+H]^+$ Calcd for $C_{22}H_{30}NO_6P$ 436.1889; Found 436.1886.

diethyl (2-(benzyloxy)-3-oxo-1,2,3,4-tetrahydroisoquinolin-4-yl)phosphonate. In a flame-dried 100 mL round-bottom flask equipped with a magnetic stir bar, compound **21** (3 mmol) was dissolved in THF (10 mL) and cooled to 0 °C. A solution of LiHMDS (1.5 M in THF, 6 mL, 9 mmol) was slowly added under a nitrogen atmosphere, and the mixture was stirred at 0°C for 3 h. The reaction was quenched at 0 °C by adding 10% aqueous AcOH (10 mL), followed by deionized water (50 mL). The organic layer was extracted with ethyl acetate (3 × 50 mL), and the combined organic extracts were washed sequentially with water (50 mL) and saturated brine (50 mL). The organic layer was dried over anhydrous sodium sulfate, filtered, and concentrated. The crude product was purified by flash chromatography on silica gel using a gradient elution from 100% DCM to 4% MeOH in DCM. Compound **22** was isolated as a pale-yellow oil in 98% yield. ^1H NMR (500 MHz, $CDCl_3$) δ 7.55 – 7.01 (m, 9H), 5.09 – 4.98 (S, 2H), 4.35 – 4.22 (m, 1H), 4.15 – 4.11 (m, 5H), 4.01 (m, 1H), 1.27 (m, 6H); $^{13}C\{^{1}H\}$ NMR (126 MHz, $CDCl_3$) δ 160.78 (d, *J* = 5.4 Hz, 1C), 135.09, 131.66 (d, *J* = 5.7 Hz, 1C), 129.72, 128.85, 128.66 (d, *J* = 4.8 Hz, 1C), 128.52, 127.66 (d, *J* = 4.4 Hz, 1C), 127.60 (d, *J* = 3.5 Hz, 1C), 125.52 (d, *J* = 3.7 Hz, 1C), 63.64 (d, *J* = 7.6 Hz, 1C), 63.48 (d, *J* = 6.7 Hz, 1C), 53.68, 50.06 (d, *J* = 126.4 Hz, 1C), 16.28 (d, *J* = 4.9 Hz, 1C) 16.26 (d, *J* = 5.1 Hz, 1C); HRMS (ESI–TOF) m/z: $[M+H]^+$ Calcd for $C_{20}H_{24}NO_5P$ 390.1470; Found 390.1472.

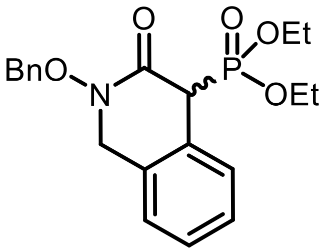

22

(2-(benzyloxy)-3-oxo-1,2,3,4-tetrahydroisoquinolin-4-yl)phosphonic acid. In a flame-dried 25 mL round-bottom flask equipped with a magnetic stir bar, compound **22** (1 mmol) was dissolved in 5 mL of DCM at 0 °C. Bromotrimethylsilane (3 mmol) was added dropwise to the solution, and the reaction mixture was stirred at room temperature for 4 h. Upon completion of the reaction, 5 mL of methanol was added, and the mixture was evaporated under reduced pressure. This methanol addition and evaporation process was repeated twice more. The resulting crude material was purified by reverse-phase chromatography on C-18 silica gel using a gradient elution system, starting with 100% water and transitioning to 80% water in methanol. Finally, the water/methanol eluent was evaporated under reduced pressure using a rotovap, yielding the target compound **23** as an off-white solid in 94% yield. ^1H NMR (500 MHz, CD_3OD) δ 7.53 – 7.45 (m, 2H), 7.36 (dd, *J* = 3.1, 1.7 Hz, 2H), 7.34 – 7.16 (m, 5H), 5.07 – 5.00 (m, 3H), 4.59 – 4.52 (m, 1H), 4.25 (d, *J* = 27.2 Hz, 1H);

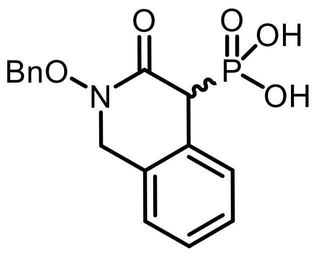

23

$^{13}C\{^1H\}$ NMR (126 MHz, CD_3OD) δ 162.76 (d, *J* = 5.2 Hz, 1C), 134.84, 131.26 (d, *J* = 5.1 Hz, 1C), 129.62, 128.75, 128.61 (d, *J* = 4.3 Hz, 1C), 128.33, 127.52 (d, *J* = 3.7 Hz, 1C), 127.35 (d, *J* = 3.6 Hz, 1C), 125.57 (d, *J* = 3.6 Hz, 1C), 76.00, 52.81, 46.62; HRMS (ESI–TOF) m/z: $[M+H]^+$ Calcd for $C_{16}H_{16}NO_5P$ 334.0844; Found 334.0846.

(2-hydroxy-3-oxo-1,2,3,4-tetrahydroisoquinolin-4-yl)phosphonic acid. In a flame-dried 25 mL round-bottom flask equipped with a magnetic stir bar, compound **23** (1 mmol) was dissolved in 5 mL of non-distilled methanol. Pearlman's catalyst (0.2 mmol) was then added, and the flask was sealed and secured with parafilm to ensure airtightness. The reaction vessel was evacuated under vacuum, and a hydrogen balloon was introduced through the septum. The mixture was stirred at room temperature for 2 h. Upon completion, the mixture was filtered through a celite pad and washed with 5 mL of MeOH. The filtrate was dried, yielding compound **2** as a white solid in 90% yield; Mp: 195-196 ˚C; IR (neat): 3591 (w) 3261 (w), 3097 (m), 2942 (m), 1716 (m), 1598 (m), 1583 (m), 1494 (m), 1476 (m), 1464 (m), 1453 (m), 1363 (s), 1343 (s), 1228 (s), 1214 (m), 1196 (s), 1178 (m), 1065 (s), 1042 (m), 1021 (w), 986 (w), 968 (w), 856 (w), 841 (m), 785 (w), 763 (m), 686 (w), 624 (w), 616 (w), 542 (m) cm^{-1}; ^1H NMR (500 MHz, DMSO-d_6) δ 7.31 – 7.19 (m, 4H), 5.02 (dd, *J* = 14.8, 8.8 Hz, 1H), 4.48 (dd, *J* = 14.8, 3.1 Hz, 1H), 3.97 (d, *J* = 26.3 Hz, 1H); $^{13}C\{^1H\}$ NMR (126 MHz, DMSO-d_6) δ 161.12 (d, *J* = 5.2 Hz, 1C), 132.49 (d, *J* = 4.8 Hz, 1C), 130.90 (d, *J* = 8.2 Hz, 1C), 129.22 (d, *J* = 4.1 Hz, 1C), 127.41, 126.95, 125.78, 54.42, 51.52 (d, *J* = 120.1 Hz, 1C); HRMS (ESI–TOF) m/z: $[M+H]^+$ Calcd for $C_9H_{10}NO_5P$ 244.0375; Found 244.0372.

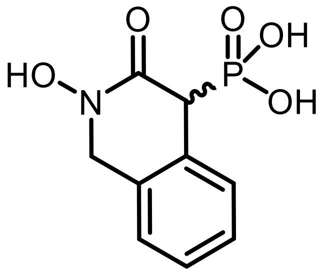

2

1.4 Synthesis of compound 3

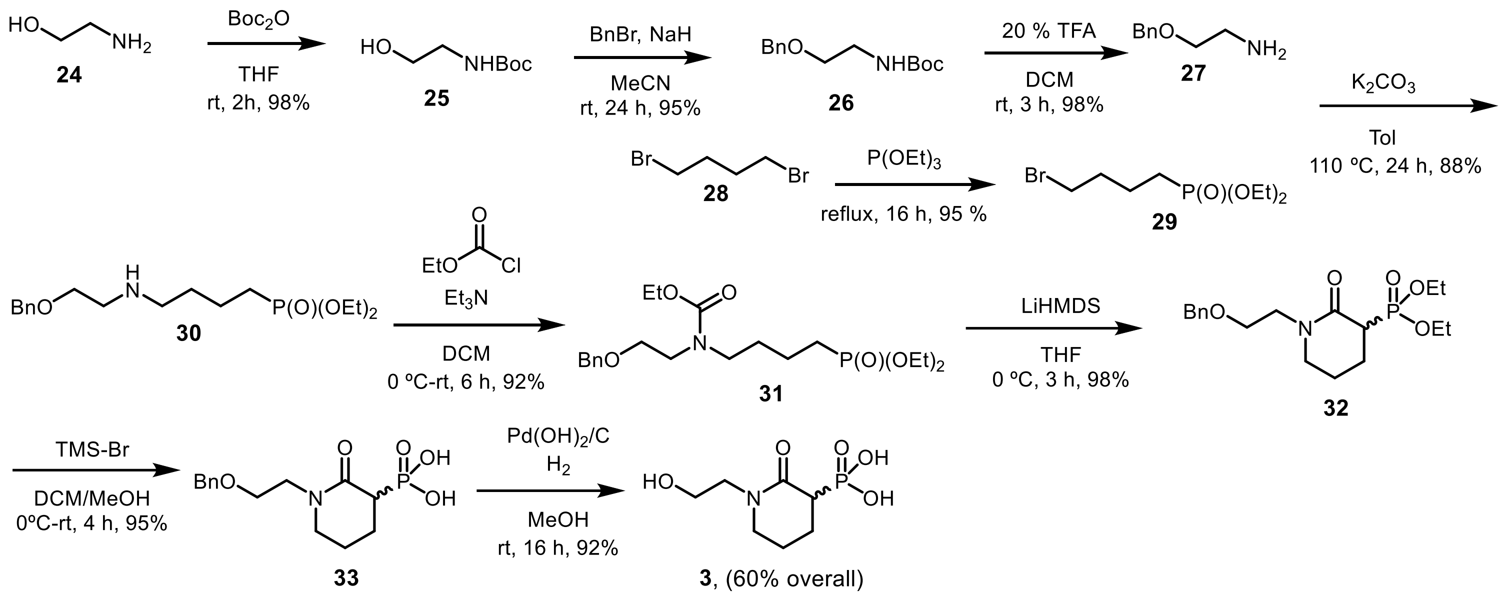

Scheme S3. Synthesis of **3**

***tert*-butyl (2-hydroxyethyl)carbamate.** In a flame-dried 25 mL round-bottom flask equipped with a magnetic stir bar, 2-aminoethan-1-ol (**24**, 1.5 mmol) and di-*tert*-butyl dicarbonate (1 mmol) were added and the reaction stirred at room temperature for 12 h. Upon completion, 50 mL of deionized water was added and the organic layer was extracted with ethyl acetate (3 × 50 mL). The combined organic extracts were washed sequentially with water (50 mL) and saturated brine (50 mL). The organic layer was dried over anhydrous sodium sulfate, filtered, and concentrated to afford compound **25** as a pale-yellow oil in 98% yield. ^{1}H NMR (500 MHz, DMSO-d_6) δ 6.60 (t, J = 5.8 Hz, 1H), 4.55 (t, J = 5.6 Hz, 1H), 3.37 (q, J = 6.1 Hz, 2H), 2.99 (q, J = 6.1 Hz, 2H), 1.37 (s, 9H); ^{13}C{^{1}H} NMR (126 MHz, DMSO-d_6) δ 156.08, 77.88, 60.54, 43.10, 28.62; HRMS (ESI–TOF) m/z: [M+H]$^+$ Calcd for $C_7H_{16}NO_3$ 162.1130; Found 162.1134.

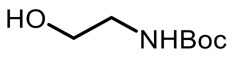

25

***tert*-butyl (2-(benzyloxy)ethyl)carbamate.** In a flame-dried 25 mL round-bottom flask equipped with a magnetic stir bar, compound **25** (3 mmol) was dissolved in 5 mL MeCN at 0 ˚C after which sodium hydride (4.5 mmol) was added. Benzyl bromide (4.5 mmol) was added dropwise to the stirring mixture and then allowed to warm to room temperature and stirred for 16 h. Upon completion, 50 mL of deionized water was added and the organic layer was extracted with ethyl acetate (3 × 50 mL). The combined organic extracts were washed sequentially with water (50 mL) and saturated brine (50 mL). The organic layer was dried over anhydrous sodium sulfate, filtered, and concentrated. The crude product was purified by flash chromatography on silica gel, using

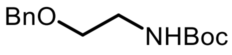

26

gradient elution from 100% hexanes to 20% EtOAc in hexanes to afford compound **26** as a pale-yellow oil in 95% yield. ^{1}H NMR (500 MHz, $CDCl_3$) δ 7.37 – 7.29 (m, 5H), 5.10 (t, *J* = 6.0 Hz, 1H), 4.51 (s, 2H), 3.53 (t, *J* = 5.2 Hz, 2H), 3.35 (q, *J* = 5.5 Hz, 2H), 1.47 (s, 9H); ^{13}C{^{1}H} NMR (126 MHz, $CDCl_3$) δ 155.99, 138.05, 128.42, 127.72, 127.70, 79.11, 73.03, 69.29, 40.47, 28.44; HRMS (ESI–TOF) m/z: $[M+H]^+$ Calcd for $C_{14}H_{22}NO_3$ 252.1600; Found 252.1602.

2-(benzyloxy)ethan-1-amine. In a flame-dried 25 mL round-bottom flask equipped with a magnetic stir bar, compound **26** (1 mmol) was dissolved in 10 mL of 20% trifluoroacetic acid in DCM. The reaction mixture was stirred at room temperature for 1 h. Upon completion of the reaction, 50 mL of deionized water was added. The organic layer was extracted with ethyl acetate (3 × 50 mL), and the combined organic extracts were washed successively with 50 mL of water and 50 mL of saturated brine. The organic layer was dried and purified by flash chromatography on silica gel, using gradient elution from 100% DCM to 10% MeOH in DCM. Compound **27** was obtained as a pale-yellow oil in 98% yield. ^{1}H NMR (500 MHz, $CDCl_3$) δ 7.28 (m, 5H), 4.45 (s, 2H), 3.56 (t, *J* = 5.4 Hz, 2H), 3.04 (m, 2H); ^{13}C{^{1}H} NMR (126 MHz, $CDCl_3$) δ 136.92, 128.59, 128.20, 128.02, 73.24, 65.24, 39.75; HRMS (ESI–TOF) m/z: $[M+H]^+$ Calcd for $C_9H_{14}NO$ 152.1075; Found 152.1076.

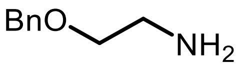

27

diethyl (4-bromobutyl)phosphonate. In a flame-dried 50 mL round-bottom flask equipped with a magnetic stir bar, 1,4-dibromobutane (10 mmol) and triethyl phosphite (3.3 mmol) were stirred at 90 °C for 15 h. Upon completion of the reaction, the crude mixture was concentrated and purified by flash chromatography on silica gel, using gradient elution from 100% DCM to 4% MeOH in DCM to afford compound **29** as a pale-yellow oil in 95 % yield. ^{1}H NMR (500 MHz, $CDCl_3$) δ 4.01 (m, 4H), 3.33 (t, *J* = 6.5 Hz, 2H), 1.88 (m, 2H), 1.79 – 1.55 (m, 4H), 1.33 – 1.18 (m, 6H).; ^{13}C{^{1}H} NMR (126 MHz, $CDCl_3$) δ 61.49, 61.40, 33.13, 32.67 (d, *J* = 1.5 Hz, 1C), 25.61, 21.12 (d, *J* = 5.0 Hz, 1C), 16.43, 16.35; HRMS (ESI–TOF) m/z: $[M+H]^+$ Calcd for $C_8H_{18}BrO_3P$ 273.0255; Found 273.0256.

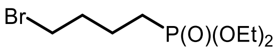

29

diethyl (4-((2-(benzyloxy)ethyl)amino)butyl)phosphonate. In a flame-dried 100 mL round-bottom flask equipped with a magnetic stir bar, compound **29** (3.6 mmol) and compound **27** (10.8 mmol) were dissolved in 15 mL anhydrous toluene. Potassium carbonate (15 mmol) was then added, and the reaction mixture was stirred at 110 °C for 24 h. Upon completion of the reaction, 50 mL of deionized water was added. The organic layer was extracted with ethyl acetate (3 × 50 mL), and the combined organic extracts were washed successively with 50 mL of water and 50 mL of saturated brine. The organic layer was dried over anhydrous sodium sulfate, filtered, dried, and purified by flash chromatography on silica gel, using gradient elution from 100% DCM to 8% MeOH in DCM. Compound **30** was obtained as a pale-yellow oil in 88% yield. ^{1}H NMR (500 MHz, $CDCl_3$) δ 7.31 (m, 5H), 4.49 (s, 2H), 4.05 (q, *J* = 7.9 Hz, 4H), 3.56 (t, *J* = 5.1 Hz, 2H), 3.52 – 3.45 (m, 2H), 2.83

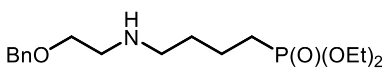

30

(dt, J = 23.7, 5.2 Hz, 1H), 2.78 (t, J = 5.2 Hz, 2H), 2.59 (t, J = 7.0 Hz, 2H), 1.71 (m, 2H), 1.65 – 1.54 (m, 1H), 1.57 – 1.49 (m, 1H), 1.28 (t, J = 7.1 Hz, 6H); $^{13}C\{^1H\}$ NMR (126 MHz, $CDCl_3$) δ 138.23, 128.36, 127.71, 127.66, 73.17, 69.52, 61.41 (d, J = 3.2 Hz), 61.35 (d, J = 3.2 Hz), 49.37, 41.90, 30.95 (d, J = 16.5 Hz), 25.57 (d, J = 140.7 Hz), 20.34 (d, J = 5.0 Hz), 16.49 (d, J = 1.8 Hz), 16.44 (d, J = 1.7 Hz); HRMS (ESI–TOF) m/z: $[M+H]^+$ Calcd for $C_{17}H_{31}NO_4P$ 344.1991; Found 344.1993.

ethyl (2-(benzyloxy)ethyl)(4-(diethoxyphosphoryl)butyl)carbamate. In a flame-dried 50 mL round-bottom flask equipped with a magnetic stir bar, compound **30** (3.5 mmol) was dissolved in 10 mL of anhydrous DCM at 0 °C. To this stirring solution was added triethyl amine (3 mmol) followed by ethyl carbonochloridate (7 mmol) and the reaction was stirred at room temperature for 6 h. Upon completion of the reaction, 50 mL of deionized water was added. The organic layer was extracted with DCM (3 × 50 mL), and the combined organic extracts were washed successively with 50 mL of water and 50 mL of saturated brine. The organic layer was dried and purified by flash chromatography on silica gel, using gradient elution from 100% DCM to 8% MeOH in DCM. Compound **31** was obtained as a pale-yellow oil in 92% yield. ^1H NMR (500 MHz, $CDCl_3$) δ 7.31 (m, 5H), 4.51 (s, 2H), 4.09 (m, 6H), 3.61 (d, J = 5.8 Hz, 1H), 3.59 – 3.55 (m, 1H), 3.48 – 3.38 (m, 2H), 3.30 (t, J = 8.2 Hz, 2H), 1.72 (m, 2H), 1.64 – 1.57 (m, 4H), 1.31 (t, J = 7.1 Hz, 6H), 1.27 – 1.19 (t, J = 7.2 Hz, 3H); $^{13}C\{^1H\}$ NMR (126 MHz, $CDCl_3$) δ 156.32 (d, J = 14.9 Hz), 138.14 (d, J = 11.6 Hz), 128.37, 127.61, 127.53, 73.11, 68.81, 61.47, 61.42, 61.15, 47.45 (d, J = 16.4 Hz), 46.81, 28.95 (d, J = 16.4 Hz), 25.96 (d, J = 10.5 Hz), 24.87, 16.49, 16.44, 14.67; HRMS (FTMS+pAPCI) m/z: $[M+H]^+$ Calcd for $C_{20}H_{35}NO_6P$ 416.2202; Found 416.2204.

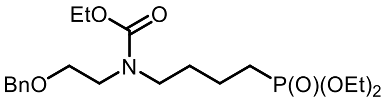

31

diethyl (1-(2-(benzyloxy)ethyl)-2-oxopiperidin-3-yl)phosphonate. In a flame-dried 100 mL round-bottom flask equipped with a magnetic stir bar, compound **31** (3 mmol) was dissolved in THF (10 mL) and cooled to 0 °C. A solution of LiHMDS (1.5 M in THF, 6 mL, 9 mmol) was slowly added under a nitrogen atmosphere, and the mixture was stirred at 0 °C for 3 h. The reaction was quenched at 0 °C by adding 10% aqueous AcOH (10 mL), followed by deionized water (50 mL). The organic layer was extracted with ethyl acetate (3 × 50 mL), and the combined organic extracts were washed sequentially with water (50 mL) and saturated brine (50 mL). The organic layer was dried over anhydrous sodium sulfate, filtered, and concentrated. The crude product was purified by flash chromatography on silica gel using a gradient elution from 100% DCM to 6% MeOH in DCM. Compound **32** was isolated as a pale-yellow oil in 98% yield. ^1H NMR (500 MHz, $CDCl_3$) δ 7.39 – 7.25 (m, 5H), 4.52 (q, J = 7.4 Hz, 2H), 4.30 – 4.17 (m, 2H), 4.21 – 4.07 (m, 2H), 3.76 – 3.63 (m, 3H), 3.57 (m, 1H), 3.48 (m, 2H), 2.99 (tt, J = 6.9 Hz, 1H), 2.25 – 2.12 (m, 1H), 2.16 – 2.02 (m, 2H), 1.74 (m, 1H), 1.34 (m, 6H); $^{13}C\{^1H\}$ NMR (126 MHz, $CDCl_3$) δ 164.99 (d, J

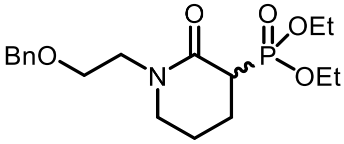

32

= 4.8 Hz), 138.21, 128.39, 127.62, 127.55, 73.17, 68.70, 62.96 (d, *J* = 6.8 Hz), 61.93 (d, *J* = 6.8 Hz), 49.75, 48.12, 41.78 (d, *J* = 138.3 Hz), 23.19 (d, *J* = 4.5 Hz), 21.80 (d, *J* = 8.2 Hz), 16.49 (d, *J* = 6.1 Hz), 16.38 (d, *J* = 6.3 Hz); HRMS (ESI–TOF) m/z: $[M+H]^+$ Calcd for $C_{18}H_{29}NO_5P$ 370.1783; Found 370.1784.

(1-(2-(benzyloxy)ethyl)-2-oxopiperidin-3-yl)phosphonic acid. In a flame-dried 25 mL round-bottom flask equipped with a magnetic stir bar, compound **32** (1 mmol) was dissolved in 5 mL of DCM at 0 °C. Bromotrimethylsilane (3 mmol) was added dropwise to the solution, and the reaction mixture was stirred at room temperature for 4 h. Upon completion of the reaction, 5 mL of methanol was added, and the mixture was evaporated under reduced pressure. This methanol addition and evaporation process was repeated twice more. The resulting crude material was purified by reverse-phase chromatography on C-18 silica gel using a gradient elution system, starting with 100% water and transitioning to 70% water in MeCN. Finally, the water/MeCN eluent was evaporated under reduced pressure using a rotovap, yielding the target compound **33** as an off-white solid in 95% yield. ^{1}H NMR (500 MHz, DMSO-d_6) δ 7.39 – 7.29 (m, 5H), 4.48 (s, 2H), 3.59 – 3.50 (m, 1H), 3.49 – 3.39 (m, 1H), 3.36 (t, *J* = 5.6 Hz, 3H), 2.75 (tt, *J* = 6.6 Hz, 1H), 1.96 (m, 4H), 1.64 (m, 1H); ^{13}C{^{1}H} NMR (126 MHz, DMSO-d_6) δ 166.62 (d, *J* = 4.5 Hz), 138.89, 128.73, 127.86, 127.86, 72.43, 67.88, 49.23, 47.34, 42.38 (d, *J* = 129.6 Hz), 23.05 (dd, *J* = 13.7, 3.7 Hz), 21.55 (dd, *J* = 7.9, 4.7 Hz); HRMS (ESI–TOF) m/z: $[M+H]^+$ Calcd for $C_{14}H_{21}NO_5P$ 314.1157; Found 314.1154.

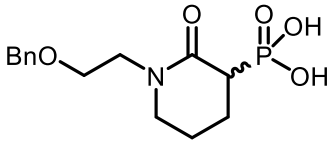

33

(1-(2-hydroxyethyl)-2-oxopiperidin-3-yl)phosphonic acid. In a flame-dried 25 mL round-bottom flask equipped with a magnetic stir bar, compound **33** (1 mmol) was dissolved in 5 mL of non-distilled methanol. Pearlman's catalyst (0.2 mmol) was then added, and the flask was sealed and secured with parafilm to ensure airtightness. The reaction vessel was evacuated under vacuum, and a hydrogen balloon was introduced through the septum. The mixture was stirred at room temperature for 2 h. Upon completion, the mixture was filtered through a celite pad and washed with 5 mL of MeOH. The filtrate was dried, yielding compound **3** as a white solid in 92% yield; Mp: 166-167 ˚C; IR (neat): 3424 (w) 3234 (w), 2962 (m), 1785 (m), 1546 (m), 1524 (m), 1486 (m), 1474 (m), 1453 (m), 1424 (m), 1374 (s), 1331 (s), 1232 (s), 1226 (m), 1182 (s), 1145 (m), 1075 (s), 1026 (m), 1017 (w), 975 (w), 923 (w), 824 (w), 812 (m), 774 (w), 721 (m), 656 (w), 632 (w), 612 (w), 562 (m) cm^{-1}; ^{1}H NMR (500 MHz, DMSO-d_6) δ 3.49 (t, *J* = 6.2 Hz, 2H), 3.40 (m, 1H), 3.35 (d, *J* = 6.1 Hz, 2H), 3.27 (dt, *J* = 12.9, 6.2 Hz, 1H), 2.74 (tt, *J* = 6.8, 6.6 Hz, 1H), 1.94 (m, 3H), 1.64 (m, 1H); ^{13}C{^{1}H} NMR (126 MHz, DMSO-d_6) δ 166.70 (d, *J* = 4.3 Hz), 58.94, 50.23, 49.23, 42.29 (d, *J* = 129.8 Hz), 23.00 (d, *J* = 3.7 Hz), 21.57 (d, *J* = 7.9 Hz); HRMS (ESI–TOF) m/z: $[M+H]^+$ Calcd for $C_7H_{15}NO_5P$ 224.0688; Found 224.0686.

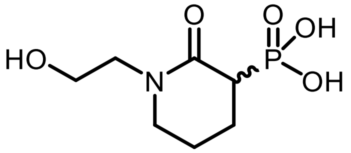

3

1.5 Synthesis of compound 4

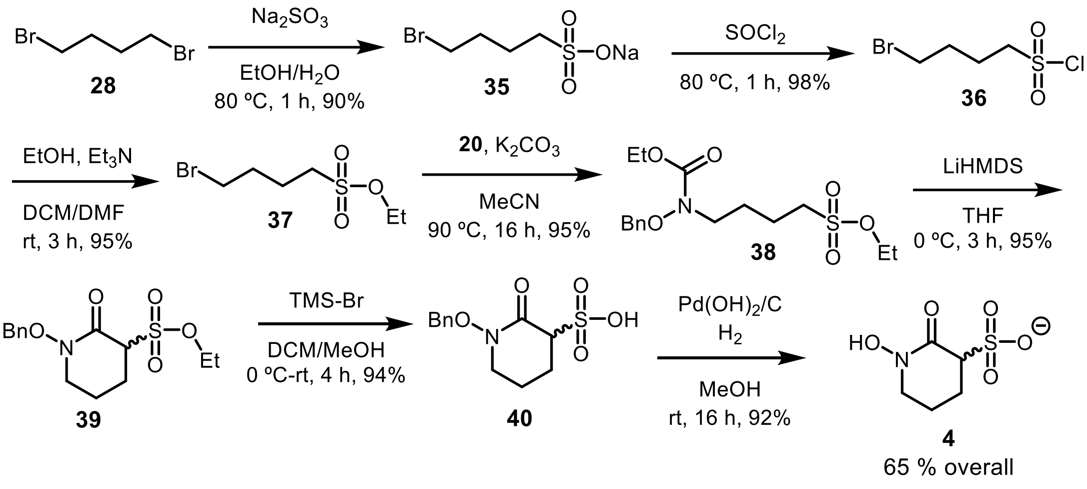

Scheme S4. Synthesis of **4**

sodium 4-bromobutane-1-sulfonate.[1] In a flame-dried 50 mL round-bottom flask equipped with a magnetic stir bar, 1,4-dibromobutane (**28**, 10 mmol) was dissolved in 5 mL of 95% ethanol. A solution of sodium sulfite (3.3 mmol) in 2 mL of deionized water was then added to the mixture, which was stirred at 80 °C for 1 h. After the reaction was complete, the solvent was evaporated, yielding compound **35** as a white solid with a 90% yield. ^{1}H NMR (500 MHz, D$_2$O) δ 3.66 – 3.57 (m, 1H), 3.11 – 2.96 (m, 3H), 2.10 – 2.02 (m, 1H), 2.06 – 1.87 (m, 3H); ^{13}C{^{1}H} NMR (126 MHz, D$_2$O) δ 50.63, 34.39, 30.88, 23.26.

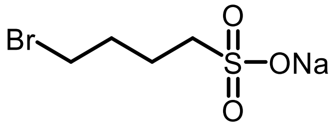

35

ethyl 4-bromobutane-1-sulfonate.[2] In a flame-dried 50 mL round-bottom flask equipped with a magnetic stir bar, compound **35** (10 mmol) was suspended in 10 mL of thionyl chloride. Three drops of DMF were added, and the mixture was stirred at 80 °C for 30 min. Upon completion, the reaction mixture was transferred to a separatory funnel containing ice. The organic layer was extracted with DCM (3 × 50 mL), and the combined extracts were washed successively with water (50 mL) and saturated brine (50 mL). The organic layer was dried over anhydrous sodium sulfate, filtered, and concentrated to afford the 4-bromobutane-1-sulfonyl chloride intermediate (98%), which was used in the next step without further purification. In a separate flame-dried 50 mL round-bottom flask equipped with a magnetic stir bar, the 4-bromobutane-1-sulfonyl chloride intermediate (10 mmol) was dissolved in 10 mL of 95% ethanol, followed by the addition of triethylamine (20 mmol). The reaction mixture was stirred at room temperature for 3 h. Upon completion, the reaction was quenched with 50 mL of deionized

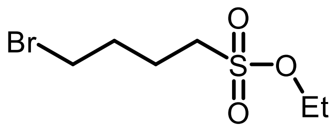

37

water. The organic layer was extracted with ethyl acetate (3 × 50 mL), and the combined extracts were washed with water (50 mL) and saturated brine (50 mL). The organic layer was dried over anhydrous sodium sulfate, filtered, and concentrated. The crude product was purified by flash chromatography on silica gel using a gradient elution from 100% hexanes to 30% EtOAc in hexanes. Compound **37** was obtained as a pale-yellow oil in 95% yield. ^{1}H NMR (500 MHz, $CDCl_3$) δ 4.30 (q, *J* = 7.1 Hz, 2H), 3.54 – 3.42 (m, 2H), 3.13 (m, 2H), 2.05 (m, 2H), 1.42 (t, *J* = 9.4, 7.3 Hz, 3H); ^{13}C{^{1}H} NMR (126 MHz, $CDCl_3$) δ 66.22, 49.42, 32.29, 30.70, 22.29, 15.17.

ethyl 4-((benzyloxy)(ethoxycarbonyl)amino)butane-1-sulfonate. In a flame-dried 100 mL round-bottom flask equipped with a magnetic stir bar, compound **20** (3 mmol) and compound **37** (3.3 mmol) were dissolved in 15 mL anhydrous MeCN. Potassium carbonate (15 mmol) was then added, and the reaction mixture was stirred at 90 °C for 16 h. Upon completion of the reaction, 50 mL of deionized water was added. The organic layer was extracted with ethyl acetate (3 × 50 mL), and the combined organic extracts were washed successively with 50 mL of water and 50 mL of saturated brine. The organic layer was dried over anhydrous sodium sulfate, filtered, dried, and purified by flash chromatography on silica gel, using gradient elution from 100% DCM to 4% MeOH in DCM. Compound **38** was obtained as a pale-yellow oil in 95% yield. ^{1}H NMR (500 MHz, $CDCl_3$) δ 7.46 (d, *J* = 2.9 Hz, 5H), 4.93 (s, 2H), 4.23 (q, *J* = 7.1 Hz, 2H), 4.08 (q, *J* = 7.3 Hz, 2H), 3.56 (m, 1H), 3.40 (m, 1H), 2.13 – 2.00 (m, 2H), 2.02 – 1.93 (m, 1H), 1.86 (m, 1H), 1.65 (m, 2H), 1.31 (m, 6H); ^{13}C{^{1}H} NMR (126 MHz, $CDCl_3$) δ 157.44, 135.35, 129.43, 128.53, 66.03, 62.28, 61.36, 49.89, 48.68, 28.48, 25.53, 20.79, 15.16, 14.57; HRMS (ESI–TOF) m/z: $[M+H]^+$ Calcd for $C_{16}H_{25}NO_6S$ 360.1481; Found 360.1484.

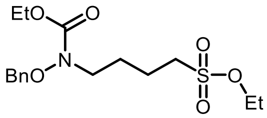

38

ethyl 1-(benzyloxy)-2-oxopiperidine-3-sulfonate. In a flame-dried 100 mL round-bottom flask equipped with a magnetic stir bar, compound **38** (3 mmol) was dissolved in THF (10 mL) and cooled to 0 °C. A solution of LiHMDS (1.5 M in THF, 6 mL, 9 mmol) was slowly added under a nitrogen atmosphere, and the mixture was stirred at 0 °C for 3 h. The reaction was quenched at 0 °C by adding 10% aqueous AcOH (10 mL), followed by deionized water (50 mL). The organic layer was extracted with ethyl acetate (3 × 50 mL), and the combined organic extracts were washed sequentially with water (50 mL) and saturated brine (50 mL). The organic layer was dried over anhydrous sodium sulfate, filtered, and concentrated. The crude product was purified by flash chromatography on silica gel using a gradient elution from 100% DCM to 4% MeOH in DCM. Compound **39** was isolated as a pale-yellow oil in 95% yield. ^{1}H NMR (500 MHz, $CDCl_3$) δ 7.53 – 7.42 (m, 2H), 7.45 – 7.32 (m, 3H), 5.04 – 4.94 (q, *J* = 7.3 Hz, 2H), 4.58 – 4.43 (m, 2H), 4.10 (ddd, *J* = 5.9, 4.8, 0.9 Hz, 1H), 3.43 (ddd, *J* = 11.5, 8.8, 5.1 Hz, 1H), 3.39 – 3.29 (m, 1H), 2.48 – 2.38 (m, 1H), 2.31 – 2.08 (m, 2H), 1.84 – 1.73 (m, 1H), 1.47 (t, *J* = 7.1 Hz, 3H); ^{13}C{^{1}H} NMR (126 MHz, $CDCl_3$) δ 158.97, 134.88, 129.87, 128.95,

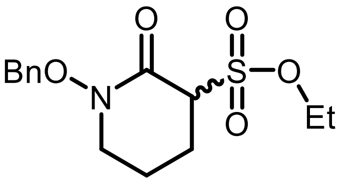

39

128.57, 76.16, 69.60, 63.10, 50.93, 23.53, 20.23, 15.29; HRMS (ESI–TOF) m/z: $[M+H]^+$ Calcd for $C_{14}H_{19}NO_5S$ 314.1062; Found 314.1062.

1-(benzyloxy)-2-oxopiperidine-3-sulfonic acid. In a flame-dried 25 mL round-bottom flask equipped with a magnetic stir bar, compound **39** (1 mmol) was dissolved in 5 mL of DCM at 0 °C. Bromotrimethylsilane (3 mmol) was added dropwise to the solution, and the reaction mixture was stirred at room temperature for 4 h. Upon completion of the reaction, 5 mL of methanol was added, and the mixture was evaporated under reduced pressure. This methanol addition and evaporation process was repeated twice more. The resulting crude material was purified by reverse-phase chromatography on C-18 silica gel using a gradient elution system, starting with 100% water and transitioning to 75% water in methanol. Finally, the water/methanol eluent was evaporated under reduced pressure using a rotovap, yielding the target compound **40** as an off-white solid in 94% yield. ^{1}H NMR (500 MHz, DMSO-d_6) δ 7.43 (m, 5H), 5.05 (s, 2H), 3.44 (dd, J = 8.8, 5.7 Hz, 1H), 3.43 – 3.24 (m, 2H), 2.00 – 1.85 (m, 2H), 1.76 – 1.64 (m, 1H), 1.61 (m, 1H).; ^{13}C{^{1}H} NMR (126 MHz, DMSO-d_6) δ 170.05, 133.72, 129.79, 129.71, 129.14, 75.25, 65.41, 48.65, 26.44, 21.78.; HRMS (ESI–TOF) m/z: $[M+H]^+$ Calcd for $C_{12}H_{15}NO_5S$ 286.0749; Found 286.0746.

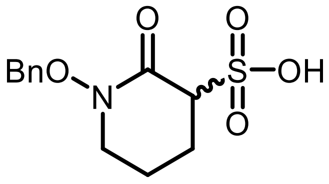

40

1-hydroxy-2-oxopiperidine-3-sulfonic acid. In a flame-dried 25 mL round-bottom flask equipped with a magnetic stirrer, compound **40** (1 mmol) was dissolved in 5 mL of non-distilled methanol. Pearlman's catalyst (0.2 mmol) was then added, and the flask was sealed and secured with parafilm to ensure airtightness. The reaction vessel was evacuated under vacuum, and a hydrogen balloon was introduced through the septum. The mixture was stirred at room temperature for 2 h. Upon completion, the mixture was filtered through a celite pad and washed with 5 mL of MeOH. The filtrate was dried, yielding compound **4** as a white solid in 92% yield; Mp: 154-155 ˚C; IR (neat): 3586 (w) 3275 (w), 3092 (m), 2942 (m), 1702 (m), 1486 (m), 1474 (m), 1466 (m), 1453 (m), 1392 (s), 1362 (s), 1253 (s), 1234 (m), 1186 (s), 1162 (m), 1083 (s), 1042 (m), 1061 (w), 953 (w), 924 (w), 842 (w), 821 (m), 775 (w), 724 (m), 686 (w), 663 (w), 624 (w), 576 (m) cm^{-1}; ^{1}H NMR (500 MHz, D_2O) δ 3.90 – 3.83 (m, 1H), 3.13 – 3.03 (m, 2H), 2.06 – 1.89 (m, 2H), 1.83 – 1.61 (m, 2H); ^{13}C{^{1}H} NMR (126 MHz, D_2O) δ 170.01, 64.88, 50.88, 25.14, 21.04; HRMS (ESI–TOF) m/z: $[M+H]^+$ Calcd for $C_5H_9NO_5S$ 196.0280; Found 196.0284.

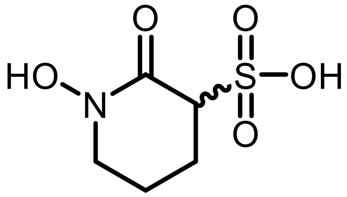

4

1.6 Synthesis of compound 5

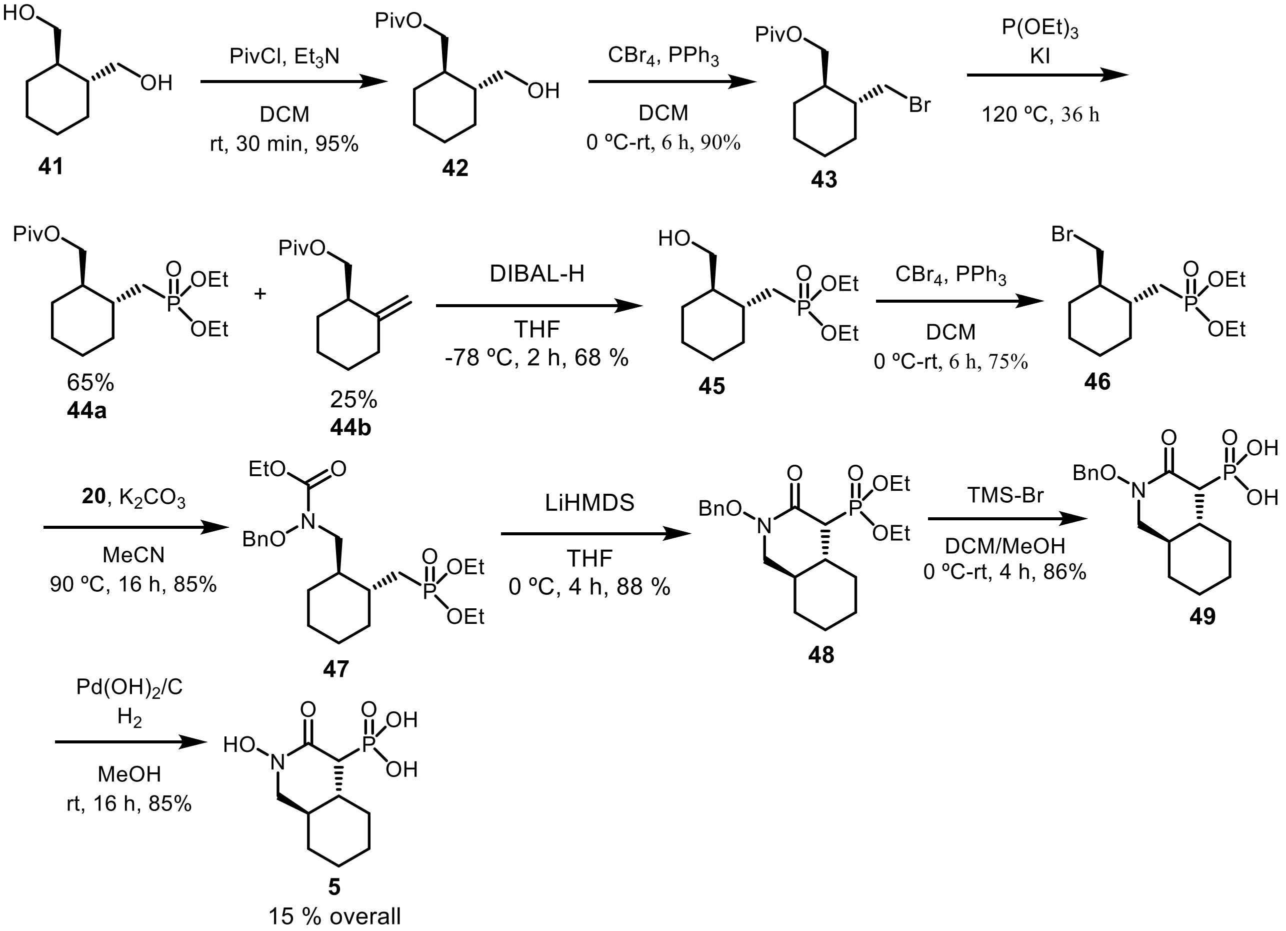

Scheme S5. Synthesis of **5**

((1R,2R)-2-(hydroxymethyl)cyclohexyl)methyl pivalate. In a flame-dried 50 mL round-bottom flask equipped with a magnetic stir bar, ((1R,2R)-cyclohexane-1,2-diyl)dimethanol (**41**, 5 mmol) was dissolved in 10 mL of anhydrous DCM at 0 °C. To this stirring solution was added sodium triethylamine (5 mmol) followed by pivaloyl chloride (2.5 mmol), and the reaction was stirred at room temperature for 6 h. Upon completion, 50 mL of deionized water was added to quench the reaction. The organic layer was extracted with ethyl acetate (3 × 50 mL), and the combined extracts were washed successively with water (50 mL) and saturated brine (50 mL). The organic layer was dried over anhydrous sodium sulfate, filtered, and concentrated. The crude product was purified by flash chromatography on silica gel using a gradient elution from 100% hexanes to 5% EtOAc in hexanes. Compound **42** was obtained as a pale-yellow oil in 95% yield. ^{1}H NMR (500 MHz, $CDCl_3$) δ 4.04 (m, 1H), 3.90 (m, 1H), 3.50 (m, 2H),

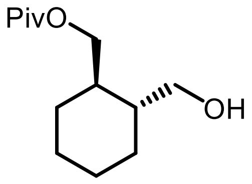

42

3.05 (s, 1H), 1.79 – 1.60 (m, 6H), 1.49 (m, 1H), 1.30 – 1.15 (m, 3H), 1.11 (s, 9H); $^{13}C\{^1H\}$ NMR (126 MHz, $CDCl_3$) δ 178.73, 67.35, 65.08, 41.76, 38.77, 38.40, 29.75, 29.37, 27.09, 25.78, 25.69; HRMS (ESI–TOF) m/z: $[M+H]^+$ Calcd for $C_{13}H_{24}NO_3$ 229.1804; Found 229.1804.

((1R,2R)-2-((diethoxyphosphoryl)methyl)cyclohexyl)methyl pivalate. In a flame-dried 25 mL round-bottom flask equipped with a magnetic stir bar, compound **42** (1 mmol) was dissolved in 5 mL of anhydrous DCM at 0 °C. Carbon tetrabromide (1.2 mmol) and triphenylphosphine (1.2 mmol) were then added sequentially. The reaction mixture was stirred at room temperature for 3 h. Upon completion, hexanes was added to precipitate the excess PPh_3 which was then filtered through a celite pad. The filtrate was dried and concentrated under reduced pressure to afford the ((1R,2R)-2-(bromomethyl)cyclohexyl)methyl pivalate (90%) intermediate which was used in the next step without purification. In a separate flame-dried 50 mL round-bottom flask equipped with a magnetic stir bar, the ((1R,2R)-2-(bromomethyl)cyclohexyl)methyl pivalate intermediate (5 mmol), triethyl phosphite (15 mmol), and potassium iodide (2.5 mmol) were stirred at 120 °C for 36 h. Upon completion, 50 mL of deionized water was added to quench the reaction. The organic layer was extracted with ethyl acetate (3 × 50 mL), and the combined extracts were washed successively with water (50 mL) and saturated brine (50 mL). The organic layer was dried over anhydrous sodium sulfate, filtered, and concentrated. The crude product was purified by flash chromatography on silica gel using a gradient elution from 100% hexanes to 80% EtOAc in hexanes. Compound **44** was obtained as a pale-yellow oil in 95% yield. ^1H NMR (500 MHz, $CDCl_3$) 3.58 – 3.49 (m, 6H), 1.63 – 1.60 (m, 2H), 1.53 – 1.51 (m, 1H), 1.18 (m, 6H), 1.08 – 1.01 (m, 1H), 0.93 (m, 2H), 0.78 (m, 6H), 0.68 (s, 9H)., 1.74 (m, 1H), 1.69 – 1.58 (m, 1H),1.31 (m, 6H), 1.18 (m, 9H); $^{13}C\{^1H\}$ NMR (126 MHz, $CDCl_3$) δ 177.96, 66.49, 60.83 (d, *J* = 6.8 Hz, 1C), 60.72 (d, *J* = 6.7 Hz, 1C), 41.72 (d, *J* = 14.3 Hz, 1C), 41.63, 38.35, 33.83, 32.53, 29.61 (d, *J* = 55.2 Hz, 1C), 28.72, 26.71, 25.10 (d, *J* = 28.5 Hz, 1C), 15.98, 15.94; HRMS (ESI–TOF) m/z: $[M+H]^+$ Calcd for $C_{17}H_{33}O_5P$ 349.2144; Found 349.2145.

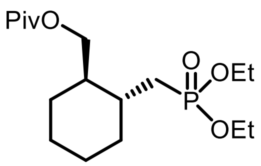

44

diethyl (((1R,2R)-2-(hydroxymethyl)cyclohexyl)methyl)phosphonate. In a flame-dried 25 mL round-bottom flask equipped with a magnetic stir bar, compound **44** (1 mmol) was dissolved in 5 mL of anhydrous THF at –78 °C. Diisobutylaluminum hydride (3 mmol) was added and the reaction mixture was stirred at –78 °C for 2 h. Upon completion, a dilute aqueous solution of acetic acid was added. Next, hexanes was added and the organic layer was extracted with ethyl acetate (3 × 50 mL), and the combined extracts were washed successively with water (50 mL) and saturated brine (50 mL). The organic layer was dried over anhydrous sodium sulfate, filtered, and concentrated. The crude product was purified by flash chromatography on silica gel using a gradient elution from 100% hexanes to 90% EtOAc in hexanes. Compound **45** was

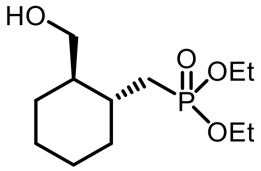

45

obtained as a pale-yellow oil in 68% yield. ^{1}H NMR (500 MHz, $CDCl_3$) 4.04 (m, 6H), 1.95 (m,, 2H), 1.82 (m, 2H), 1.59 (m, 3H), 1.52 (m, 1H), 1.37 (t, *J* = 14.4 Hz, 1H), 1.20 – 1.12 (m, 6H), 1.13 – 0.96 (m, 2H); $^{13}C\{^{1}H\}$ NMR (126 MHz, $CDCl_3$) δ 64.78, 61.64 (d, *J* = 6.5 Hz, 1C), 61.54 (d, *J* = 6.5 Hz, 1C), 41.18 (d, *J* = 5.6 Hz, 1C), 38.44 (d, *J* = 6.8 Hz, 1C), 35.12 (d, *J* = 7.1 Hz, 1C), 34.93, 32.90 (d, *J* = 4.1 Hz, 1C), 29.69, 26.95, 16.38 (d, *J* = 3.5 Hz, 1C), 16.34 (d, *J* = 3.6 Hz, 1C); HRMS (ESI–TOF) m/z: $[M+H]^+$ Calcd for $C_{12}H_{25}O_4P$ 265.1569; Found 265.1565.

diethyl (((1R,2R)-2-(bromomethyl)cyclohexyl)methyl)phosphonate. In a flame-dried 25 mL round-bottom flask equipped with a magnetic stir bar, compound **45** (1 mmol) was dissolved in 5 mL of anhydrous DCM at 0 °C. Carbon tetrabromide (1.2 mmol) and triphenylphosphine (1.2 mmol) were then added sequentially. The reaction mixture was stirred at room temperature for 3 h. Upon completion, hexanes was added to precipitate the excess PPh_3 which was then filtered through celite pad. The filtrate was dried and concentrated under reduced pressure, and the crude product was purified by flash chromatography on silica gel using a gradient elution from 100% hexanes to 20% hexanes in EtOAc. Compound **46** was obtained as a pale-yellow oil in 75% yield. ^{1}H NMR (500 MHz, $CDCl_3$) δ 4.11 (m, 4H), 2.12 – 2.00 (m, 1H), 2.03 – 1.87 (m, 1H), 1.81 (m, 2H), 1.77 – 1.67 (m, 5H), 1.68 – 1.55 (m, 1H), 1.58 – 1.42 (m, 1H), 1.34 (m, 6H), 1.27 (m, 3H); $^{13}C\{^{1}H\}$ NMR (126 MHz, $CDCl_3$) δ 63.17 (d, *J* = 6.5 Hz, 1C), 63.02 (d, *J* = 6.4 Hz, 1C), 45.05 (d, *J* = 13.6 Hz, 1C), 42.84 (d, *J* = 5.2 Hz, 1C), 40.82, 36.93 (d, *J* = 4.2 Hz, 1C), 34.18, 32.59, 28.64, 27.37, 18.10, 18.05; HRMS (ESI–TOF) m/z: $[M+H]^+$ Calcd for $C_{12}H_{24}BrO_3P$ 327.0725; Found 327.0727.

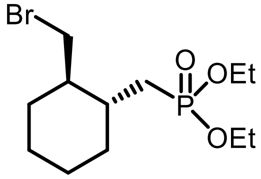

46

ethyl(benzyloxy)(((1R,2R)-2 ((diethoxyphosphoryl)methyl)cyclohexyl)methyl)carbamate. In a flame-dried 100 mL round-bottom flask equipped with a magnetic stir bar, compound **20** (3 mmol) and compound **36** (3.3 mmol) were dissolved in 15 mL anhydrous MeCN. Potassium carbonate (15 mmol) was then added, and the reaction mixture was stirred at 90 °C for 16 h. Upon completion of the reaction, 50 mL of deionized water was added. The organic layer was extracted with ethyl acetate (3 × 50 mL), and the combined organic extracts were washed successively with 50 mL of water and 50 mL of saturated brine. The organic layer was dried over anhydrous sodium sulfate, filtered, dried, and purified by flash chromatography on silica gel, using gradient elution from 100% DCM to 5% MeOH in DCM. Compound **47** was obtained as a pale-yellow oil in 85% yield. ^{1}H NMR (500 MHz, $CDCl_3$) δ 7.45 – 7.39 (m, 5H), 4.88 (m, 2H), 4.24 (m, 2H), 4.08 (m, 4H), 3.56 (dd, *J* = 14.6, 3.8 Hz, 2H), 3.40 (dd, *J* = 14.6, 8.6 Hz, 2H), 2.12 – 2.05 (m, 2H), 2.08 – 1.93 (m, 2H), 1.86 (dt, *J* = 13.3, 3.5 Hz, 2H), 1.64 (m, 4H), 1.33 (m, 9H); $^{13}C\{^{1}H\}$ NMR (126 MHz, $CDCl_3$) δ 157.16, 135.36, 128.44, 128.37, 128.35, 76.58, 61.93, 61.29 (d, *J* = 6.6 Hz, 1C), 61.17 (d, *J* = 6.5 Hz, 1C), 40.63 (d, *J* = 13.8 Hz, 1C), 34.52 (d, *J* = 4.3 Hz, 1C), 32.81, 30.25, 29.66, 29.15, 25.06,

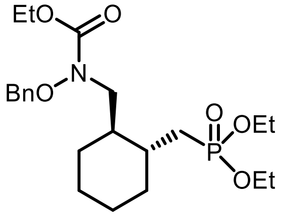

47

24.82, 16.41 (d, J = 2.0 Hz, 1C), 16.37 (d, J = 2.0 Hz, 1C), 14.50; HRMS (ESI–TOF) m/z: $[M+H]^+$ Calcd for $C_{22}H_{36}NO_6P$ 442.2358; Found 442.2359.

diethyl ((4S,4aR,8aR)-2-(benzyloxy)-3-oxodecahydroisoquinolin-4-yl)phosphonate. In a flame-dried 100 mL round-bottom flask equipped with a magnetic stir bar, compound **47** (3 mmol) was dissolved in THF (10 mL) and cooled to 0 °C. A solution of LiHMDS (1.5 M in THF, 6 mL, 9 mmol) was slowly added under a nitrogen atmosphere, and the mixture was stirred at 0°C for 3 h. The reaction was quenched at 0 °C by adding 10% aqueous acetic acid (10 mL), followed by deionized water (50 mL). The organic layer was extracted with ethyl acetate (3 × 50 mL), and the combined organic extracts were washed sequentially with water (50 mL) and saturated brine (50 mL). The organic layer was dried over anhydrous sodium sulfate, filtered, and concentrated. The crude product was purified by flash chromatography on silica gel using a gradient elution from 100% DCM to 5% MeOH in DCM. Compound **48** was isolated as a pale-yellow oil in 88% yield. ^1H NMR (500 MHz, $CDCl_3$) δ 7.49 – 7.40 (m, 5H), 5.07 – 4.83 (m, 2H), 4.31 – 4.15 (m, 4H), 3.28 (dd, J = 12.0, 4.5 Hz, 1H), 3.18 (t, J = 11.6 Hz, 1H), 2.71 (dd, J = 25.6, 8.9 Hz, 1H), 2.33 – 2.26 (m, 1H), 1.74 (m, 3H), 1.69 (m, 6H), 1.38 (s, 6H); $^{13}C\{^1H\}$ NMR (126 MHz, $CDCl_3$) δ 162.24 (d, J = 7.5 Hz), 135.57, 128.62, 128.52, 128.42, 76.21, 63.19 (d, J = 6.9 Hz, 1C), 62.28 (d, J = 6.5 Hz, 1C), 56.45, 48.77 (d, J = 131.6 Hz, 1C), 40.27 (d, J = 10.7 Hz, 1C), 39.39 (d, J = 3.6 Hz, 1C), 33.09, 29.51, 25.36, 24.88, 16.47 (d, J = 6.1 Hz, 1C), 16.33 (d, J = 6.3 Hz, 1C).; HRMS (ESI–TOF) m/z: $[M+H]^+$ Calcd for $C_{20}H_{30}NO_5P$ 396.1940; Found 396.1944.

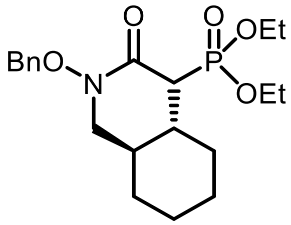

48

((4S,4aR,8aR)-2-(benzyloxy)-3-oxodecahydroisoquinolin-4-yl)phosphonic acid. In a flame-dried 25 mL round-bottom flask equipped with a magnetic stir bar, compound **48** (1 mmol) was dissolved in 5 mL of DCM at 0 °C. Bromotrimethylsilane (3 mmol) was added dropwise to the solution, and the reaction mixture was stirred at room temperature for 4 h. Upon completion of the reaction, 5 mL of methanol was added, and the mixture was evaporated under reduced pressure. This methanol addition and evaporation process was repeated twice more. The resulting crude material was purified by reverse-phase chromatography on C-18 silica gel using a gradient elution system, starting with 100% water and transitioning to 90% water in methanol. Finally, the water/methanol eluent was evaporated under reduced pressure using a rotovap, yielding the target compound **49** as an off-white solid in 86% yield. ^1H NMR (500 MHz, $CDCl_3$) δ 7.37 (m, 3H), 4.97 (s, 2H), 3.28 (dd, J = 12.0, 4.7 Hz, 1H), 3.06 (t, J = 7.5 Hz, 1H), 2.67 (dd, J = 25.2, 9.8 Hz, 1H), 2.45 (d, J = 13.2 Hz, 1H), 1.78 – 1.69 (m, 3H), 1.62 (d, J = 7.6 Hz, 1H), 1.47 (m, 1H), 1.29 (m, 2H), 0.98 (m, 2H); $^{13}C\{^1H\}$ NMR (126 MHz, $CDCl_3$) δ 165.13 (d, J = 4.0 Hz), 134.75, 129.88, 128.96, 128.61, 76.23, 55.67, 47.96 (d, J = 126.5 Hz), 39.13 (d, J = 11.9 Hz), 37.67, 32.36, 29.55, 25.16, 24.82; HRMS (ESI–TOF) m/z: $[M+H]^+$ Calcd for $C_{16}H_{22}NO_5P$ 340.1314; Found 340.1316.

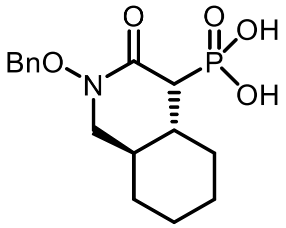

49

((4S,4aR,8aR)-2-hydroxy-3-oxodecahydroisoquinolin-4-yl)phosphonic acid. In a flame-dried 25 mL round-bottom flask equipped with a magnetic stir bar, compound **49** (1 mmol) was dissolved in 5 mL of non-distilled methanol. Pearlman's catalyst (0.2 mmol) was then added, and the flask was sealed and secured with parafilm to ensure airtightness. The reaction vessel was evacuated under vacuum, and a hydrogen balloon was introduced through the septum. The mixture was stirred at room temperature for 2 h. Upon completion, the mixture was filtered through a celite pad and washed with 5 mL of MeOH. The filtrate was dried, yielding compound **5** as a white solid in 85% yield; Mp: 141-142 ˚C; IR (neat): 3576 (m) 3235 (m), 3023 (m), 2992 (m), 1688 (m), 1492 (m), 1493 (m), 1461 (m), 1434 (m), 1383 (s), 1342 (s), 1231 (s), 1221 (m), 1196 (s), 1154 (m), 1075 (s), 1063 (m), 1066 (w), 982 (w), 974 (w), 853 (w), 842 (m), 763 (w), 741 (m), 686 (w), 665 (w), 643 (w), 562 (m) cm^{-1}; ^{1}H NMR (500 MHz, D_2O) δ 3.38 (d, *J* = 6.4 Hz, 1H), 3.26 (d, *J* = 6.3 Hz, 1H), 2.51 (m, 1H), 2.29 (m, 1H), 1.67 (m, 4H), 1.19 (m, 3H), 1.04 – 0.99 (m, 2H); $^{13}C\{^{1}H\}$ NMR (126 MHz, D_2O) δ 163.70, 56.38, 48.12 (d, *J* = 123.7 Hz), 39.05, 38.60, 32.50, 29.63, 25.63, 25.09; HRMS (ESI–TOF) m/z: $[M+H]^+$ Calcd for $C_9H_{17}NO_5P$ 250.0844; Found 250.0843.

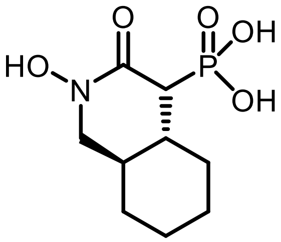

5

1.7 Synthesis of compound 6

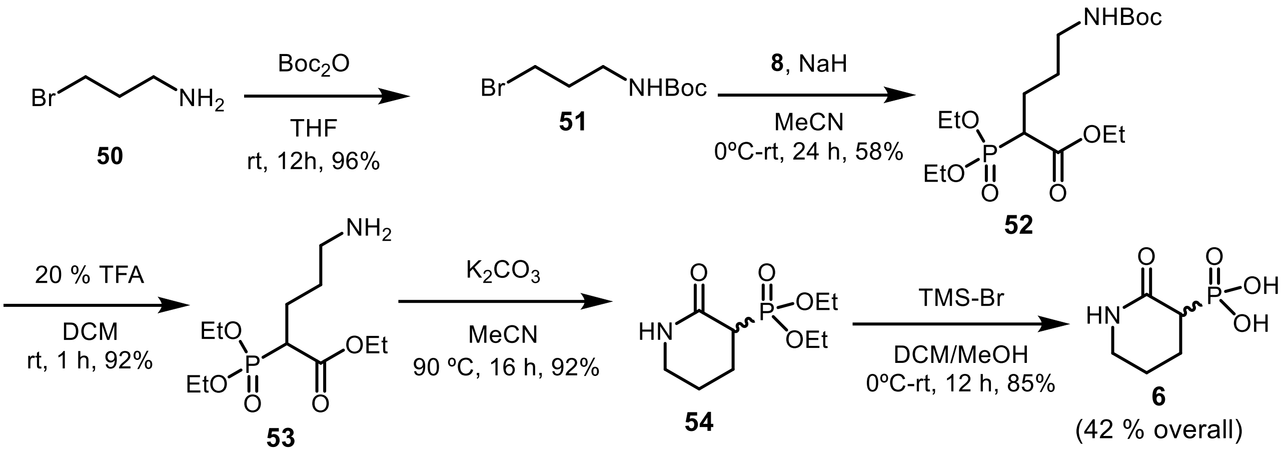

Scheme S6. Synthesis of **6**

***tert*-butyl (3-bromopropyl)carbamate.** In a flame-dried 25 mL round-bottom flask equipped with a magnetic stir bar, 3-bromopropan-1-amine (**50**, 1 mmol) was dissolved in THF (5 mL). Di-*tert*-butyl dicarbonate (2 mmol) was added and the reaction stirred at room temperature for 12 h. Upon completion, 50 mL of deionized water was added and the organic layer was extracted with ethyl acetate (3 × 50 mL). The combined organic extracts were washed sequentially with water (50 mL) and saturated brine (50 mL). The organic layer was dried over anhydrous sodium sulfate, filtered, and concentrated. The crude product was purified by flash chromatography on silica gel using a gradient elution from 100% hexanes to 20% EtOAc in hexanes. Compound **51** was isolated as a pale-yellow oil in 88% yield. ^{1}H NMR (500 MHz, $CDCl_3$) δ 4.97 (s, 1H), 3.37 (t, J = 6.8 Hz, 2H), 3.18 (m, 2H), 1.98 (t, J = 6.5 Hz, 2H), 1.37 (s, 9H); ^{13}C{^{1}H} NMR (126 MHz, $CDCl_3$) δ 156.00, 79.15, 38.94, 32.74, 30.79, 28.35; HRMS (ESI–TOF) m/z: $[M+H]^+$ Calcd for $C_8H_{16}BrNO_2$ 238.0443; Found 238.0445.

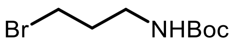

51

ethyl 5-((*tert*-butoxycarbonyl)amino)-2-(diethoxyphosphoryl)pentanoate. In a flame-dried 50 mL round-bottom flask equipped with a magnetic stirrer, ethyl 2-(diethoxyphosphoryl)acetate (**8**, 1 mmol) was dissolved in 5 mL of anhydrous THF at 0 °C. Sodium hydride (1.05 mmol) was then added, and the reaction mixture was stirred at 0 °C for 1 h. Compound **51** (1 mmol) was added dropwise and the reaction mixture allowed to stir at room temperature for 48 h. Upon completion of the reaction, 50 mL of deionized water was added. The organic layer was extracted with ethyl acetate (3 × 50 mL), and the combined organic extracts were washed successively

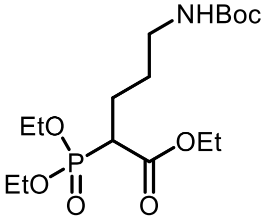

52

with 50 mL of water and 50 mL of saturated brine. The organic layer was dried and purified by flash chromatography on silica gel, using gradient elution from 100% hexanes to 20% hexanes/ethyl acetate. Compound **52** was obtained as a pale-yellow oil in 72% yield. ^{1}H NMR (500 MHz, $CDCl_3$) δ 4.96 (s, 1H), 4.02 – 3.88 (m, 6H), 2.89 (m, 2H), 2.81 – 2.67 (m, 1H), 1.86 – 1.60 (m, 2H), 1.39 – 1.25 (m, 2H), 1.22 (s, 9H), 1.10 (m, 9H).; ^{13}C{^{1}H} NMR (126 MHz, $CDCl_3$) δ 168.78 (d, *J* = 4.7 Hz, 1C), 155.80, 78.53, 62.43 (d, *J* = 3.0 Hz, 1C), 62.37, 61.11, 45.09 (d, *J* = 131.0 Hz, 1C), 39.58, 28.17, 24. 05, 24.01, 16.14, 16.13, 39.89; HRMS (ESI–TOF) m/z: $[M+H]^+$ Calcd for $C_{16}H_{32}NO_7P$ 382.1995; Found 382.1997.

ethyl 5-amino-2-(diethoxyphosphoryl)pentanoate. In a flame-dried 25 mL round-bottom flask equipped with a magnetic stir bar, compound **52** (1 mmol) was dissolved in 10 mL of 20% trifluoroacetic acid in DCM. The reaction mixture was stirred at room temperature for 1 h. Upon completion of the reaction, 50 mL of deionized water was added. The organic layer was extracted with ethyl acetate (3 × 50 mL), and the combined organic extracts were washed successively with 50 mL of water and 50 mL of saturated brine. The organic layer was dried and purified by flash chromatography on silica gel, using gradient elution from 100% DCM to 10% MeOH in DCM. Compound **53** was obtained as off-white oil in 92% yield. ^{1}H NMR (500 MHz, $CDCl_3$) δ 7.89 (m, 6H), 4.24 (m, 2H), 3.08 (m, 3H), 2.13 – 2.03 (m, 1H), 1.96 (ddd, *J* = 21.6, 10.8, 6.4 Hz, 1H), 1.82 (p, *J* = 7.9 Hz, 2H), 1.36 (m, 9H); ^{13}C{^{1}H} NMR (126 MHz, $CDCl_3$) δ 168.62 (d, *J* = 4.8 Hz, 1C), 63.72 (d, *J* = 7.2 Hz, 1C), 63.58 (d, *J* = 6.7 Hz, 1C), 61.97, 44.45 (d, *J* = 133.5 Hz, 1C), 39.37, 25.68 (d, *J* = 14.3 Hz, 1C), 23.51 (d, *J* = 4.4 Hz, 1C), 15.88 (d, *J* = 6.0 Hz, 1C), 15.85 (d, *J* = 6.0 Hz, 1C), 13.63; HRMS (ESI–TOF) m/z: $[M+H]^+$ Calcd for $C_{11}H_{24}NO_5P$ 282.1470; Found 282.1470.

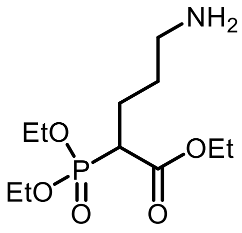

53

diethyl (2-oxopiperidin-3-yl)phosphonate. In a flame-dried 100 mL round-bottom flask equipped with a magnetic stir bar, compound **53** (1 mmol) was dissolved in 15 mL anhydrous MeCN. Potassium carbonate (4 mmol) was then added, and the reaction mixture was stirred at 90 °C for 16 h. Upon completion of the reaction, 50 mL of deionized water was added. The organic layer was extracted with ethyl acetate (3 × 50 mL), and the combined organic extracts were washed successively with 50 mL of water and 50 mL of saturated brine. The organic layer was dried over anhydrous sodium sulfate, filtered, dried, and purified by flash chromatography on silica gel, using gradient elution from 100% DCM to 5% MeOH in DCM. Compound **54** was obtained as pale-yellow oil in 92% yield. ^{1}H NMR (500 MHz, $CDCl_3$) δ 4.22 – 4.17 (m, 3H), 3.10 (dt, *J* = 27.0, 7.3 Hz, 1H), 2.20 – 2.09 (m, 2H), 2.09 – 1.95 (m, 2H), 1.81 – 1.69 (m, 2H), 1.37 – 1.34 (m, 6H); ^{13}C{^{1}H} NMR (126 MHz, $CDCl_3$) δ 167.43 (d, *J* = 4.7 Hz, 1C), 63.09 (d, *J* = 6.9 Hz, 1C), 62.20 (d, *J* = 6.9 Hz, 1C), 41.51, 40.48 (d, *J* = 139.3 Hz, 1C), 22.50 (d, *J* = 4.5 Hz, 1C), 20.50 (d, *J* = 8.2 Hz, 1C), 15.39 (d, *J* = 1.7 Hz, 1C), 15.34 (d, *J* = 1.8 Hz, 1C); HRMS (ESI–TOF) m/z: $[M+H]^+$ Calcd for $C_9H_{18}NO_5P$ 236.1052; Found 236.1053.

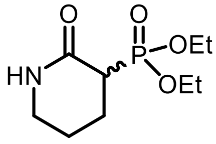

54

(2-oxopiperidin-3-yl)phosphonic acid. In a flame-dried 25 mL round-bottom flask equipped with a magnetic stir bar, compound **54** (1 mmol) was dissolved in 5 mL of DCM at 0 °C. Bromotrimethylsilane (3 mmol) was added dropwise to the solution, and the reaction mixture was stirred at room temperature for 4 h. Upon completion of the reaction, 5 mL of methanol was added, and the mixture was evaporated under reduced pressure. This methanol addition and evaporation process was repeated twice more. The resulting crude material was purified by reverse-phase chromatography on C-18 silica gel using a gradient elution system, starting with 100% water and transitioning to 90% water in methanol. Finally, the water/methanol eluent was evaporated under reduced pressure using a rotovap, yielding the target compound **6** as an pale yellow solid in 85% yield; Mp: 135-136 ˚C; IR (neat): 3523 (w) 3419 (m), 3051 (m), 2983 (m), 1723 (s), 1481 (m), 1495 (m), 1434 (m), 1427 (m), 1397 (s), 1333 (s), 1270 (s), 1232 (m), 1195 (s), 1147 (m), 1088 (s), 1072 (m), 1068 (w), 994 (w), 967 (w), 825 (w), 815 (m), 786 (w), 743 (m), 686 (w), 642 (w), 658 (w), 575 (m) cm^{-1}; ^{1}H NMR (500 MHz, D_2O) δ 3.14 – 3.01 (m, *J* = 6.0 Hz, 2H), 2.81 (dt, *J* = 26.6, 14.4 Hz, 1H), 1.96 – 1.83 (m, 2H), 1.84 – 1.77 (m, 1H), 1.80 – 1.68 (m, 2H), 1.55 – 1.44 (m, 1H); ^{13}C{^{1}H} NMR (126 MHz, D_2O) δ 170.31, 41.58, 40.60 (d, *J* = 132.3 Hz, 1C), 21.75 (d, *J* = 3.9 Hz, 1C), 19.74 (d, *J* = 8.1 Hz, 1C); HRMS (ESI–TOF) m/z: $[M+H]^+$ Calcd for $C_5H_{10}NO_4P$ 180.0426; Found 180.0427.

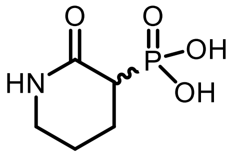

6

1.8 Synthesis of compound 7

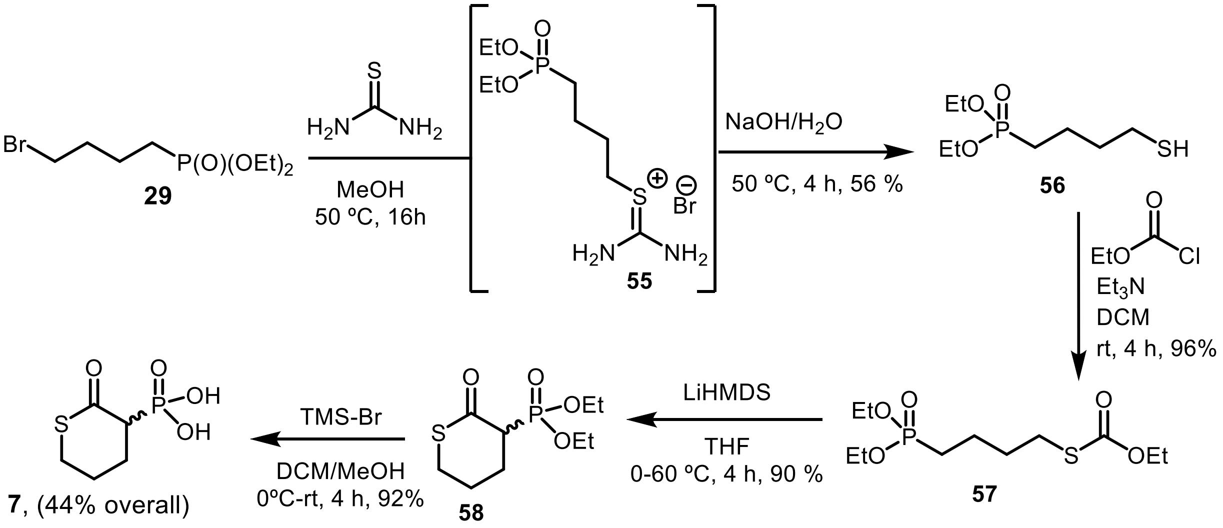

Scheme S7. Synthesis of **7**

S-(4-(diethoxyphosphoryl)butyl) O-ethyl carbonothioate. In a flame-dried 100 mL round-bottom flask equipped with a magnetic stir bar, compound **29** (6 mmol) and thiourea (2 mmol) were dissolved in 20 mL of methanol. The reaction mixture was stirred at 50 °C for 16 h, after which sodium hydroxide (18 mmol) was added, and the mixture was stirred for an additional 2 h at 50 °C. Upon completion, the solvent was evaporated, and the resulting crude diethyl (4-mercaptobutyl)phosphonate (**56**, 56%) intermediate was used in the next step without further purification. In a separate flame-dried 25 mL round-bottom flask equipped with a magnetic stirrer, **56** (2 mmol) was dissolved in 5 mL of dichloromethane (DCM). Triethylamine (2 mmol) was added, followed by ethyl chloroformate (2 mmol). The reaction mixture was stirred at room temperature for 4 h. After completion of the reaction, 50 mL of deionized water was added. The organic layer was extracted with ethyl acetate (3 × 50 mL), and the combined organic extracts were washed sequentially with 50 mL of water and 50 mL of saturated brine. The organic layer was dried over anhydrous sodium sulfate, filtered, and concentrated. The crude product was purified by flash chromatography on silica gel, using a gradient elution from 100% DCM to 5% MeOH in DCM. Compound **57** was obtained as a pale-yellow oil in 96% yield. ^{1}H NMR (500 MHz, $CDCl_3$) δ 4.06 (q, *J* = 7.1 Hz, 2H), 3.90 – 3.88 (m, 4H), 2.65 (t, *J* = 6.9 Hz, 2H), 2.32 (m, 2H), 1.57 – 1.46 (m, 4H), 1.12 – 1.10 (m, 9H); ^{13}C{^{1}H} NMR (126 MHz, $CDCl_3$) δ 170.57, 63.18, 61.23 (d, *J* = 6.3 Hz, 1C), 61.17(d, *J* = 6.4 Hz, 1C), 30.02 (d, *J* = 1.7 Hz, 1C), 25.94 – 25.23 (d, *J* = 3.4 Hz, 1C), 24.39, 21.32 (d, *J* = 4.8 Hz, 1C), 16.28, 16.24, 14.08; HRMS (ESI–TOF) m/z: $[M+H]^+$ Calcd for $C_{11}H_{23}O_5PS$ 299.1083; Found 299.1084.

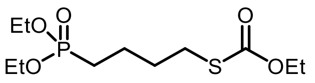

57

diethyl (2-oxotetrahydro-2H-thiopyran-3-yl)phosphonate. In a flame-dried 100 mL round-bottom flask equipped with a magnetic stir bar, compound **57** (3 mmol) was

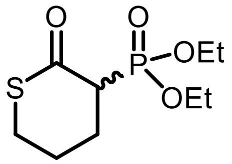

58

dissolved in 10 mL of tetrahydrofuran (THF) and cooled to 0 °C. Under a nitrogen atmosphere, a solution of lithium hexamethyldisilazide (LiHMDS, 1.5 M in THF, 6 mL, 9 mmol) was added slowly. The reaction mixture was stirred at room temperature and subsequently heated to 60 °C, where it was stirred for 3 h. The reaction was quenched at room temperature by adding 10 mL of 10% aqueous acetic acid (AcOH), followed by 50 mL of deionized water. The organic layer was extracted with ethyl acetate (3 × 50 mL), and the combined organic extracts were washed sequentially with 50 mL of water and 50 mL of saturated brine. The organic layer was dried over anhydrous sodium sulfate, filtered, and concentrated. The crude product was purified by flash chromatography on silica gel using a gradient elution from 100% DCM to 5% MeOH in DCM. Compound **58** was isolated as a pale-yellow oil in 90% yield. ^{1}H NMR (500 MHz, $CDCl_3$) δ 4.15 – 4.11 (m, 4H), 2.95 – 2.86 (m, 1H), 2.53 – 2.46 (m, 2H), 2.07 – 1.89 (m, 2H), 1.73 – 1.52 (m, 2H), 1.33 – 1.31 (m, 6H); ^{13}C{^{1}H} NMR (126 MHz, $CDCl_3$) δ 169.01 (d, *J* = 5.1 Hz, 1C), 62.74 (d, *J* = 6.6 Hz, 1C), 62.67, 45.43 (d, *J* = 131.2 Hz, 1C), 30.87, 28.28 (d, *J* = 14.9 Hz, 1C), 26.23 (d, *J* = 4.8 Hz, 1C), 16.38 (d, *J* = 3.8 Hz, 1C), 16.33 (d, *J* = 3.9 Hz, 1C); HRMS (ESI–TOF) m/z: $[M+H]^+$ Calcd for $C_9H_{17}O_4PS$ 253.0663; Found 253.0665.

(2-oxotetrahydro-2H-thiopyran-3-yl)phosphonic acid. In a flame-dried 25 mL round-bottom flask equipped with a magnetic stirrer, compound **58** (1 mmol) was dissolved in 5

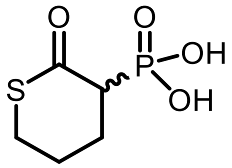

7

mL of DCM at 0 °C. Bromotrimethylsilane (3 mmol) was added dropwise to the solution, and the reaction mixture was stirred at room temperature for 4 h. Upon completion of the reaction, 5 mL of methanol was added, and the mixture was evaporated under reduced pressure. This methanol addition and evaporation process was repeated twice more. The resulting crude material was purified by reverse-phase chromatography on C-18 silica gel using a gradient elution system, starting with 100% water and transitioning to 90% water in methanol. Finally, the water/methanol eluent was evaporated under reduced pressure using a rotovap, yielding the target compound **7** as a yellow oil in 92% yield; IR (neat): 3551 (w), 3034 (m), 2988 (m), 1745 (s), 1481 (m), 1491 (m), 1396 (s), 1324 (s), 1272 (s), 1248 (m), 1174 (s), 1087 (s), 1066 (m), 1031 (w), 982 (w), 956 (w), 843 (w), 837 (m), 756 (w), 734 (m), 696 (w), 612 (w), 615 (w), 540 (m) cm^{-1}; ^{1}H NMR (500 MHz, DMSO-d_6) δ 2.71 (ddd, *J* = 22.3, 11.0, 4.1 Hz, 1H), 2.52 – 2.48 (m, 2H), 1.95 – 1.80 (m, 1H), 1.77 (m, 1H), 1.50 (m, 2H); ^{13}C{^{1}H} NMR (126 MHz, D_2O) 172.34 (d, *J* = 4.9 Hz, 1C), 46.49 (d, *J* = 124.7 Hz, 1C), 29.86, 27.56 (d, *J* = 14.8 Hz, 1C), 25.95 (d, *J* = 4.3 Hz, 1C); HRMS (ESI–TOF) m/z: $[M+H]^+$ Calcd for $C_5H_9O_4PS$ 197.0037; Found 197.0035.

2.0 X-ray crystallographic data

2.1 X-ray crystallography experimental studies

Single crystals of compounds **1** and **2** were grown from a 1:1 mixture of DCM/hexane and used for the X-ray crystallographic analysis. The X-ray intensity data were measured using Mo Kα radiation (λ = 0.71073 Å) with a Bruker D8 Quest diffractometer and a Photon 3 detector. Data were processed and corrected for absorption using the SAINT and SADABS routines in the Apex3 software package (Apex3; Bruker AXS Inc.: Madison, WI, USA, 2015). Structure solution and space group determination were performed by intrinsic phasing (SHELXT)[3], and the structures were subsequently refined using full-matrix least squares techniques on F^2 (SHELXL).[4] All non-hydrogen atoms were refined anisotropically. Hydrogen atoms attached to carbon atoms were placed in calculated positions using riding models. Hydrogen atoms attached to nitrogen and oxygen atoms were identified from the difference electron density map and their positions and isotropic displacement parameters fully refined. Details of the data collection and refinement are provided in Table S1 below. Complete crystallographic data are available in CIF form through the Cambridge Crystallographic Data Centre, deposition numbers 2434790 and 2434791.

Table S1. Crystallographic data for compounds **1** and **2**.

	**1**	**2**
empirical formula	$C_5H_{13}N_2O_5P$	$C_9H_{10}NO_5P$
formula wt. (g/mol)	212.14	243.15
crystal system	orthorhombic	monoclinic
space group, *Z*	$P2_12_12_1$, 4	$P2_1/n$, 4
temperature (K)	150(2)	300(2)
a (Å)	6.1084(3)	8.3552(2)
b (Å)	8.9906(5)	6.4722(2)
c (Å)	15.9200(9)	18.7224(5)
α (°)	90	90
β (°)	90	90.8761(9)
γ (°)	90	90
volume (Å^3)	874.30(8)	1012.32(5)
D_{calc} (g/cm^3)	1.612	1.595
crystal size (mm)	0.03 x 0.08 x 0.30	0.10 x 0.15 x 0.20
absorption coeff. (mm^{-1})	0.310	0.277
F(000)	448	504
Tmax, Tmin	1.000, 0.926	1.000, 0.941
Θ range for data	3.42-27.94	4.36-28.72
reflections coll.	48153	24087
data/restr./param.	2086/0/142	2603/1/157
R(int)	0.0725	0.0709

final R1, wR2 [$I > 2\sigma(I)$]	0.0314, 0.0774	0.0461, 0.1065
final R1, wR2 (all data)	0.0331, 0.0787	0.0592, 0.1202
goodness-of-fit on F^2	1.102	1.081
largest diff. peak, hole (eÅ^{-3})	0.403, -0.208	0.346, -0.404
absolute struct. param. (Flack)	-0.05(4)	-
CCDC Deposition No.	2434790	2434791

2.2 Crystal structure descriptions of compound 1

Compound **1** crystallized as a monohydrate, $C_5H_{11}N_2O_4P{\cdot}H_2O$, as the (R) enantiomer in the chiral space group $P2_12_12_1$. The absolute structure is supported by the Flack parameter of -0.05(4). The compound takes the zwitterionic form shown in Figure S2.1. The hydrogen atom locations on the nitrogen and oxygen atoms were derived from the difference electron density map, fully refined, and supported by resulting hydrogen bonding interactions. This hydrogen bonding is extensive and integral to the long range structure. In particular, the water molecule facilitates strong hydrogen bonds as an acceptor for the P-OH hydrogen bond donor (H···A = 1.63(5) Å) of one molecule of **1**, and as a hydrogen bond donor toward the P-O acceptors (H···A = 1.77(4) Å and 1.89(4) Å) of two additional molecules of **1**. These form chains propagating along the *a*-axis. The chains are further connected into a three-dimensional framework through four N-H···O hydrogen bonds originating from the ammonium donor.

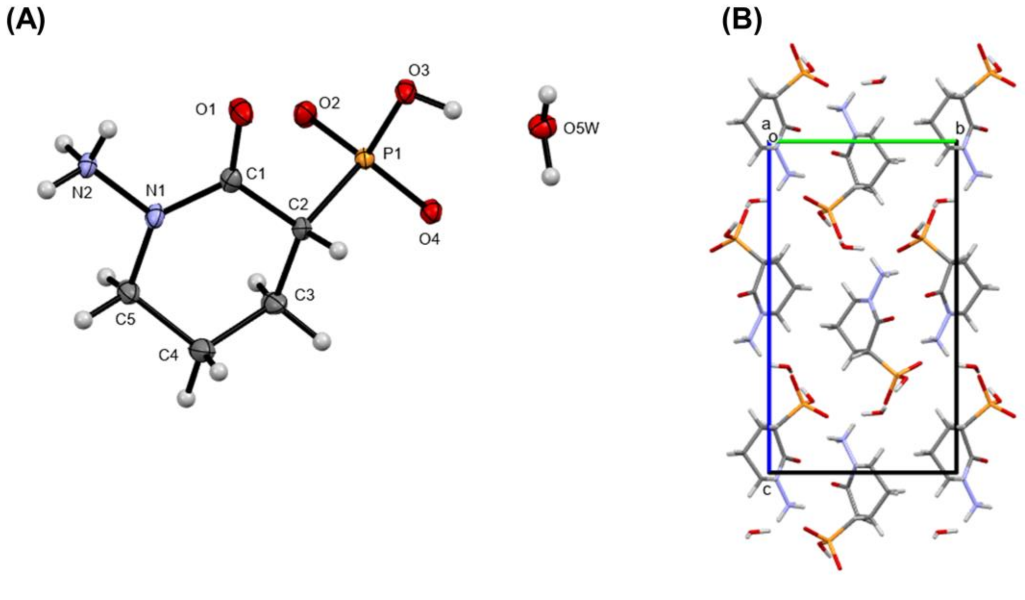

Figure S1 XRD structure (A) and the packing diagram (B) of **1**. Displacement ellipsoids in (A) are shown at the 50% probability level.

2.3 Crystal structure descriptions of compound 2

Compound **2** crystallized as the racemate in the centrosymmetric space group $P2_1/n$. Hydrogen atoms on the N-OH and P-OH entities were again fully refined based on the difference electron density map. Neighboring molecules form inversion-symmetric dimers via hydrogen bonds between P-OH donors and P=O acceptors (H···A = 1.763(18) Å). These dimers extend into sheets in the *ab* plane through O-H···O interactions between the N-OH donors and P=O acceptors (H···A = 1.77(3) Å) and O-H···O interactions between P-OH donors and C=O acceptors (H···A = 1.67(3) Å).

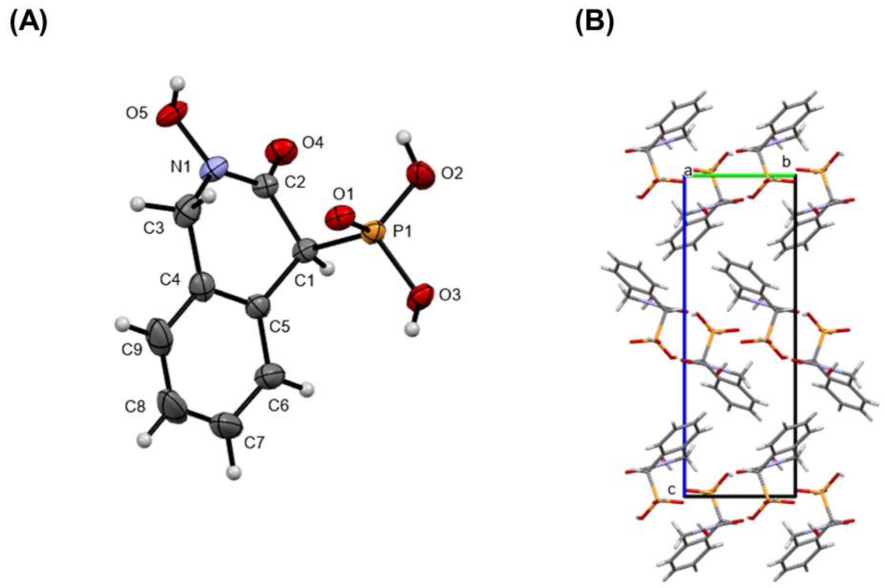

Figure S2 XRD structure (A) and the packing diagram (B) of **2**. Displacement ellipsoids in (A) are shown at the 50% probability level.

3. 0 References

1. Fujii, A.; Cook, E. S. Probiotics. Antistaphylococcal and Antifibrinolytic Activities of. Omega.-Guanidino Acids and. Omega.-Guanidinoacyl-L-Histidines. *Journal of Medicinal Chemistry* **1973**, *16* (12), 1409–1411.
2. King, J. F.; Skonieczny, S.; Poole, G. A. Betylates. 4. The synthesis and preparative nucleophilic substitution reactions of alkyl S-[3]betylates. *Can. J. Chem.* **1983**, *61* (2), 235–243.
3. Sheldrick, G. M. SHELXT-Integrated Space-Group and Crystal-Structure Determination. *Acta Crystallographica Section A: Foundations and Advances* **2015**, *71*, 3–8.
4. Sheldrick, G. M. Crystal Structure Refinement with SHELXL. *Acta Crystallogr. C Struct. Chem.* **2015**, *71* (Pt 1), 3–8.

4.0 NMR Spectra

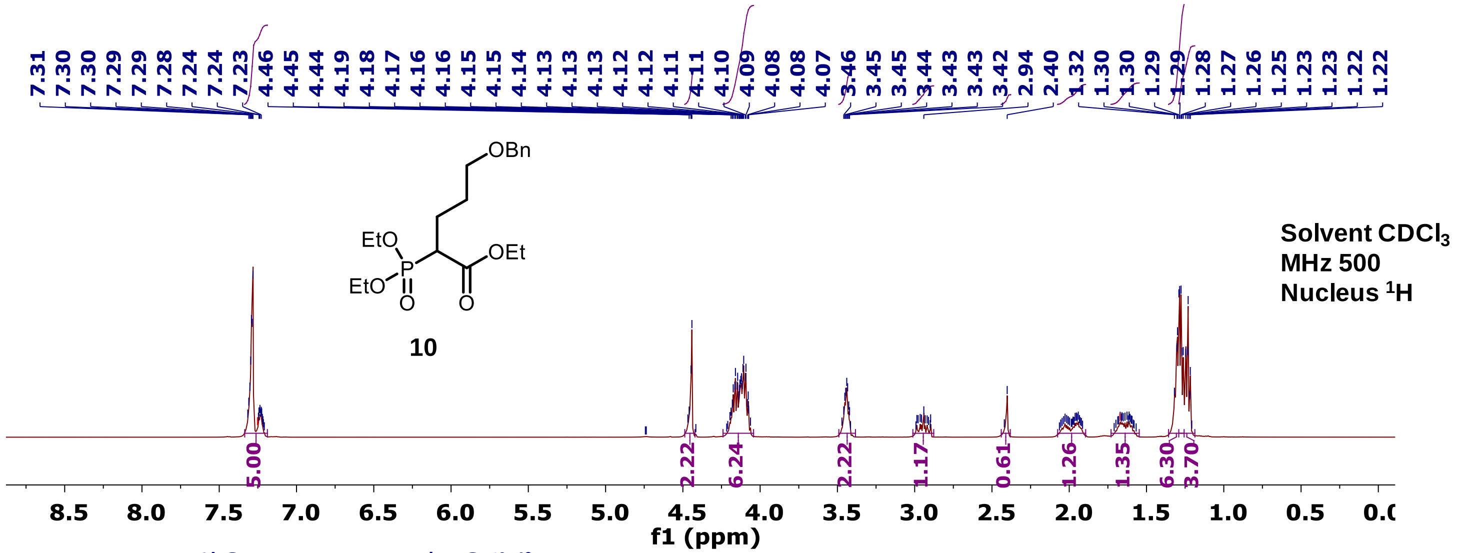

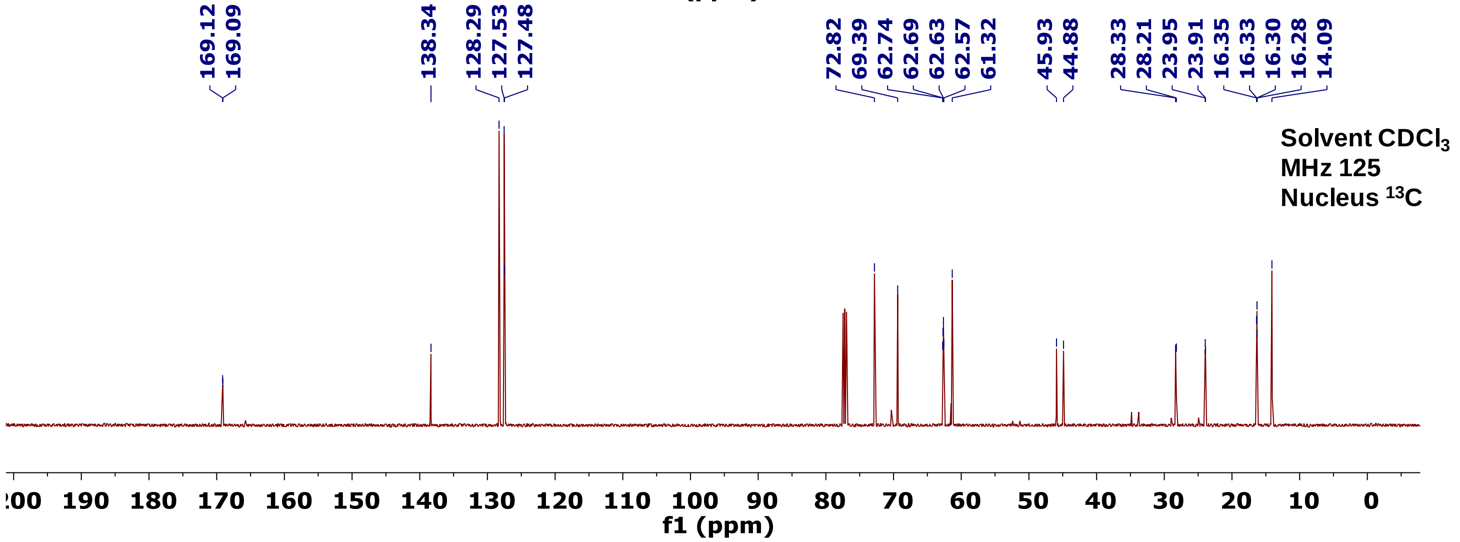

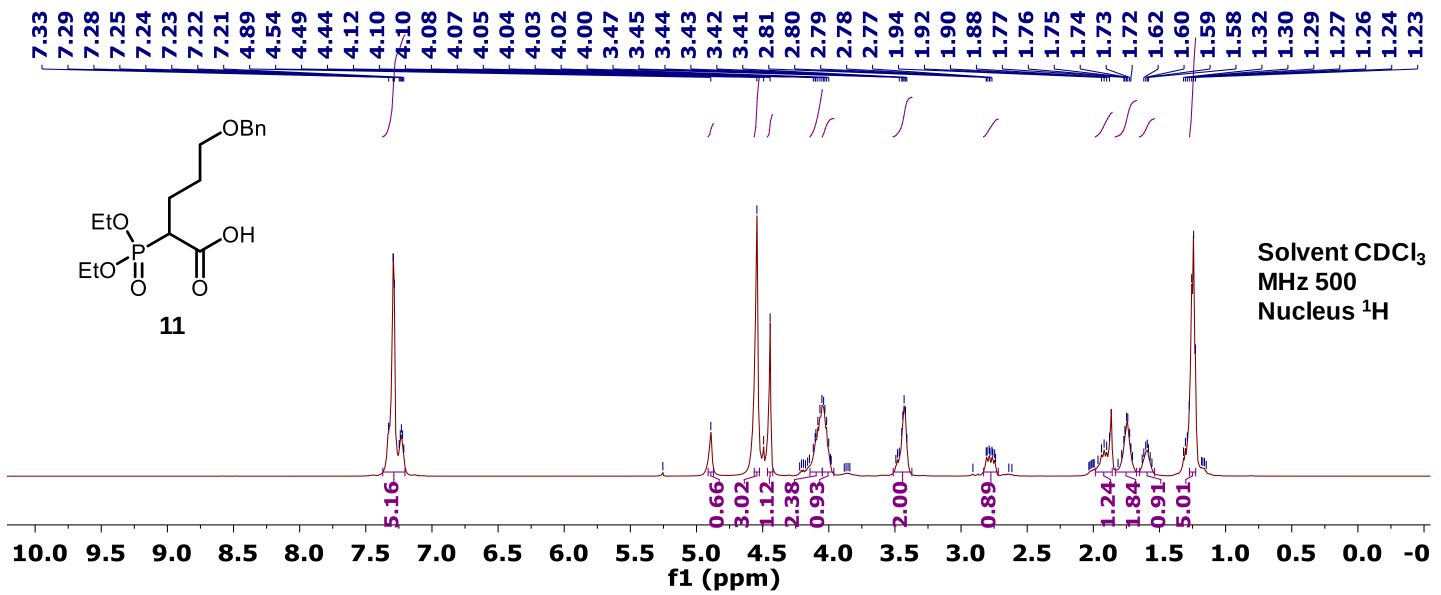
7.33 7.29 7.28 7.25 7.24 7.23 7.22 7.21 4.89 4.54 4.49 4.44 4.12 4.10 4.10 4.08 4.07 4.05 4.04 4.03 4.02 4.00 3.47 3.45 3.44 3.43 3.42 3.41 2.81 2.80 2.79 2.78 2.77 1.94 1.92 1.90 1.88 1.77 1.76 1.75 1.74 1.73 1.72 1.62 1.60 1.59 1.58 1.32 1.30 1.29 1.27 1.26 1.24 1.23
OBn
EtO
P
EtO
O
OH
O
11
Solvent $CDCl_3$
MHz 500
Nucleus ^1H
5.16
0.66
3.02
1.12
2.38
0.93
2.00
0.89
1.24
1.84
0.91
5.01
10.0 9.5 9.0 8.5 8.0 7.5 7.0 6.5 6.0 5.5 5.0 4.5 4.0 3.5 3.0 2.5 2.0 1.5 1.0 0.5 0.0 -0
f1 (ppm)

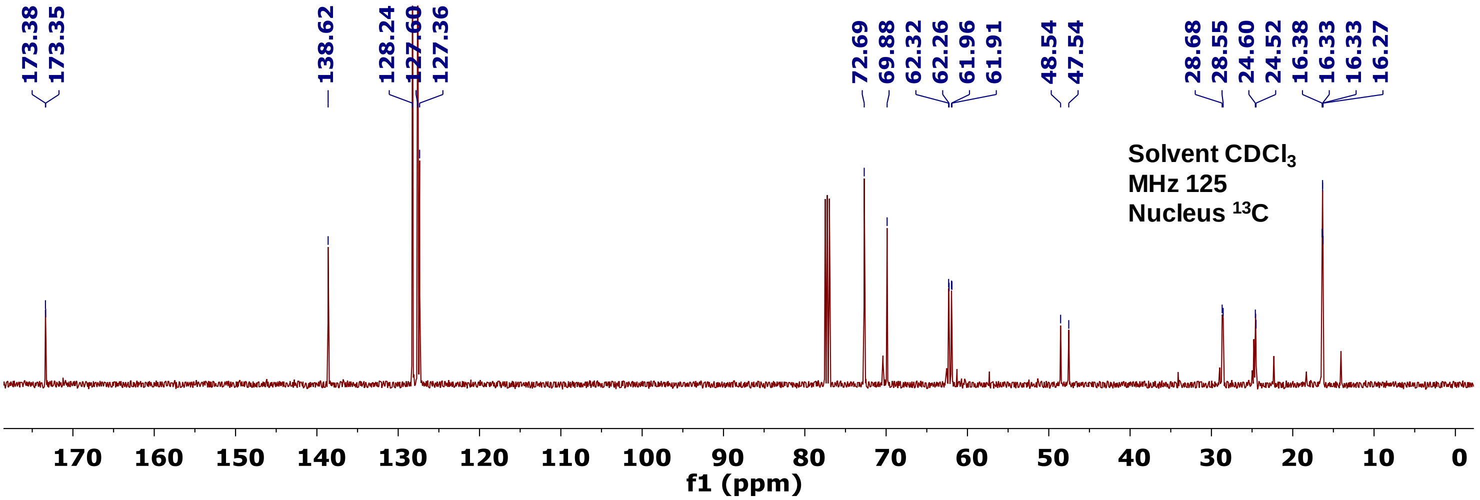
173.38 173.35 138.62 128.24 127.60 127.36 72.69 69.88 62.32 62.26 61.96 61.91 48.54 47.54 28.68 28.55 24.60 24.52 16.38 16.33 16.33 16.27
Solvent $CDCl_3$
MHz 125
Nucleus ^{13}C
170 160 150 140 130 120 110 100 90 80 70 60 50 40 30 20 10 0
f1 (ppm)

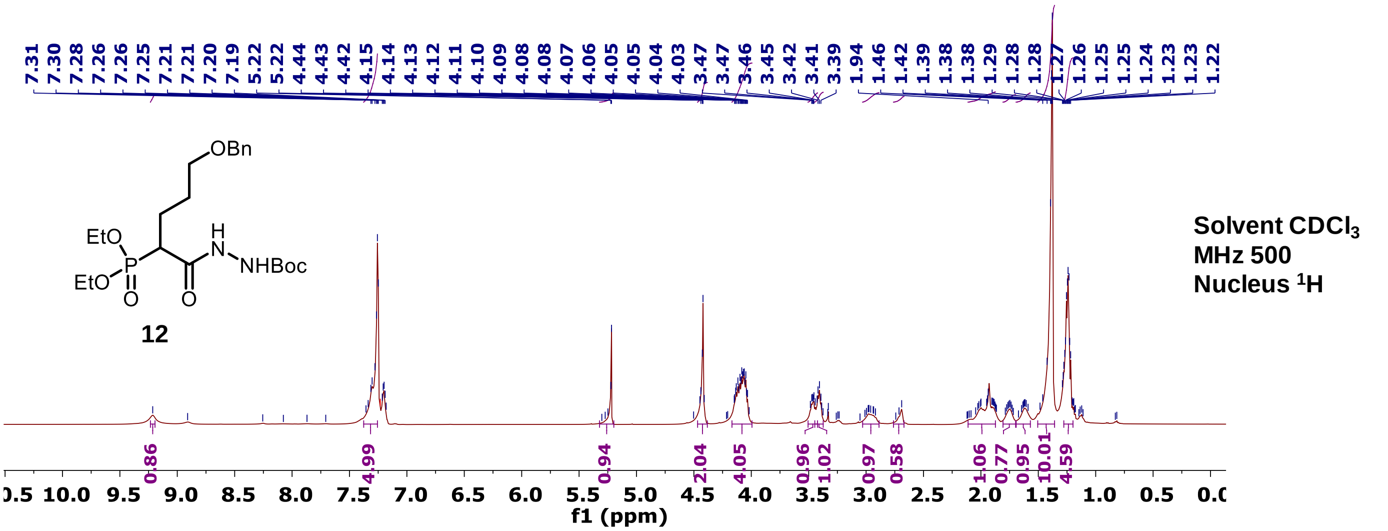
7.31 7.30 7.28 7.26 7.26 7.25 7.21 7.21 7.20 7.19 5.22 5.22 4.44 4.43 4.42 4.15 4.14 4.13 4.12 4.11 4.10 4.09 4.08 4.08 4.07 4.06 4.05 4.05 4.04 4.03 3.47 3.47 3.46 3.45 3.42 3.41 3.39 1.94 1.46 1.42 1.39 1.38 1.38 1.29 1.28 1.28 1.27 1.26 1.25 1.25 1.24 1.23 1.23 1.22
OBn
EtO
EtO
P
O
H
N
NHBoc
O
12
Solvent $CDCl_3$
MHz 500
Nucleus ^1H
0.86 4.99 0.94 2.04 4.05 0.96 1.02 0.97 0.58 1.06 0.77 0.95 10.01 4.59
f1 (ppm)

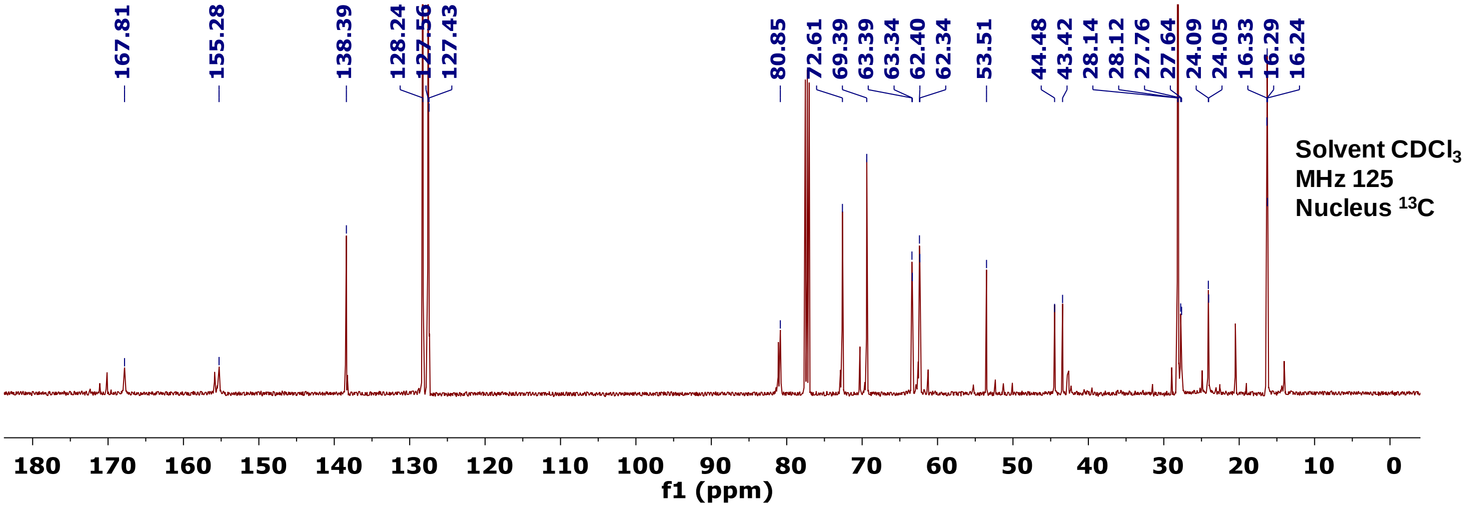
167.81 155.28 138.39 128.24 127.56 127.43 80.85 72.61 69.39 63.39 63.34 62.40 62.34 53.51 44.48 43.42 28.14 28.12 27.76 27.64 24.09 24.05 16.33 16.29 16.24
Solvent $CDCl_3$
MHz 125
Nucleus ^{13}C
180 170 160 150 140 130 120 110 100 90 80 70 60 50 40 30 20 10 0
f1 (ppm)

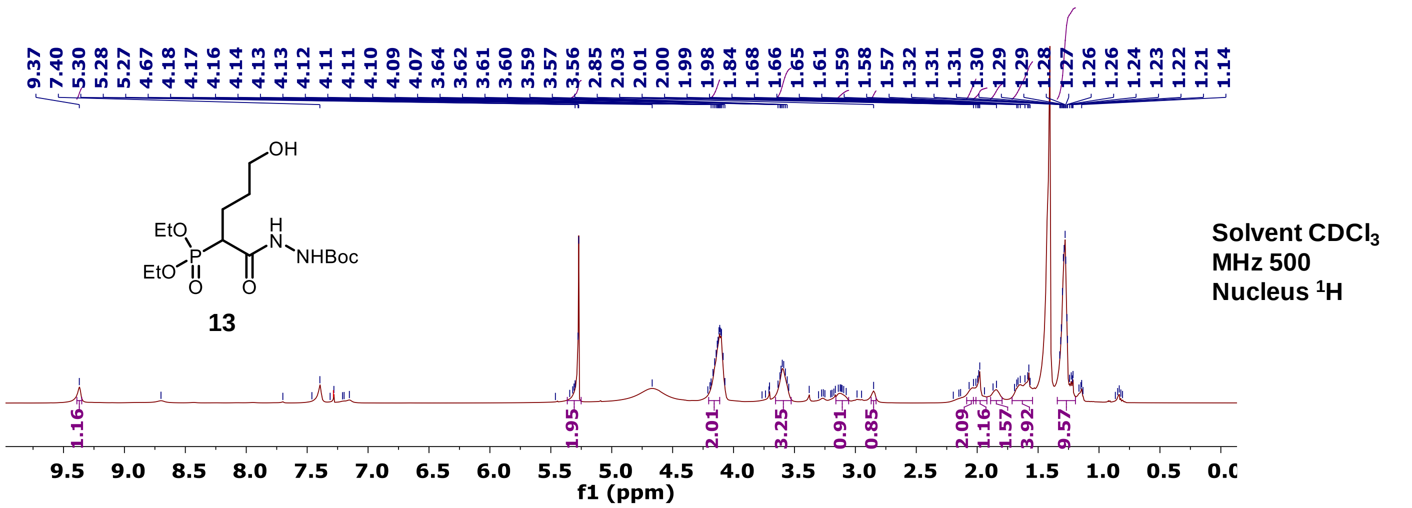
9.37
7.40
5.30
5.28
5.27
4.67
4.18
4.17
4.16
4.14
4.13
4.13
4.12
4.11
4.11
4.10
4.09
4.07
3.64
3.62
3.61
3.60
3.59
3.57
3.56
2.85
2.03
2.01
2.00
1.99
1.98
1.84
1.68
1.66
1.65
1.61
1.59
1.58
1.57
1.32
1.31
1.31
1.30
1.29
1.29
1.28
1.27
1.26
1.26
1.24
1.23
1.22
1.21
1.14
OH
EtO
EtO
P
O
H
N
NHBoc
O
13
Solvent CDCl3
MHz 500
Nucleus 1H
1.16
1.95
2.01
3.25
0.91
0.85
2.09
1.16
1.57
3.92
9.57
9.5
9.0
8.5
8.0
7.5
7.0
6.5
6.0
5.5
5.0
4.5
4.0
3.5
3.0
2.5
2.0
1.5
1.0
0.5
0.0
f1 (ppm)

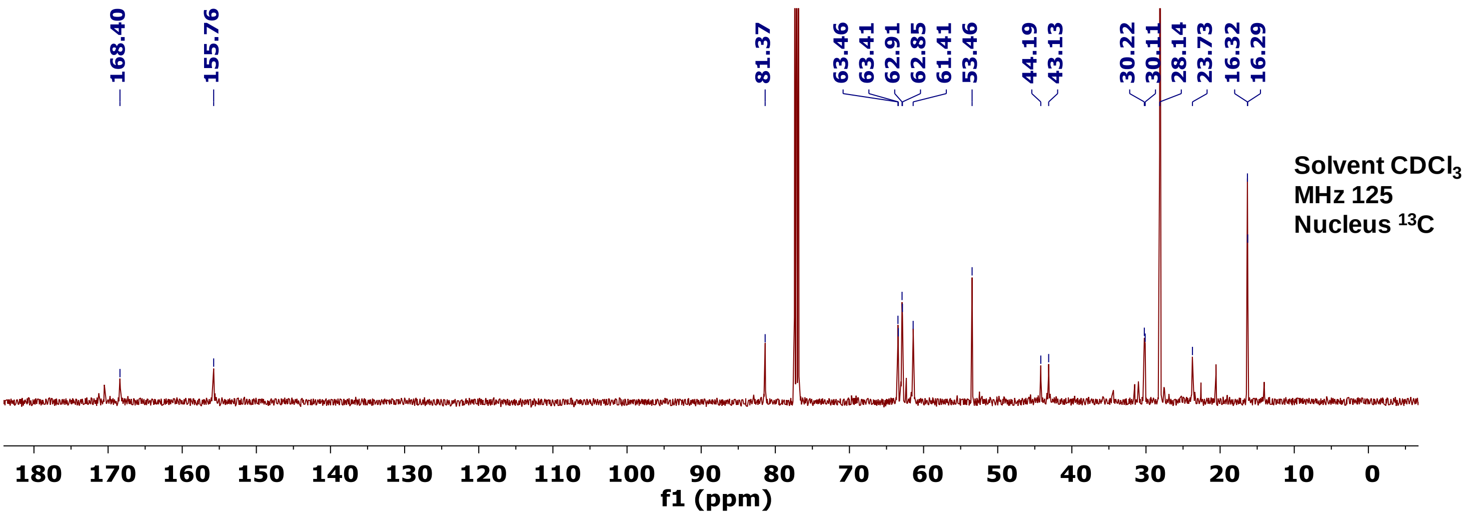
168.40
155.76
81.37
63.46
63.41
62.91
62.85
61.41
53.46
44.19
43.13
30.22
30.11
28.14
23.73
16.32
16.29
Solvent CDCl3
MHz 125
Nucleus 13C
180
170
160
150
140
130
120
110
100
90
80
70
60
50
40
30
20
10
0
f1 (ppm)

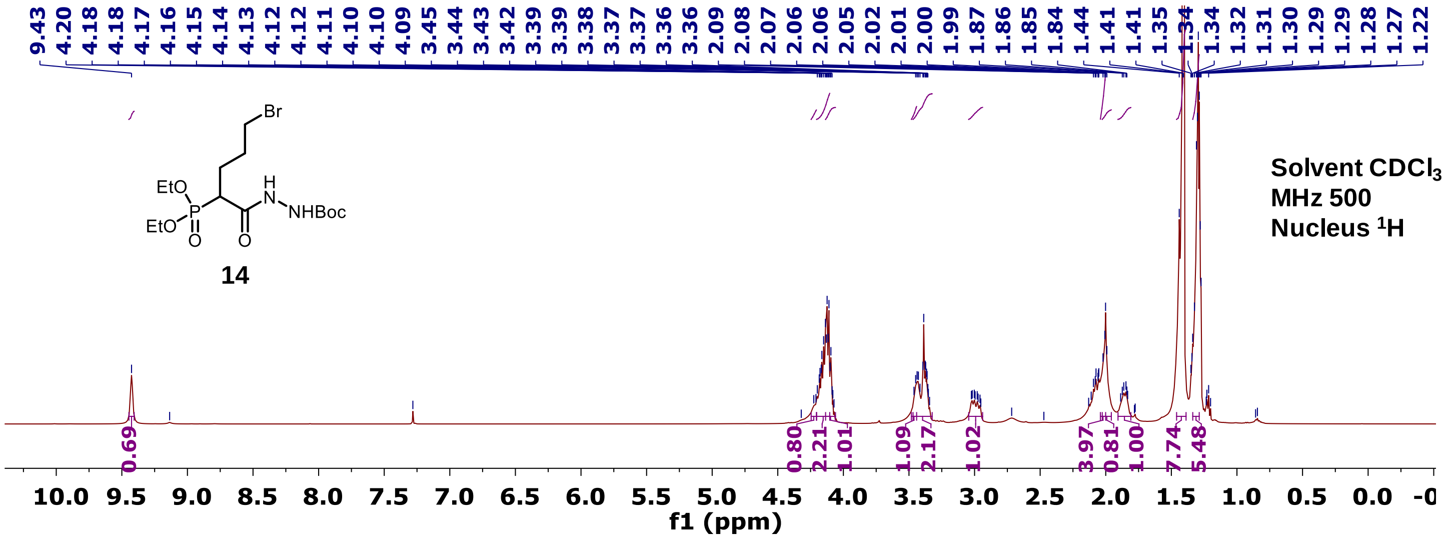
9.43 4.20 4.18 4.18 4.17 4.16 4.15 4.14 4.13 4.12 4.12 4.11 4.10 4.10 4.09 3.45 3.44 3.43 3.42 3.39 3.39 3.38 3.37 3.37 3.36 3.36 2.09 2.08 2.07 2.06 2.06 2.05 2.02 2.01 2.00 1.99 1.87 1.86 1.85 1.84 1.44 1.41 1.41 1.35 1.34 1.34 1.32 1.31 1.30 1.29 1.29 1.28 1.27 1.22
Br
EtO
EtO
P
O
H
N
NHBoc
O
14
Solvent CDCl3
MHz 500
Nucleus 1H
0.69 0.80 2.21 1.01 1.09 2.17 1.02 3.97 0.81 1.00 7.74 5.48
10.0 9.5 9.0 8.5 8.0 7.5 7.0 6.5 6.0 5.5 5.0 4.5 4.0 3.5 3.0 2.5 2.0 1.5 1.0 0.5 0.0
f1 (ppm)

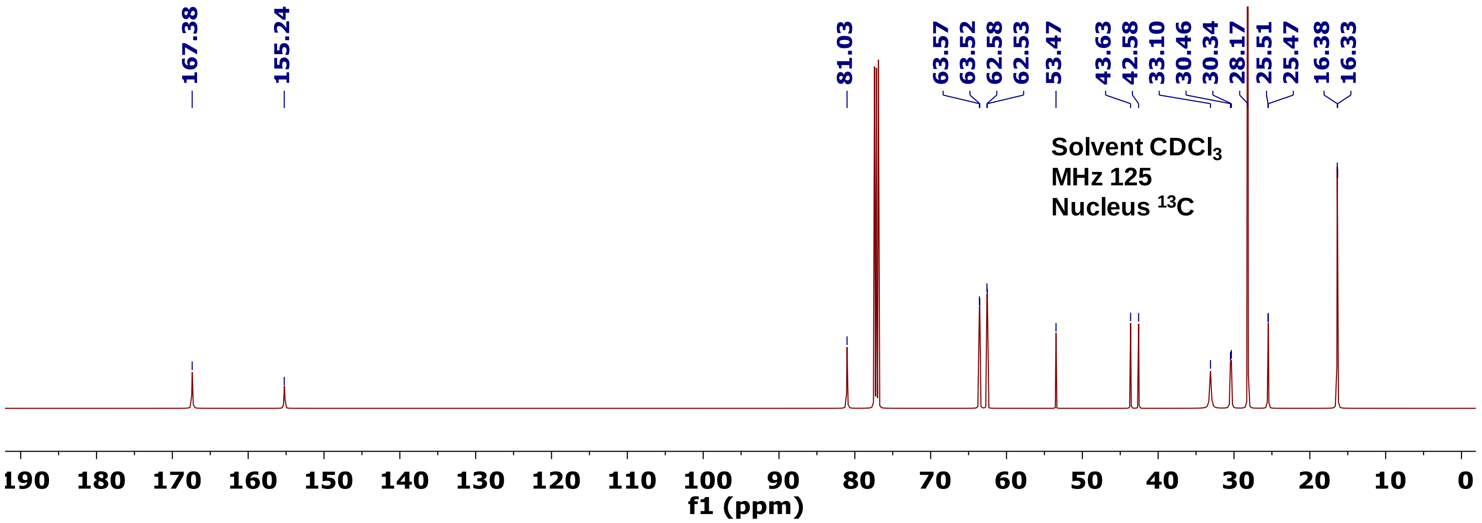
167.38 155.24 81.03 63.57 63.52 62.58 62.53 53.47 43.63 42.58 33.10 30.46 30.34 28.17 25.51 25.47 16.38 16.33
Solvent CDCl3
MHz 125
Nucleus 13C
190 180 170 160 150 140 130 120 110 100 90 80 70 60 50 40 30 20 10 0
f1 (ppm)

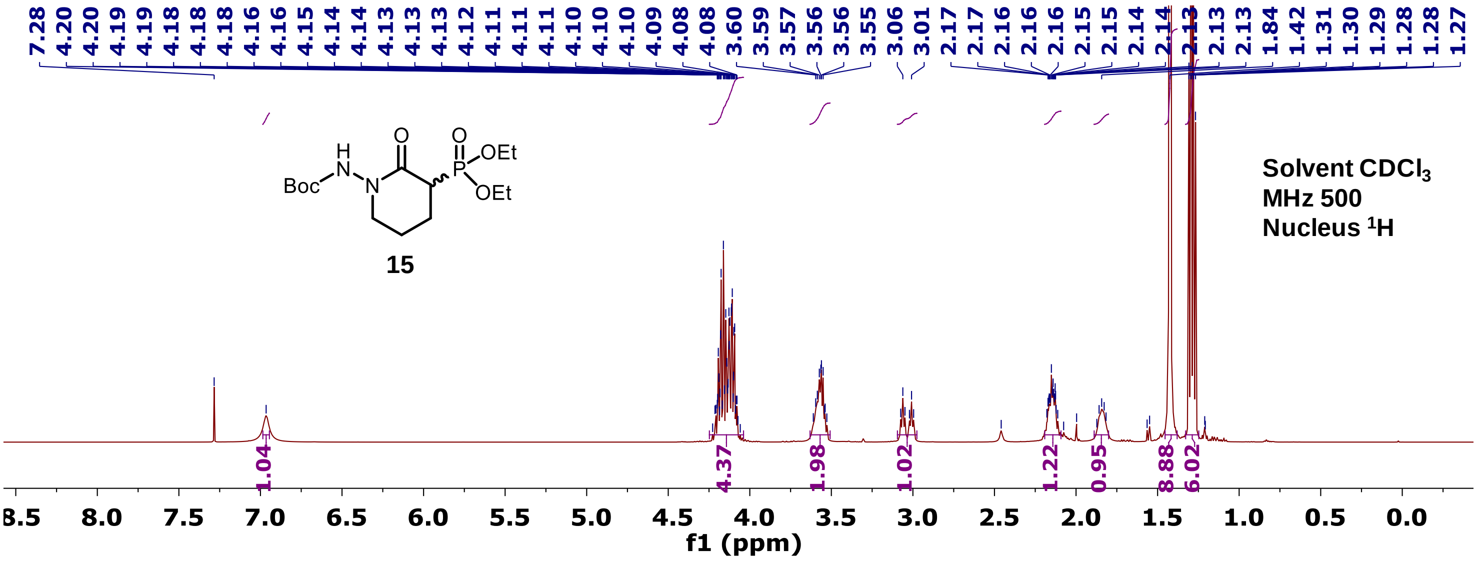
7.28 4.20 4.20 4.19 4.19 4.18 4.18 4.18 4.16 4.16 4.15 4.14 4.14 4.13 4.13 4.13 4.12 4.11 4.11 4.11 4.10 4.10 4.10 4.09 4.08 4.08 3.60 3.59 3.57 3.56 3.56 3.55 3.06 3.01 2.17 2.17 2.16 2.16 2.16 2.15 2.15 2.14 2.14 2.13 2.13 2.13 1.84 1.42 1.31 1.30 1.29 1.28 1.28 1.27
Boc
H
N
N
O
O
P
OEt
OEt
15
Solvent CDCl3
MHz 500
Nucleus 1H
1.04 4.37 1.98 1.02 1.22 0.95 8.88 6.02
8.5 8.0 7.5 7.0 6.5 6.0 5.5 5.0 4.5 4.0 3.5 3.0 2.5 2.0 1.5 1.0 0.5 0.0
f1 (ppm)

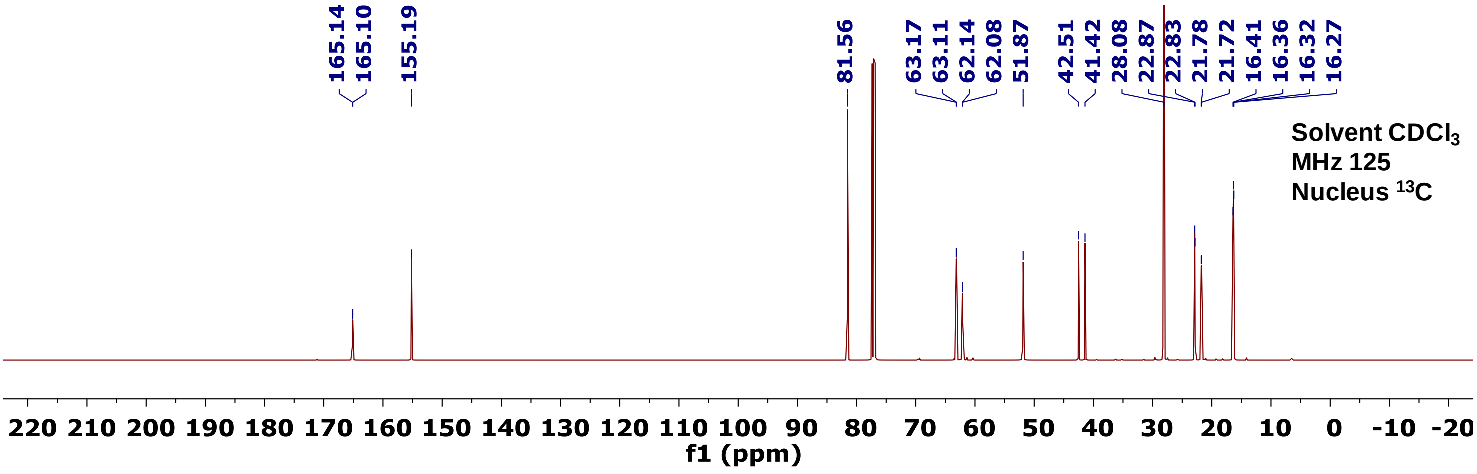
165.14 165.10 155.19 81.56 63.17 63.11 62.14 62.08 51.87 42.51 41.42 28.08 22.87 22.83 21.78 21.72 16.41 16.36 16.32 16.27
Solvent CDCl3
MHz 125
Nucleus 13C
220 210 200 190 180 170 160 150 140 130 120 110 100 90 80 70 60 50 40 30 20 10 0 -10 -20
f1 (ppm)

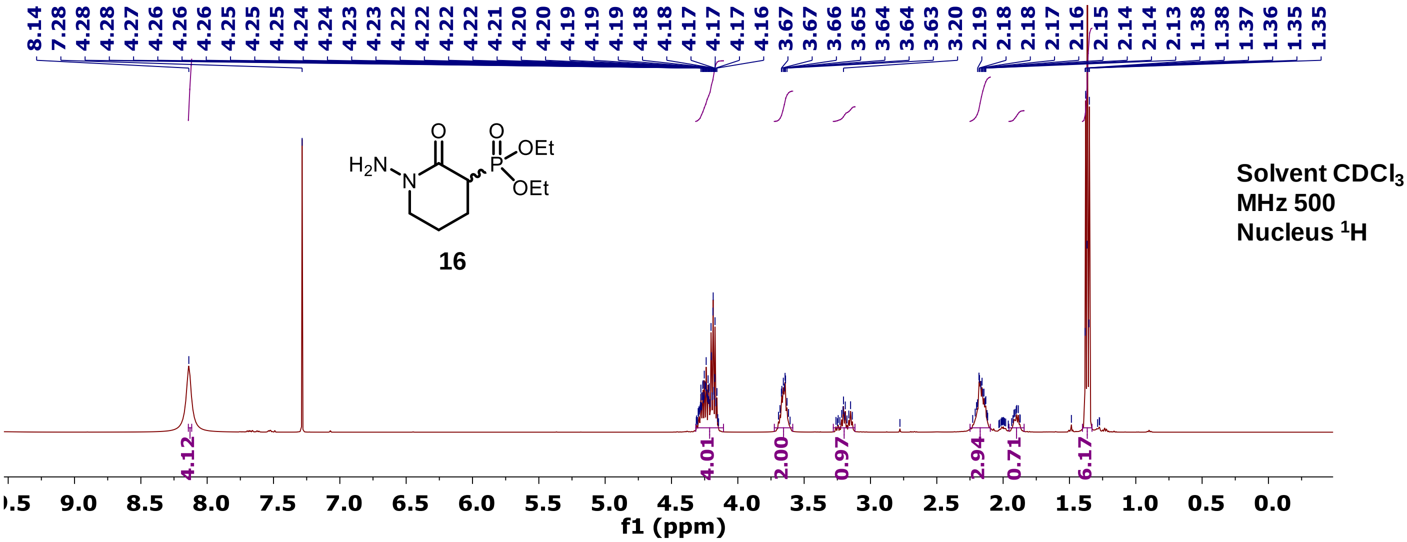
8.14 7.28 4.28 4.28 4.27 4.26 4.26 4.26 4.25 4.25 4.25 4.24 4.24 4.23 4.23 4.22 4.22 4.22 4.22 4.21 4.20 4.20 4.19 4.19 4.19 4.18 4.18 4.17 4.17 4.17 4.16 3.67 3.67 3.66 3.65 3.64 3.64 3.63 3.20 2.19 2.18 2.18 2.17 2.16 2.15 2.14 2.14 2.13 1.38 1.38 1.37 1.36 1.35 1.35
H2N
N
O
O
P
OEt
OEt
16
Solvent CDCl3
MHz 500
Nucleus 1H
4.12
4.01
2.00
0.97
2.94
0.71
6.17
9.0 8.5 8.0 7.5 7.0 6.5 6.0 5.5 5.0 4.5 4.0 3.5 3.0 2.5 2.0 1.5 1.0 0.5 0.0
f1 (ppm)

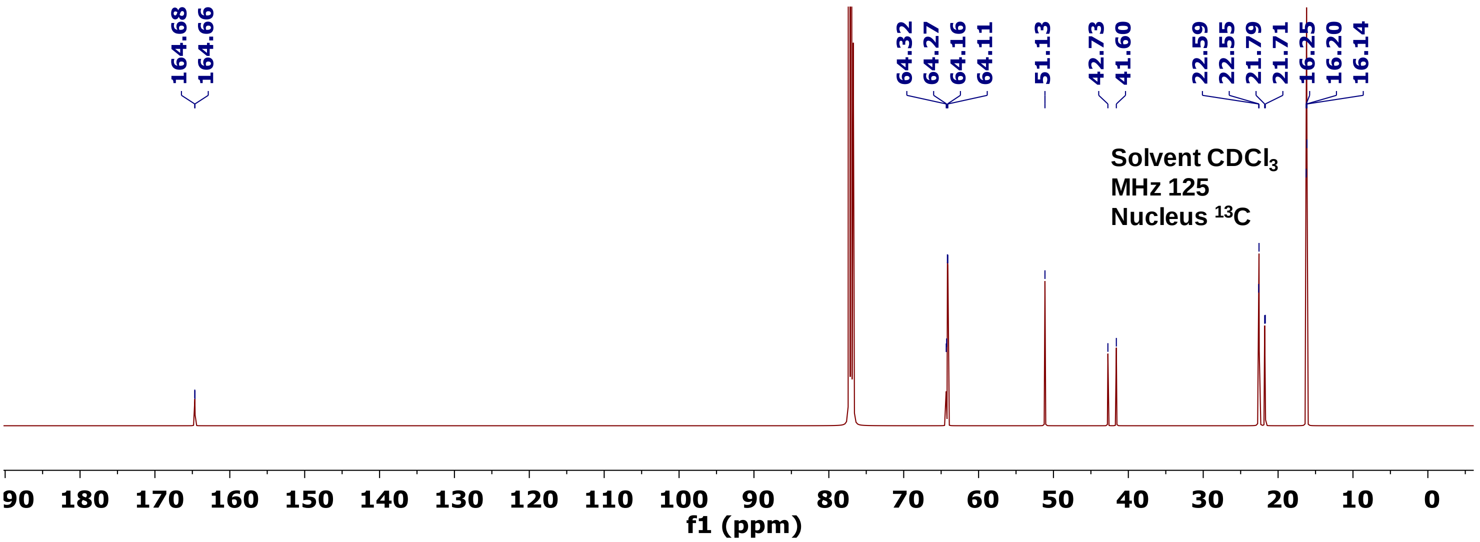
164.68 164.66
64.32 64.27 64.16 64.11
51.13
42.73 41.60
22.59 22.55 21.79 21.71 16.25 16.20 16.14
Solvent CDCl3
MHz 125
Nucleus 13C
90 180 170 160 150 140 130 120 110 100 90 80 70 60 50 40 30 20 10 0
f1 (ppm)

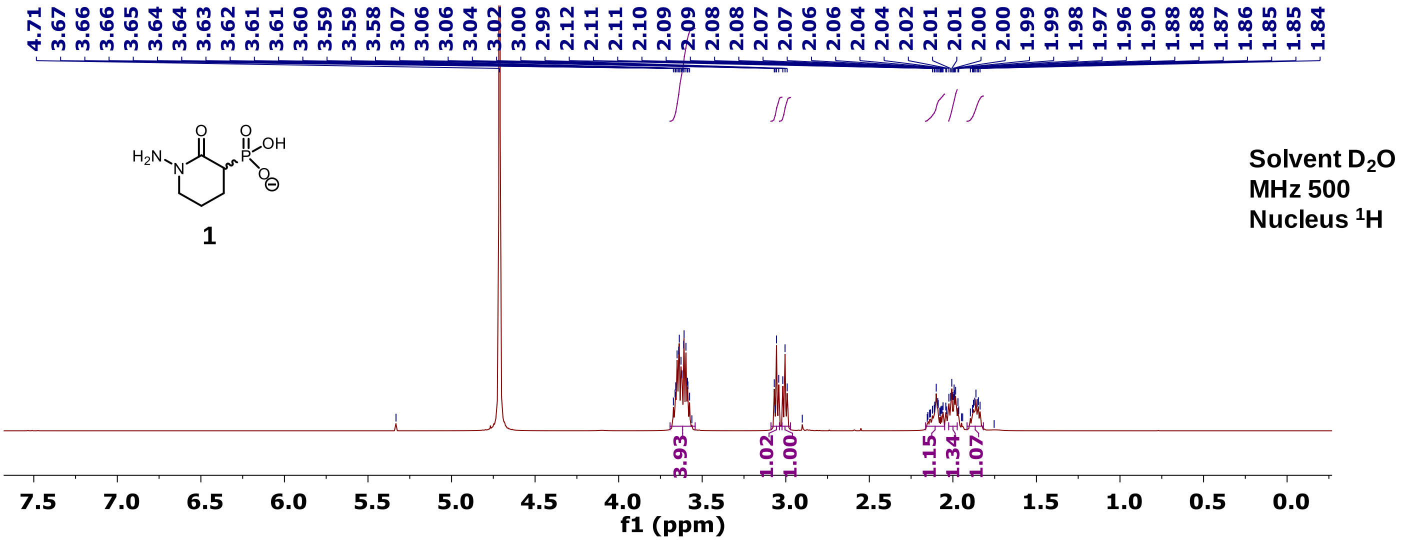
4.71 3.67 3.66 3.66 3.65 3.64 3.64 3.63 3.62 3.61 3.61 3.60 3.59 3.59 3.58 3.07 3.06 3.06 3.04 3.02 3.00 2.99 2.12 2.11 2.11 2.10 2.09 2.09 2.08 2.08 2.07 2.07 2.06 2.06 2.04 2.04 2.02 2.01 2.01 2.00 2.00 1.99 1.99 1.98 1.97 1.96 1.90 1.88 1.88 1.87 1.86 1.85 1.85 1.84
1
Solvent D_2O
MHz 500
Nucleus ^1H
3.93
1.02
1.00
1.15
1.34
1.07
f1 (ppm)

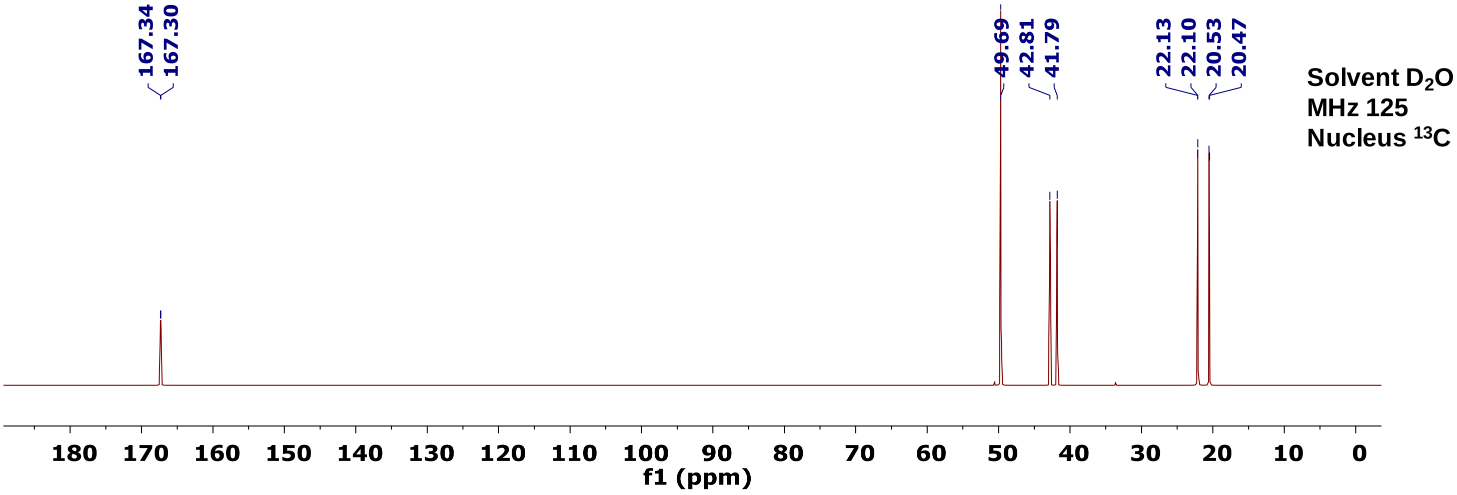
167.34
167.30
49.69
42.81
41.79
22.13
22.10
20.53
20.47
Solvent D_2O
MHz 125
Nucleus ^{13}C
f1 (ppm)

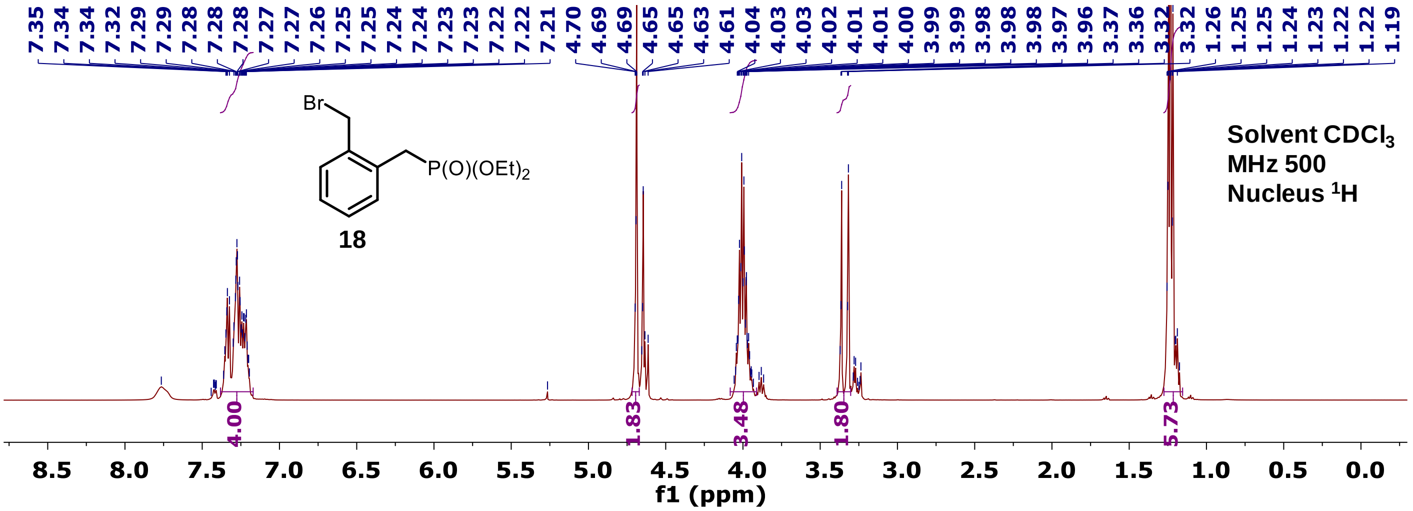
Br
P(O)(OEt)2
18
Solvent CDCl3
MHz 500
Nucleus 1H
f1 (ppm)

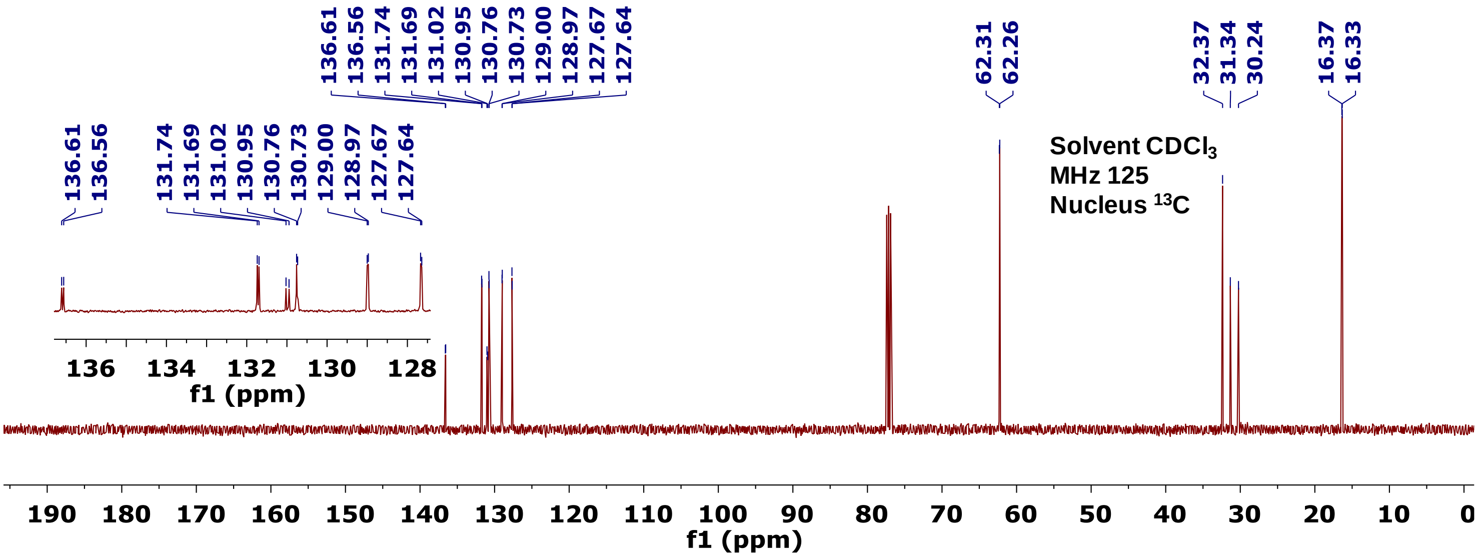
Solvent CDCl3
MHz 125
Nucleus 13C
f1 (ppm)

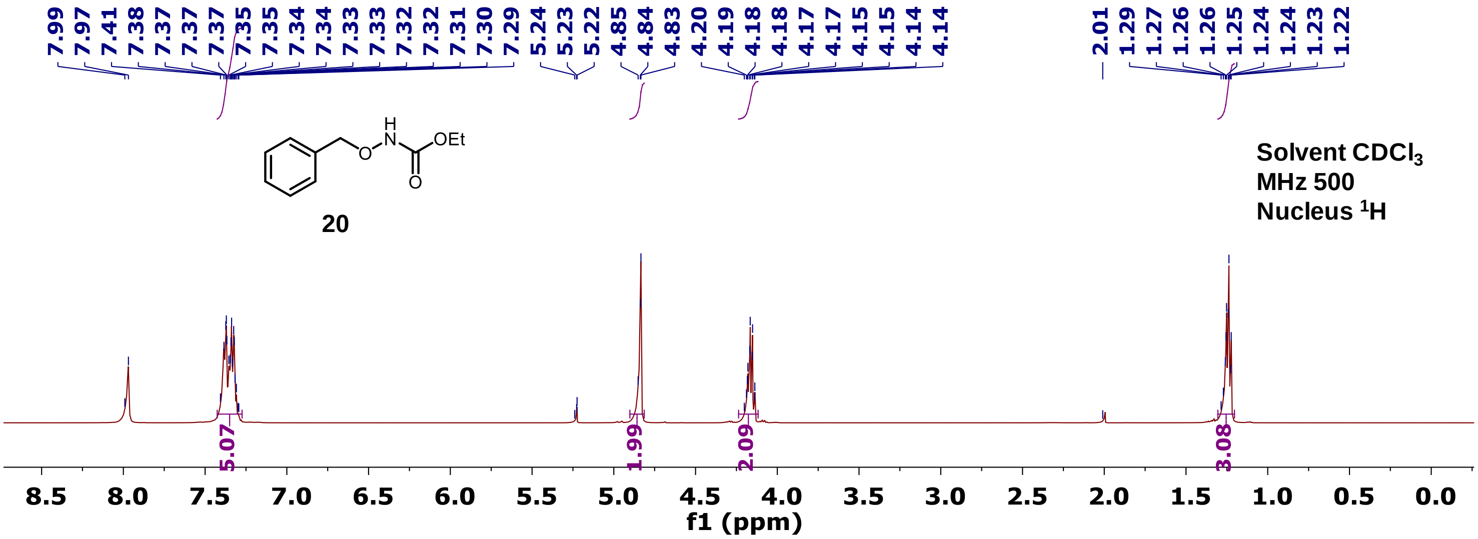
7.99 7.97 7.41 7.38 7.37 7.37 7.37 7.35 7.35 7.34 7.34 7.33 7.33 7.32 7.32 7.31 7.30 7.29 5.24 5.23 5.22 4.85 4.84 4.83 4.20 4.19 4.18 4.18 4.17 4.17 4.15 4.15 4.14 4.14 2.01 1.29 1.27 1.26 1.26 1.25 1.24 1.24 1.23 1.22
OEt
20
Solvent CDCl3
MHz 500
Nucleus 1H
5.07
1.99
2.09
3.08
8.5 8.0 7.5 7.0 6.5 6.0 5.5 5.0 4.5 4.0 3.5 3.0 2.5 2.0 1.5 1.0 0.5 0.0
f1 (ppm)

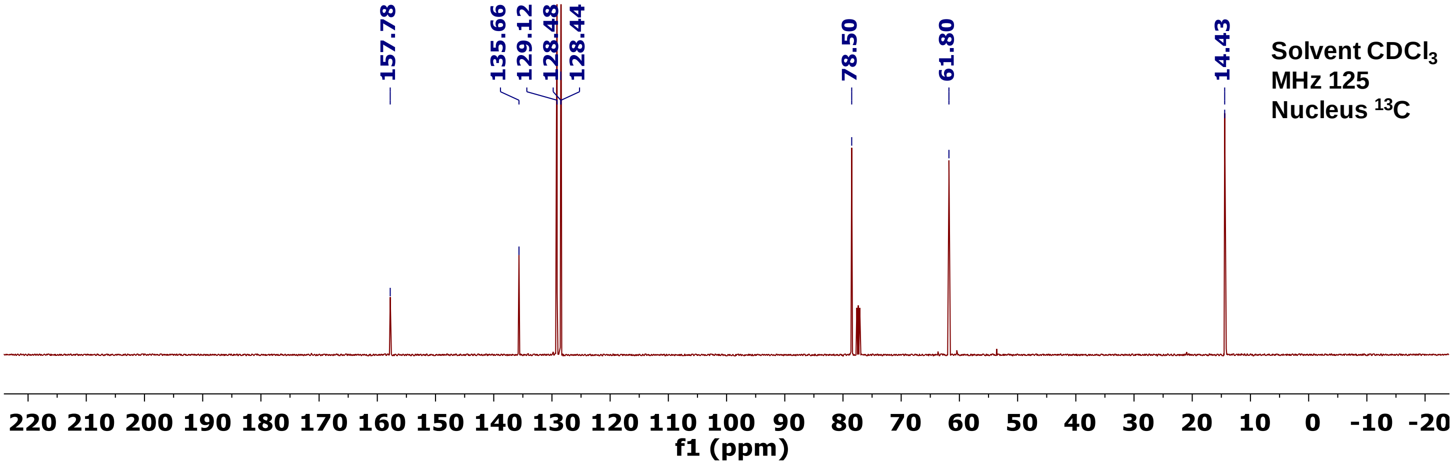
157.78
135.66 129.12 128.48 128.44
78.50
61.80
14.43
Solvent CDCl3
MHz 125
Nucleus 13C
220 210 200 190 180 170 160 150 140 130 120 110 100 90 80 70 60 50 40 30 20 10 0 -10 -20
f1 (ppm)

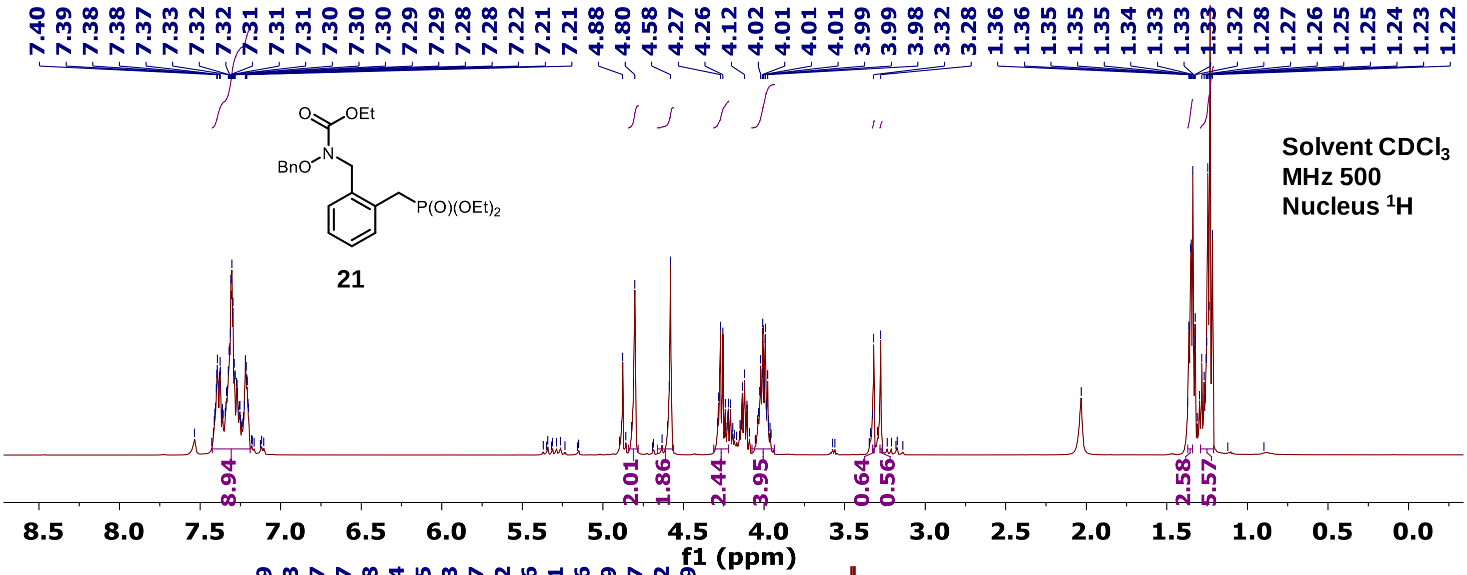
7.40 7.39 7.38 7.38 7.37 7.33 7.32 7.32 7.31 7.31 7.31 7.30 7.30 7.30 7.29 7.29 7.28 7.28 7.22 7.21 7.21 4.88 4.80 4.58 4.27 4.26 4.12 4.02 4.01 4.01 3.99 3.99 3.98 3.32 3.28 1.36 1.36 1.35 1.35 1.35 1.34 1.33 1.33 1.33 1.32 1.28 1.27 1.26 1.25 1.25 1.24 1.23 1.22
OEt
O
N
BnO
P(O)(OEt)2
21
Solvent CDCl3
MHz 500
Nucleus 1H
8.94
2.01
1.86
2.44
3.95
0.64
0.56
2.58
5.57
8.5 8.0 7.5 7.0 6.5 6.0 5.5 5.0 4.5 4.0 3.5 3.0 2.5 2.0 1.5 1.0 0.5 0.0
f1 (ppm)

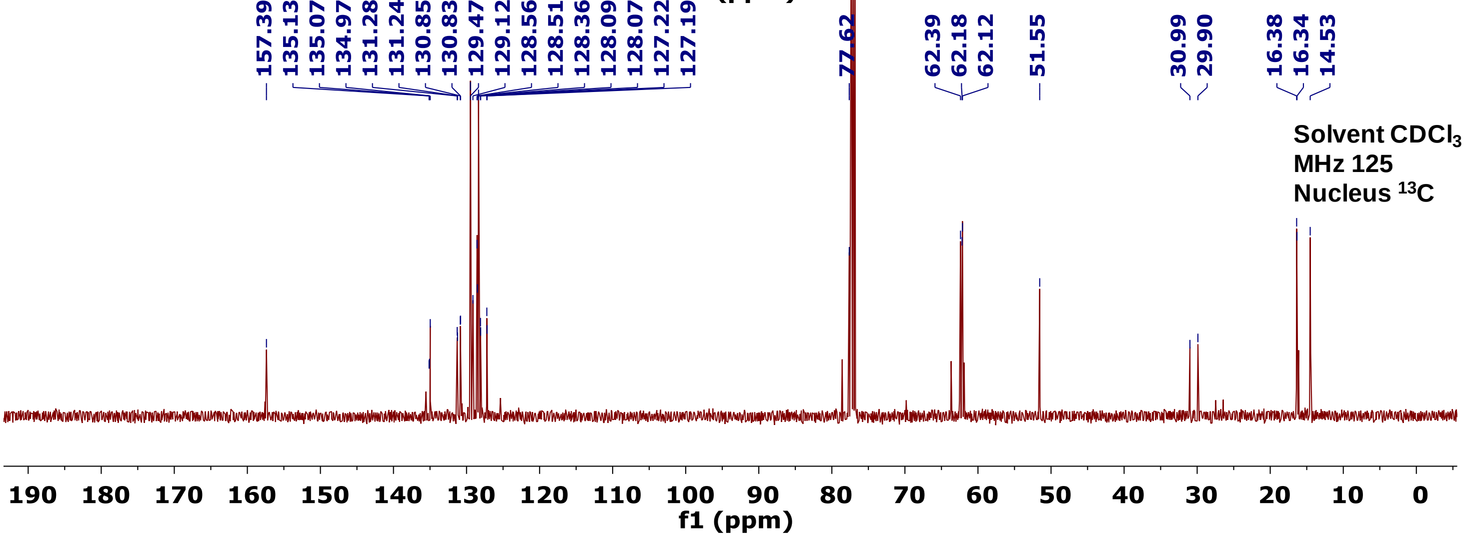
157.39 135.13 135.07 134.97 131.28 131.24 130.85 130.83 129.47 129.12 128.56 128.51 128.36 128.09 128.07 127.22 127.19
77.62
62.39 62.18 62.12
51.55
30.99 29.90
16.38 16.34 14.53
Solvent CDCl3
MHz 125
Nucleus 13C
190 180 170 160 150 140 130 120 110 100 90 80 70 60 50 40 30 20 10 0
f1 (ppm)

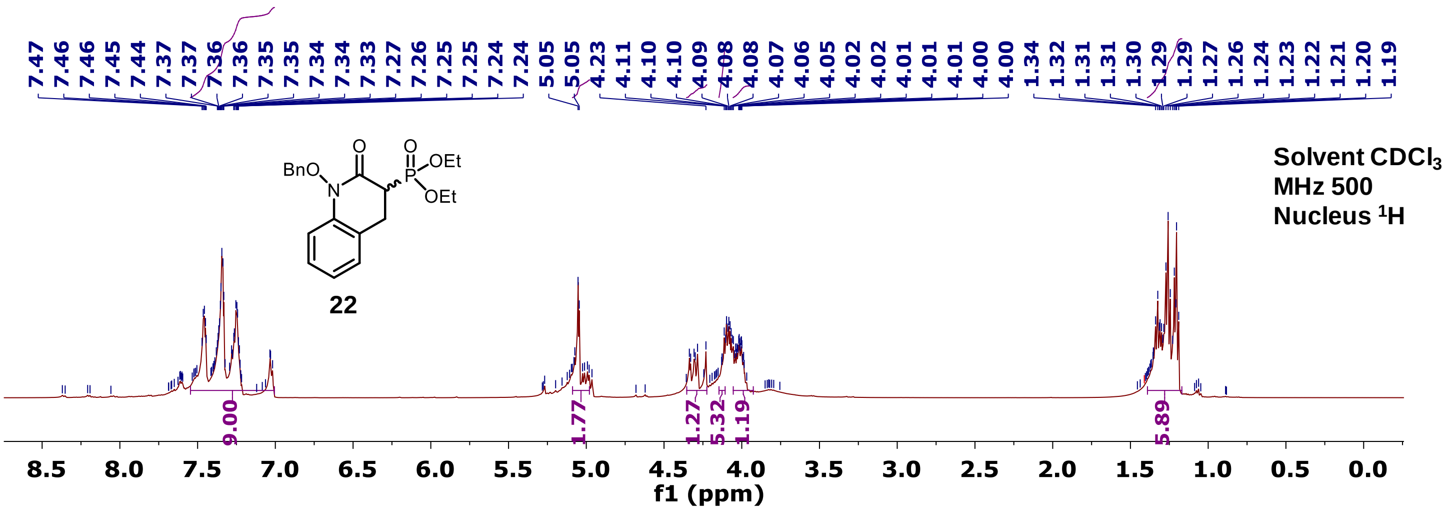
BnO
N
O
O
P
OEt
OEt
22
Solvent CDCl3
MHz 500
Nucleus 1H
f1 (ppm)

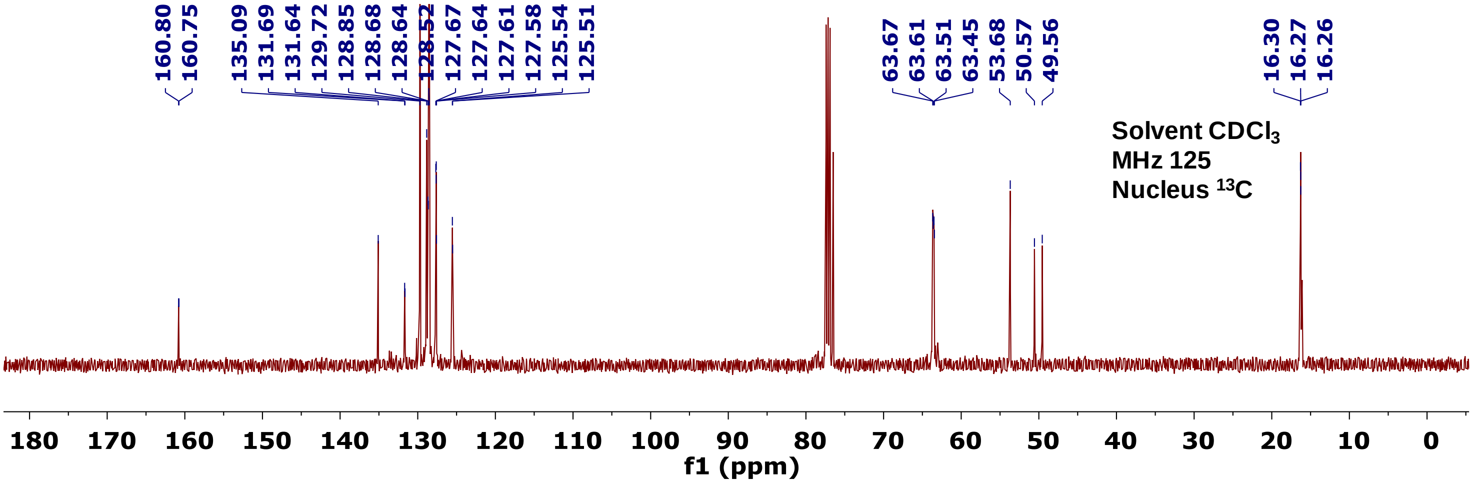
Solvent CDCl3
MHz 125
Nucleus 13C
f1 (ppm)

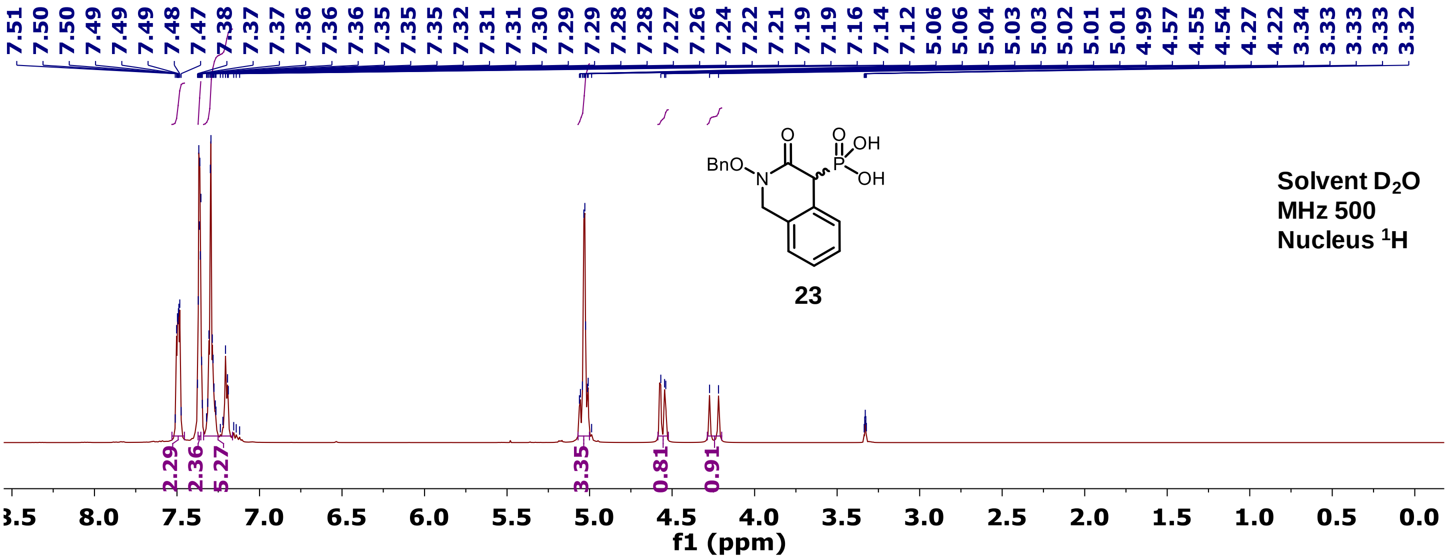
7.51 7.50 7.50 7.49 7.49 7.49 7.48 7.47 7.38 7.37 7.37 7.36 7.36 7.36 7.35 7.35 7.35 7.32 7.31 7.31 7.30 7.29 7.29 7.28 7.28 7.27 7.26 7.24 7.22 7.21 7.19 7.19 7.16 7.14 7.12 5.06 5.06 5.04 5.03 5.03 5.02 5.01 5.01 4.99 4.57 4.55 4.54 4.27 4.22 3.34 3.33 3.33 3.33 3.32
BnO
N
O
P
OH
OH
23
Solvent D_2O
MHz 500
Nucleus ^1H
2.29
2.36
5.27
3.35
0.81
0.91
f1 (ppm)

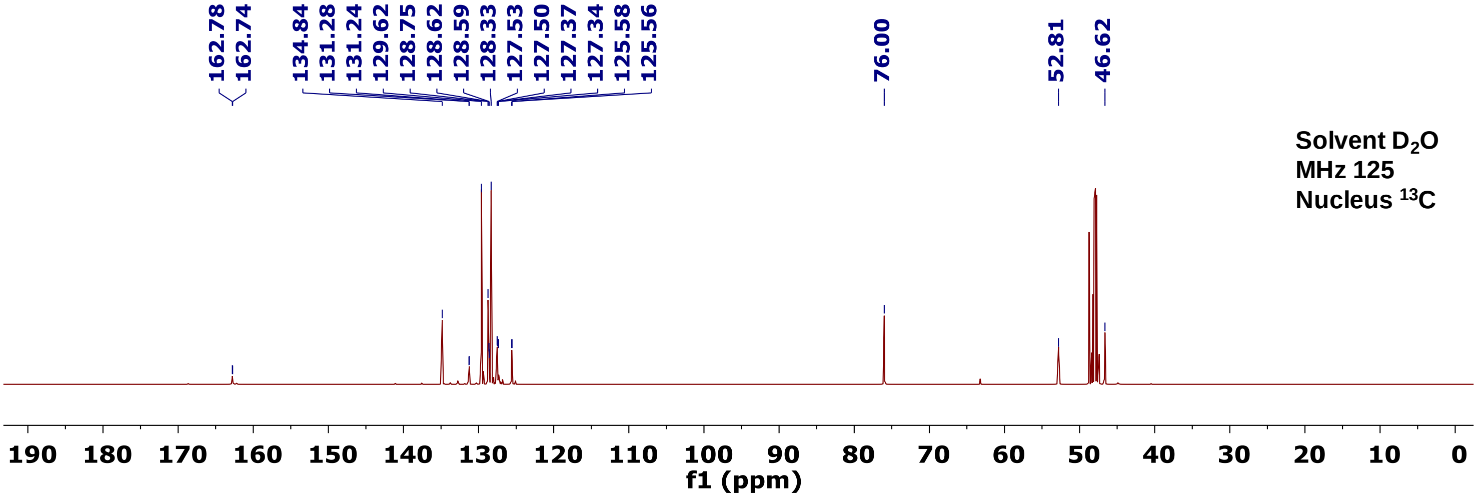
162.78 162.74 134.84 131.28 131.24 129.62 128.75 128.62 128.59 128.33 127.53 127.50 127.37 127.34 125.58 125.56
76.00
52.81
46.62
Solvent D_2O
MHz 125
Nucleus ^{13}C
f1 (ppm)

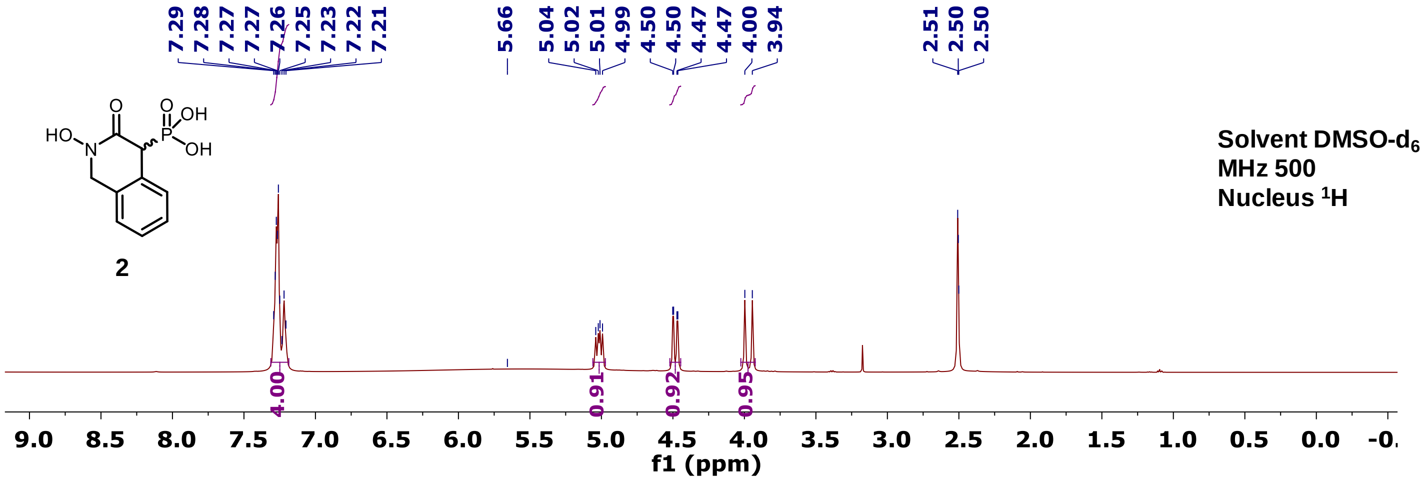
HO
N
O
P
OH
OH
2
7.29
7.28
7.27
7.27
7.26
7.25
7.23
7.22
7.21
5.66
5.04
5.02
5.01
4.99
4.50
4.50
4.47
4.47
4.00
3.94
2.51
2.50
2.50
Solvent DMSO-d$_6$
MHz 500
Nucleus ^{1}H
4.00
0.91
0.92
0.95
9.0 8.5 8.0 7.5 7.0 6.5 6.0 5.5 5.0 4.5 4.0 3.5 3.0 2.5 2.0 1.5 1.0 0.5 0.0
f1 (ppm)

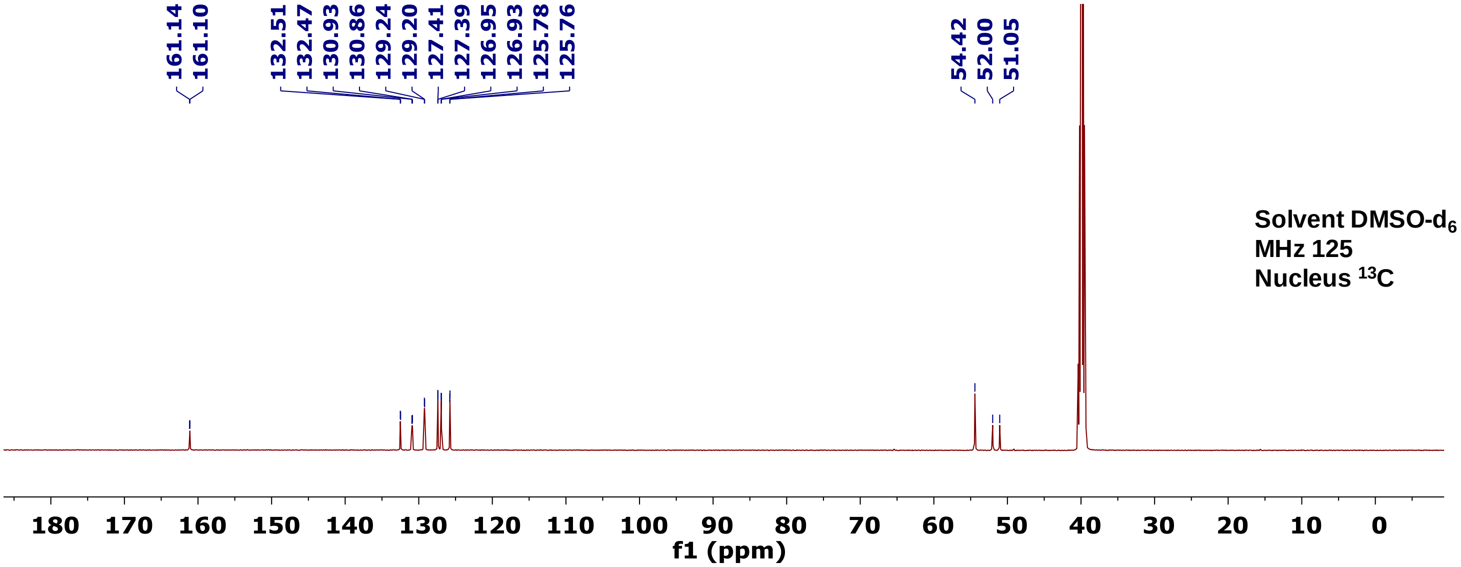
161.14
161.10
132.51
132.47
130.93
130.86
129.24
129.20
127.41
127.39
126.95
126.93
125.78
125.76
54.42
52.00
51.05
Solvent DMSO-d$_6$
MHz 125
Nucleus ^{13}C
180 170 160 150 140 130 120 110 100 90 80 70 60 50 40 30 20 10 0
f1 (ppm)

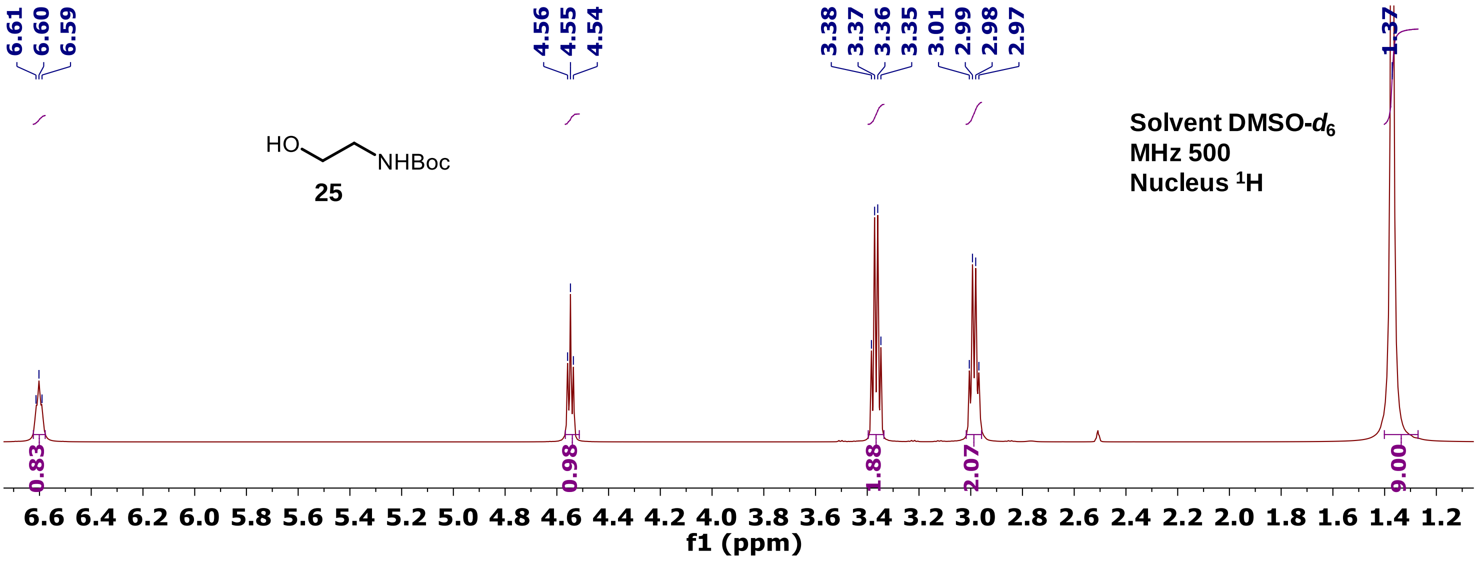
6.61
6.60
6.59
4.56
4.55
4.54
3.38
3.37
3.36
3.35
3.01
2.99
2.98
2.97
1.37
HO
NHBoc
25
Solvent DMSO-d_6
MHz 500
Nucleus ^{1}H
0.83
0.98
1.88
2.07
9.00
6.6 6.4 6.2 6.0 5.8 5.6 5.4 5.2 5.0 4.8 4.6 4.4 4.2 4.0 3.8 3.6 3.4 3.2 3.0 2.8 2.6 2.4 2.2 2.0 1.8 1.6 1.4 1.2
f1 (ppm)

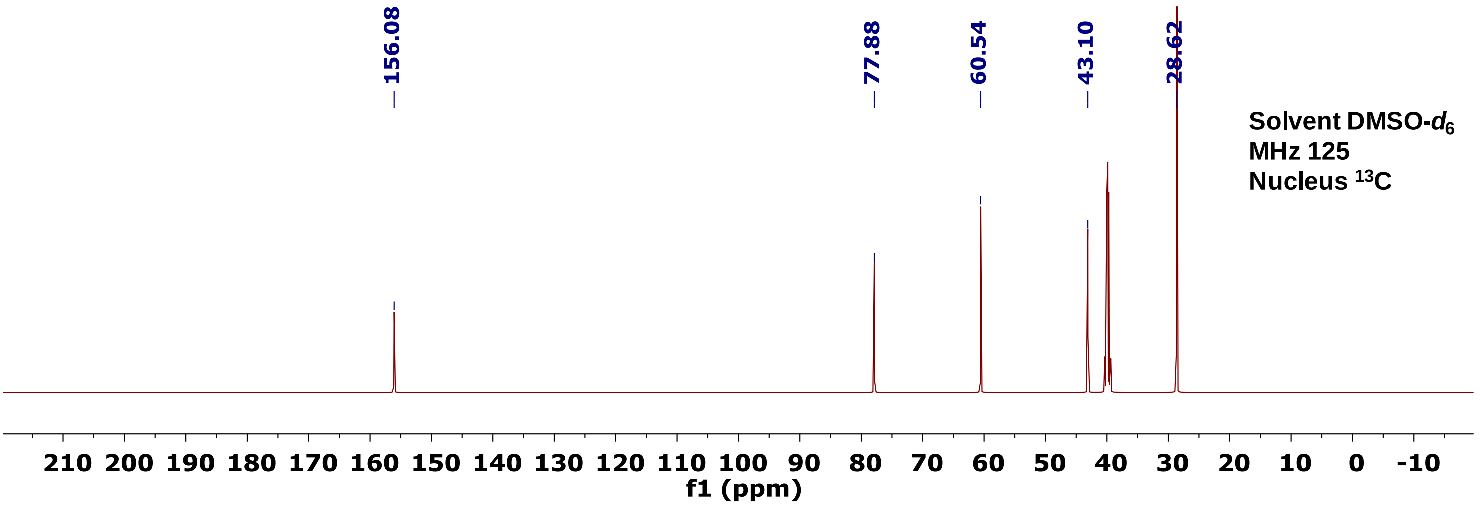
156.08
77.88
60.54
43.10
28.62
Solvent DMSO-d_6
MHz 125
Nucleus ^{13}C
210 200 190 180 170 160 150 140 130 120 110 100 90 80 70 60 50 40 30 20 10 0 -10
f1 (ppm)

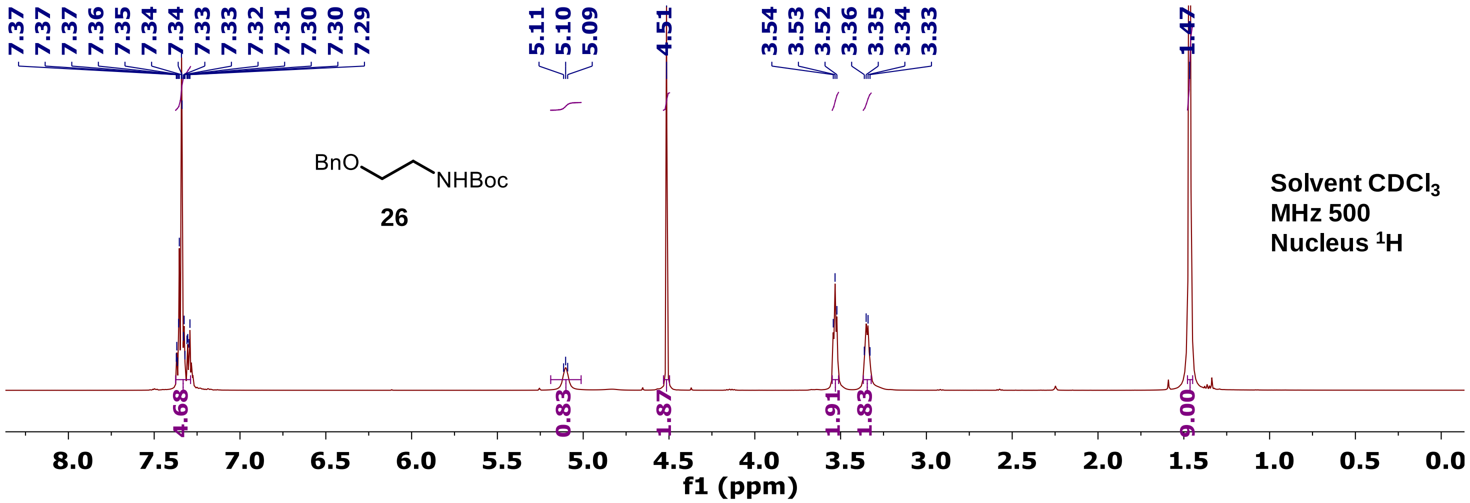
BnO NHBoc
26
Solvent $CDCl_3$
MHz 500
Nucleus ^1H
7.37 7.37 7.37 7.36 7.35 7.34 7.34 7.33 7.33 7.32 7.31 7.30 7.30 7.29
5.11 5.10 5.09
4.51
3.54 3.53 3.52 3.36 3.35 3.34 3.33
1.47
4.68 0.83 1.87 1.91 1.83 9.00
8.0 7.5 7.0 6.5 6.0 5.5 5.0 4.5 4.0 3.5 3.0 2.5 2.0 1.5 1.0 0.5 0.0
f1 (ppm)

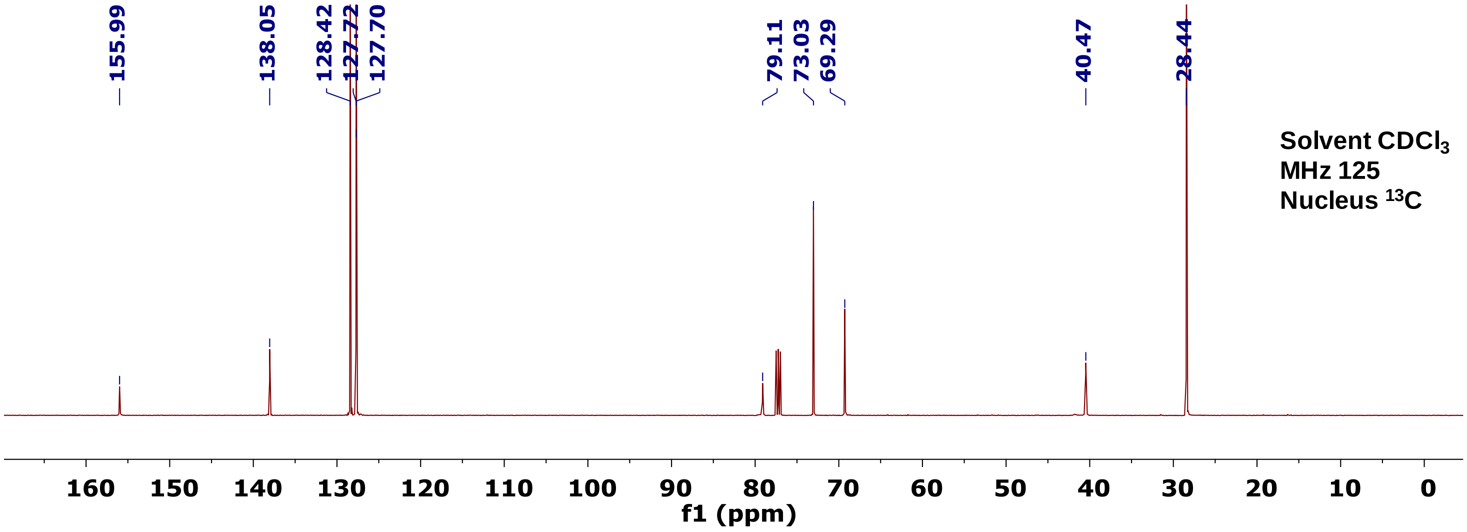
155.99
138.05
128.42 127.72 127.70
79.11 73.03 69.29
40.47
28.44
Solvent $CDCl_3$
MHz 125
Nucleus ^{13}C
160 150 140 130 120 110 100 90 80 70 60 50 40 30 20 10 0
f1 (ppm)

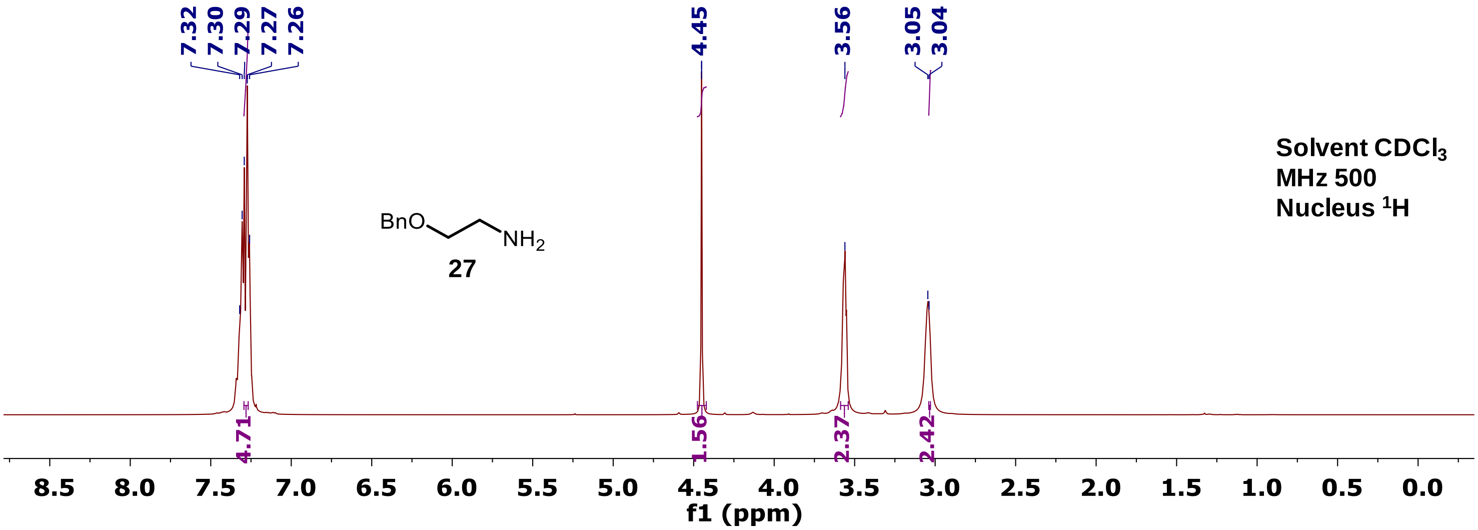
7.32
7.30
7.29
7.27
7.26
4.45
3.56
3.05
3.04
BnO
NH2
27
Solvent CDCl3
MHz 500
Nucleus 1H
4.71
1.56
2.37
2.42
8.5 8.0 7.5 7.0 6.5 6.0 5.5 5.0 4.5 4.0 3.5 3.0 2.5 2.0 1.5 1.0 0.5 0.0
f1 (ppm)

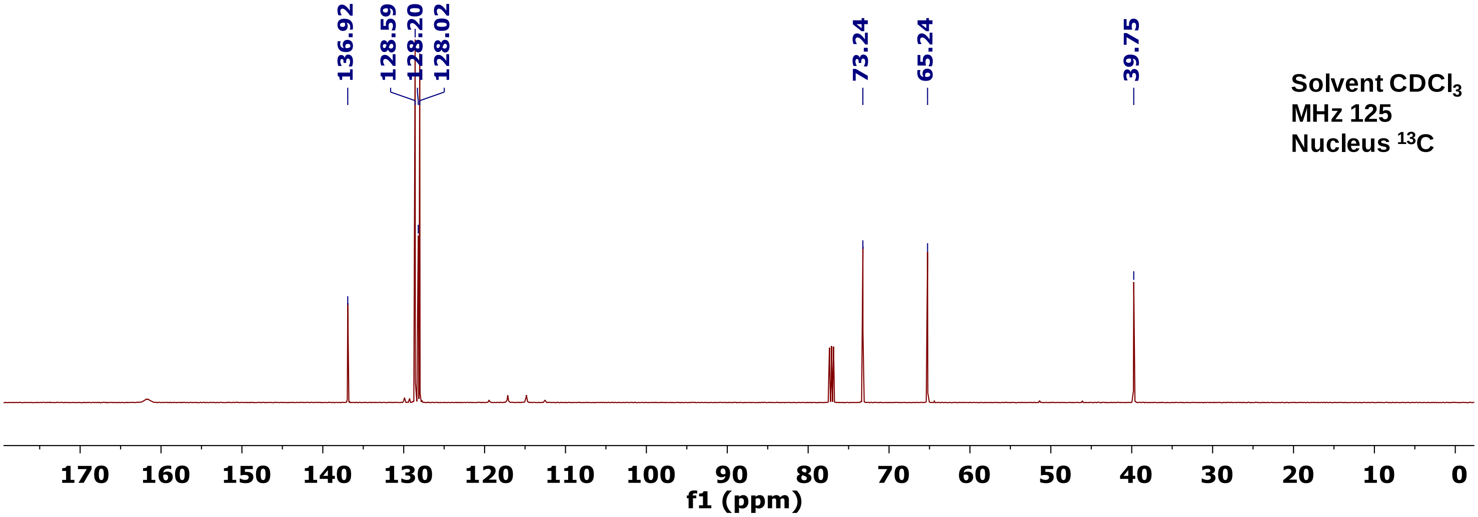
136.92
128.59
128.20
128.02
73.24
65.24
39.75
Solvent CDCl3
MHz 125
Nucleus 13C
170 160 150 140 130 120 110 100 90 80 70 60 50 40 30 20 10 0
f1 (ppm)

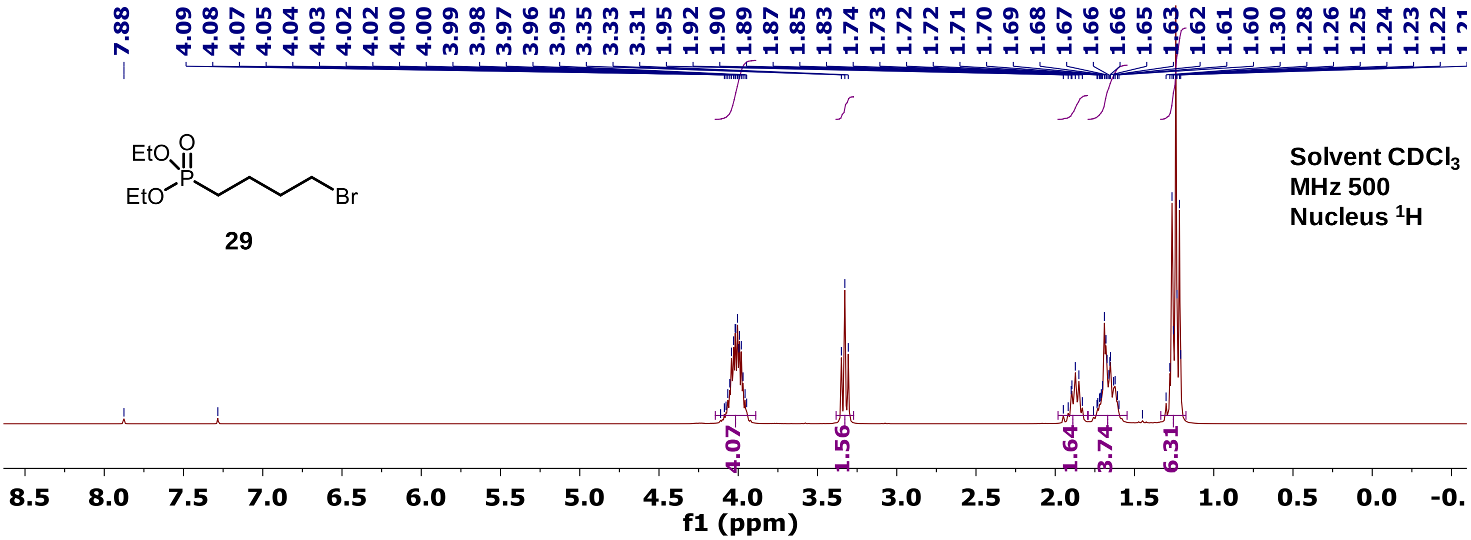
EtO
EtO
O
P
Br
29
Solvent CDCl3
MHz 500
Nucleus 1H
4.07
1.56
1.64
3.74
6.31
f1 (ppm)

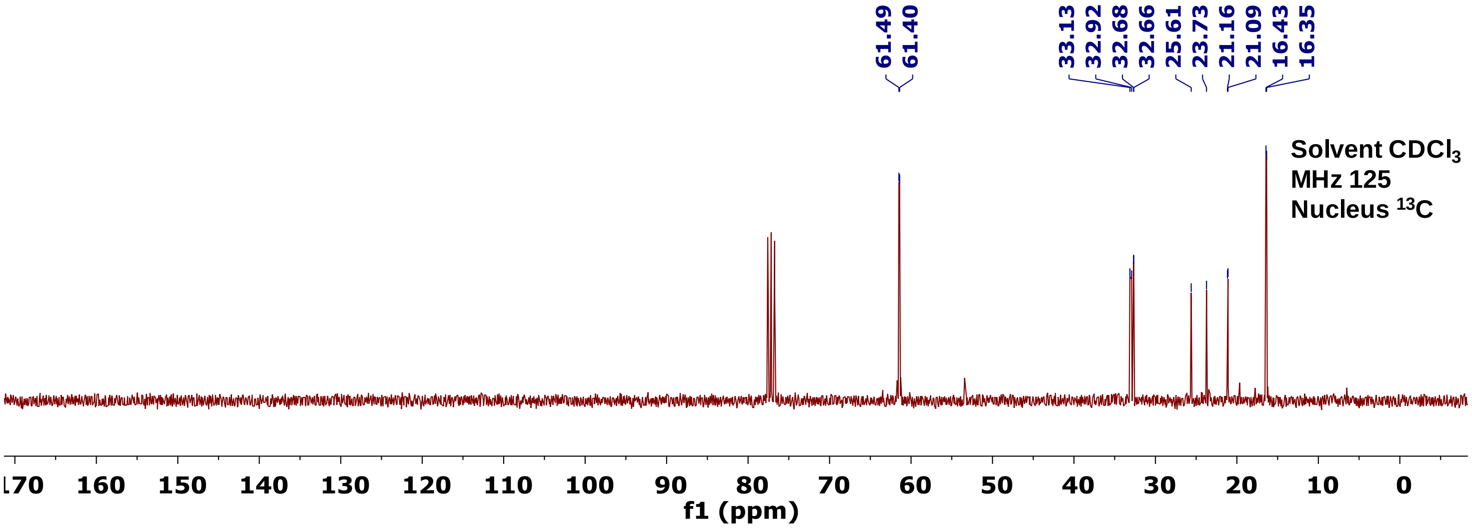
Solvent CDCl3
MHz 125
Nucleus 13C
f1 (ppm)

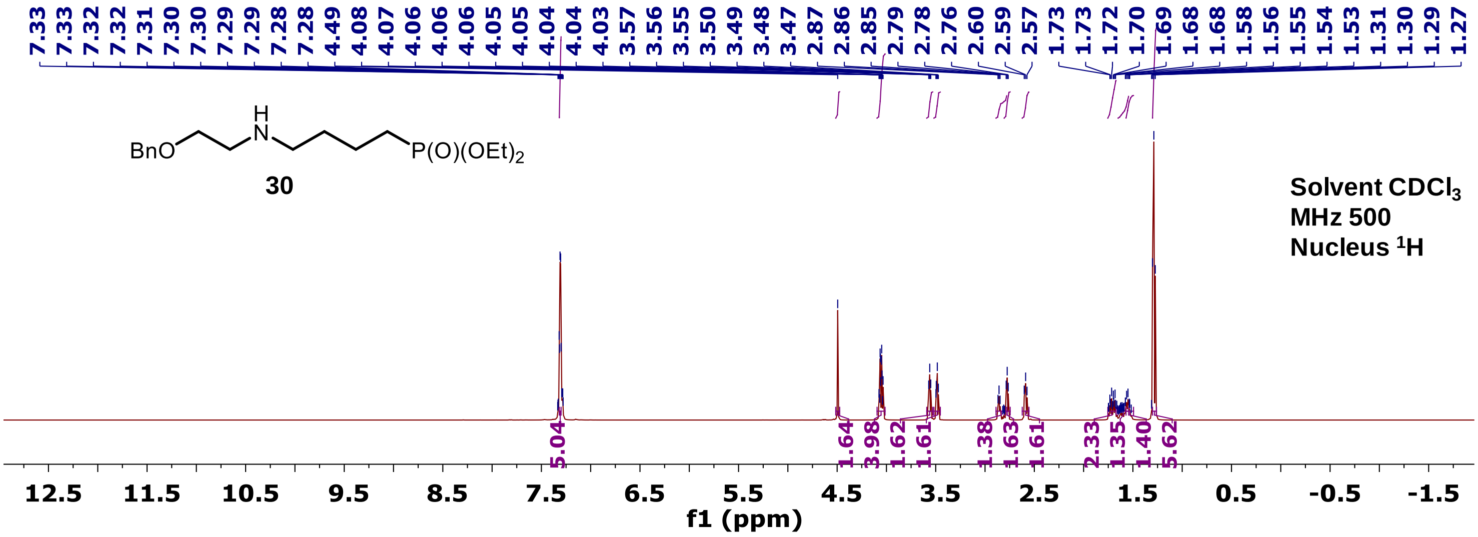
7.33 7.33 7.32 7.32 7.31 7.30 7.30 7.29 7.29 7.28 7.28 4.49 4.08 4.07 4.06 4.06 4.06 4.05 4.05 4.04 4.04 4.03 3.57 3.56 3.55 3.50 3.49 3.48 3.47 2.87 2.86 2.85 2.79 2.78 2.76 2.60 2.59 2.57 1.73 1.73 1.72 1.70 1.69 1.68 1.68 1.58 1.56 1.55 1.54 1.53 1.31 1.30 1.29 1.27
BnO
H
N
P(O)(OEt)2
30
Solvent CDCl3
MHz 500
Nucleus 1H
5.04 1.64 3.98 1.62 1.61 1.38 1.63 1.61 2.33 1.35 1.40 5.62
12.5 11.5 10.5 9.5 8.5 7.5 6.5 5.5 4.5 3.5 2.5 1.5 0.5 -0.5 -1.5
f1 (ppm)

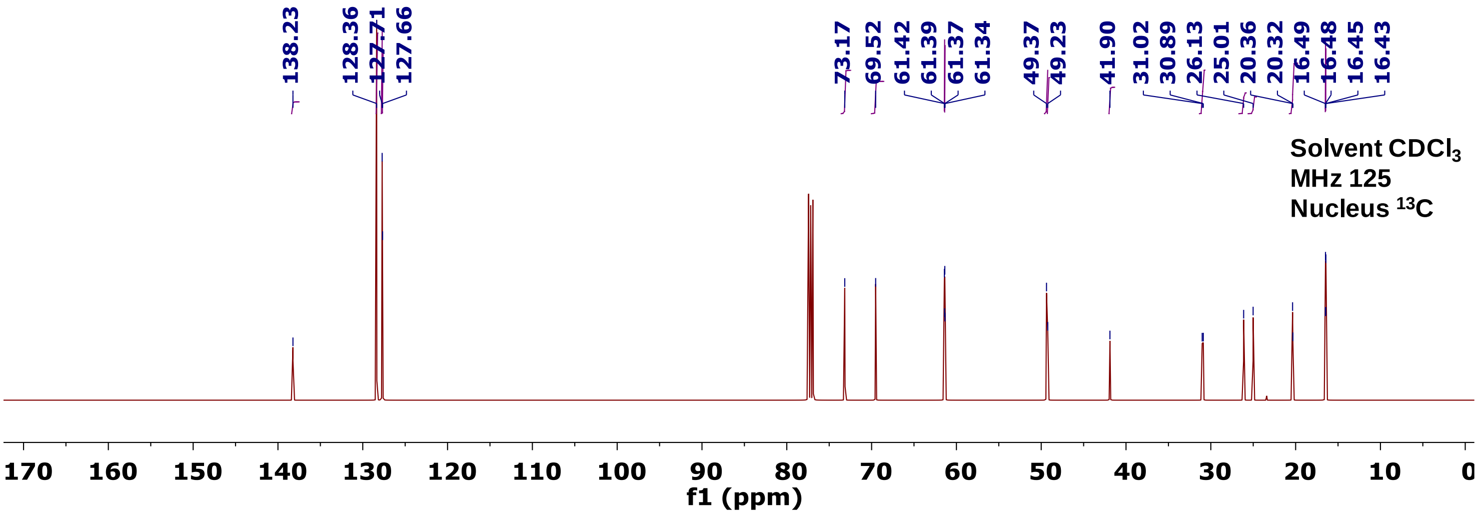
138.23 128.36 127.71 127.66 73.17 69.52 61.42 61.39 61.37 61.34 49.37 49.23 41.90 31.02 30.89 26.13 25.01 20.36 20.32 16.49 16.48 16.45 16.43
Solvent CDCl3
MHz 125
Nucleus 13C
170 160 150 140 130 120 110 100 90 80 70 60 50 40 30 20 10 0
f1 (ppm)

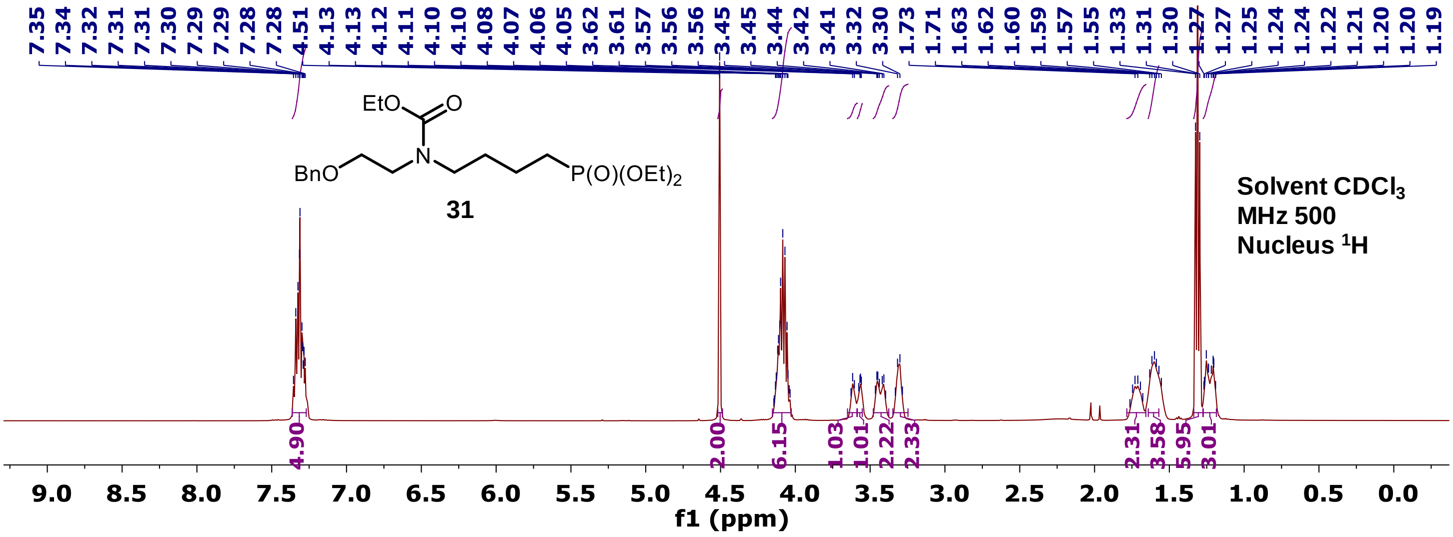
31
Solvent CDCl3
MHz 500
Nucleus 1H
f1 (ppm)

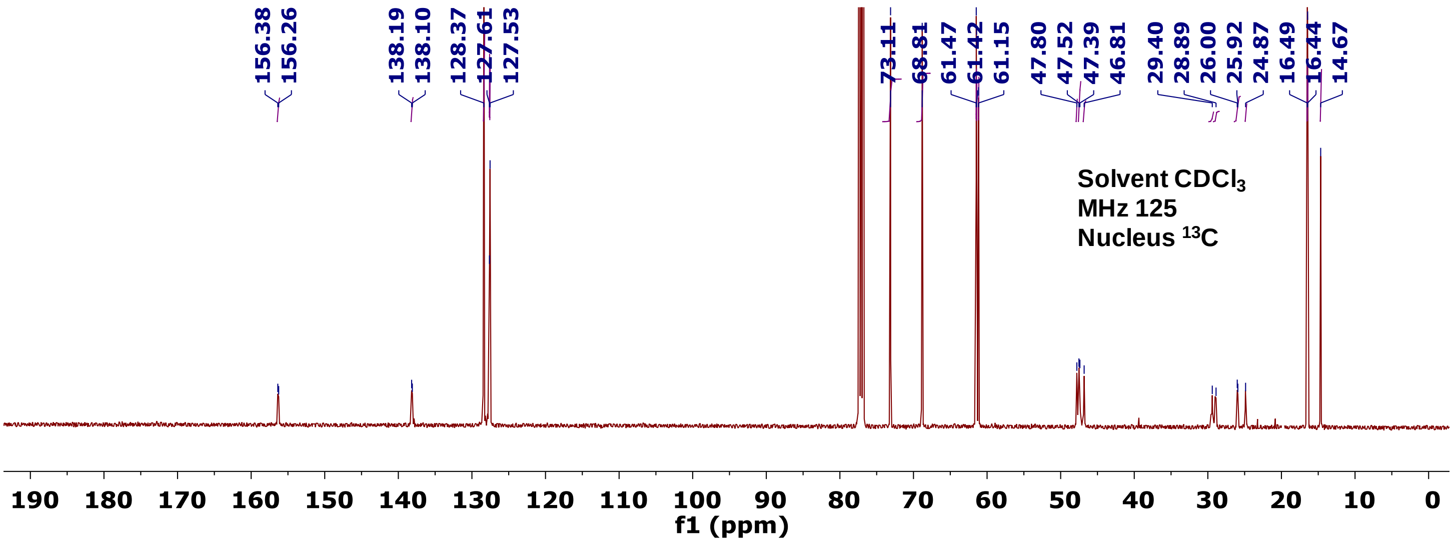
Solvent CDCl3
MHz 125
Nucleus 13C
f1 (ppm)

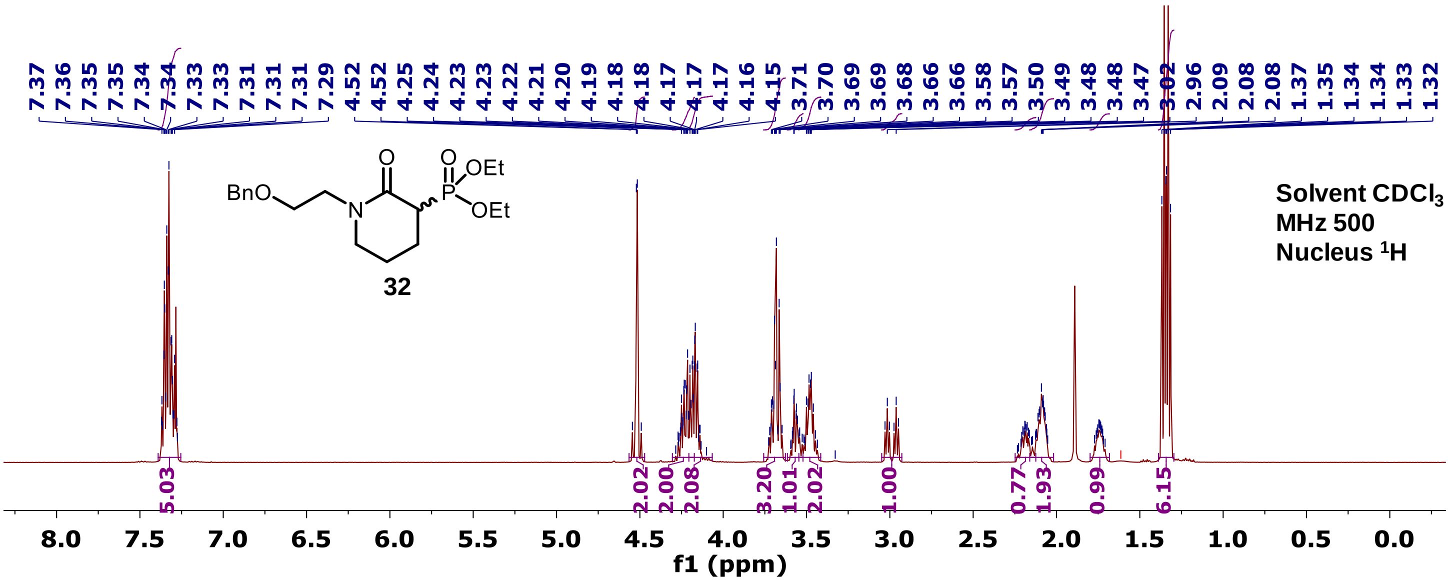
7.37 7.36 7.35 7.35 7.34 7.34 7.33 7.33 7.31 7.31 7.31 7.29 4.52 4.52 4.25 4.24 4.23 4.23 4.22 4.21 4.20 4.19 4.18 4.18 4.17 4.17 4.17 4.16 4.15 3.71 3.70 3.69 3.69 3.68 3.66 3.66 3.58 3.57 3.50 3.49 3.48 3.48 3.47 3.02 2.96 2.09 2.08 1.37 1.35 1.34 1.34 1.33 1.32
BnO
N
O
O
P
OEt
OEt
32
Solvent $CDCl_3$
MHz 500
Nucleus ^1H
5.03 2.02 2.00 2.08 3.20 1.01 2.02 1.00 0.77 1.93 0.99 6.15
8.0 7.5 7.0 6.5 6.0 5.5 5.0 4.5 4.0 3.5 3.0 2.5 2.0 1.5 1.0 0.5 0.0
f1 (ppm)

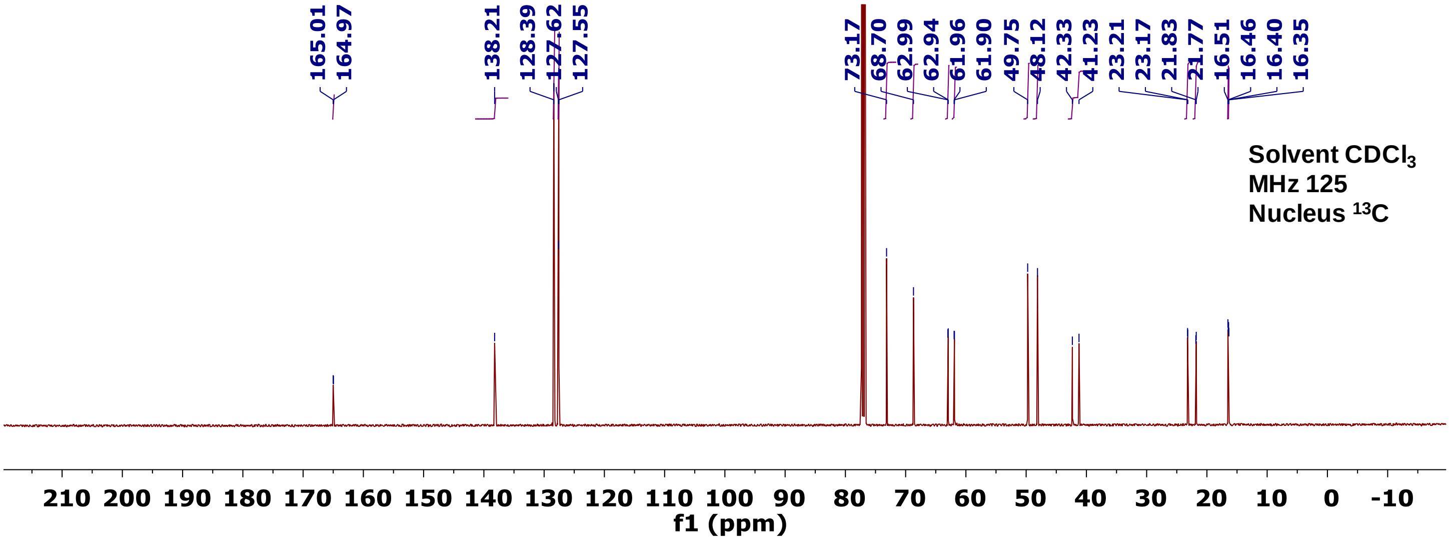
165.01 164.97 138.21 128.39 127.62 127.55 73.17 68.70 62.99 62.94 61.96 61.90 49.75 48.12 42.33 41.23 23.21 23.17 21.83 21.77 16.51 16.46 16.40 16.35
Solvent $CDCl_3$
MHz 125
Nucleus ^{13}C
210 200 190 180 170 160 150 140 130 120 110 100 90 80 70 60 50 40 30 20 10 0 -10
f1 (ppm)

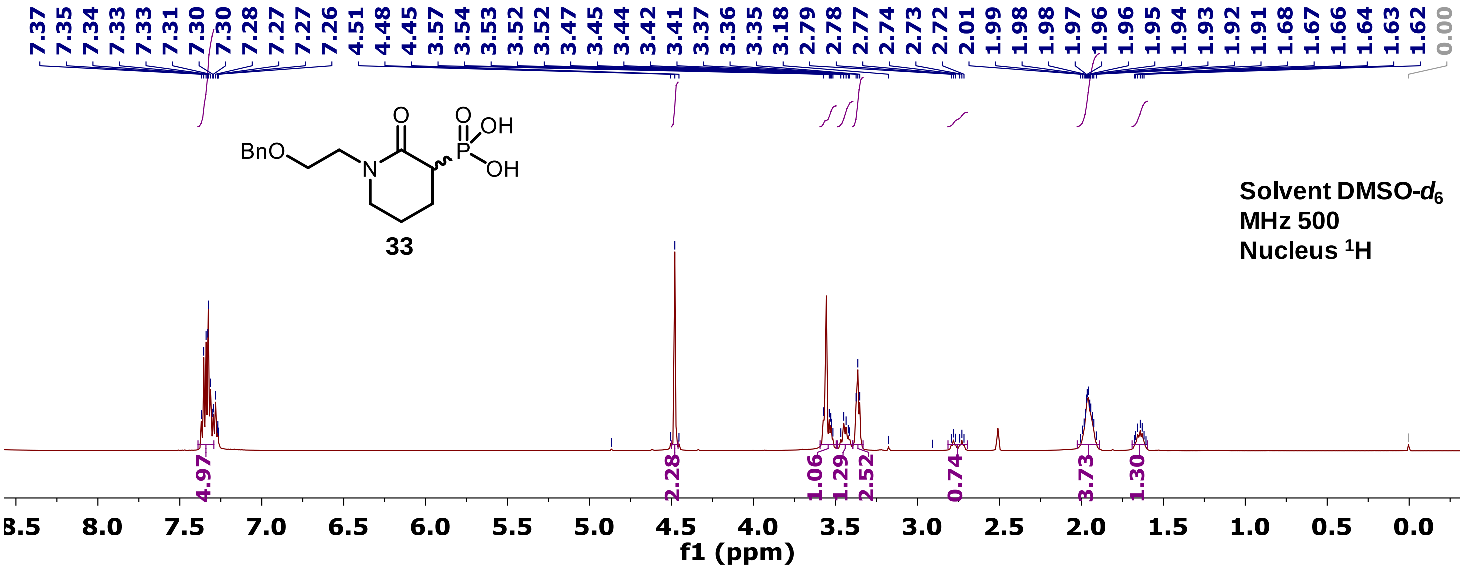
BnO
N
O
P
OH
OH
33
Solvent DMSO-d_6
MHz 500
Nucleus ^{1}H
f1 (ppm)

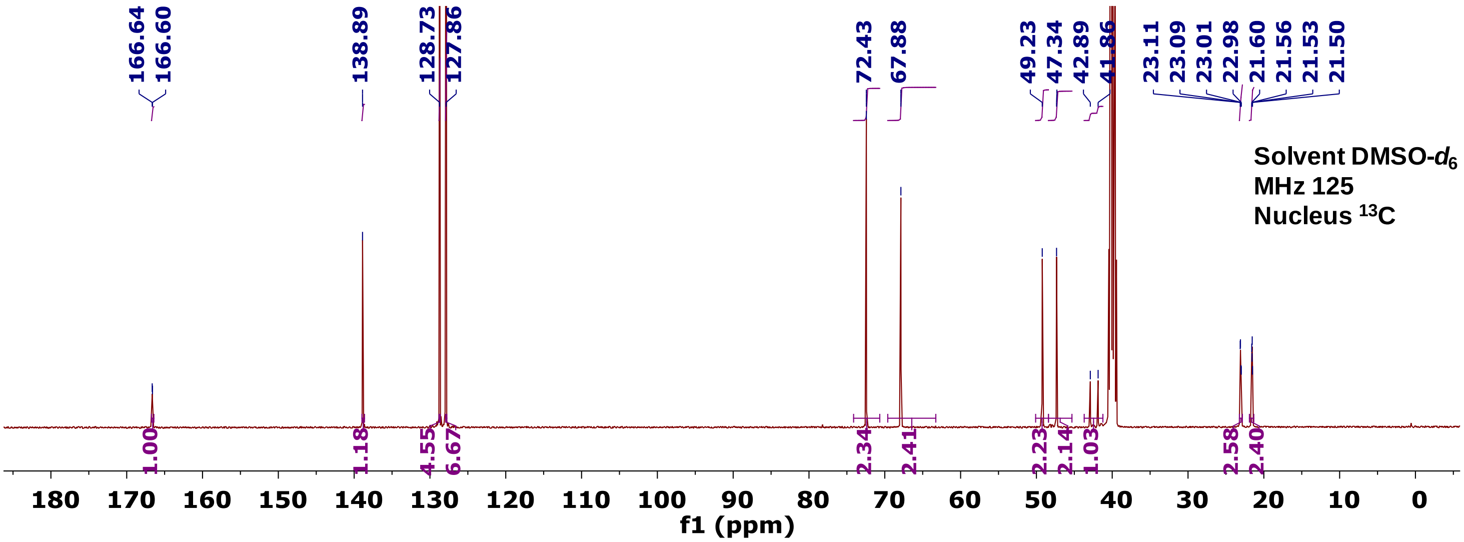
Solvent DMSO-d_6
MHz 125
Nucleus ^{13}C
f1 (ppm)

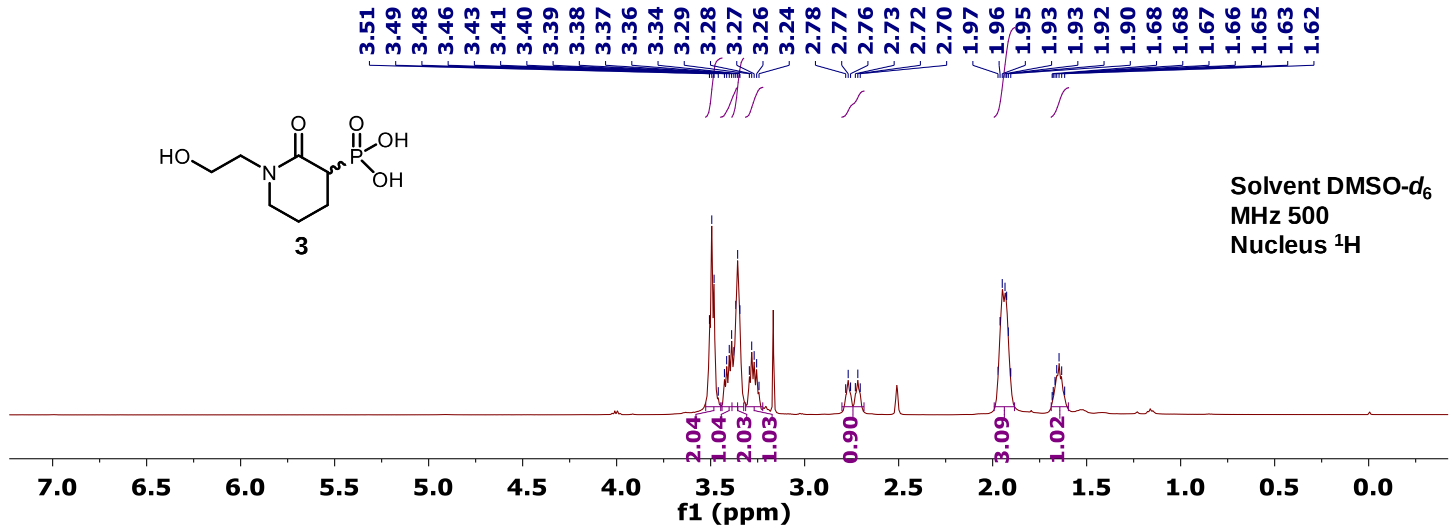
3.51 3.49 3.48 3.46 3.43 3.41 3.40 3.39 3.38 3.37 3.36 3.34 3.29 3.28 3.27 3.26 3.24 2.78 2.77 2.76 2.73 2.72 2.70 1.97 1.96 1.95 1.93 1.93 1.92 1.90 1.68 1.68 1.67 1.66 1.65 1.63 1.62
HO
N
O
O
P
OH
OH
3
Solvent DMSO-d_6
MHz 500
Nucleus ^{1}H
2.04 1.04 2.03 1.03 0.90 3.09 1.02
7.0 6.5 6.0 5.5 5.0 4.5 4.0 3.5 3.0 2.5 2.0 1.5 1.0 0.5 0.0
f1 (ppm)

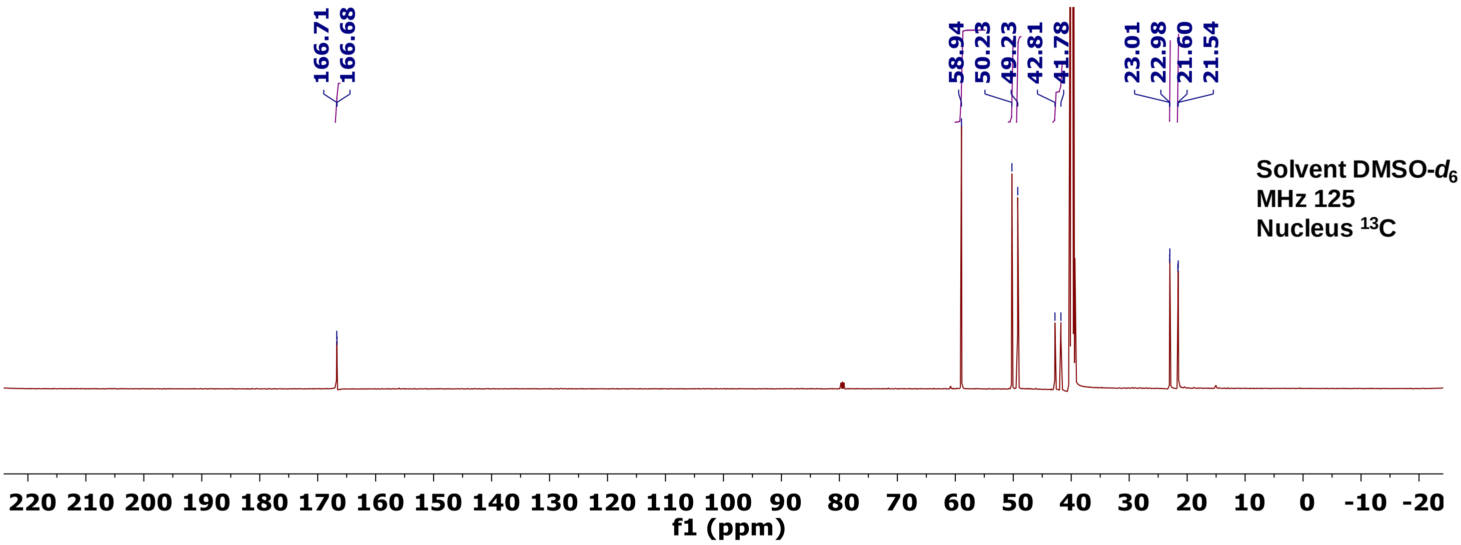
166.71 166.68
58.94 50.23 49.23 42.81 41.78
23.01 22.98 21.60 21.54
Solvent DMSO-d_6
MHz 125
Nucleus ^{13}C
220 210 200 190 180 170 160 150 140 130 120 110 100 90 80 70 60 50 40 30 20 10 0 -10 -20
f1 (ppm)

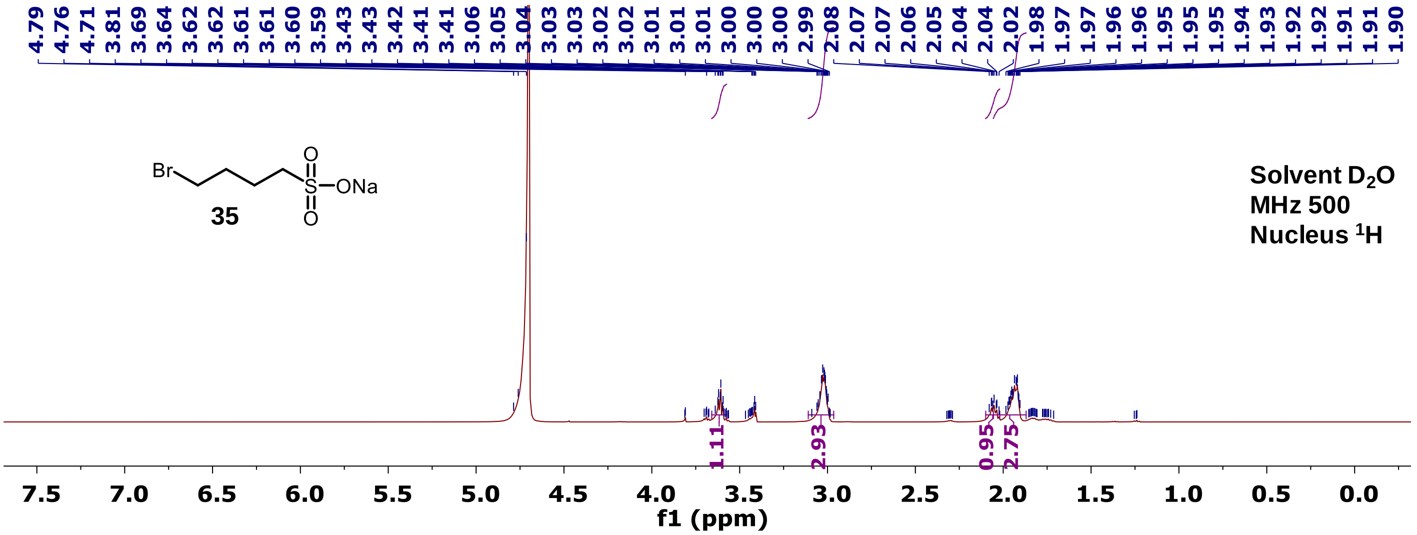
4.79 4.76 4.71 3.81 3.69 3.64 3.62 3.62 3.61 3.61 3.60 3.59 3.43 3.43 3.42 3.41 3.41 3.06 3.05 3.04 3.03 3.03 3.02 3.02 3.01 3.01 3.01 3.00 3.00 3.00 2.99 2.08 2.07 2.07 2.06 2.05 2.04 2.04 2.02 1.98 1.97 1.97 1.96 1.96 1.95 1.95 1.95 1.94 1.93 1.92 1.92 1.91 1.91 1.90
Br
O
S—ONa
O
35
Solvent D_2O
MHz 500
Nucleus ^1H
1.11
2.93
0.95
2.75
7.5 7.0 6.5 6.0 5.5 5.0 4.5 4.0 3.5 3.0 2.5 2.0 1.5 1.0 0.5 0.0
f1 (ppm)

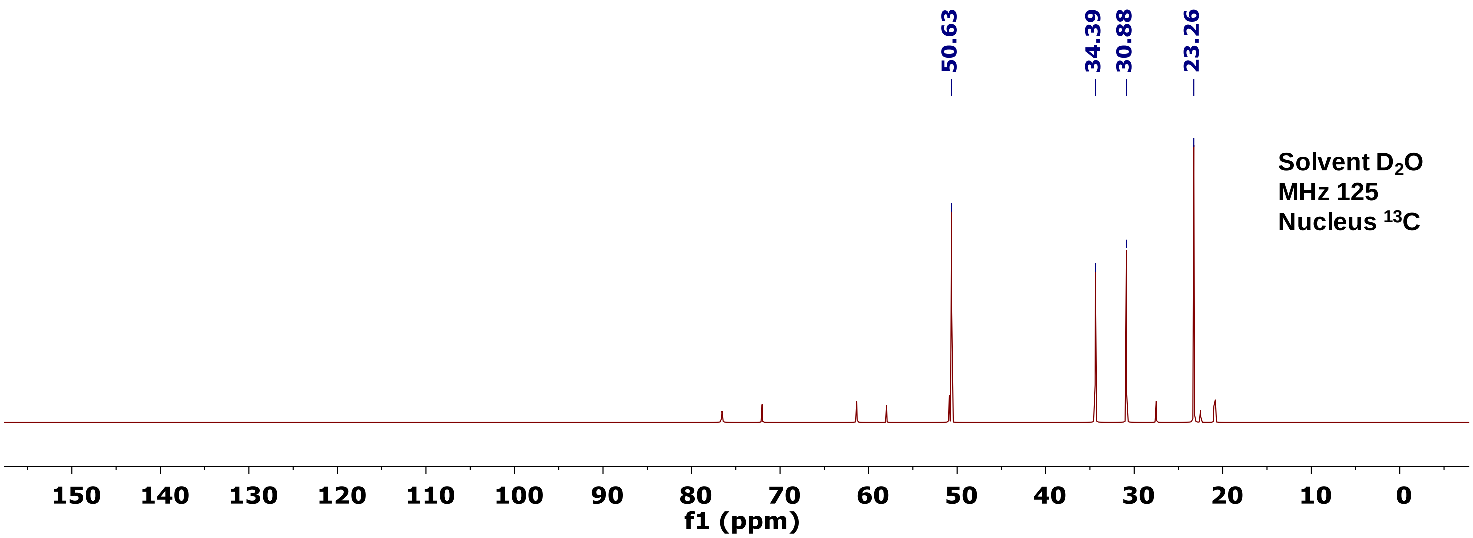
50.63
34.39
30.88
23.26
Solvent D_2O
MHz 125
Nucleus ^{13}C
150 140 130 120 110 100 90 80 70 60 50 40 30 20 10 0
f1 (ppm)

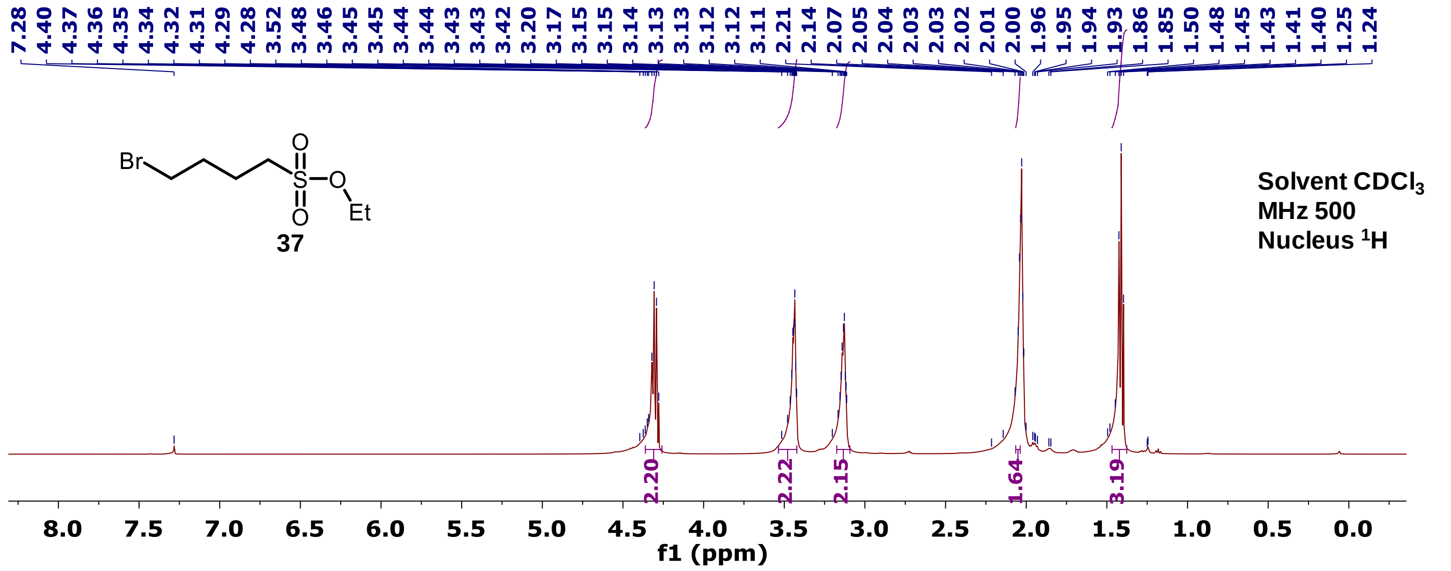
7.28 4.40 4.37 4.36 4.35 4.34 4.32 4.31 4.29 4.28 3.52 3.48 3.46 3.45 3.45 3.44 3.44 3.43 3.43 3.42 3.20 3.17 3.15 3.15 3.14 3.13 3.13 3.12 3.12 3.11 2.21 2.14 2.07 2.05 2.04 2.03 2.03 2.02 2.01 2.00 1.96 1.95 1.94 1.93 1.86 1.85 1.50 1.48 1.45 1.43 1.41 1.40 1.25 1.24
Br
O
S
O
O
Et
37
Solvent $CDCl_3$
MHz 500
Nucleus ^1H
2.20
2.22
2.15
1.64
3.19
8.0 7.5 7.0 6.5 6.0 5.5 5.0 4.5 4.0 3.5 3.0 2.5 2.0 1.5 1.0 0.5 0.0
f1 (ppm)

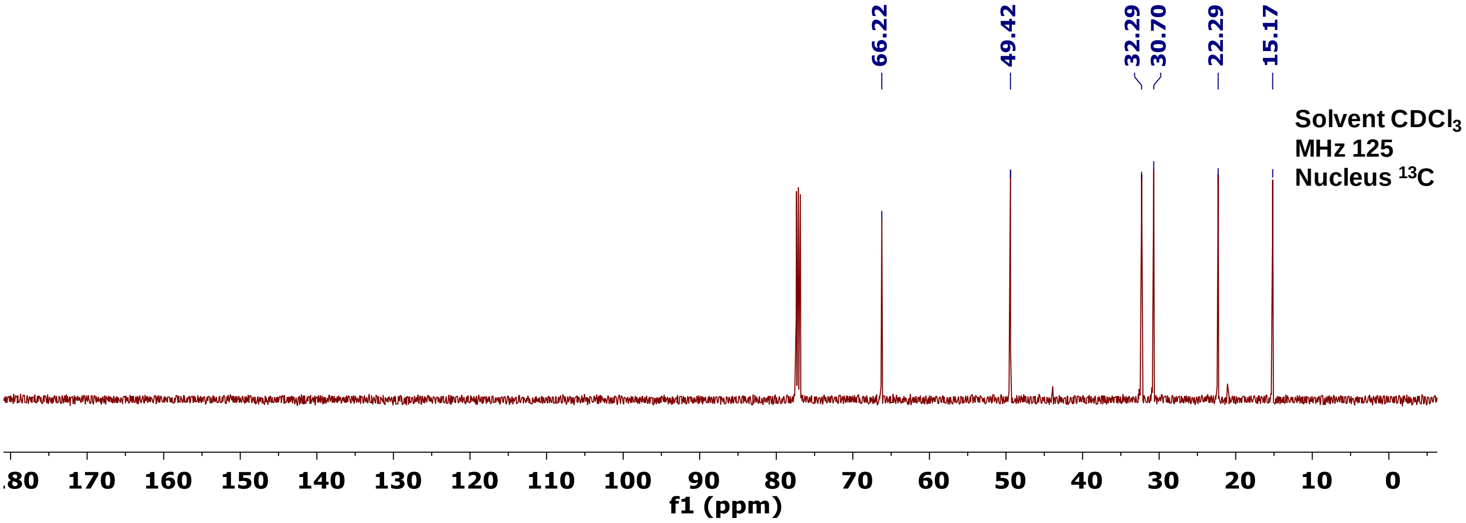
66.22
49.42
32.29
30.70
22.29
15.17
Solvent $CDCl_3$
MHz 125
Nucleus ^{13}C
180 170 160 150 140 130 120 110 100 90 80 70 60 50 40 30 20 10 0
f1 (ppm)

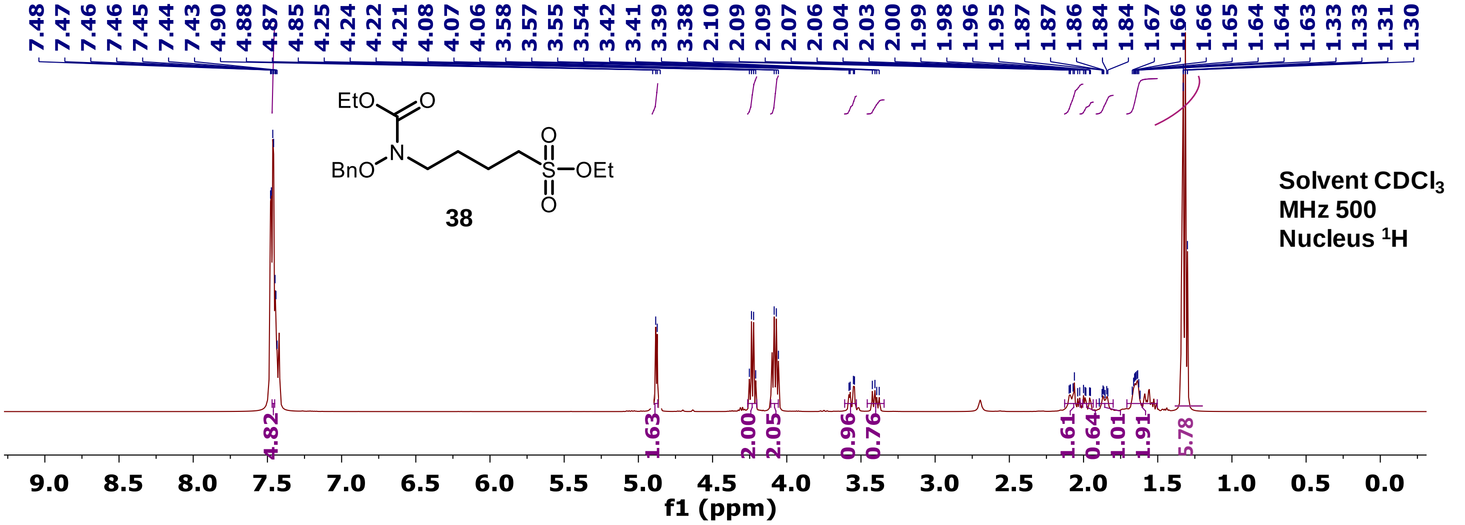
7.48 7.47 7.46 7.46 7.45 7.44 7.43 4.90 4.88 4.87 4.85 4.25 4.24 4.22 4.21 4.08 4.07 4.06 3.58 3.57 3.55 3.54 3.42 3.41 3.39 3.38 2.10 2.09 2.09 2.07 2.06 2.04 2.03 2.00 1.99 1.98 1.96 1.95 1.87 1.87 1.86 1.84 1.84 1.67 1.66 1.66 1.65 1.64 1.64 1.63 1.33 1.33 1.31 1.30
EtO
O
N
BnO
S
O
O
OEt
38
Solvent $CDCl_3$
MHz 500
Nucleus ^1H
4.82 1.63 2.00 2.05 0.96 0.76 1.61 0.64 1.01 1.91 5.78
9.0 8.5 8.0 7.5 7.0 6.5 6.0 5.5 5.0 4.5 4.0 3.5 3.0 2.5 2.0 1.5 1.0 0.5 0.0
f1 (ppm)

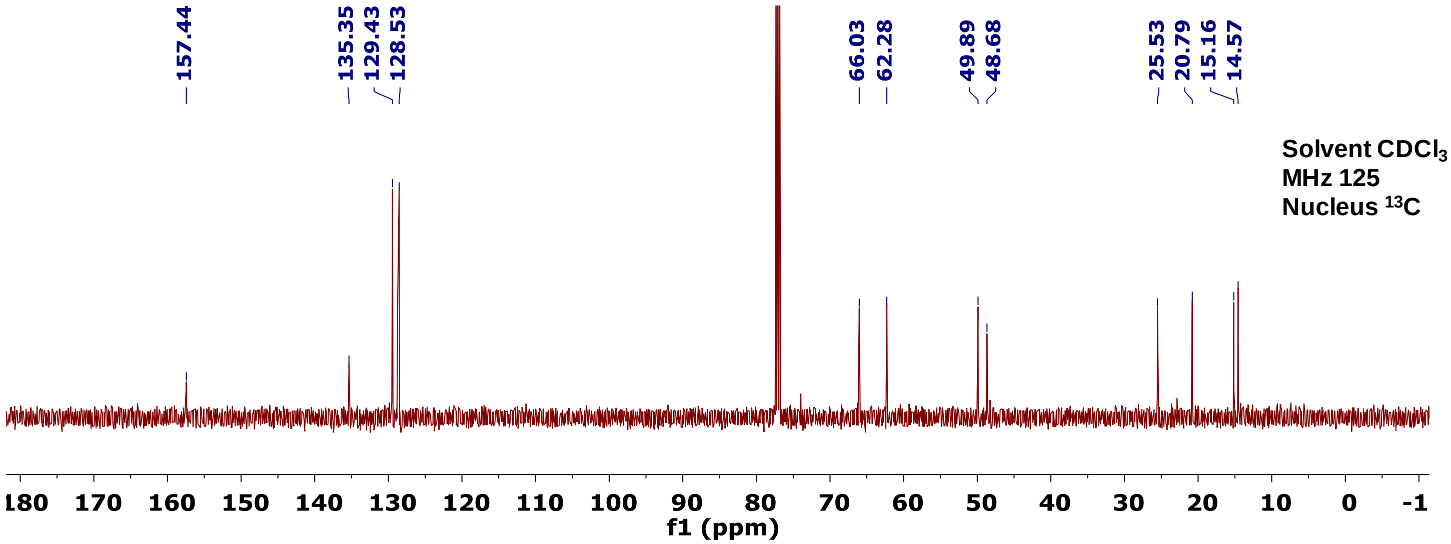
157.44 135.35 129.43 128.53 66.03 62.28 49.89 48.68 25.53 20.79 15.16 14.57
Solvent $CDCl_3$
MHz 125
Nucleus ^{13}C
180 170 160 150 140 130 120 110 100 90 80 70 60 50 40 30 20 10 0 -1
f1 (ppm)

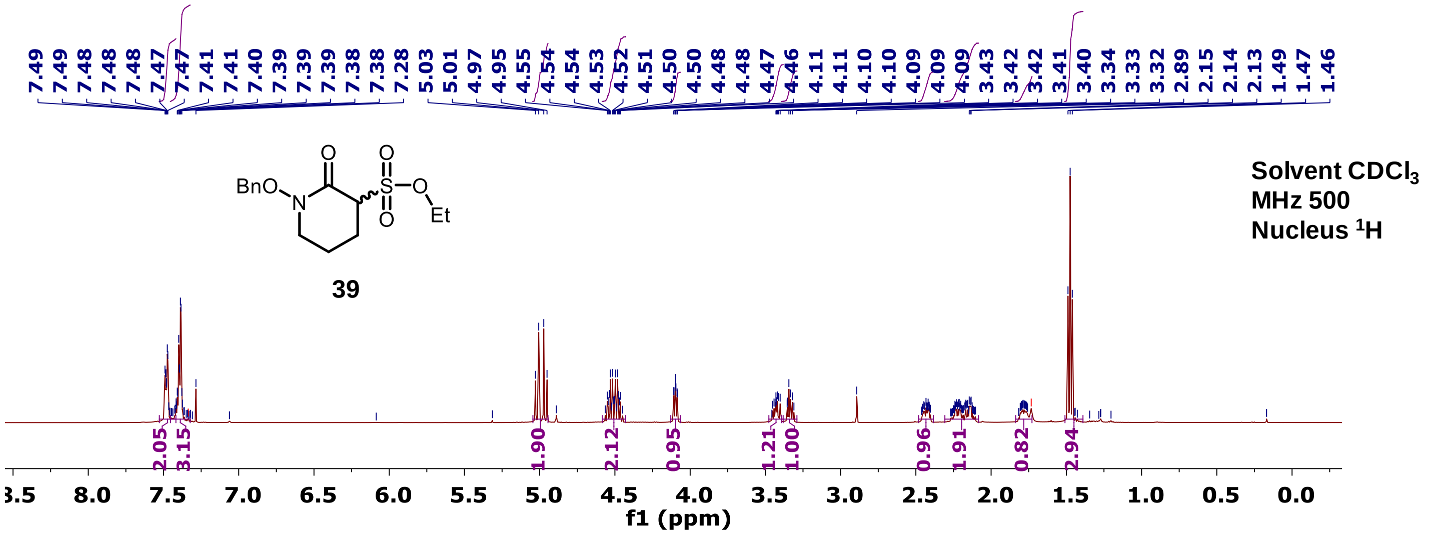
7.49 7.49 7.48 7.48 7.48 7.47 7.47 7.41 7.41 7.40 7.39 7.39 7.39 7.38 7.38 7.28 5.03 5.01 4.97 4.95 4.55 4.54 4.54 4.53 4.52 4.51 4.50 4.50 4.48 4.48 4.47 4.46 4.11 4.10 4.10 4.09 4.09 4.09 3.43 3.42 3.42 3.41 3.40 3.34 3.33 3.32 2.89 2.15 2.14 2.13 1.49 1.47 1.46
BnO
N
O
S
O
O
O
Et
39
Solvent CDCl$_3$
MHz 500
Nucleus ^{1}H
2.05 3.15 1.90 2.12 0.95 1.21 1.00 0.96 1.91 0.82 2.94
[illegible] 8.0 7.5 7.0 6.5 6.0 5.5 5.0 4.5 4.0 3.5 [illegible] 2.5 2.0 1.5 [illegible] 0.5 0.0
f1 (ppm)

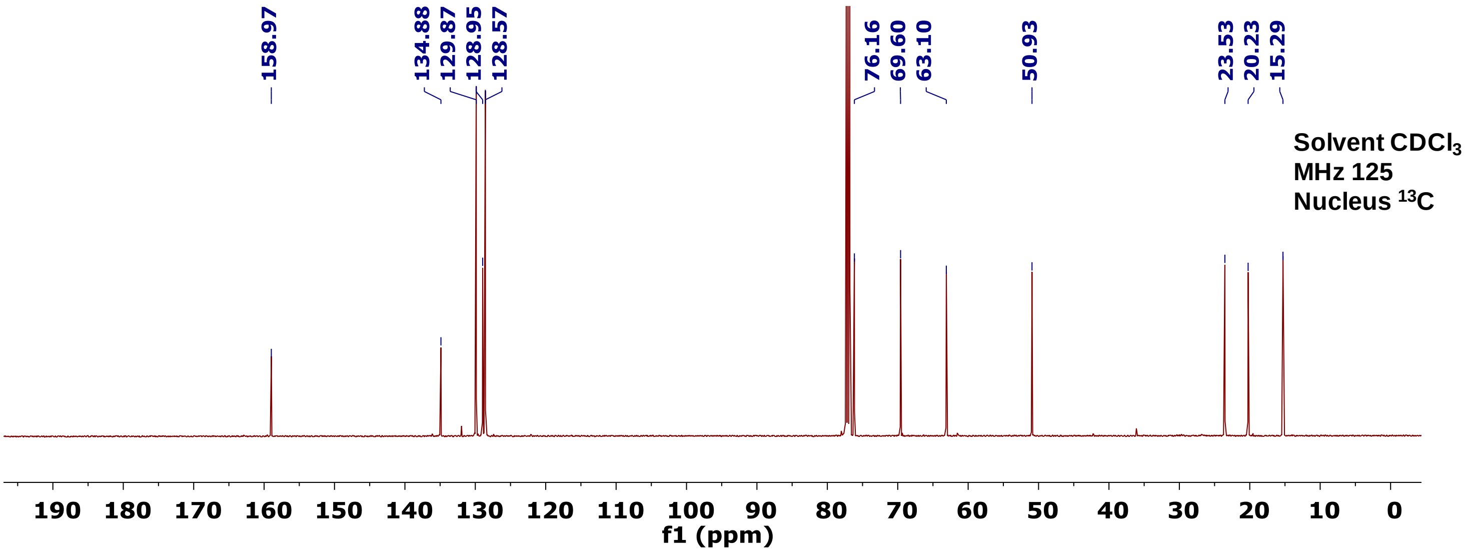
158.97
134.88 129.87 128.95 128.57
76.16 69.60 63.10
50.93
23.53 20.23 15.29
Solvent CDCl$_3$
MHz 125
Nucleus ^{13}C
190 180 170 160 150 140 130 120 110 100 90 80 70 60 50 40 30 20 10 0
f1 (ppm)

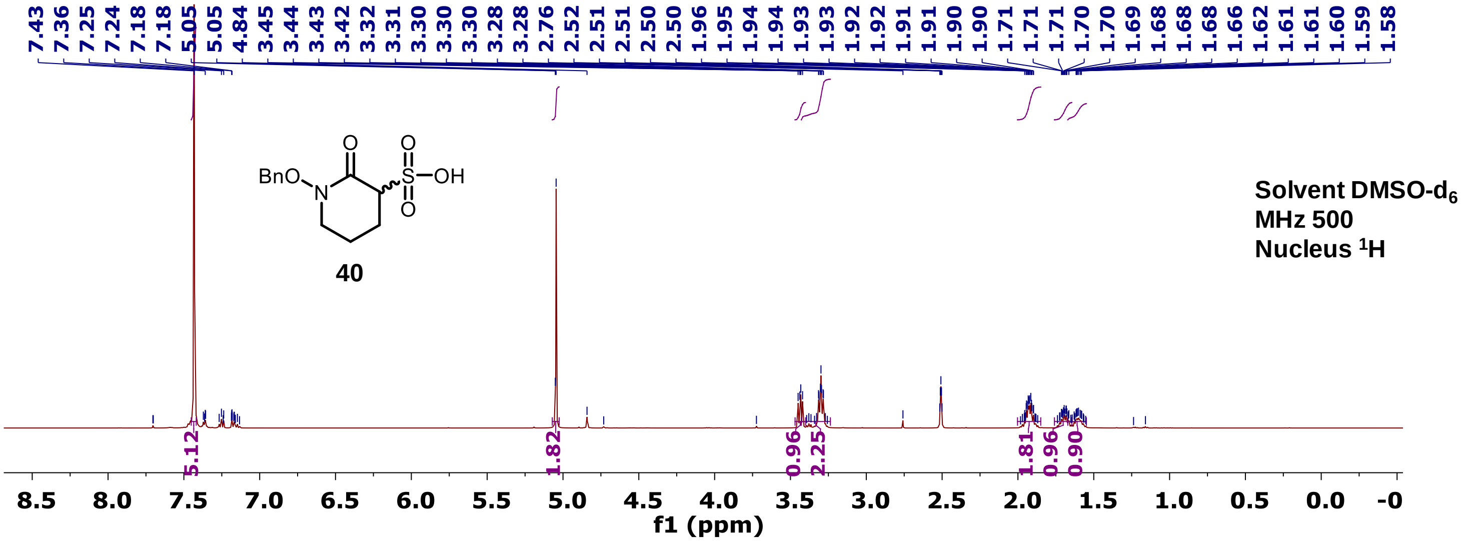
7.43
7.36
7.25
7.24
7.18
7.18
5.05
5.05
4.84
3.45
3.44
3.43
3.42
3.32
3.31
3.30
3.30
3.30
3.28
3.28
2.76
2.52
2.51
2.51
2.50
2.50
1.96
1.95
1.94
1.94
1.93
1.93
1.92
1.92
1.91
1.91
1.90
1.90
1.71
1.71
1.71
1.70
1.70
1.69
1.68
1.68
1.68
1.66
1.62
1.61
1.61
1.60
1.59
1.58
BnO
N
O
S
O
O
OH
40
Solvent DMSO-d6
MHz 500
Nucleus 1H
5.12
1.82
0.96
2.25
1.81
0.96
0.90
8.5 8.0 7.5 7.0 6.5 6.0 5.5 5.0 4.5 4.0 3.5 3.0 2.5 2.0 1.5 1.0 0.5 0.0 -0
f1 (ppm)

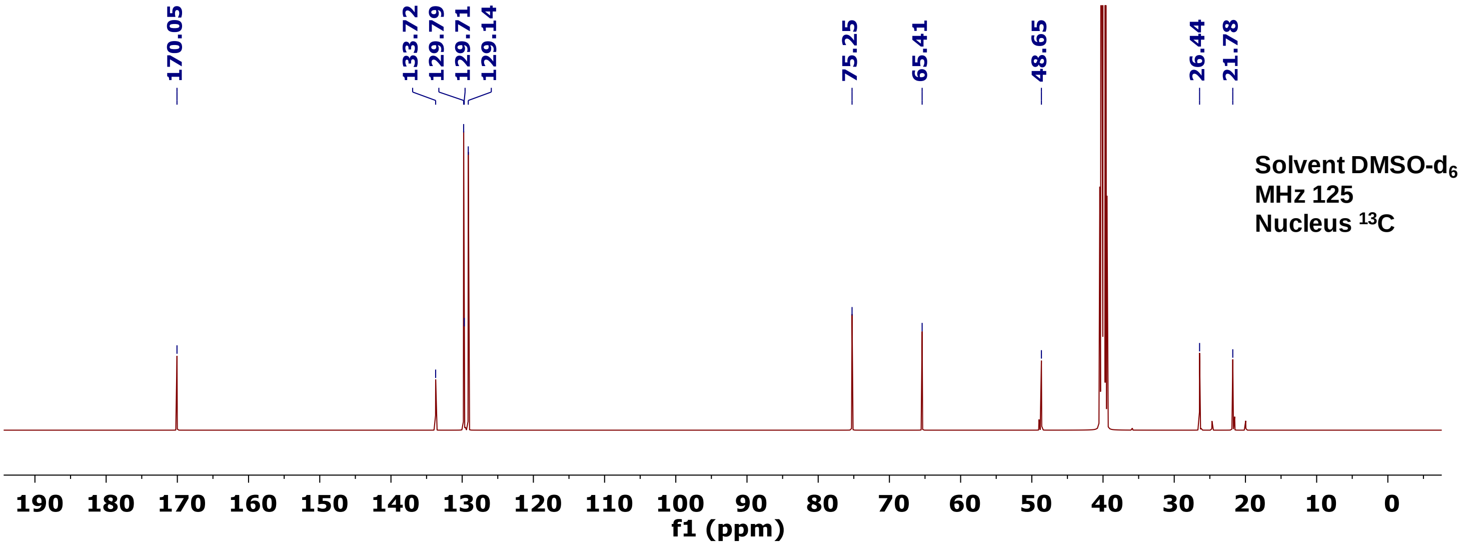
170.05
133.72
129.79
129.71
129.14
75.25
65.41
48.65
26.44
21.78
Solvent DMSO-d6
MHz 125
Nucleus 13C
190 180 170 160 150 140 130 120 110 100 90 80 70 60 50 40 30 20 10 0
f1 (ppm)

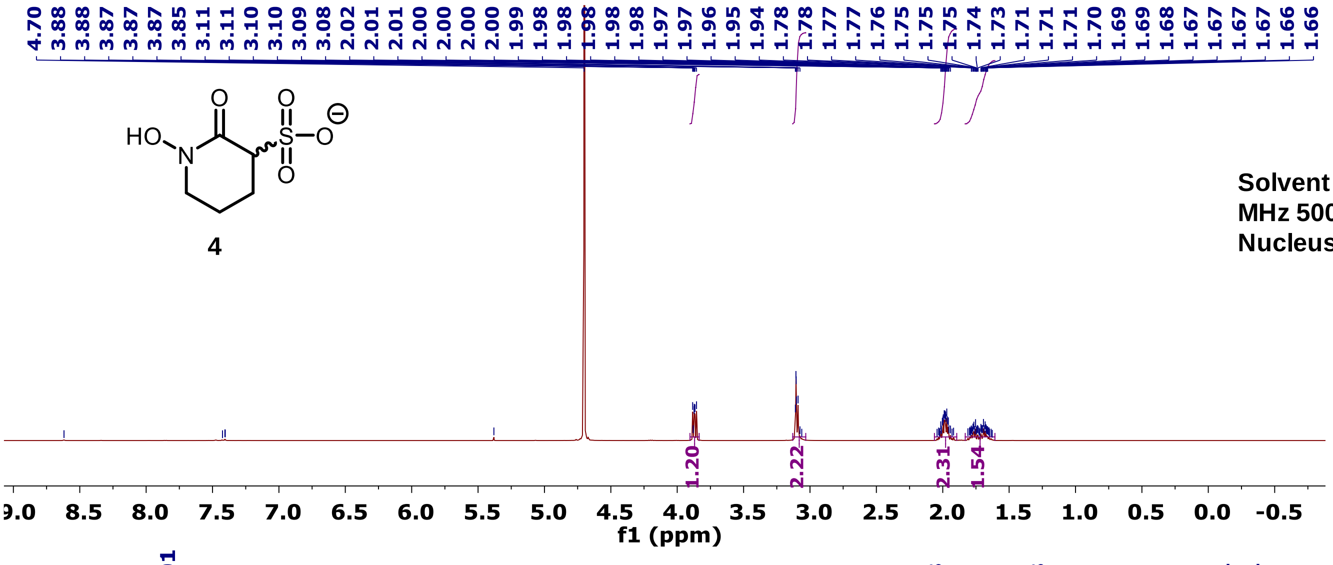
4.70 3.88 3.88 3.87 3.87 3.87 3.85 3.11 3.11 3.10 3.10 3.09 3.08 2.02 2.01 2.01 2.00 2.00 2.00 2.00 1.99 1.98 1.98 1.98 1.98 1.98 1.97 1.97 1.96 1.95 1.94 1.78 1.78 1.77 1.77 1.76 1.75 1.75 1.75 1.74 1.73 1.71 1.71 1.71 1.70 1.69 1.69 1.68 1.67 1.67 1.67 1.67 1.66 1.66
HO
N
O
S
O
4
Solvent D2O
MHz 500
Nucleus 1H
1.20
2.22
2.31
1.54
f1 (ppm)

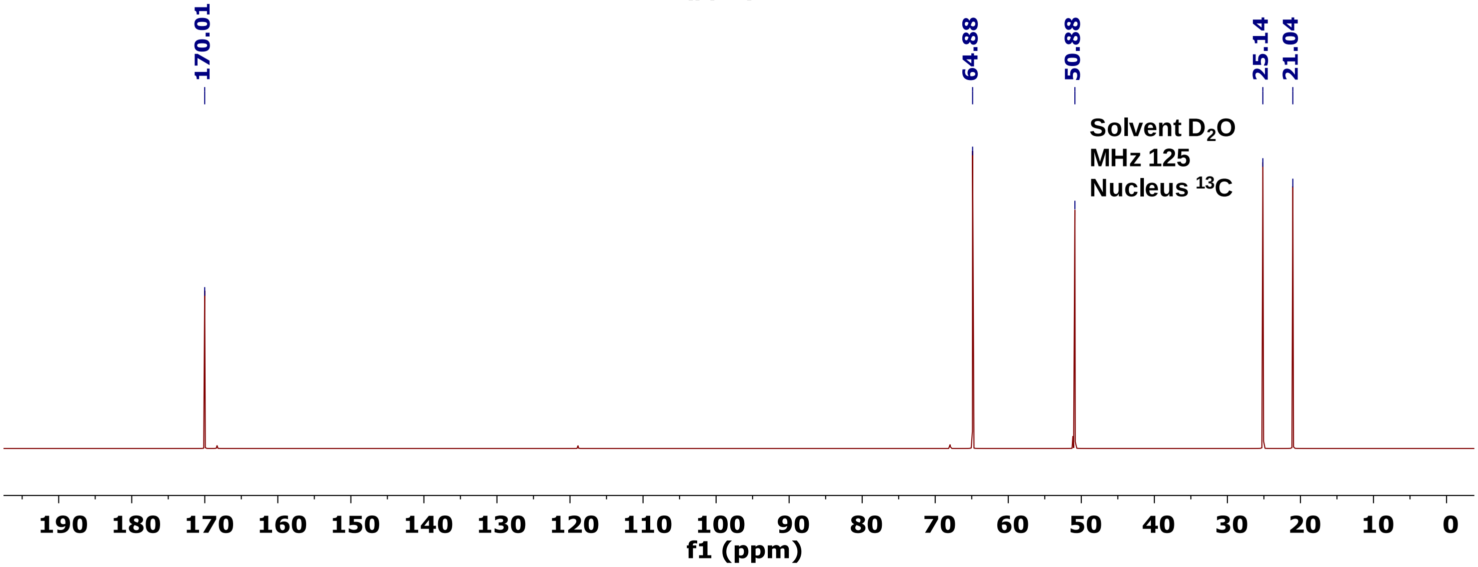
170.01
64.88
50.88
25.14
21.04
Solvent D2O
MHz 125
Nucleus 13C
f1 (ppm)

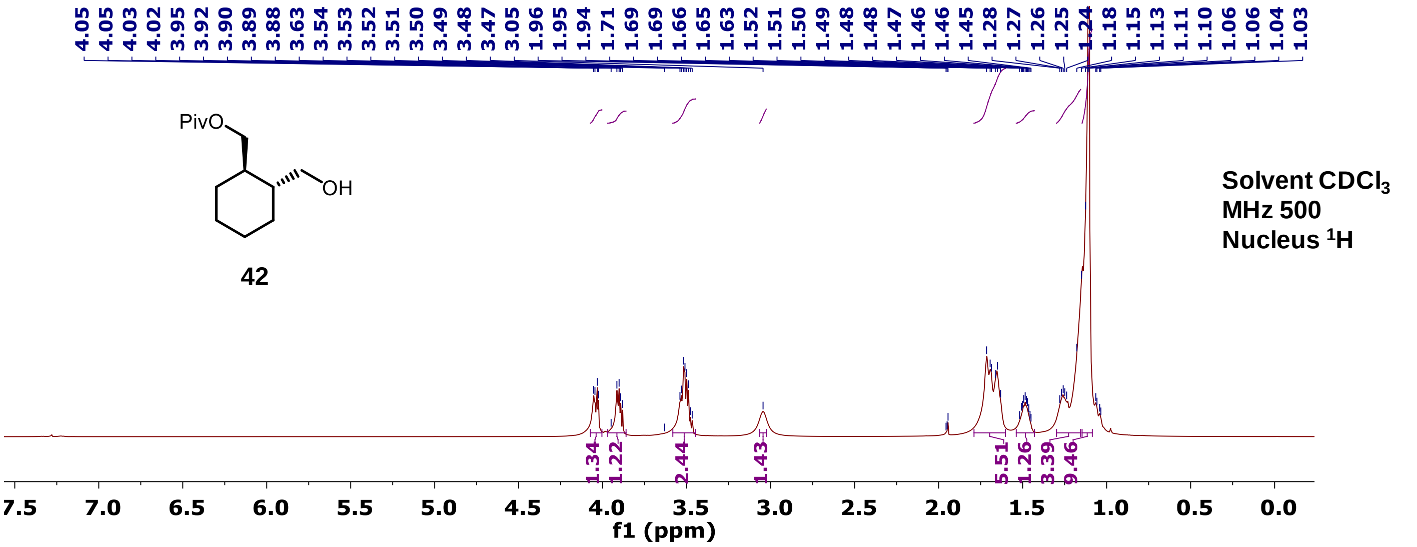
4.05 4.05 4.03 4.02 3.95 3.92 3.90 3.89 3.88 3.63 3.54 3.53 3.52 3.51 3.50 3.49 3.48 3.47 3.05 1.96 1.95 1.94 1.71 1.69 1.69 1.66 1.65 1.63 1.52 1.51 1.50 1.49 1.48 1.48 1.47 1.46 1.46 1.45 1.28 1.27 1.26 1.25 1.24 1.18 1.15 1.13 1.11 1.10 1.06 1.06 1.04 1.03
PivO
OH
42
Solvent $CDCl_3$
MHz 500
Nucleus ^1H
1.34 1.22 2.44 1.43 5.51 1.26 3.39 9.46
7.5 7.0 6.5 6.0 5.5 5.0 4.5 4.0 3.5 3.0 2.5 2.0 1.5 1.0 0.5 0.0
f1 (ppm)

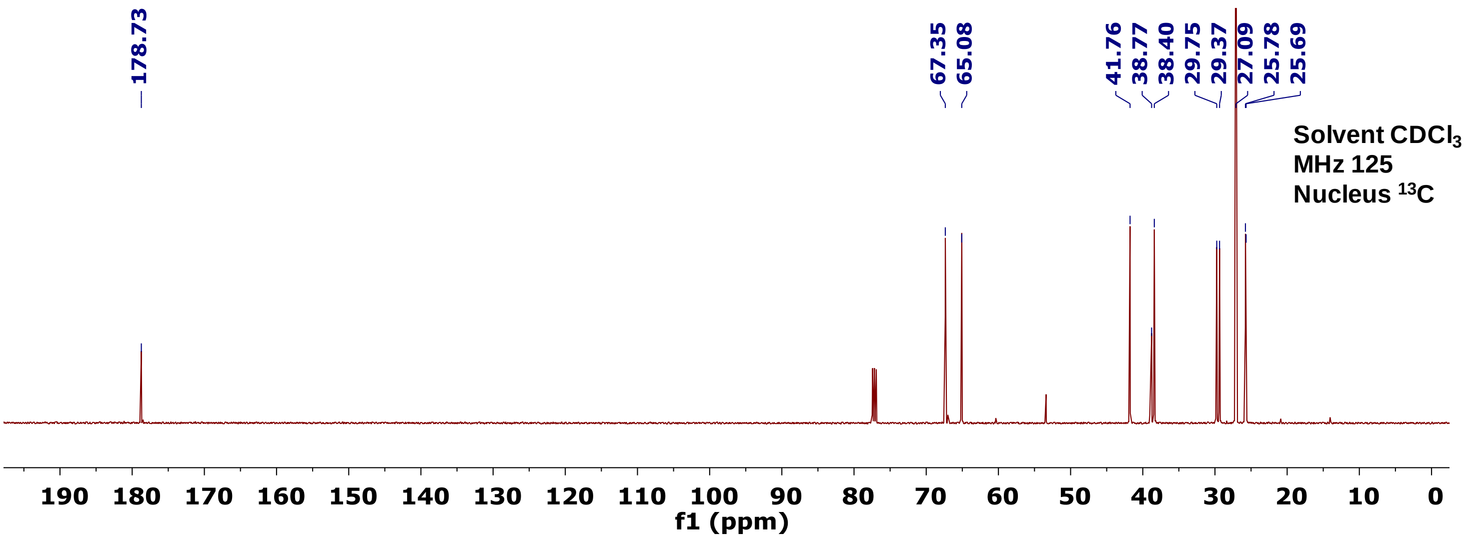
178.73
67.35 65.08
41.76 38.77 38.40 29.75 29.37 27.09 25.78 25.69
Solvent $CDCl_3$
MHz 125
Nucleus ^{13}C
190 180 170 160 150 140 130 120 110 100 90 80 70 60 50 40 30 20 10 0
f1 (ppm)

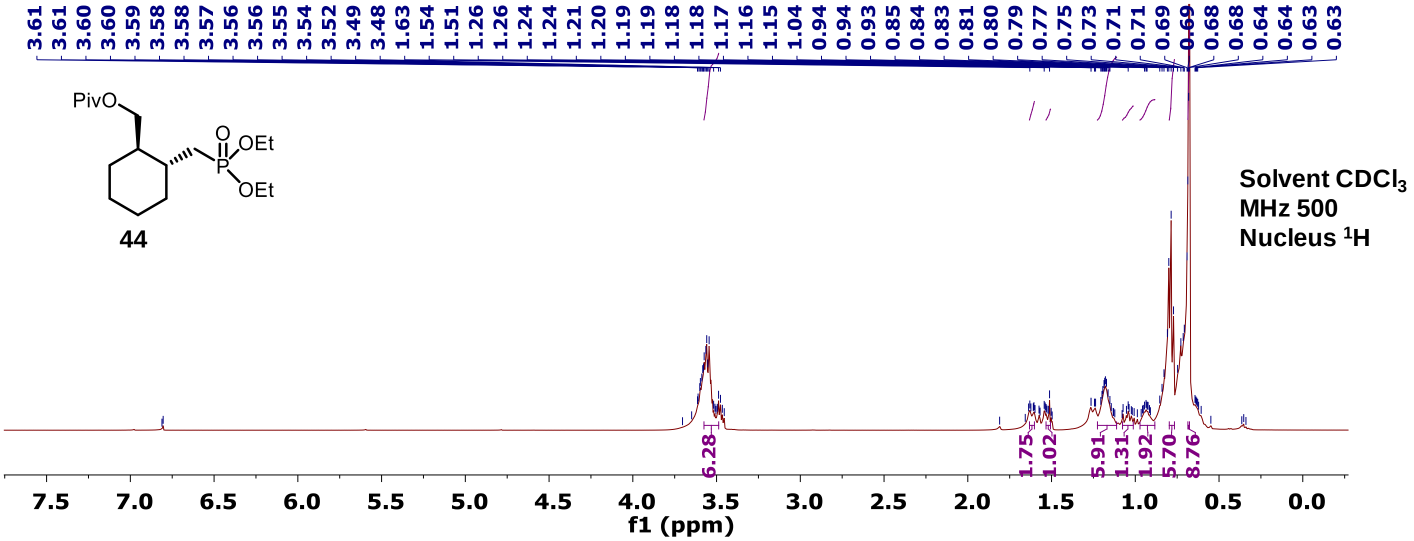
PivO
O
OEt
P
OEt
44
3.61 3.61 3.60 3.60 3.59 3.58 3.58 3.57 3.56 3.56 3.55 3.54 3.52 3.49 3.48 1.63 1.54 1.51 1.26 1.26 1.24 1.24 1.21 1.20 1.19 1.19 1.18 1.18 1.17 1.16 1.15 1.04 0.94 0.94 0.93 0.85 0.84 0.83 0.81 0.80 0.79 0.77 0.75 0.73 0.71 0.71 0.69 0.69 0.68 0.68 0.64 0.64 0.63 0.63
Solvent $CDCl_3$
MHz 500
Nucleus ^1H
6.28
1.75
1.02
5.91
1.31
1.92
5.70
8.76
7.5 7.0 6.5 6.0 5.5 5.0 4.5 4.0 3.5 3.0 2.5 2.0 1.5 1.0 0.5 0.0
f1 (ppm)

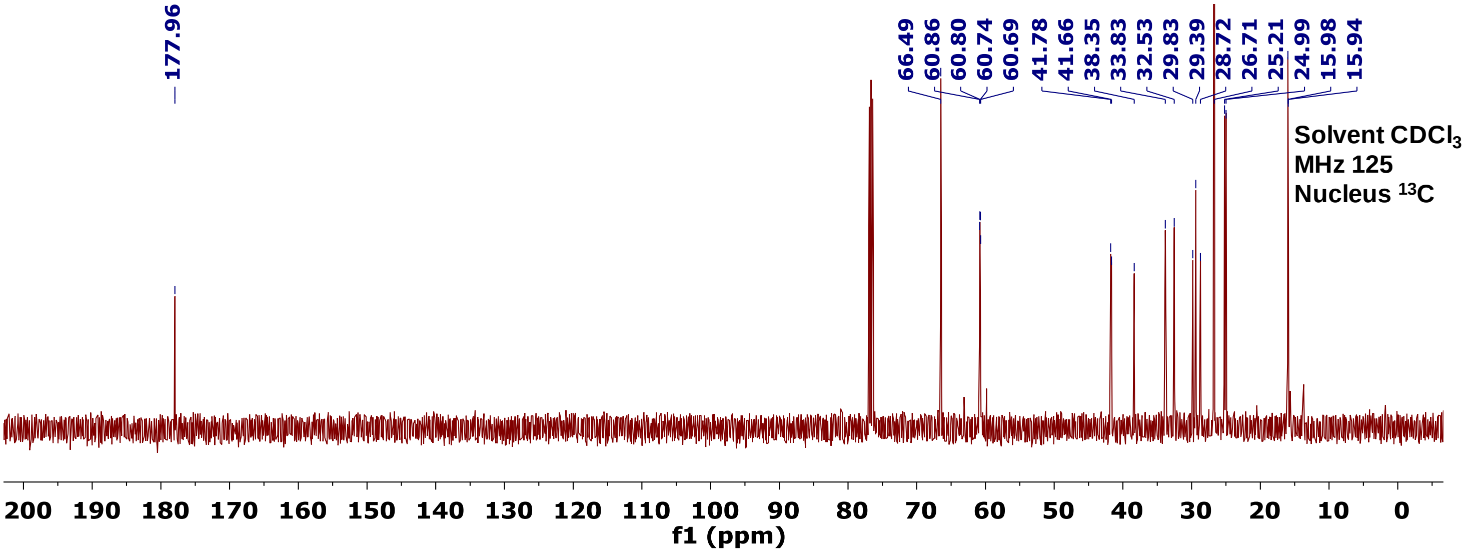
177.96
66.49 60.86 60.80 60.74 60.69 41.78 41.66 38.35 33.83 32.53 29.83 29.39 28.72 26.71 25.21 24.99 15.98 15.94
Solvent $CDCl_3$
MHz 125
Nucleus ^{13}C
200 190 180 170 160 150 140 130 120 110 100 90 80 70 60 50 40 30 20 10 0
f1 (ppm)

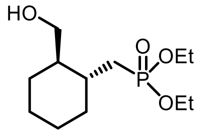
HO
O
P
OEt
OEt

45

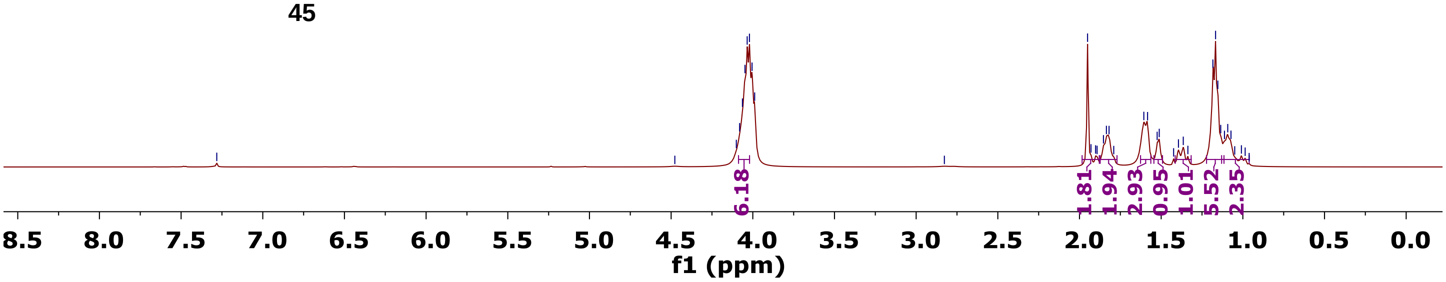
7.28
4.48
4.10
4.08
4.06
4.05
4.03
4.02
4.00
3.99
2.83
1.95
1.93
1.90
1.89
1.85
1.84
1.82
1.79
1.61
1.58
1.52
1.51
1.42
1.39
1.36
1.34
1.18
1.17
1.15
1.14
1.11
1.09
1.07
1.05
1.01
0.99
0.96
Solvent $CDCl_3$
MHz 500
Nucleus ^{1}H
6.18
1.81
1.94
2.93
0.95
1.01
5.52
2.35
8.5 8.0 7.5 7.0 6.5 6.0 5.5 5.0 4.5 4.0 3.5 3.0 2.5 2.0 1.5 1.0 0.5 0.0
f1 (ppm)

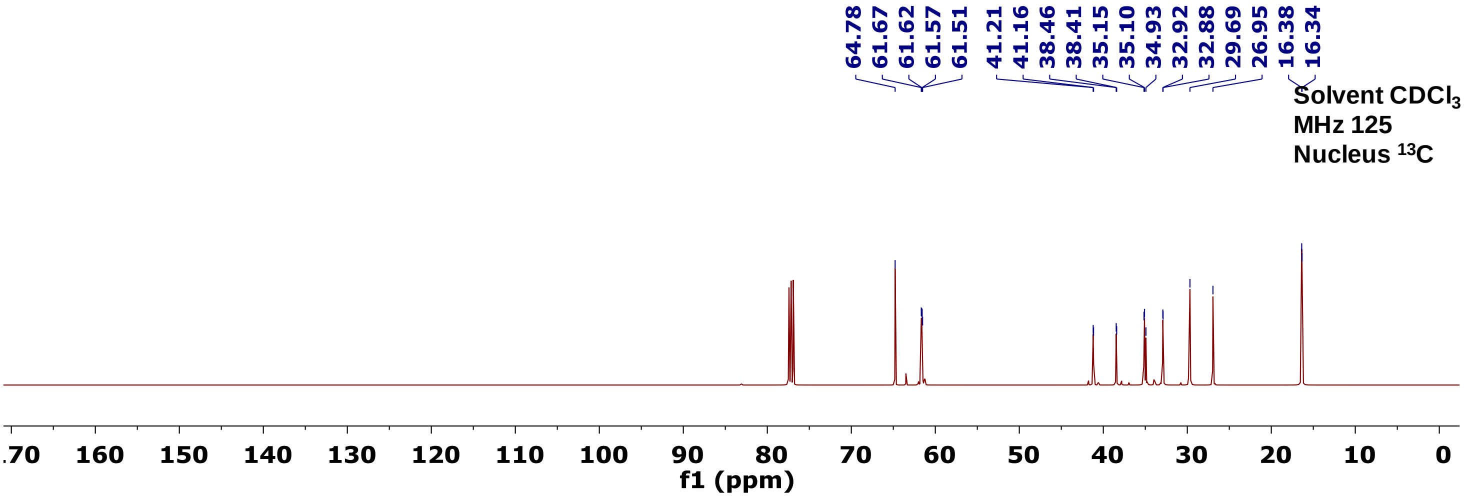
64.78
61.67
61.62
61.57
61.51
41.21
41.16
38.46
38.41
35.15
35.10
34.93
32.92
32.88
29.69
26.95
16.38
16.34
Solvent $CDCl_3$
MHz 125
Nucleus ^{13}C
.70 160 150 140 130 120 110 100 90 80 70 60 50 40 30 20 10 0
f1 (ppm)

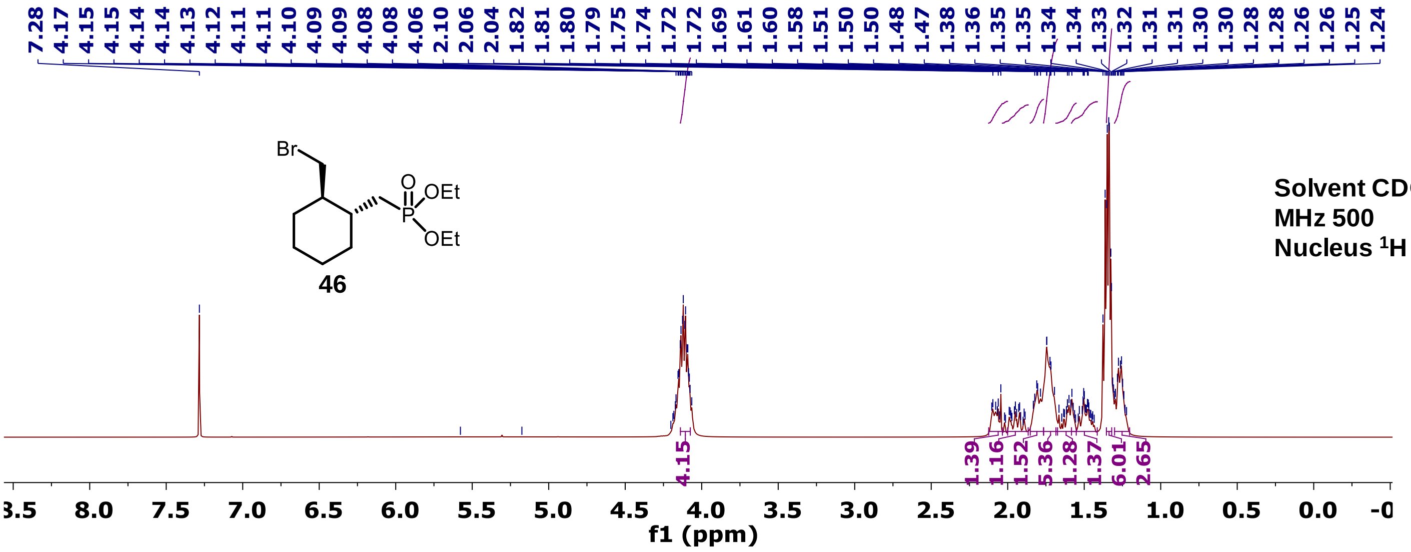
7.28 4.17 4.15 4.15 4.14 4.14 4.13 4.12 4.11 4.11 4.10 4.09 4.09 4.08 4.08 4.06 2.10 2.06 2.04 1.82 1.81 1.80 1.79 1.75 1.74 1.72 1.72 1.69 1.61 1.60 1.58 1.51 1.50 1.50 1.48 1.47 1.38 1.36 1.35 1.35 1.34 1.34 1.33 1.32 1.31 1.31 1.30 1.30 1.28 1.28 1.26 1.26 1.25 1.24
Br
O
OEt
P
OEt
46
Solvent CDCl3
MHz 500
Nucleus 1H
4.15
1.39 1.16 1.52 5.36 1.28 1.37 6.01 2.65
f1 (ppm)

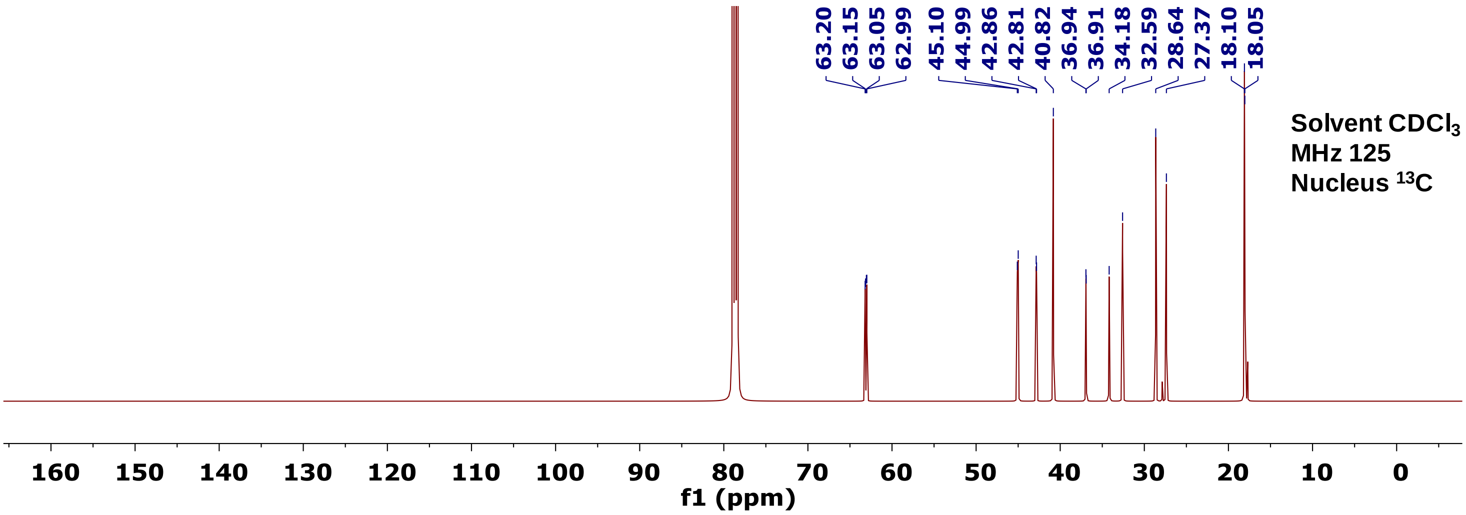
63.20 63.15 63.05 62.99 45.10 44.99 42.86 42.81 40.82 36.94 36.91 34.18 32.59 28.64 27.37 18.10 18.05
Solvent CDCl3
MHz 125
Nucleus 13C
f1 (ppm)

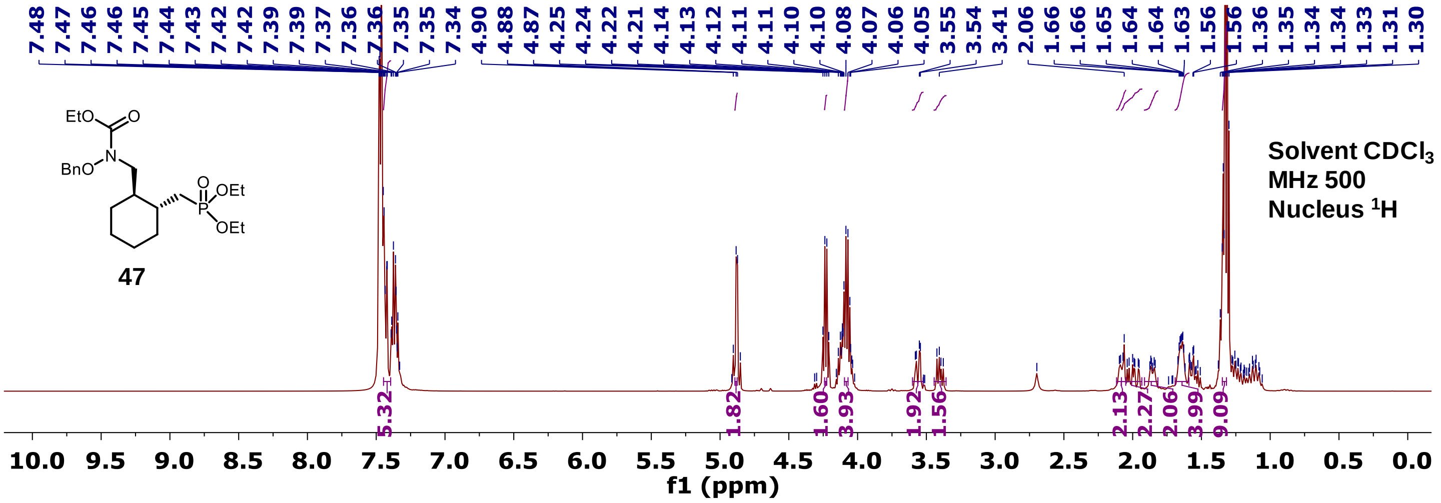
EtO
O
N
BnO
O
P
OEt
OEt
47
Solvent CDCl3
MHz 500
Nucleus 1H
7.48 7.47 7.46 7.46 7.45 7.44 7.43 7.42 7.42 7.39 7.39 7.37 7.36 7.36 7.35 7.35 7.34 4.90 4.88 4.87 4.25 4.24 4.22 4.21 4.14 4.13 4.12 4.11 4.11 4.10 4.10 4.08 4.07 4.06 4.05 3.55 3.54 3.41 2.06 1.66 1.66 1.65 1.64 1.63 1.63 1.56 1.56 1.36 1.35 1.34 1.34 1.33 1.31 1.30
5.32 1.82 1.60 3.93 1.92 1.56 2.13 2.27 2.06 3.99 9.09
10.0 9.5 9.0 8.5 8.0 7.5 7.0 6.5 6.0 5.5 5.0 4.5 4.0 3.5 3.0 2.5 2.0 1.5 1.0 0.5 0.0
f1 (ppm)

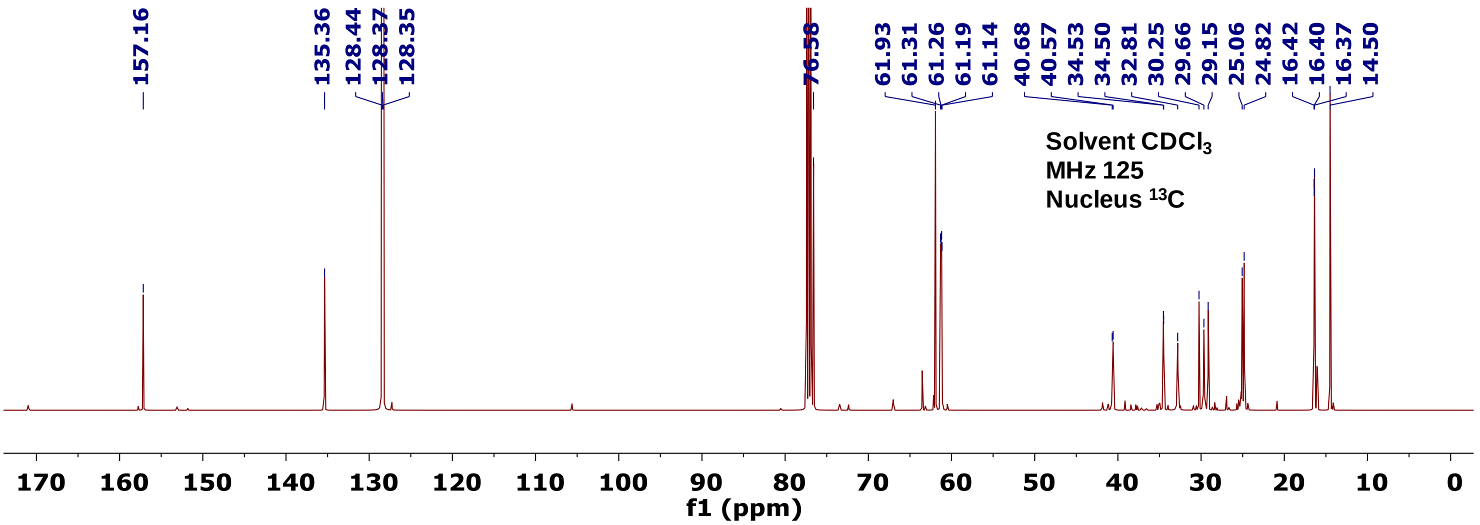
157.16
135.36 128.44 128.37 128.35
76.58
61.93 61.31 61.26 61.19 61.14 40.68 40.57 34.53 34.50 32.81 30.25 29.66 29.15 25.06 24.82 16.42 16.40 16.37 14.50
Solvent CDCl3
MHz 125
Nucleus 13C
170 160 150 140 130 120 110 100 90 80 70 60 50 40 30 20 10 0
f1 (ppm)

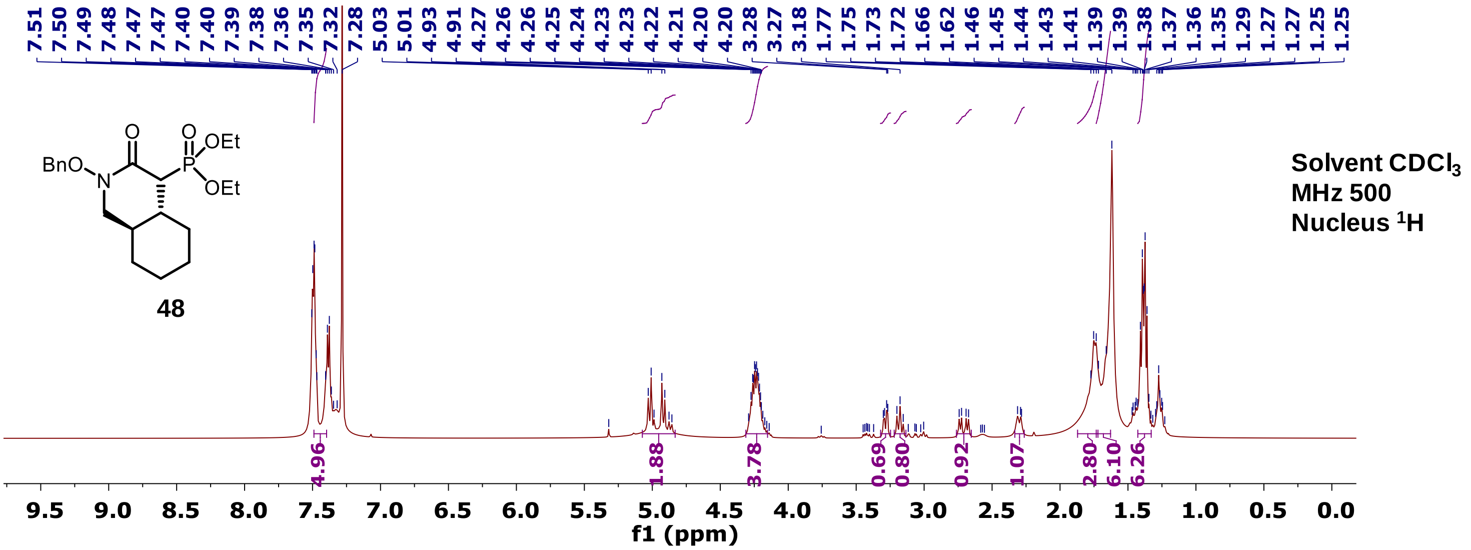
7.51 7.50 7.49 7.48 7.47 7.47 7.40 7.40 7.39 7.38 7.36 7.35 7.32 7.28 5.03 5.01 4.93 4.91 4.27 4.26 4.26 4.25 4.24 4.23 4.23 4.22 4.21 4.20 4.20 3.28 3.27 3.18 1.77 1.75 1.73 1.72 1.66 1.62 1.46 1.45 1.44 1.43 1.41 1.39 1.39 1.38 1.37 1.36 1.35 1.29 1.27 1.27 1.25 1.25
BnO
N
O
P
OEt
OEt
48
Solvent $CDCl_3$
MHz 500
Nucleus ^1H
4.96 1.88 3.78 0.69 0.80 0.92 1.07 2.80 6.10 6.26
9.5 9.0 8.5 8.0 7.5 7.0 6.5 6.0 5.5 5.0 4.5 4.0 3.5 3.0 2.5 2.0 1.5 1.0 0.5 0.0
f1 (ppm)

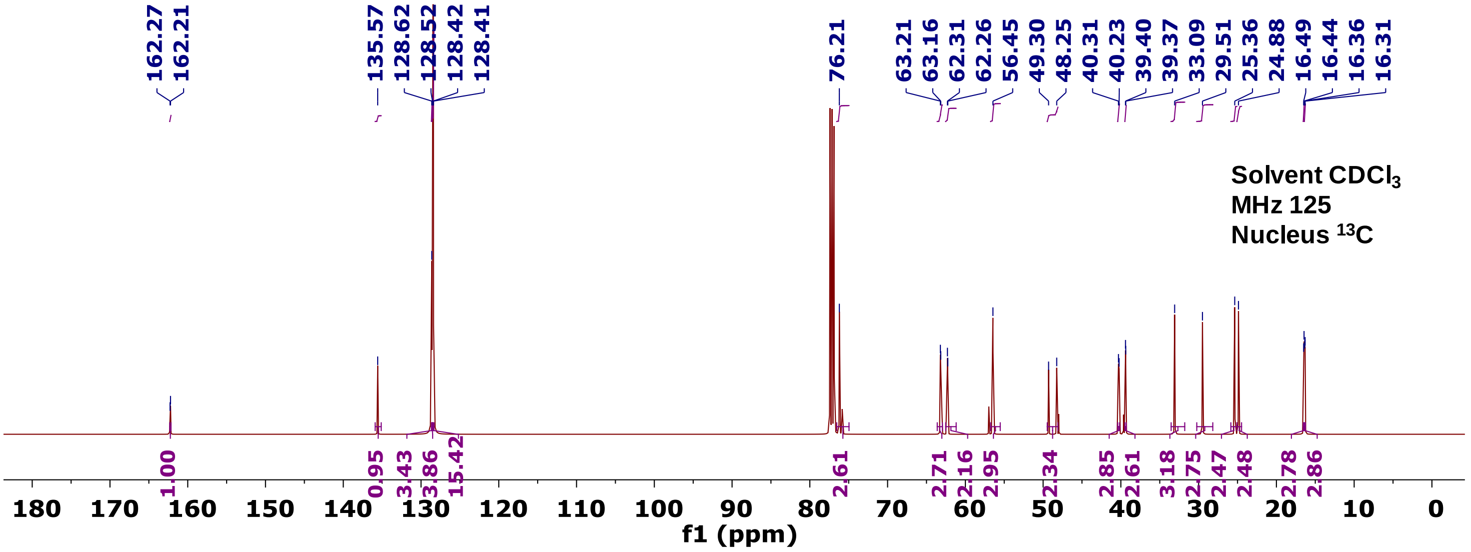
162.27 162.21 135.57 128.62 128.52 128.42 128.41 76.21 63.21 63.16 62.31 62.26 56.45 49.30 48.25 40.31 40.23 39.40 39.37 33.09 29.51 25.36 24.88 16.49 16.44 16.36 16.31
Solvent $CDCl_3$
MHz 125
Nucleus ^{13}C
1.00 0.95 3.43 3.86 15.42 2.61 2.71 2.16 2.95 2.34 2.85 2.61 3.18 2.75 2.47 2.48 2.78 2.86
180 170 160 150 140 130 120 110 100 90 80 70 60 50 40 30 20 10 0
f1 (ppm)

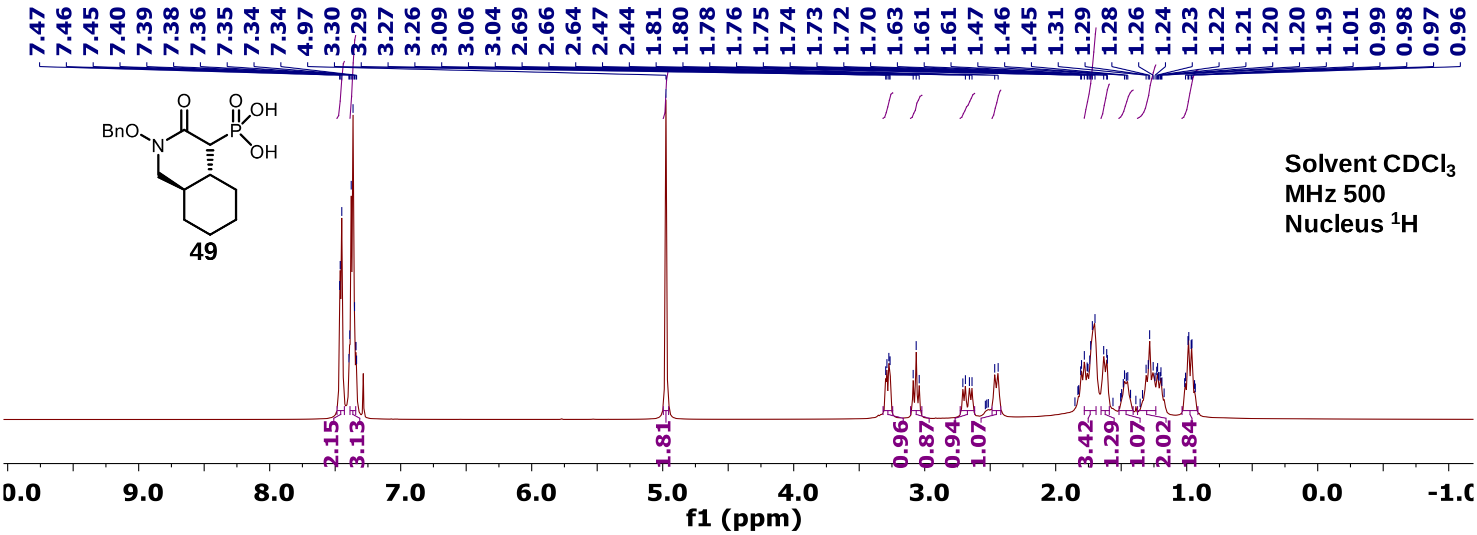
7.47 7.46 7.45 7.40 7.39 7.38 7.36 7.35 7.34 7.34 4.97 3.30 3.29 3.27 3.26 3.09 3.06 3.04 2.69 2.66 2.64 2.47 2.44 1.81 1.80 1.78 1.76 1.75 1.74 1.73 1.72 1.70 1.63 1.61 1.61 1.47 1.46 1.45 1.31 1.29 1.28 1.26 1.24 1.23 1.22 1.21 1.20 1.19 1.01 0.99 0.98 0.97 0.96
BnO
O
OH
OH
N
P
49
Solvent CDCl3
MHz 500
Nucleus 1H
2.15 3.13 1.81 0.96 0.87 0.94 1.07 3.42 1.29 1.07 2.02 1.84
f1 (ppm)

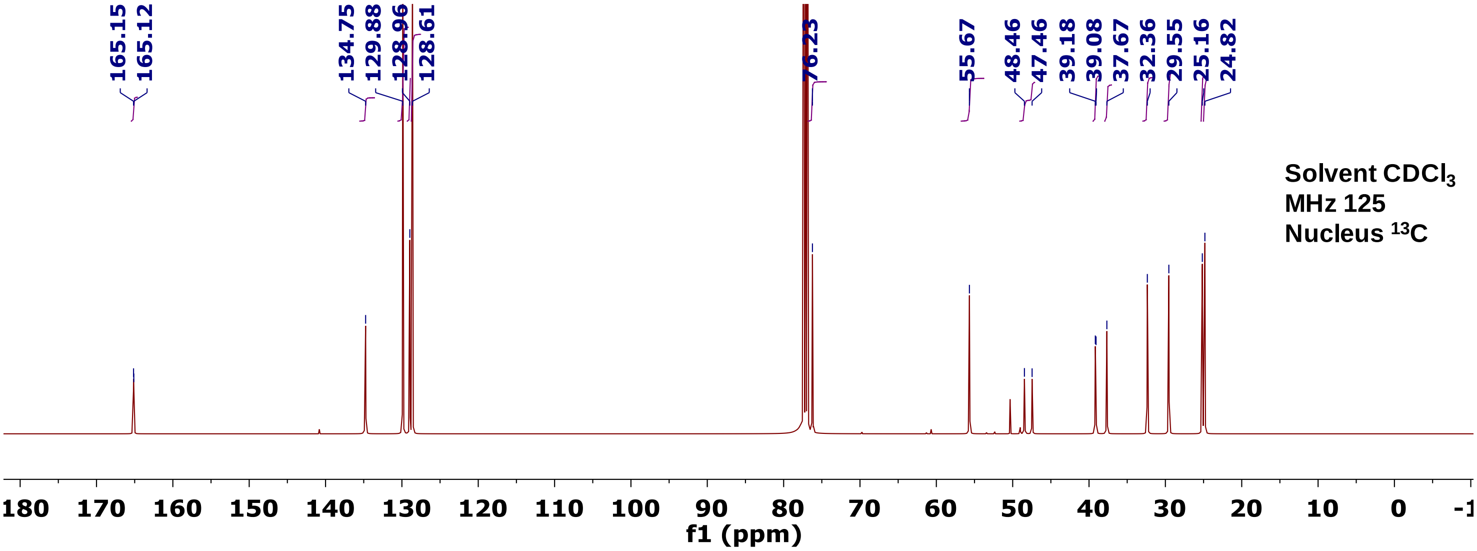
165.15 165.12 134.75 129.88 128.96 128.61 76.23 55.67 48.46 47.46 39.18 39.08 37.67 32.36 29.55 25.16 24.82
Solvent CDCl3
MHz 125
Nucleus 13C
f1 (ppm)

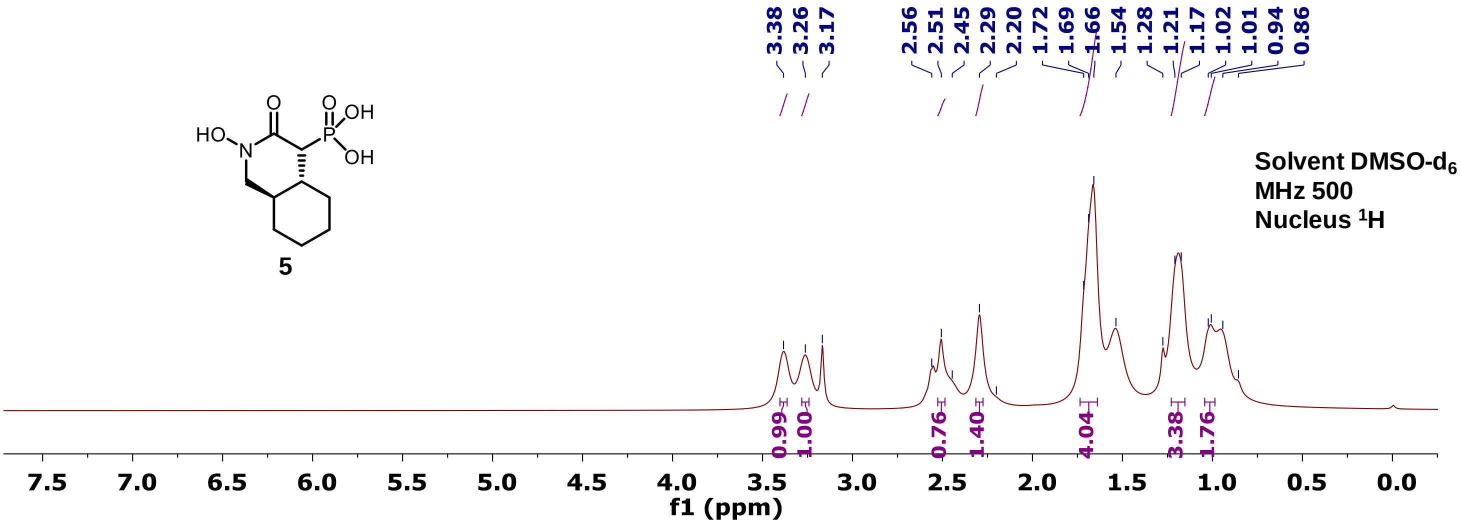
5
Solvent DMSO-d6
MHz 500
Nucleus 1H
f1 (ppm)

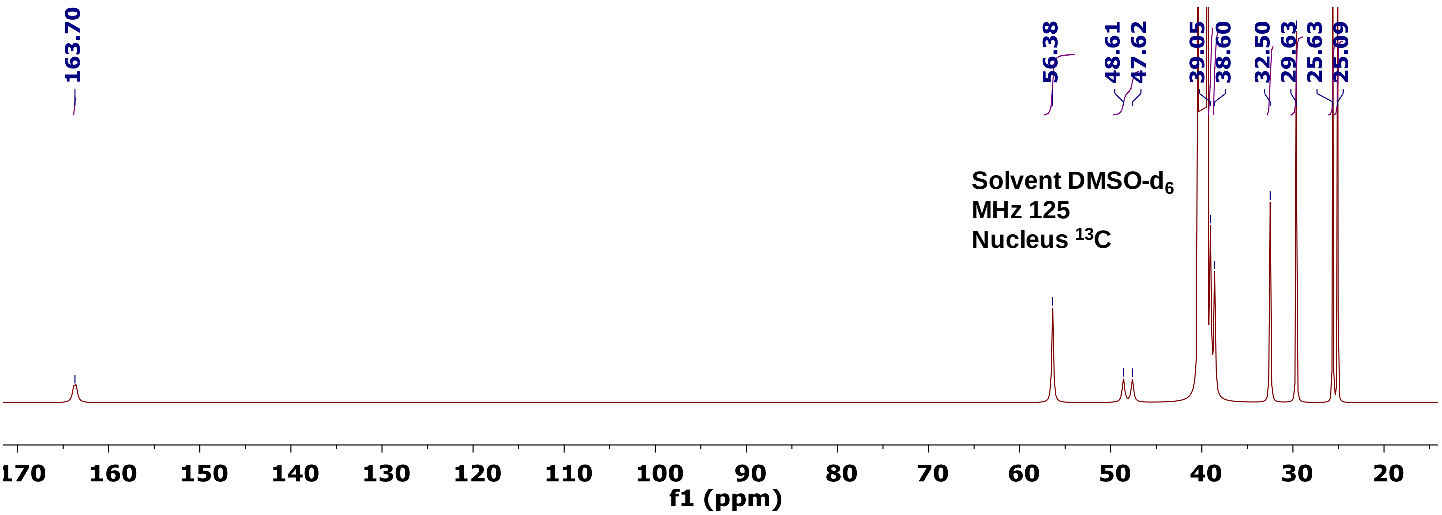
Solvent DMSO-d6
MHz 125
Nucleus 13C
f1 (ppm)

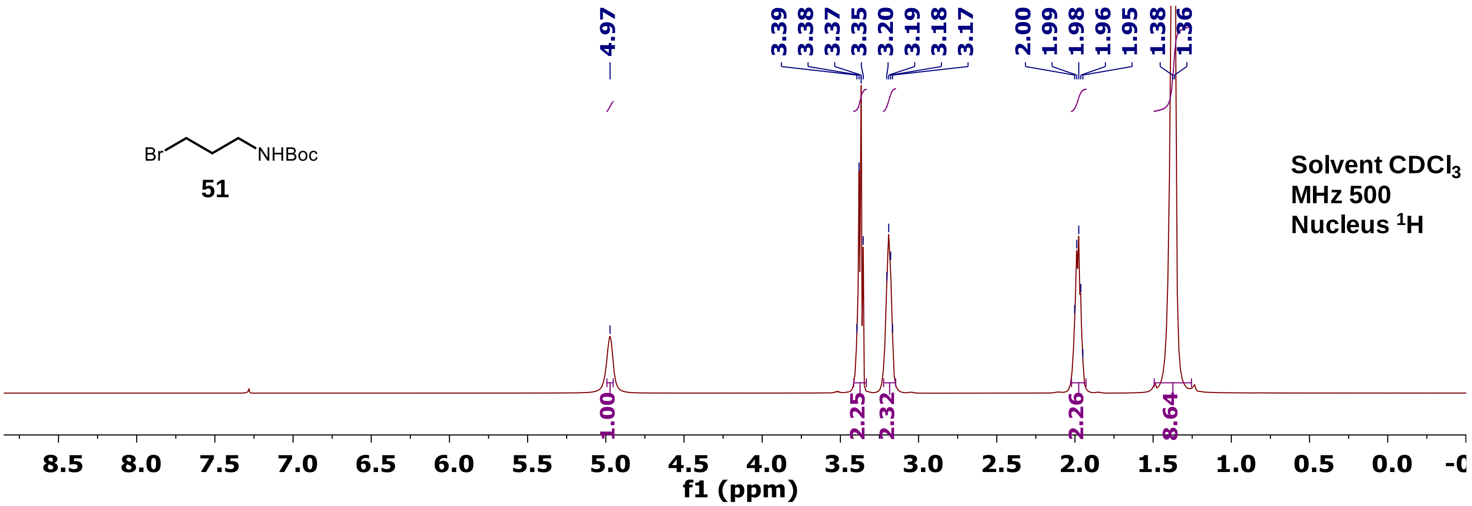

Br
NHBoc
51
Solvent CDCl3
MHz 500
Nucleus 1H
4.97
3.39 3.38 3.37 3.35 3.20 3.19 3.18 3.17
2.00 1.99 1.98 1.96 1.95 1.38 1.36
1.00 2.25 2.32 2.26 8.64
f1 (ppm)

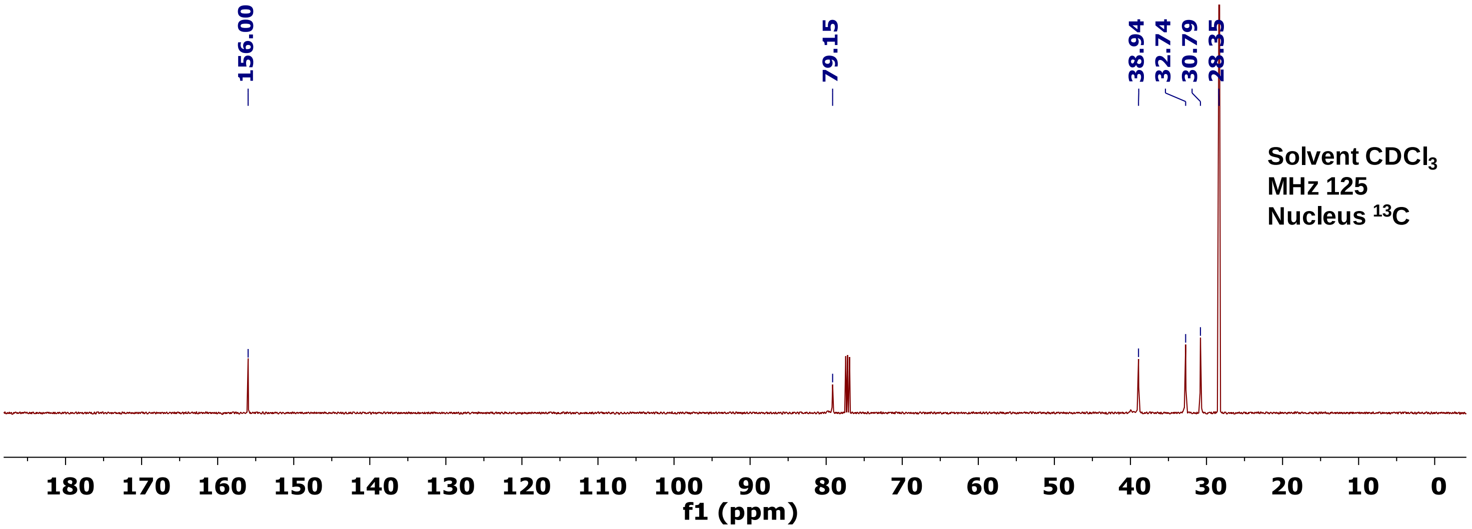

156.00
79.15
38.94 32.74 30.79 28.35
Solvent CDCl3
MHz 125
Nucleus 13C
f1 (ppm)

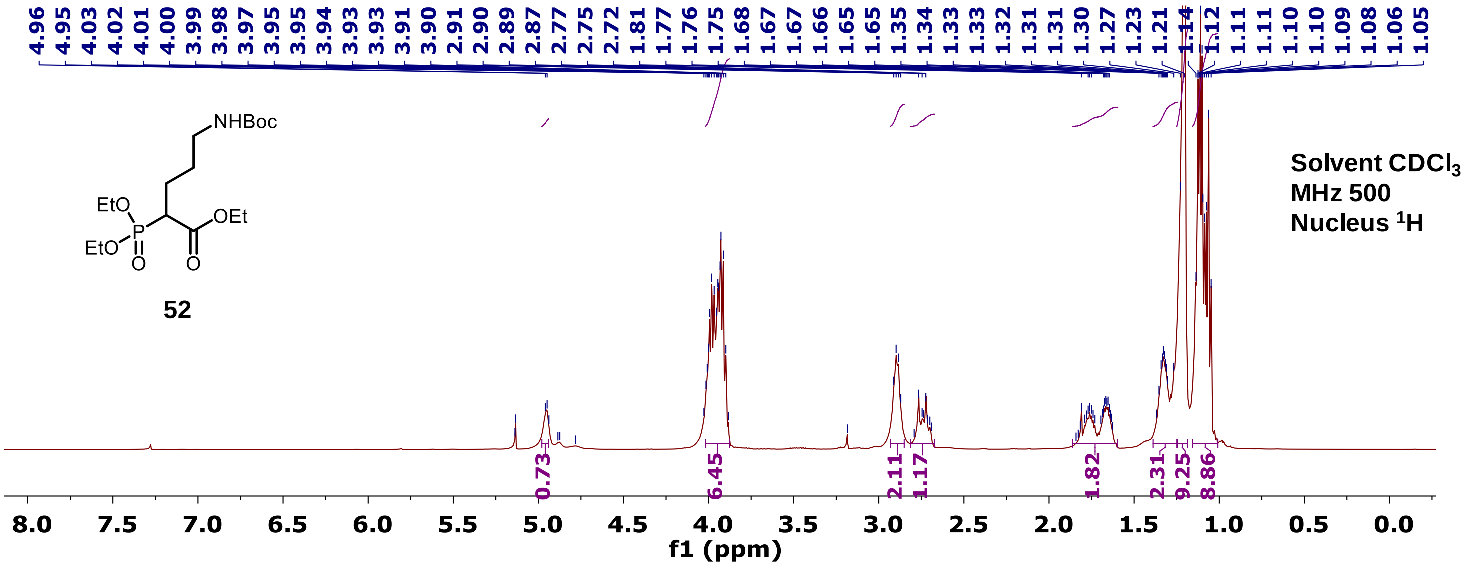
4.96 4.95 4.03 4.02 4.01 4.00 3.99 3.98 3.97 3.95 3.95 3.94 3.93 3.93 3.91 3.90 2.91 2.90 2.89 2.87 2.77 2.75 2.72 1.81 1.77 1.76 1.75 1.68 1.67 1.67 1.66 1.65 1.65 1.35 1.34 1.33 1.33 1.32 1.31 1.31 1.30 1.27 1.23 1.21 1.14 1.12 1.11 1.11 1.10 1.10 1.09 1.08 1.06 1.05
NHBoc
EtO
OEt
EtO
P
O
O
52
Solvent CDCl3
MHz 500
Nucleus 1H
0.73
6.45
2.11
1.17
1.82
2.31
9.25
8.86
8.0 7.5 7.0 6.5 6.0 5.5 5.0 4.5 4.0 3.5 3.0 2.5 2.0 1.5 1.0 0.5 0.0
f1 (ppm)

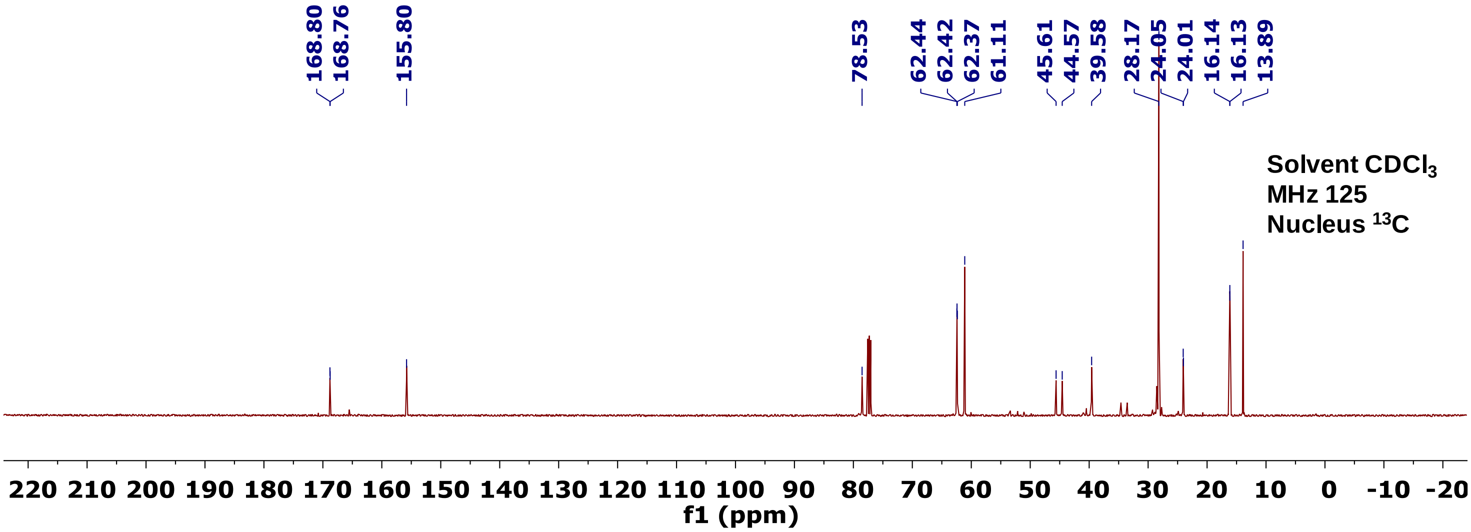
168.80 168.76 155.80 78.53 62.44 62.42 62.37 61.11 45.61 44.57 39.58 28.17 24.05 24.01 16.14 16.13 13.89
Solvent CDCl3
MHz 125
Nucleus 13C
220 210 200 190 180 170 160 150 140 130 120 110 100 90 80 70 60 50 40 30 20 10 0 -10 -20
f1 (ppm)

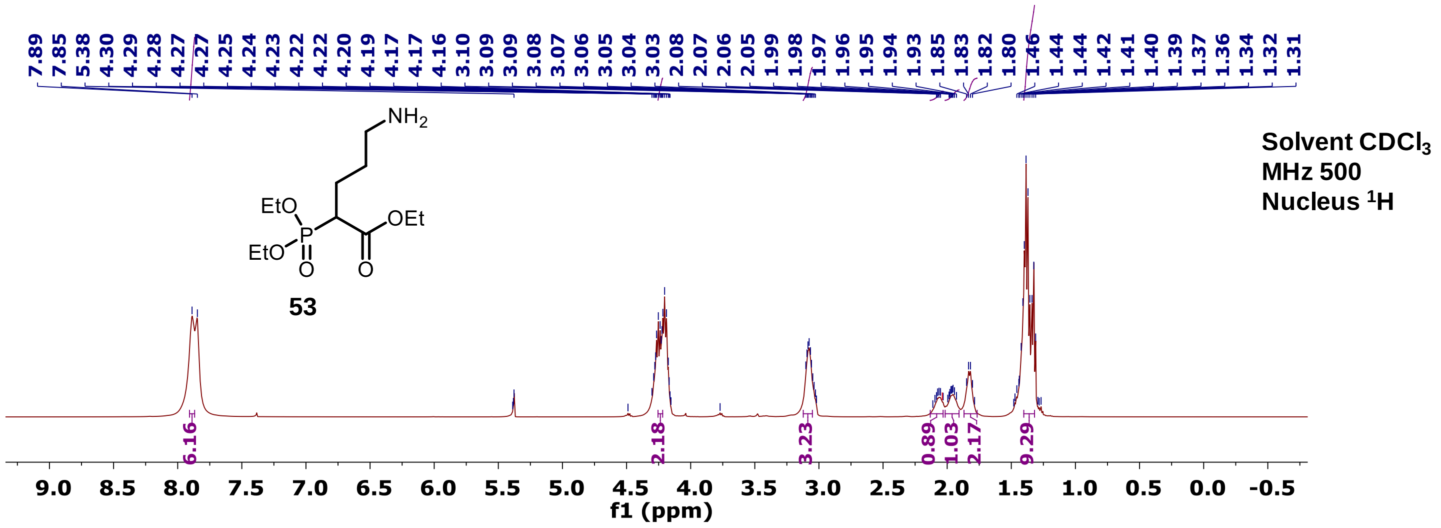
7.89 7.85 5.38 4.30 4.29 4.28 4.27 4.27 4.25 4.24 4.23 4.22 4.22 4.20 4.19 4.17 4.17 4.16 3.10 3.09 3.09 3.08 3.07 3.06 3.05 3.04 3.03 2.08 2.07 2.06 2.05 1.99 1.98 1.97 1.96 1.95 1.94 1.93 1.85 1.83 1.82 1.80 1.46 1.44 1.44 1.42 1.41 1.40 1.39 1.37 1.36 1.34 1.32 1.31
NH2
EtO
OEt
EtO
P
O
O
53
Solvent CDCl3
MHz 500
Nucleus 1H
6.16
2.18
3.23
0.89
1.03
2.17
9.29
9.0 8.5 8.0 7.5 7.0 6.5 6.0 5.5 5.0 4.5 4.0 3.5 3.0 2.5 2.0 1.5 1.0 0.5 0.0 -0.5
f1 (ppm)

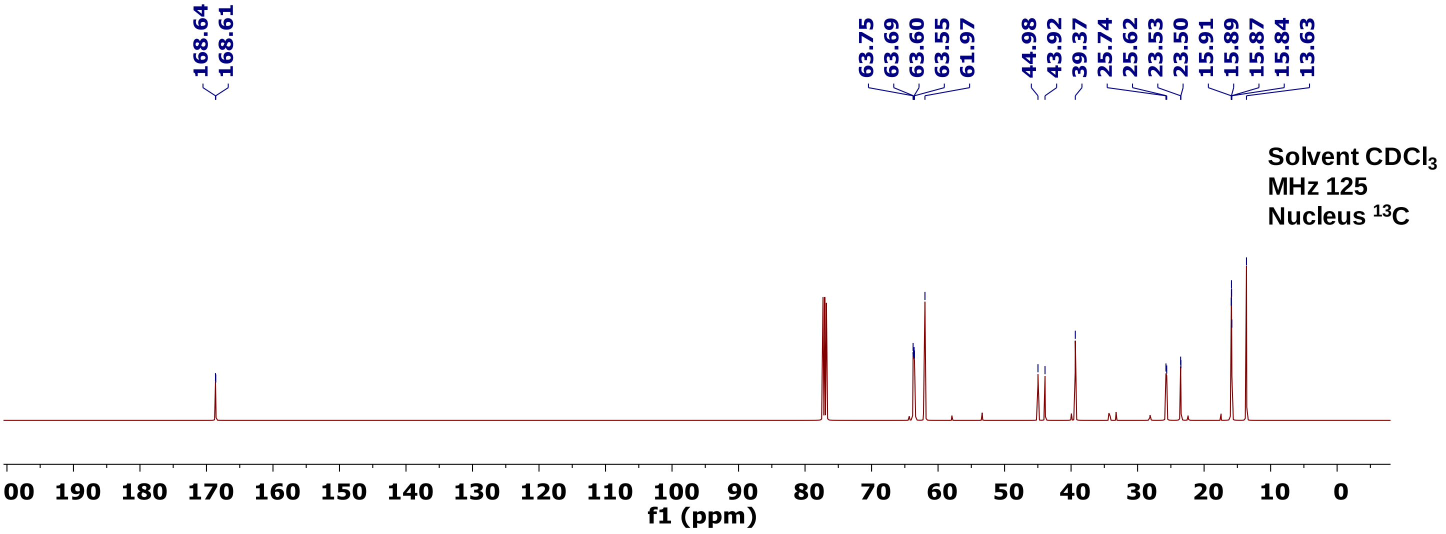
168.64 168.61
63.75 63.69 63.60 63.55 61.97
44.98 43.92 39.37 25.74 25.62 23.53 23.50 15.91 15.89 15.87 15.84 13.63
Solvent CDCl3
MHz 125
Nucleus 13C
00 190 180 170 160 150 140 130 120 110 100 90 80 70 60 50 40 30 20 10 0
f1 (ppm)

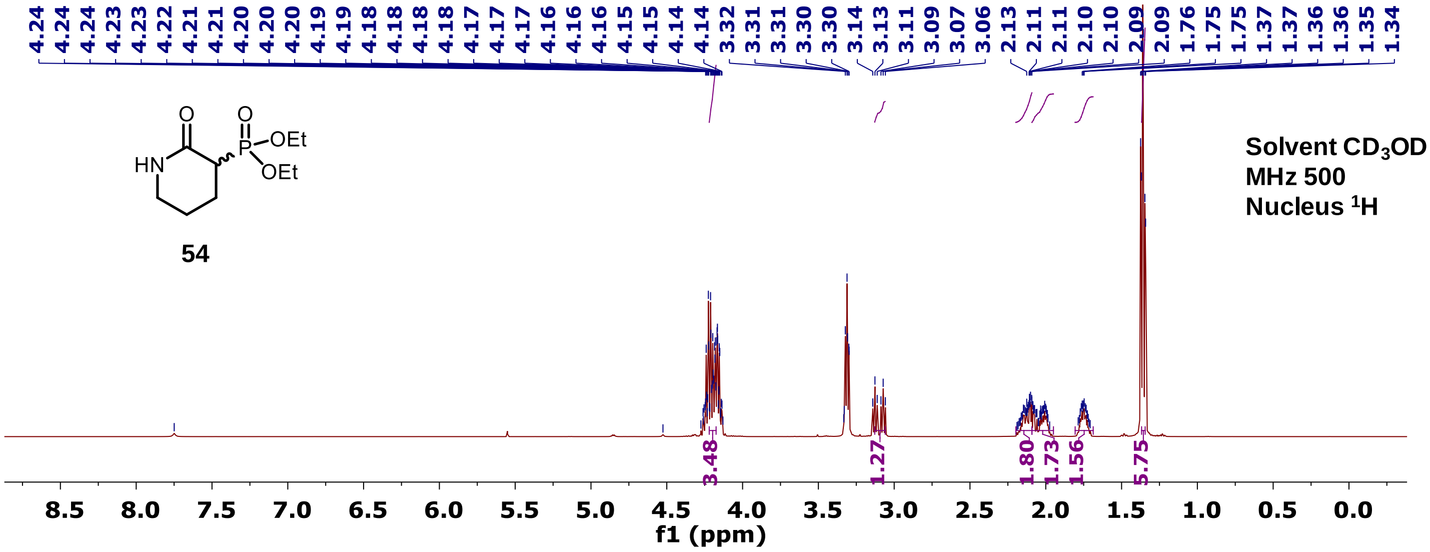
4.24 4.24 4.24 4.23 4.23 4.22 4.21 4.21 4.20 4.20 4.20 4.19 4.19 4.18 4.18 4.18 4.18 4.17 4.17 4.17 4.16 4.16 4.16 4.15 4.15 4.14 4.14 3.32 3.31 3.31 3.30 3.30 3.14 3.13 3.11 3.09 3.07 3.06 2.13 2.11 2.11 2.10 2.10 2.09 2.09 1.76 1.75 1.75 1.37 1.37 1.36 1.36 1.35 1.34
O
O
P
OEt
OEt
HN
54
Solvent CD$_3$OD
MHz 500
Nucleus ^{1}H
3.48
1.27
1.80
1.73
1.56
5.75
8.5 8.0 7.5 7.0 6.5 6.0 5.5 5.0 4.5 4.0 3.5 3.0 2.5 2.0 1.5 1.0 0.5 0.0
f1 (ppm)

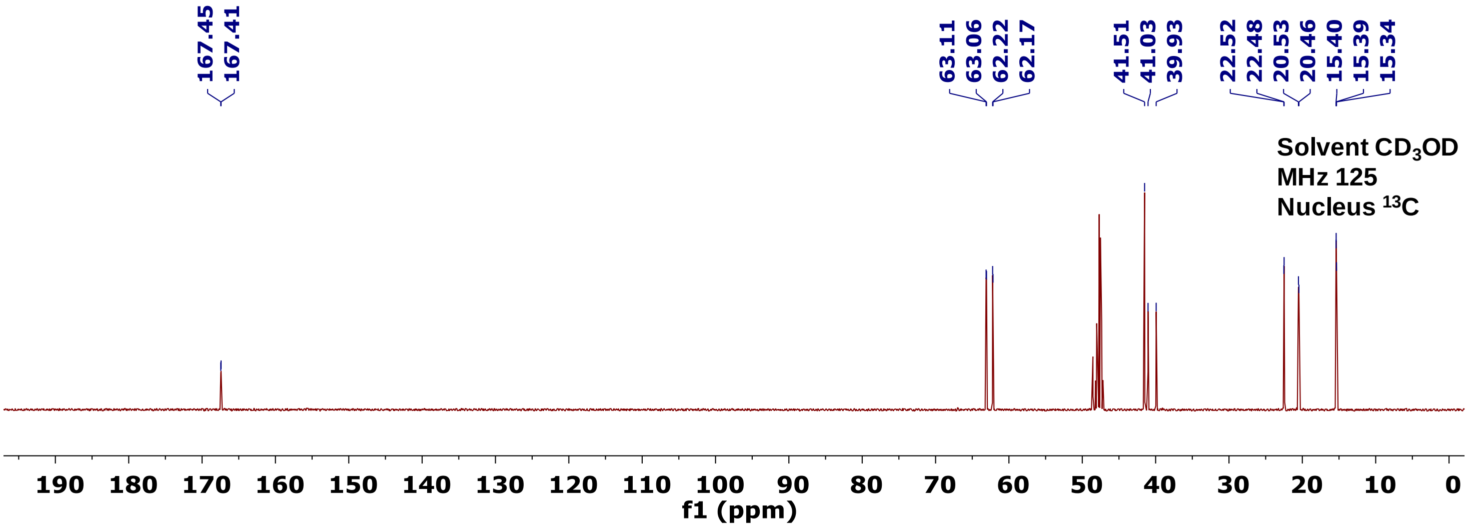
167.45 167.41
63.11 63.06 62.22 62.17
41.51 41.03 39.93
22.52 22.48 20.53 20.46 15.40 15.39 15.34
Solvent CD$_3$OD
MHz 125
Nucleus ^{13}C
190 180 170 160 150 140 130 120 110 100 90 80 70 60 50 40 30 20 10 0
f1 (ppm)

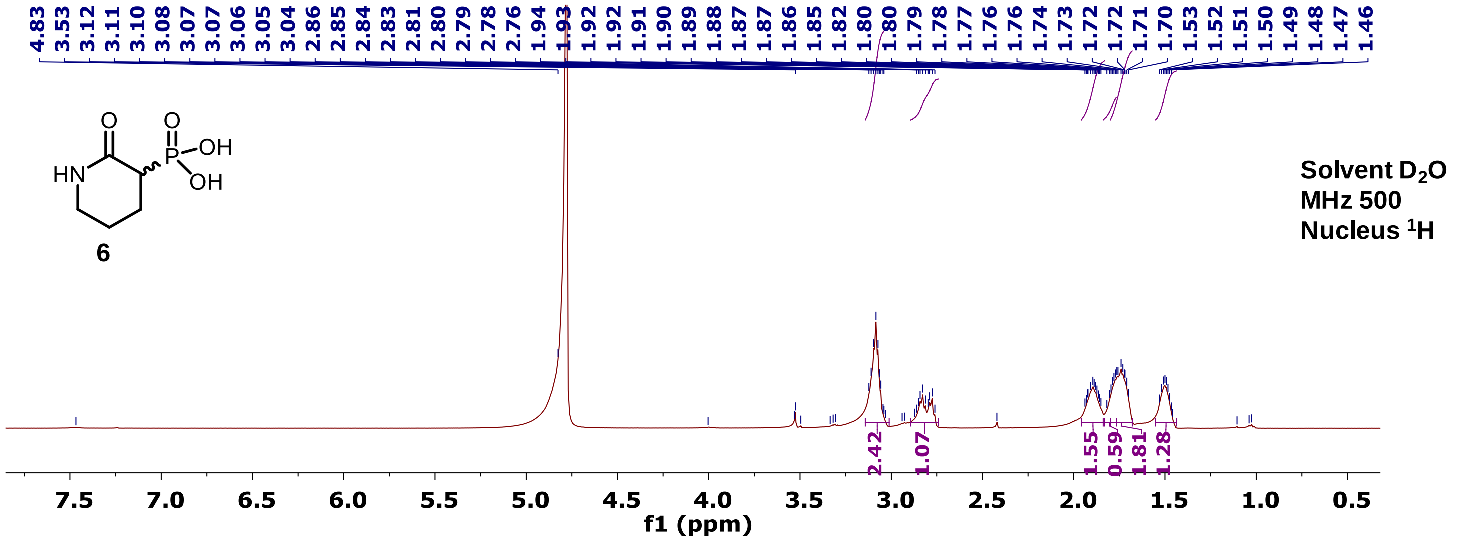
4.83 3.53 3.12 3.11 3.10 3.08 3.07 3.07 3.06 3.05 3.04 2.86 2.85 2.84 2.83 2.81 2.80 2.79 2.78 2.76 1.94 1.93 1.92 1.92 1.91 1.90 1.89 1.88 1.87 1.87 1.86 1.85 1.82 1.80 1.80 1.79 1.78 1.77 1.76 1.76 1.74 1.73 1.72 1.72 1.71 1.70 1.53 1.52 1.51 1.50 1.49 1.48 1.47 1.46
O
O
P-OH
OH
HN
6
Solvent D_2O
MHz 500
Nucleus ^1H
2.42 1.07 1.55 0.59 1.81 1.28
7.5 7.0 6.5 6.0 5.5 5.0 4.5 4.0 3.5 3.0 2.5 2.0 1.5 1.0 0.5
f1 (ppm)

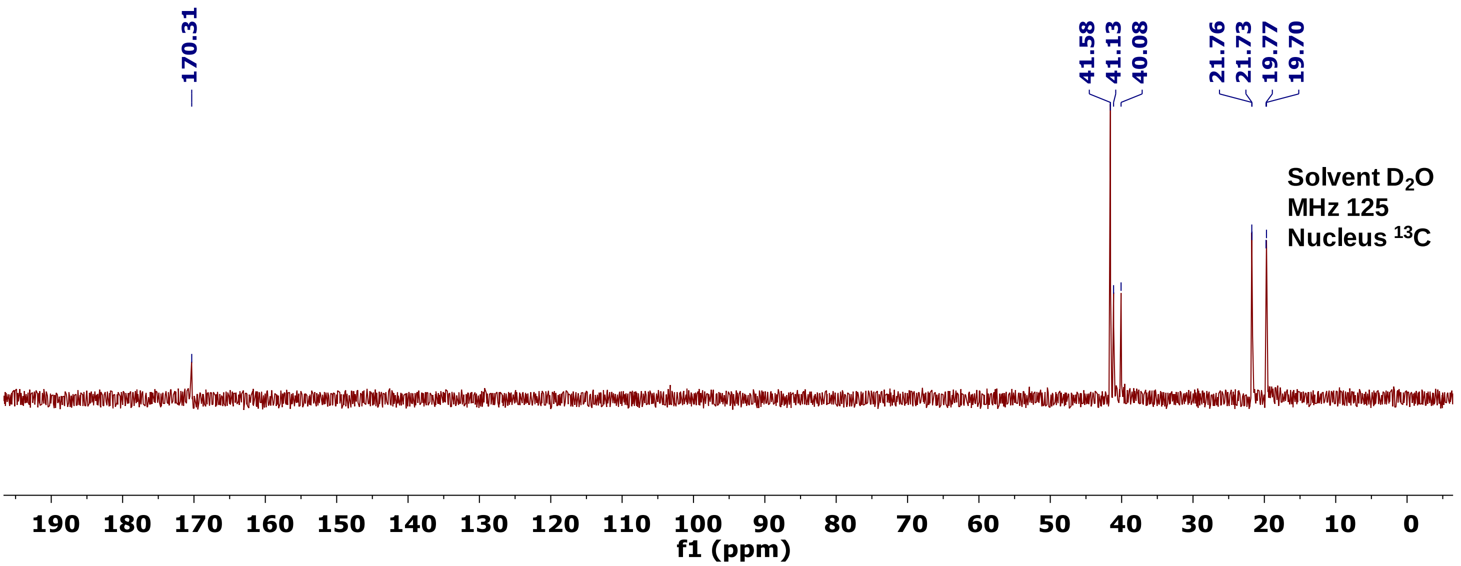
170.31
41.58 41.13 40.08
21.76 21.73 19.77 19.70
Solvent D_2O
MHz 125
Nucleus ^{13}C
190 180 170 160 150 140 130 120 110 100 90 80 70 60 50 40 30 20 10 0
f1 (ppm)

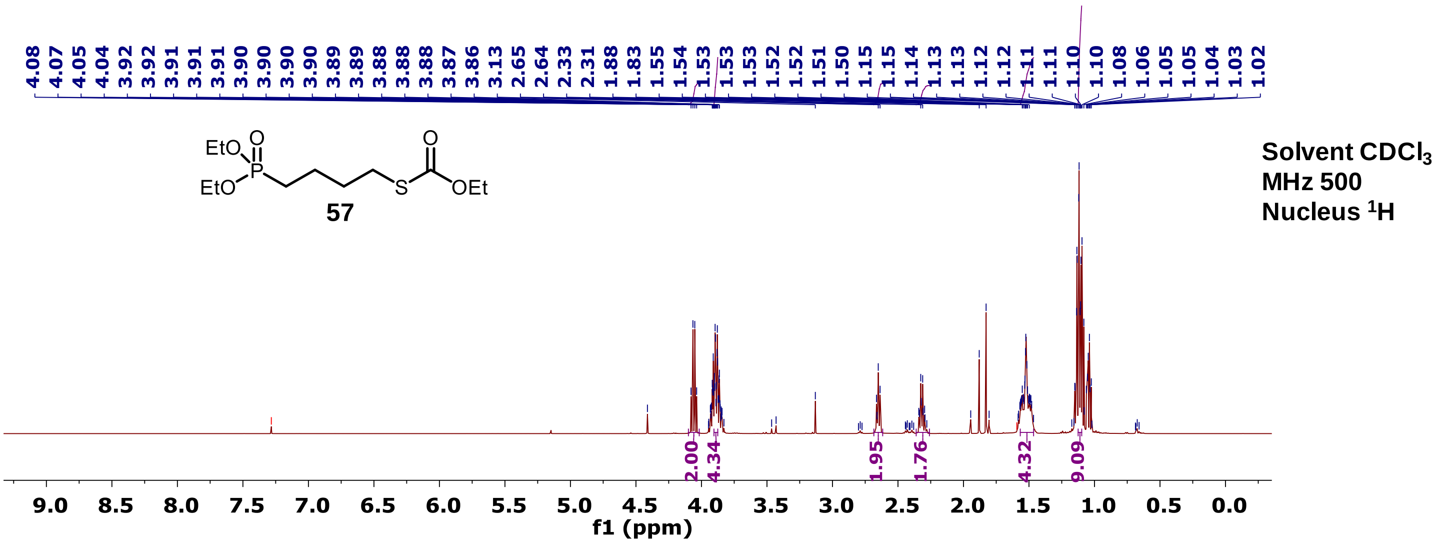
4.08 4.07 4.05 4.04 3.92 3.92 3.91 3.91 3.91 3.90 3.90 3.90 3.89 3.89 3.88 3.88 3.88 3.87 3.86 3.13 2.65 2.64 2.33 2.31 1.88 1.83 1.55 1.54 1.53 1.53 1.53 1.52 1.52 1.51 1.50 1.15 1.15 1.14 1.13 1.13 1.12 1.12 1.11 1.11 1.10 1.10 1.08 1.06 1.05 1.05 1.04 1.03 1.02
EtO
EtO
P
O
S
O
OEt
57
Solvent $CDCl_3$
MHz 500
Nucleus ^1H
2.00
4.34
1.95
1.76
4.32
9.09
9.0 8.5 8.0 7.5 7.0 6.5 6.0 5.5 5.0 4.5 4.0 3.5 3.0 2.5 2.0 1.5 1.0 0.5 0.0
f1 (ppm)
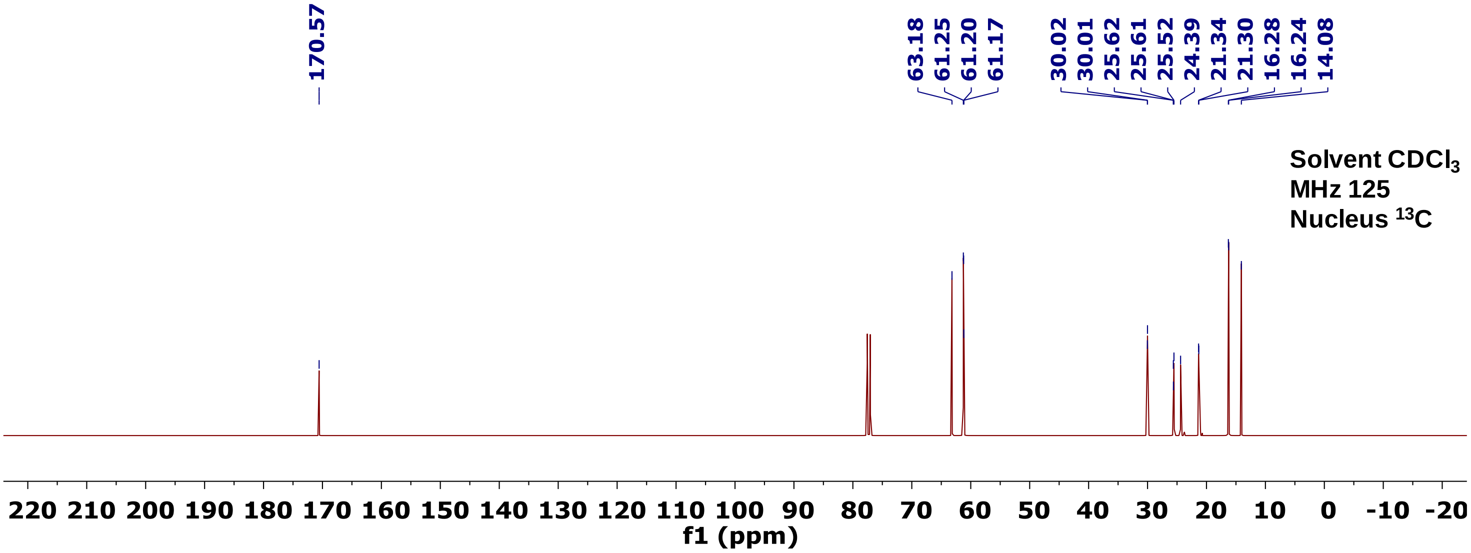
170.57
63.18 61.25 61.20 61.17
30.02 30.01 25.62 25.61 25.52 24.39 21.34 21.30 16.28 16.24 14.08
Solvent $CDCl_3$
MHz 125
Nucleus ^{13}C
220 210 200 190 180 170 160 150 140 130 120 110 100 90 80 70 60 50 40 30 20 10 0 -10 -20
f1 (ppm)

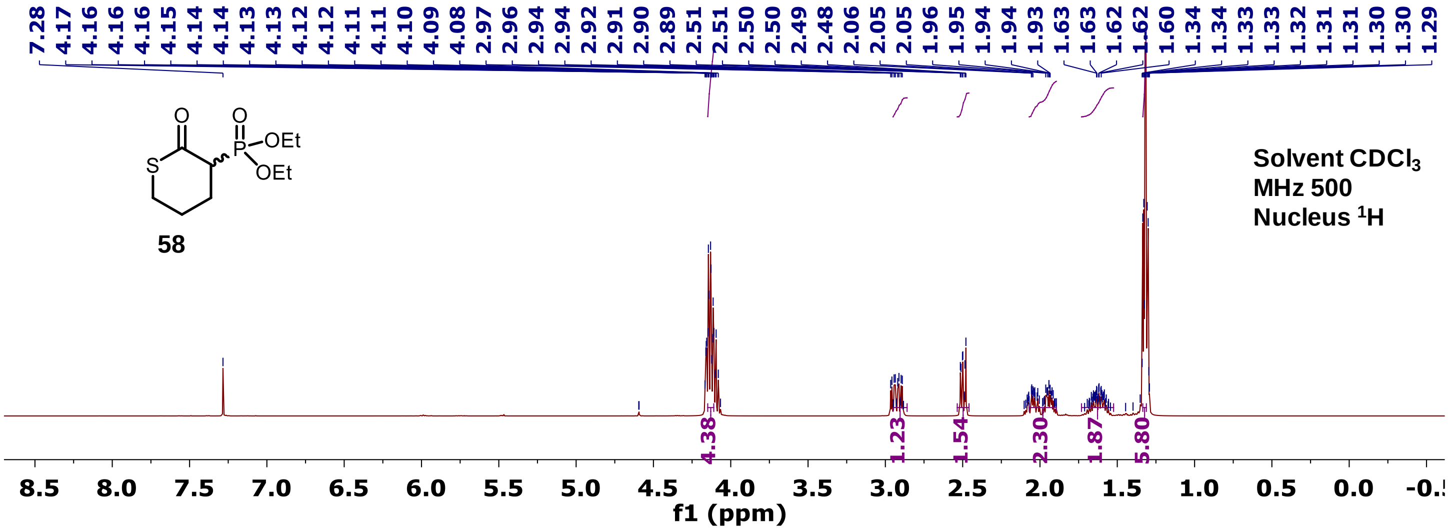
7.28 4.17 4.16 4.16 4.16 4.15 4.14 4.14 4.13 4.13 4.12 4.12 4.11 4.11 4.10 4.09 4.08 2.97 2.96 2.94 2.94 2.92 2.91 2.90 2.89 2.51 2.51 2.50 2.50 2.49 2.48 2.06 2.05 2.05 1.96 1.95 1.94 1.94 1.93 1.63 1.63 1.62 1.62 1.60 1.34 1.34 1.33 1.33 1.32 1.31 1.31 1.30 1.30 1.29
O
O
P
OEt
OEt
S
58
Solvent $CDCl_3$
MHz 500
Nucleus ^{1}H
4.38
1.23
1.54
2.30
1.87
5.80
8.5 8.0 7.5 7.0 6.5 6.0 5.5 5.0 4.5 4.0 3.5 3.0 2.5 2.0 1.5 1.0 0.5 0.0
f1 (ppm)

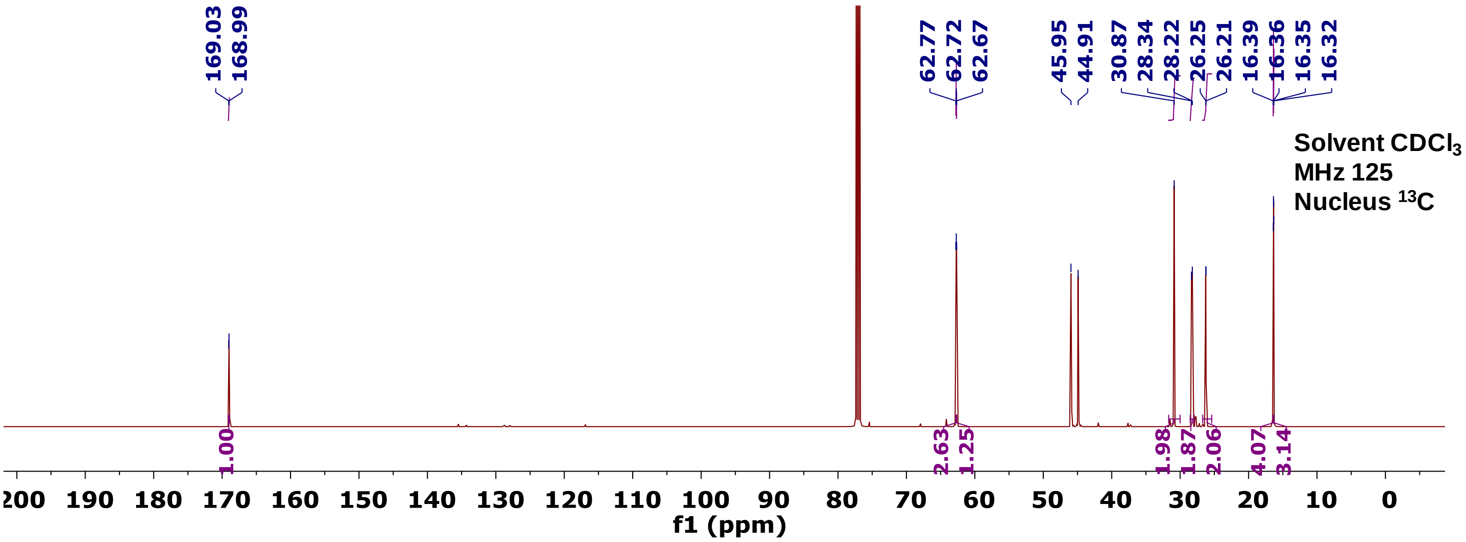
169.03 168.99
62.77 62.72 62.67
45.95 44.91
30.87 28.34 28.22 26.25 26.21
16.39 16.36 16.35 16.32
Solvent $CDCl_3$
MHz 125
Nucleus ^{13}C
1.00
2.63
1.25
1.98
1.87
2.06
4.07
3.14
190 180 170 160 150 140 130 120 110 100 90 80 70 60 50 40 30 20 10 0
f1 (ppm)

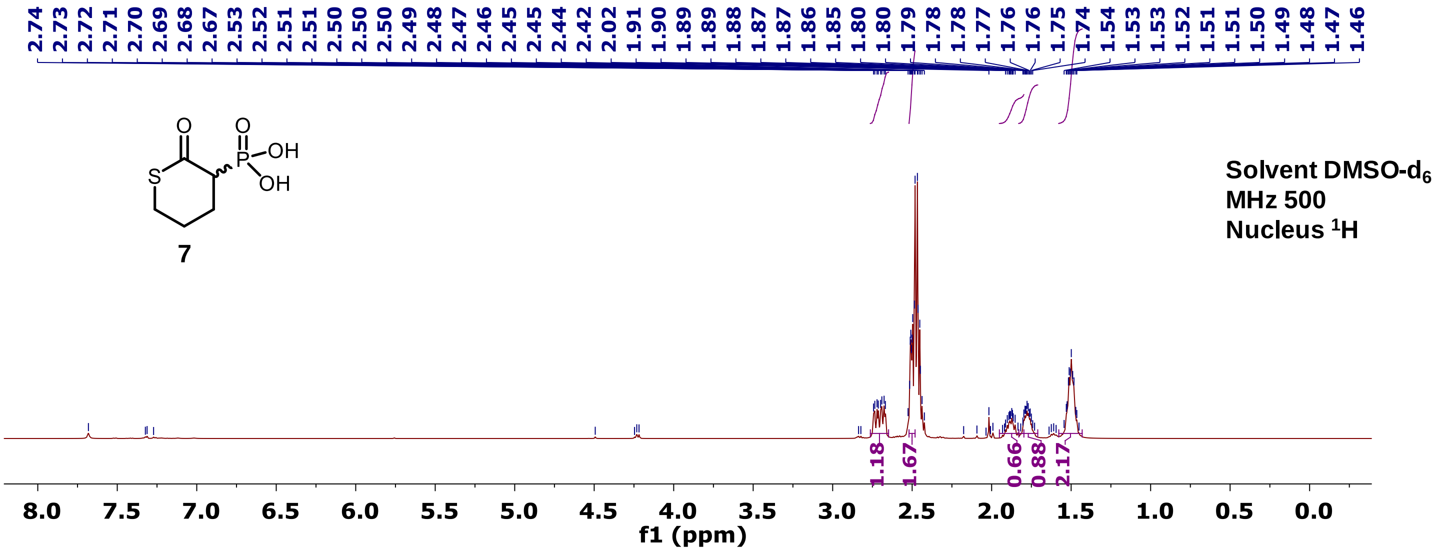
2.74 2.73 2.72 2.71 2.70 2.69 2.68 2.67 2.53 2.52 2.51 2.51 2.50 2.50 2.50 2.49 2.48 2.47 2.46 2.45 2.45 2.44 2.42 2.02 1.91 1.90 1.89 1.89 1.88 1.87 1.87 1.86 1.85 1.80 1.80 1.79 1.78 1.77 1.76 1.76 1.75 1.74 1.54 1.53 1.53 1.52 1.51 1.51 1.50 1.49 1.48 1.47 1.46
O
O
P
OH
S
OH
7
Solvent DMSO-d6
MHz 500
Nucleus 1H
1.18
1.67
0.66
0.88
2.17
8.0 7.5 7.0 6.5 6.0 5.5 5.0 4.5 4.0 3.5 3.0 2.5 2.0 1.5 1.0 0.5 0.0
f1 (ppm)

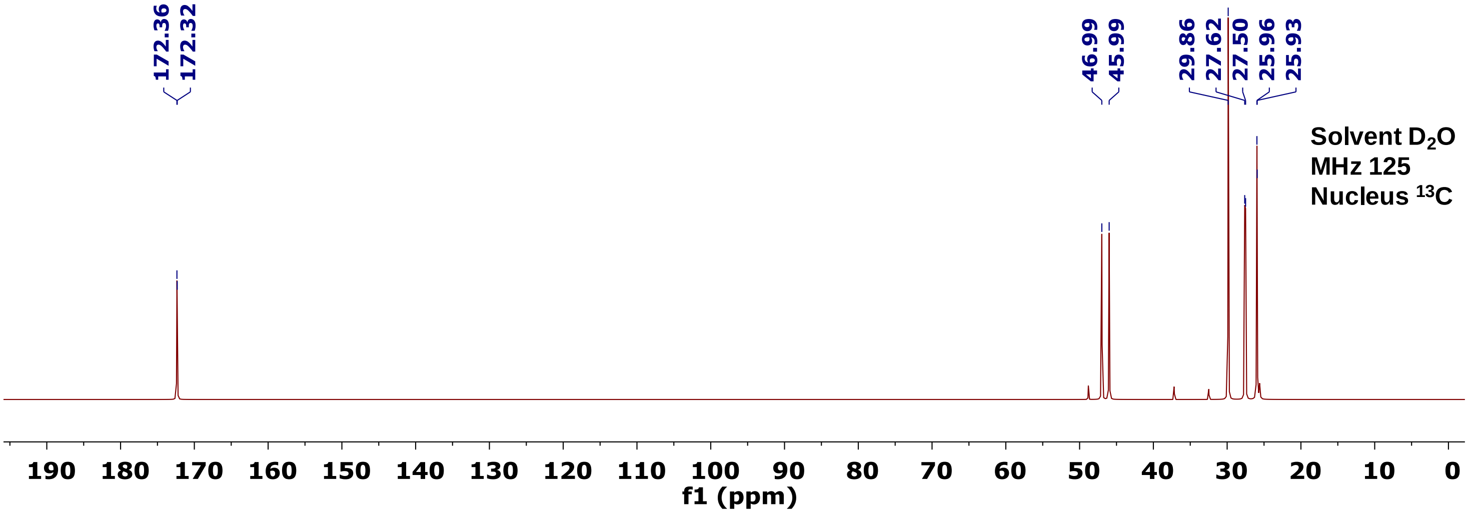
172.36
172.32
46.99
45.99
29.86
27.62
27.50
25.96
25.93
Solvent D2O
MHz 125
Nucleus 13C
190 180 170 160 150 140 130 120 110 100 90 80 70 60 50 40 30 20 10 0
f1 (ppm)
